# Photocatalytic Generation of Divalent Lanthanide Reducing Agents

**DOI:** 10.1021/jacs.3c07508

**Published:** 2023-10-05

**Authors:** Monika Tomar, Rohan Bhimpuria, Daniel Kocsi, Anders Thapper, K. Eszter Borbas

**Affiliations:** Department of Chemistry, Ångström Laboratory, Uppsala University, Uppsala 75120, Sweden

## Abstract

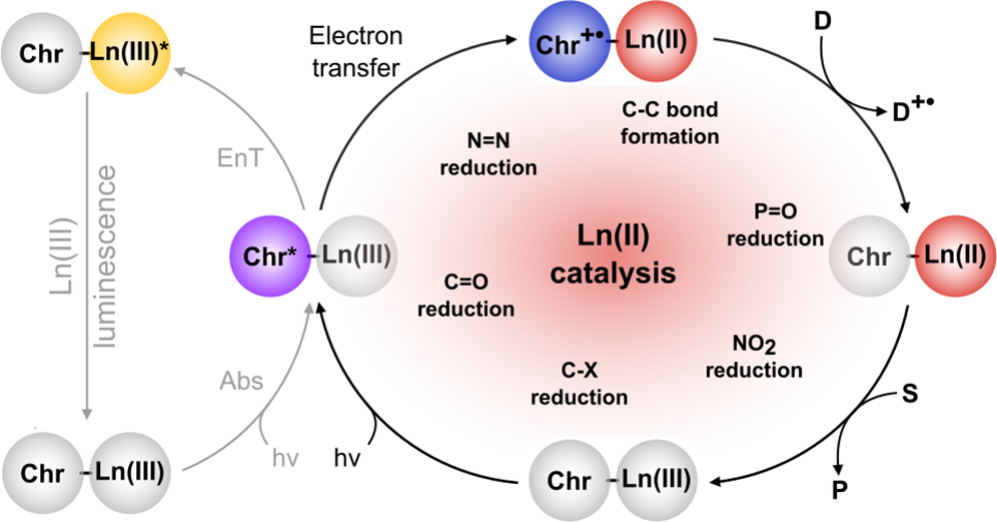

Divalent lanthanide (Ln) compounds are excellent reducing agents with unique reactivity profiles. These reagents are typically used in superstoichiometric amounts, often in combination with harmful additives. Reactions catalytic in Ln(II) reagents that retain the reactivity and selectivity of the stoichiometric transformations are currently lacking due to the absence of effective and selective methods to form reactive Ln(II) species from stable precursors. Here, active Ln(II) is generated from a Ln(III) precursor through reduction by a photoexcited coumarin or carbostyril chromophore, which, in turn, is regenerated by a sacrificial reductant. The reductant can be metallic (Zn) or organic (amines) and can be used in strictly stoichiometric amounts. A broad range of reactions, including C–halogen, C=C, C=X (X = O, N), P=O, and N=N reductions, as well as C–C, C–X (X = N, S, P), and N–N couplings were readily carried out in yields and selectivities comparable to or better than those afforded by the analogous stoichiometric transformations. The reaction outcomes could be altered by changing the ligand or the lanthanide or through the addition of environmentally benign additives (e.g., water). EPR spectroscopy supported the formation of both Ln(II) and oxidized chromophore intermediates. Taken together, these results establish photochemical Ln(II) generation as a powerful strategy for rendering Ln(II)-mediated reactions catalytic.

## Introduction

The unique physical and chemical properties of lanthanides (Ln) make them indispensable for applications ranging from renewable energy production to bioimaging and catalysis.^1−4^ Ln(II) ions have reducing capabilities that range from moderate (Sm(II), Eu(II), or Yb(II)) to powerful (Tm(II), Dy(II), Nd(II)).^5−9^ SmI_2_ is widely used in academic research as a chemoselective reagent in challenging settings that necessitate the preservation of reactive functional groups and stereochemical information.^10−12^ SmI_2_ often needs to be deployed in combination with superstoichiometric amounts of toxic additives, e.g., highly carcinogenic hexamethylphosphoramide (HMPA). Lanthanides, like many transition metals, are expensive, and their mining causes significant environmental damage.^13,14^ Thus, there is an urgent need to develop protocols that are catalytic in Ln(II).^15^ A handful of examples have demonstrated the possibility of Ln(II) catalysis by regenerating Ln(II) photochemically with rhodamine 6G and an amine,^16^ with Zn,^17^ or electrochemically;^18^ sacrificial reductants were used in large excess.^18^ Crucially, existing examples have transformed only a narrow range of substrates lacking potentially sensitive functional groups and have thus not demonstrated the retention of the most attractive features of the stoichiometric reactions: high functional group tolerance, tunability of reducing power, and versatility.

**Figure 1:**
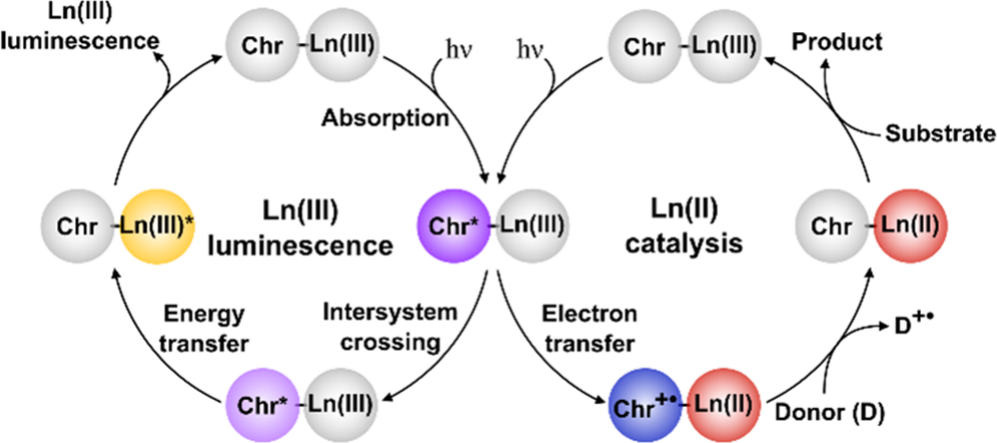
Proposed pathway for Ln(II) generation (right cycle) as an alternative to Ln(III) luminescence sensitization (left cycle). The order of the substrate reduction and chromophore (**Chr**) regeneration steps may be inverted.

In photoredox catalysis, excited-state photosensitizers initiate chemical transformations.^19−22^ Catalysts are often based on organic dyes^19^ or metal complexes, mainly transition metals;^23,24^ they have short (ns) excited-state lifetimes, and their reducing power is limited to a narrow range. Ln(II)-based reductants have oxidation potentials that cover a broad range from −0.4 to −3.9 V; however, lanthanides have been underutilized as photocatalysts. Most Ln(III) ions can luminesce when sensitized by a light-harvesting chromophore (Figure 1, left cycle). Direct Ln(III) excitation is inefficient, as the 4f–4f transitions are Laporte-forbidden.^25^ The photoexcited chromophore is a powerful reductant. In the case of the most reducible Yb(III)^26^ (*E*_1/2_(Yb(III)/Yb(II), YbCl_3_) = −1.1 V vs NHE) and Eu(III)^27^ (*E*_1/2_(Eu(III)/Eu(II), EuCl_3_) = −0.42 V vs NHE), Ln(III) reduction competes with Ln(III) luminescence sensitization via energy transfer (Figure 1, right cycle).^27,28^ Photochemical generation of Ln(II) from a Ln(III) precursor followed by reoxidation of the Ln(II) by a substrate and the reduction of the oxidized chromophore with a sacrificial reductant enables the use of catalytic amounts of lanthanide reagent. While such a lanthanide turnover has been demonstrated, the reported procedure relies on strongly reducing Dy(II), Nd(II), or Tm(II) (*E*_1/2_(Ln(III)/Ln(II) = −2.5, −2.6, – 2.3 V vs NHE, respectively^29^));^16^ the authors noted that using the much more accessible Ln(III) precursors instead of the Ln(II) ones afforded the product in lower yields.^16^ We hypothesized that a practical protocol for the in situ generation of Ln(II) from Ln(III) could be obtained by combining the chromophore with a multidentate ligand.

Here, we show that in situ-generated Ln(II) (Ln = Eu, Sm, Dy) species can reduce benzylic, allylic, and aryl halides efficiently and affect a broad range of functional group interconversions previously achieved only by (super)stoichiometric amounts of SmI_2_. Reductions were successfully coupled to a variety of C–C, C–N, C–P, C–S, and N–N bond formations, as well as to carbocycle and heterocycle syntheses. Taken together, these practical catalytic processes reproduce and even surpass stoichiometric Ln(II)-mediated reductions in scope, functional group tolerance, and versatility and are thus viable alternatives to the attractive, but resource-intensive stoichiometric transformations.

## Results and Discussion

### Catalyst Design and Reaction Development

**Chart 1:**
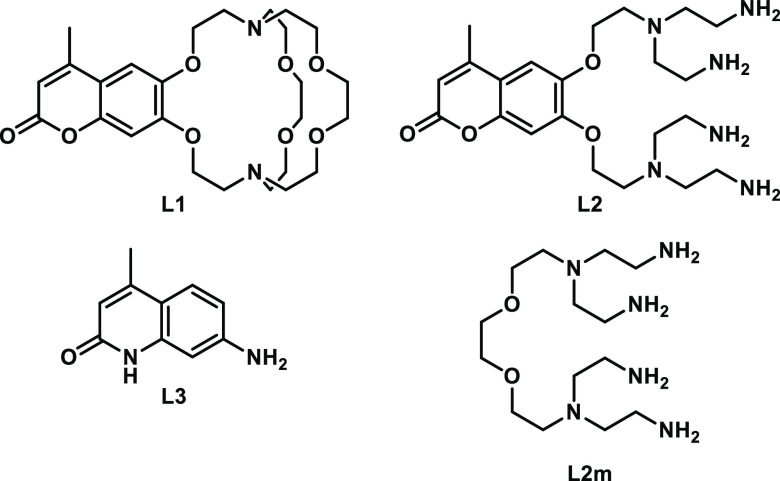
Ligands **L1**, **L2**, and **L3** used for photocatalysisa

We designed three catalysts (**LnL1**–**LnL3**) (Ln = Eu, Sm, Gd, Dy) with different coordination environments (Chart 1). **L1** and **L2** were expected to strongly bind Ln(III) and Ln(II) and thus yield stable, long-lived catalysts. **L3** relies on weak interactions between Eu(III) and 7-aminocarbostyril, and most lanthanide coordination sites are occupied by labile solvent molecules. **L1**–**L3** incorporate a light-harvesting heterocycle, either a 6,7-oxycoumarin (**L1**, **L2**), or a 7-aminocarbostyril (**L3**), which allows for excitation with UV and visible light. Structurally **L1** is related to the Allen cryptands.^17,30^**L2** is a flexible and synthetically more accessible open-chain analogue of **L1**. **L1** and **L2** were expected to stabilize Ln(II) compared to the solvated ion. The excited-state reduction potentials of the chromophores are < –1.88 V (vs Fc^+^/Fc, Page S78), which is sufficiently negative to reduce Eu(III) and Sm(III) and thus yield the reactive Ln(II) species. The catalyst design described here is highly modular. Catalyst reactivity can be tuned through the ligand that regulates Ln(II) stability and substrate access, through the chromophore excited state and the lanthanide.

a **LnL** could be prepared in advance, or generated in situ. **L2m** is a model ligand lacking the **Chr** unit.

**Figure 2:**
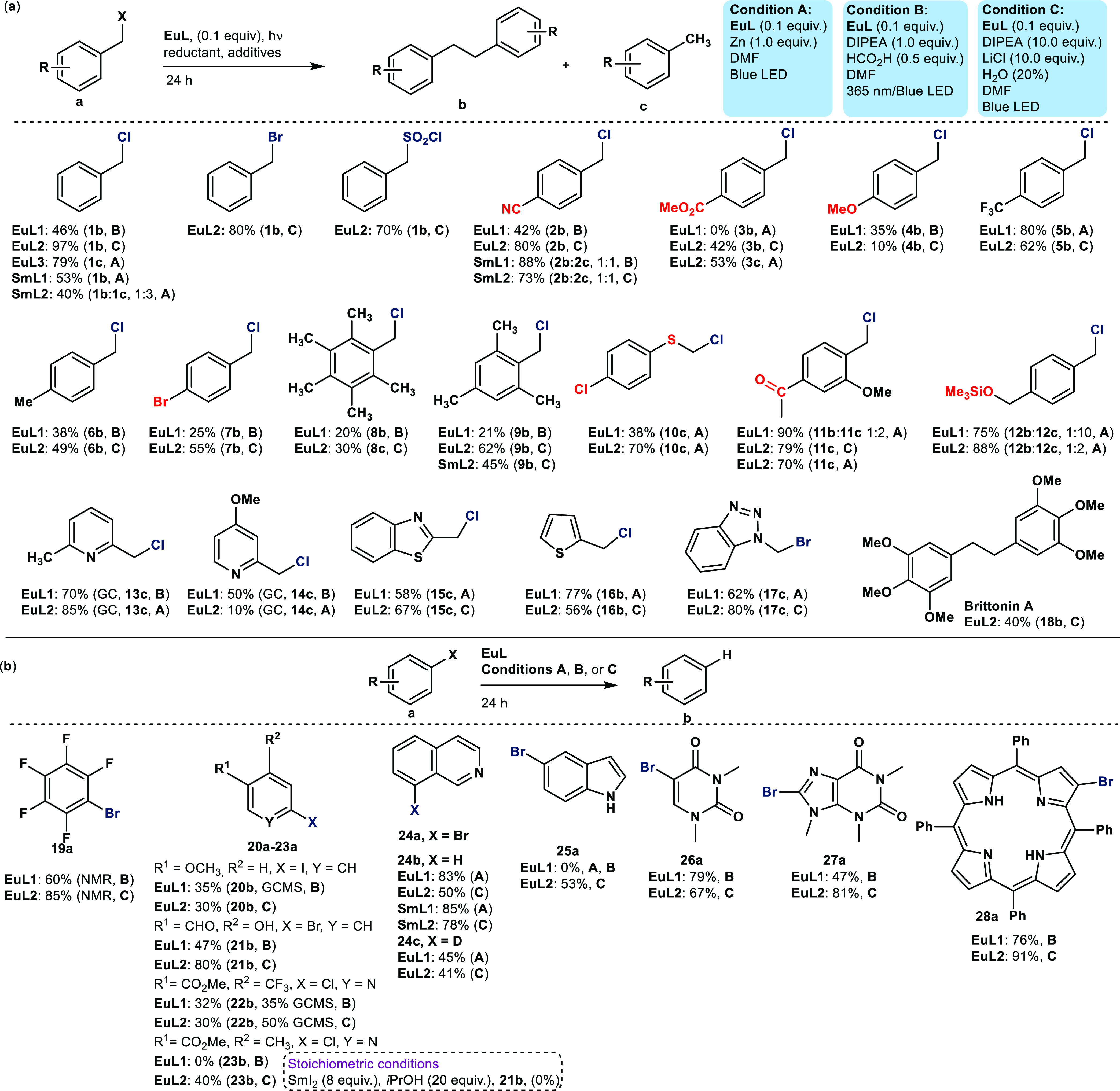
Substrate scopes of benzylic (a) and aryl halide (b) reduction reactions. Reduced functionalities are shown in blue, and potentially sensitive functional groups are shown in red.

Initial investigations focused on the mildest divalent lanthanide reductant Eu(II) to ensure a high functional group tolerance for the reaction and Zn as the sacrificial donor due to its ability to reduce cryptand-encapsulated Eu(III) to Eu(II) when used in large excess.^17^ Irradiation with 365 nm light or with a blue LED (λ_em_ = 463 nm) of a solution of benzyl chloride (**1a**) containing 0.1 equiv of **Eu(III)L** (**L** = **L1**, **L2**, **L3**) yielded bibenzyl **1b** using only 1 equiv of Zn (**Condition A**, Figure 2, Table S1). A range of nonmetallic sacrificial donors were then screened to reduce the amount of metal in the reactions using **1a** as the substrate (Table S1). These studies allowed optimized conditions to be identified for the use of **EuL1** and **EuL2**, as well as conditions for using either metallic (Zn) or organic sacrificial reductants. **Conditions B** (**Eu(III)L1** (0.1 equiv), *N*,*N*-diisopropyl ethylamine (DIPEA, 1 equiv), HCO_2_H (0.5 equiv), 365 nm or blue LED) and **C** (**Eu(III)L2** (0.1 equiv), DIPEA (10 equiv), LiCl (10 equiv), H_2_O (20%), blue LED). **Conditions B** and **C** are tailored to **EuL1** and **EuL2**, respectively, and differ in the excitation wavelength and the amount of sacrificial donor. The shorter excitation wavelength is incompatible with several iodinated and brominated substrates that absorb in this region. Water addition (20%, condition **C**) shifts **EuL2** absorption above 400 nm (ε(**EuL2**, 400 nm) = 3094 M^–1^ cm^–1^ with water vs 714 M^–1^ cm^–1^ without water), which allows for excitation with a blue LED, and thus increases the substrate scope and the selectivity toward bibenzyl formation. A catalyst loading of 10% was suitable for both small- and large-scale reactions and could be lowered to 5 mol % for reactions with >0.1 mmol substrate. **EuL** can be generated in situ from the ligand and an Eu(III) salt without impacting the reaction outcome (Table S4). Control experiments established that light was necessary for catalysis, as was the presence of **EuL** or **SmL**. The replacement of **Eu/SmL** either with the uncomplexed ligand (**L1**, **L2**, or **L3**) or with redox-inactive **Gd(III)L** gave only a trace amount of product.

### Substrate Scope

Under the optimized conditions, either **1b** or **1c** could be obtained selectively from **1a** in excellent yield (97 and 79%, respectively, first substrate in Figure 2a) using **EuL1/L2** or **EuL3**, respectively. For comparison, the corresponding stoichiometric SmI_2_-mediated reaction affords 67% of **1b**.^31^ A range of benzylic halides could be reduced under conditions **A**, **B**, or **C** using **EuL1** and **EuL2** (Figure 2a). Benzyl bromides and chlorides were reduced selectively in the presence of a variety of redox-active functionalities, including ester (**3a**), ketone (**11a**), nitrile (**2a**), aryl bromide (**7a**), ether (**4a**, **11a**, **18a**), thioether (**10a**) and silyl ether (**12a**), and heteroaryl groups (**13a**–**17a**).

Benzyl halide reduction could yield dehalogenated (**1c**–**17c**) or bibenzyl (**1b**–**18b**) products, including biologically active Brittonin A (**18b**). While the reaction outcome was substrate-dependent, in several cases, either product could be selectively obtained by a simple change in the catalyst or reaction conditions. Such ligand-based selectivity is unprecedented for Ln(II)-mediated reductions. The product distribution likely depends on the rate at which a benzyl radical is generated and its stability, as is the case for Ir and Ru catalysts^32,33^ (see entries **1**, **3**, and **11** in Figure 2a). Brittonin A synthesis could be scaled up to yield 807 mg of **18b** in a single batch.

Aryl halides were successfully reduced by using catalytic amounts of lanthanide (Figure 2b). Electron-poor (hetero)aryl bromides and chlorides (**19a**, **22a**–**24a**, **26a**–**27a**) were efficiently dehalogenated, as were electron-rich bromides (e.g., **21a**, **25a**) and iodide (**20a**). The functional group tolerance of C(Ar)-X reductions was remarkable: aldehydes (**21a**) and esters (**22a**, **23a**) were left intact. In contrast, the stoichiometric transformations either did not afford the desired product (**21b**) or gave mixtures of dehalogenated products and byproducts formed by ketone and ester reduction, while requiring 2–8 equiv. of SmI_2_, and either HMPA (>20 equiv) or a proton source.^34−36^ Deuterium labeling experiments (**24c**) reveal the DMF solvent as a proton source under conditions **A** and **C**. However, no deuterium incorporation was seen under condition **B** when conducting reactions either in DMF-*d*_*7*_ or with added formic acid-*d*_*2*_. These results are consistent with DIPEA being the proton source in condition **B** (Table S18).

**Figure 3:**
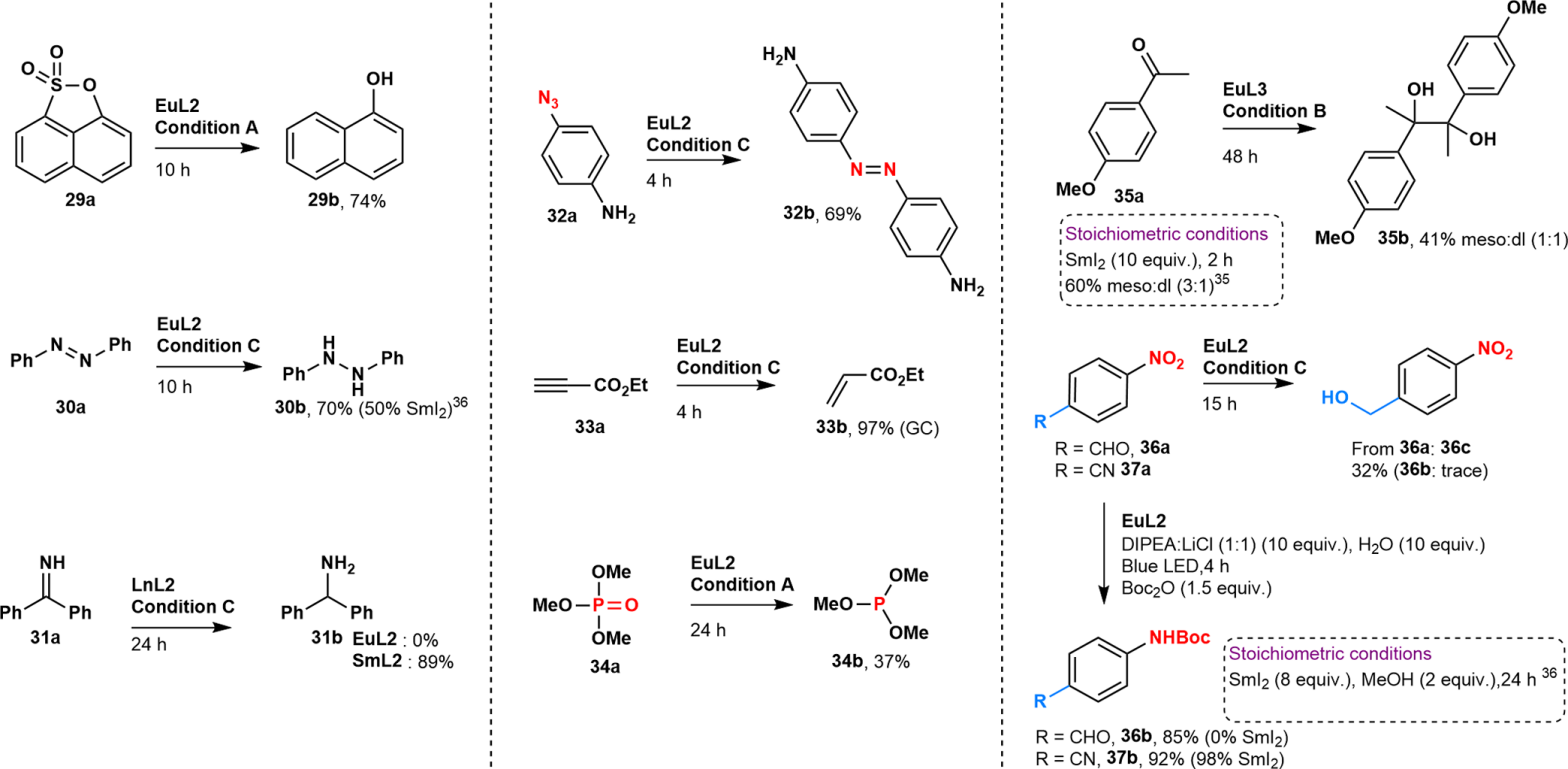
Catalytic C–S, N=N, C=C, and P=O reductions (left, center). C=O reduction followed by pinacol coupling and selective aldehyde and nitro group reductions.

A range of catalytic functional group transformations that have previously been performed using superstoichiometric amounts of Ln(II) were then carried out under photocatalytic conditions (Figure 3). The reductions of **29a**–**37a** proceed without side product formation in yields comparable to or better than that of the stoichiometric process while requiring up to 99% less lanthanide. Diazo compound **30a** was reduced to hydrazine **30b**, while azide in **32a** yielded diazo compound **32b** in 69% yield without over-reduction presumably due to the presence of the electron-donating *p*-amino groups. Pinacol coupling of **35a** proceeded in yields comparable to that obtained previously using 2–10 equiv of SmI_2_.^37,38^ The reaction of **35a** was catalyzed by **EuL3** and not by **EuL1** or **EuL2**. Substrate binding was ligand- and substrate-dependent, as indicated by the differences in the changes of Eu(III) luminescence spectral shapes and luminescence lifetimes (τ_Eu_) upon the addition of various substrates to **EuL1–3** (Figures S55–S67). The open Eu(III) coordination sphere of **EuL3** is likely beneficial for binding a bulky substrate such as **35a**. Additionally, the coordination of the H-donor (XH) to Ln(II) is more likely for **EuL3** than for ligand-encapsulated **EuL1** and **EuL2**. Such XH-coordination has been shown to generate a strong proton-coupled electron transfer reagent, capable of simultaneously transferring an electron and a proton to the substrates that are difficult to reduce.^39^ The reduction of pentavalent phosphorus compounds is an industrially important reaction, often requiring silyl or metal trapping reagents.^40^ Trimethyl phosphate was reduced in moderate yield to phosphite **34b** using **EuL2** as the catalyst and 1 equiv of Zn.

**Figure 4:**
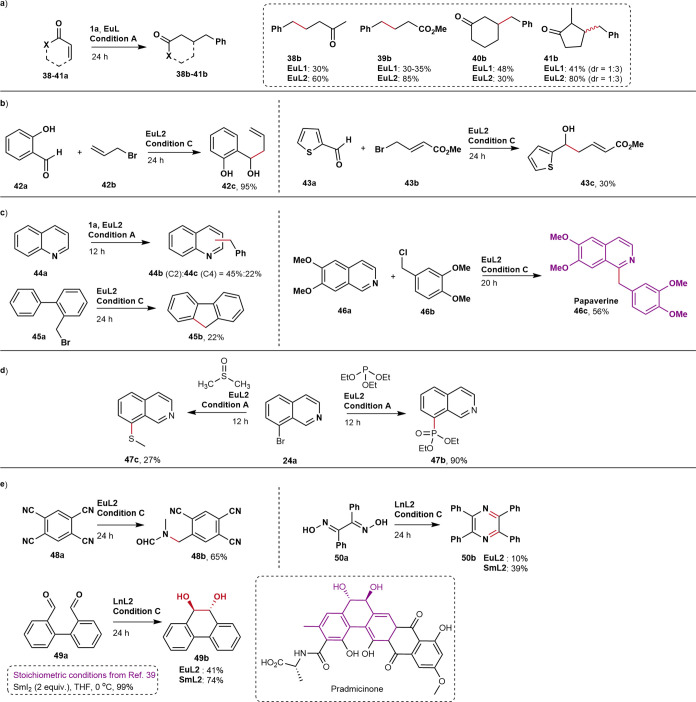
Cross-coupling reactions, C–C, C–P, C–N, and C–S bond formations, and heterocycle and carbocycle syntheses.

The reactivity of the catalytic system is readily tuned. Either **36b** or **36c** could be obtained selectively from **36a** by performing the reaction in the presence of strictly 10 equiv. or a large excess of H_2_O (20% v/v), respectively. Catalyst reactivity was also lanthanide-dependent. Imine (**31a**), aldehyde (**49a**), and oxime (**50a**) were not efficiently reduced with **EuL2**, but the more powerful **SmL2** afforded the products in yields comparable to what was observed using stoichiometric Sm(II) reagents.^41,42^ A similar improvement in the yield was seen upon reduction of **24a** with **SmL2** rather than **EuL2**, presumably also because of the higher reducing power of **SmL2**. Conversely, the more reducible substrates **1a** and **2a** underwent unselective reactions in the presence of **SmL**, while with the milder reductant **EuL** products were obtained with excellent selectivity (Figure 2a). The radical intermediate can be captured by acceptors such as α,β-unsaturated ketones (**38a**–**41a**), aldehydes (**42a**, **43a**), heteroarenes (**44a**, **46a**), and arenes (**47a**) enabling C–C bond formation in good yields (Figure 4a–c). Benzyl addition to α,β-unsaturated ketones (**38a**–**41a**) was possible in a 1,4-fashion to afford **38b**–**41b** in good yields. Allylic bromides **42b** and **43b** reacted with aldehydes **42a** and **43a**, respectively; the 1,2-addition products were obtained in moderate yields. Quinoline (**44a**) alkylation produced a mixture of C2/C4 alkylated isomers (**44b**, **44c**) (Figure 4c), while an analogous intramolecular reaction to a phenyl ring in **45a** gave tricyclic fluorene (**45b**), albeit in low yield. A similar addition of **46b** to isoquinoline **46a** enabled the synthesis of a natural product, papaverine, in a good yield. The aryl radical formed from **24a** could also be harnessed to enable C–S and C–P bond formations (Figure 4d).

Reactions with triethyl phosphite and DMSO gave the corresponding phosphate and methyl sulfide-substituted isoquinolines **47b** and **47c**, respectively. Such synthetic modifications of the isoquinoline pharmacophore^43^ could open up new possibilities for medicinal chemists.

Some products are of particular interest. Pinacol cyclization of **49a** to *trans*-1,2-diol **49b**, a structural motif central to the pradimicin and benanomicin antibiotic classes,^44^ was stereoselective. Preparations of formamide analogue **48b** and tetrasubstituted pyrazines **50b** from simple precursors (DMF and oxime, respectively) could open up new avenues for the synthesis of various drugs incorporating these important pharmacophores.^45,46^

Limitations to the protocol remain. Figure S7 presents a range of aryl halides that were not reduced under conditions **A**, **B**, or **C**. These substrates may require more powerful reductants, e.g., Ln(II) species with more negative reduction potentials.^47^ Attempted cross-reactions between benzyl chloride and aryl, alkynyl, or carbonyl acceptors and unsuccessful pinacol coupling reactions are shown in Tables S48–S51 and Figure S7, respectively. The success of these cross-couplings likely requires an adjustment of the ligand.

### Mechanistic Studies

**Figure 5:**
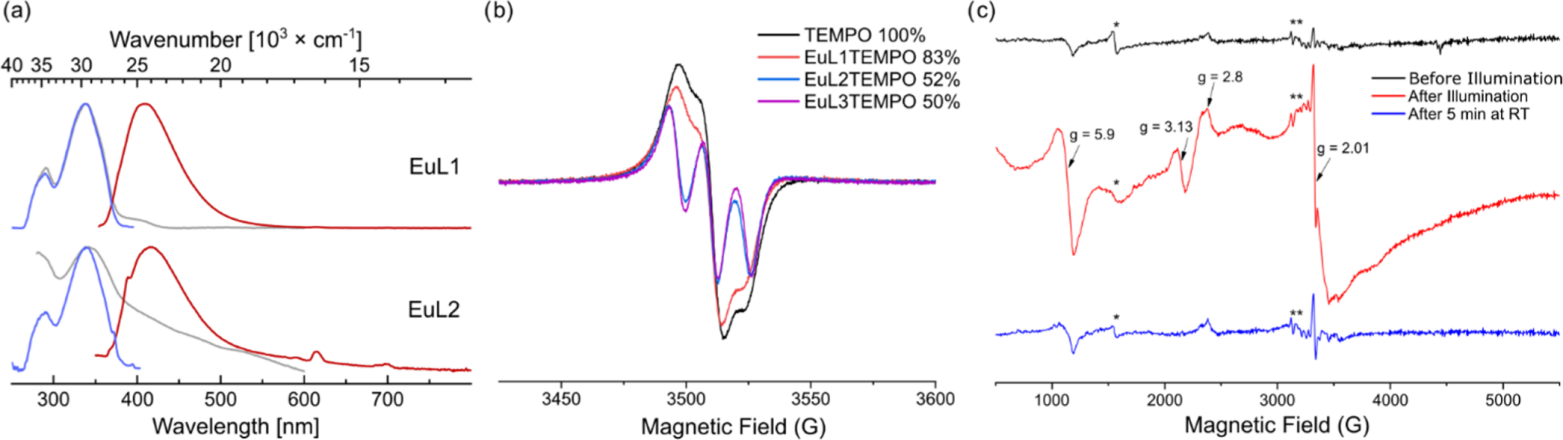
(a) Normalized absorption (gray), excitation (blue), and steady-state emission (red) spectra for **EuL1** and **EuL2** in DMF (λ_exc_ = 339 and 348 nm, respectively). (b) Room-temperature EPR spectra of TEMPO and **EuL1**/**EuL2**/**EuL3** containing TEMPO (1:1) after 12 h irradiation with a blue LED, microwave power 2 μW, and modulation amplitude 1 G. The integrated area of the radical signal (normalized to the TEMPO-only sample) is shown in the figure. (c) EPR spectra of **EuL2** (1 mM in DMF) before illumination (black), directly after illumination (red), and after 5 min at room temperature (blue). EPR parameters: microwave power 2 mW, modulation amplitude 19.4 G, temperature: 10 K. The signal marked with * in the EPR spectra is a contamination from Fe(III), and the signals marked with ** are a small amount of Mn(II). The cavity signal has been subtracted from the spectra, and a baseline correction has been applied.

**LnL** absorb at the excitation wavelengths used in the reactions; absorption is through the chromophore (**Chr**, Figure 5a). To investigate the fate of the excitation energy, the fluorescence quantum yields (Φ_L_) and lifetimes (τ_L_) of the chromophores (Chart 1) in **GdL1** and **GdL2** were compared to those of **EuL1**/**SmL1** and **EuL2**/**SmL2**, respectively. Gd(III) has a similar ionic radius and heavy atom effect to Eu(III) and Sm(III) but is not photoactive.^25^ The reduction potential required to access Gd(II) is much more negative than that required for accessing Eu(II).^29,48^ Thus, **Gd(III)L** provides a model for **Eu(III)L** that recreates the coordination and electronic properties of the complexes but does not allow for Ln(II) formation. The Gd complexes had higher Φ_L_ and longer τ_L_, which is consistent with the presence of additional processes (energy or electron transfer) quenching the first singlet excited state of **Chr** in **EuL** and **SmL**. Electron transfer from **Chr** to Ln(III) (Ln = Eu, Sm) was calculated to be thermodynamically feasible (Tables S55 and S56).

There are several conceivable mechanistic pathways for the catalytic processes to follow. Ln(III) reduction by the photoexcited chromophore yields Ln(II),^49,50^ which may be intercepted by a substrate either before or after **Chr** regeneration by the sacrificial reductant (Figure 1). Control experiments established that it is the photoexcitation of **Eu/SmL** that yields the active reagent (Table S2). Neither the uncomplexed ligand (**L1**, **L2**, or **L3**) nor **Gd(III)L** gave trace amounts of product. These experiments show that Ln(II) formed from the reducible Ln(III) rather than the excited chromophore reduces the substrate. An Eu(III) complex of the ligand lacking the sensitizing antenna (**L2m**, Chart 1) did not promote the reaction; therefore, the Eu(III) is reduced by the excited antenna and not directly by the sacrificial reductant. Irradiation of a mixture of 1 equiv of either **EuL1** or **EuL2** and **1a** in the absence of a sacrificial reductant gave **1b** in 78% and 42% GC yield, respectively, while the same reaction with **GdL1** or **GdL2** did not afford any product. Thus, Eu(III) can be reduced by the photoexcited **Chr** and in turn can reduce the substrate; the role of the sacrificial reductant is to regenerate **Chr** and enable the use of catalytic amounts of **EuL**. Eu(II) is a more powerful reductant in its excited state than in its ground state.^51^**Eu(II)L1** was inactive in the absence of light (Scheme S2). Thus, either the photoinduced electron transfer (PeT) yields Eu(II) in its excited state or ground-state Eu(II) is initially formed and gets excited.

In the presence of the radical quencher 2,2,6,6-tetramethylpiperidinyloxy (TEMPO), the reaction does not proceed (table S6). LCMS analysis of an irradiated sample containing **EuL1** and TEMPO showed the protonated molecule ion of the **L1**-TEMPO adduct (*m*/*z* = 662, Figure S118). To gather further evidence of either **Chr^•+^** or Eu^2+^ formation, two EPR experiments were carried out. First, solutions of **EuL**/**GdL** containing equimolar amounts of the radical quenchers TEMPO or PBN (*N*-tert-butyl-α-phenylnitrone) were irradiated. TEMPO, but not PBN, is an EPR-active free radical. PBN can instead form stable radical species after reaction with an organic radical. EPR analysis of the irradiated TEMPO-containing solutions indicated 20–50% reduction of the TEMPO signal in the presence of **EuL** (Figure 5b) but no quenching in the presence of **GdL** (Figure S69). The irradiation of a mixture of **EuL2** and PBN showed the emergence of an EPR signal corresponding to a N-based radical from a PBN adduct (Figure S70).^52^ EPR analysis at cryo temperatures (10 K) of an irradiated (30 min, blue LED) solution of **EuL2** showed features at *g* = 5.9, 3.13, 2.8 and a broad feature at *g* = 2, in addition to a sharp radical-like signal visible at *g* = 2.01 (Figure 5c, red). All of these features disappeared after the sample was thawed, kept at room temperature for 5 min, and frozen again (Figure 5c, blue). The sharp signal at *g* = 2.01 is an organic radical. The other features collectively indicated the formation of Eu^2+^ species,^53,54^ the identity of which was further supported by comparison to the EPR signals of **Eu(II)L1** and isoelectronic **GdL2** (Figure S73). These results are consistent with the formation of a radical cation and Eu^2+^ in **EuL** under illumination by electron transfer from **Chr**. To the best of our knowledge, the Eu(II) EPR spectrum is the first spectroscopic evidence of such photochemical Ln(II) formation.

## Conclusions

Strongly reducing Ln(II) species were accessed from Ln(III) precursors through photochemical reduction by an excited-state organic chromophore and used as catalysts. Excitation could be performed with both UV and visible light. The Ln(II) could reduce a broad range of substrates with excellent functional group tolerance. The catalytic cycle was closed by reduction of the oxidized chromophore with Zn or nonmetallic sacrificial reductants. The selectivity of the reaction and the reducing power of the catalyst could be independently tuned by the ligand, the lanthanide, and additives (H_2_O). Ln(II) catalysis has been demonstrated on a broad substrate scope with the yield and selectivity comparable to or better than the corresponding stoichiometric processes on a synthetically useful scale. The scope of the Ln(II) catalysis includes synthetically important C–C, C–N, C–P, C–S, and N–N bond formation reactions, C–halogen, P–O, C–C, C–S, and N–N bond cleavage reactions, and the synthesis of biologically important carbocyclic and heterocyclic structural motifs. Eu(II) formation via the reduction of Eu(III) by a photoexcited nearby chromophore was supported by EPR spectroscopic findings. These photocatalytic reactions are substantially more benign than their stoichiometric analogues, as they can be performed without toxic HMPA, and with up to 99% less lanthanide.

## Supporting Information Available

Synthesis and characterization of new compounds; optimization and control experiments, additional evaluated substrate scope and mechanistic studies (PDF)
