# Supplementary material for: Photocatalytic Generation of Divalent Lanthanide Reducing Agents
Source: J Am Chem Soc. 2023 Oct 5;145(41):22555–62. doi: 10.1021/jacs.3c07508 (PMC10591332; doi:10.1021/jacs.3c07508)
Supplement: Supplementary file 1 — ja3c07508_si_001.pdf [file ja3c07508_si_001.pdf]

Photocatalytic generation of divalent lanthanide reducing agents

Monika Tomar[1]†, Rohan Bhimpuria[1]†, Daniel Kocsi[1], Anders Thapper[1], K. Eszter Borbas[1]*

[1]Department of Chemistry, Ångström Laboratory, Uppsala University; Uppsala, 75120, Sweden.

*eszter.borbas@kemi.uu.se

† These authors contributed equally to this work.

Contents

Materials and methods 1
General information on Ln(II) catalysis 5
Optimization studies for reductive dehalogenation 6
Ligand and LnL syntheses 14
Synthesis of starting materials 21
    Synthetic procedures and product characterization of Ln(II)-catalyzed reductions 23
Limitations of the catalytic procedure 48
Photophysical characterization 50
Electrochemical characterization 74
Calculation of excited state potential 78
Calculation of driving force for photoinduced electron transfer 79
Substrate binding study 80
EPR study 87
GCMS traces 92
^{1}H, ^{13}C, ^{19}F, and ^{31}P NMR spectra 115
References 163

Materials and methods

Materials. Compounds **S1**,[1] **L3**,[2] and **16a**,[3] were synthesized following literature methods. All other chemicals were from commercial sources and used as received. DMF was obtained from an Inert Puresolv solvent purification system and was dried over molecular sieves and degassed using three freeze-pump-thaw cycles. Zn dust was dried under vacuum for overnight before use.

General Procedures. ^{1}H NMR (400 MHz), ^{13}C NMR (100 MHz), ^{31}P NMR (160 MHz), ^{2}H NMR (60 MHz), and ^{19}F NMR (376 MHz) spectra were recorded on a JEOL 400 MHz instrument. Chemical shifts were referenced to residual solvent peaks and are given as follows: chemical shift (δ, ppm), multiplicity (s, singlet; br, broad; d, doublet, t, triplet; q, quartet; m, multiplet), coupling constant (Hz), integration. Accurate mass analyses were performed at the Organisch Chemisches Institut WWU Münster, Germany. All compounds displayed the expected isotope distribution pattern.

Paramagnetic ^{1}H NMR. ^{1}H NMR spectra of Eu-complexes were recorded at ambient temperature (21 °C) at 400 MHz (1–16 mM) using the following parameters: relaxation delay: 1 s; number of scans: 32; number of points: 32768–131072; range: −30 to +50 ppm. The chemical shifts were referenced to D_2O (4.77 ppm) for **EuL1, EuL2** and to DMSO-d_6 (2.50 ppm) for **EuL3**. Phase correction and exponential decay as apodization function 3 and 10 Hz have been applied for ^{1}H spectra.

Chromatography. Preparative chromatography was carried out on silica gel [Normasil 60 chromatographic silica media (40–63 micron)] stationary phase. Thin layer chromatography was performed on silica-coated (60G F254) aluminium plates from Merck and aluminium oxide coated with 254 nm fluorescent indicator aluminium plates from Sigma-Aldrich. Samples were visualized by UV-light (254 and 365 nm) and permanganate stain.

HPLC-analysis was performed using the Agilent 1290 Infinity II HPLC system equipped with a 1290 Infinity II High Speed pump and a 1260 II Infinity DAD HS UV-vis detector, using an InfinityLab POROSHELL 120 EC-C18 column with dimensions of 50 mm×2.1 mm and 1.9 µm particle size was performed. The HPLC is coupled to an InfinityLab LC/MSD G6125B detector equipped with an ESI source as ionization. LC separation was performed with water (A, 0.05% formic acid):acetonitrile (B, 0.05% formic acid) eluent system using the methods **LC1** and **LC2**. **LC1**: 1→10 min: 10% B; 1→10 min: 10%→90% B and 10–11 min: 90% B,

11–12 min: 10% B, Flow rate: 0.8 mL/min. **LC2**: 1→10 min: 10% B; 1→10 min: 10%→90% B and 10–11 min: 90% B, 11–12 min: 10% B, 0.3 mL/min.

Spectroscopy. All measurements were performed in DMF (taken from the solvent purification system) unless indicated otherwise. Sample absorptions were A = 0.07–0.1. Quartz cells with 1 cm optical pathlengths were used for the room temperature measurements. The absorption spectra were recorded on a Varian Cary 100 Bio UV-Visible spectrophotometer. The emission and excitation spectra, lanthanide luminescence lifetimes, time-resolved spectra, and luminescence quantum yields were recorded on a Horiba FluoroMax-4P instrument. All emissions were corrected by the wavelength sensitivity (correction function) of the spectrometer. All measurements were performed at room temperature unless stated otherwise.

Quantum yields were determined at room temperature using quinine sulfate (QS) in H_2SO_4 0.05 M (Φ_{ref} = 0.59)[4] as reference in Equation S1. Quantum yields were calculated according to Eq. S1, with Φ_s the quantum yield of the sample, Φ_{ref} the quantum yield of the reference, I the integrated corrected emission intensity of the sample (s) and of the reference (ref), f_A the absorption factor of the sample (s) and of the reference (ref) at the excitation wavelength and n the refractive indexes of the sample (s) and of the reference (ref). The concentrations of the complexes were adjusted to obtain an absorbance around the maxima of the antennae matching that of the QS fluorescence standard. The excitation wavelength where the absorption factors of the samples and of the reference were the same was chosen (i.e. where the absorptions are identical). The corrected emission spectra of the sample and reference standard were then measured under the same conditions over the (350–800 nm for **LnL1** and 364–800 nm for **LnL2**) spectral range as well as blank samples containing only the solvent. The appropriate blanks were subtracted from their respective spectra and the antenna fluorescence was separated by fitting the section of the antenna emission exponentially overlapping the lanthanide emission. The quantum yields were calculated according to Equation S1. The given relative error on the quantum yields ($\delta\Phi = \Delta\Phi/\Phi$, where $\Delta\Phi$ is the absolute error) take into account the accuracy of the spectrometer and of the integration procedure [$\delta(I_s/I_{ref}) < 2\%$], an error of 0.59 ±0.01 on the quantum yield of the reference QS [$\delta(\Phi_{ref}) < 2\%$], an error on the ratio of the absorption factors [$\delta(f_{Aref}/f_{As}) < 5\%$, relative to the fixed absorption factor of the reference QS] and an error on the ratio of the squared refractive indexes [$\delta(n_s^2/n_{ref}^2) < 1\%$, < 0.25% around 1.333 for H_2O and 1.430 for DMF on each individual refractive index], which sums to a total estimated relative error that should be $\delta\Phi_s < 10\%$. A limit value of 10% is thus chosen.

$$\Phi_s = \frac{I_s}{I_{ref}} \cdot \frac{f_{Aref}}{f_{As}} \cdot \frac{(n_s)^2}{(n_{ref})^2} \cdot \Phi_{ref} \quad \text{(S1)}$$

Low temperature measurements were done in quartz capillaries (0.2 cm optical pathlength) at 77 K by immersion in a liquid N_2-filled quartz Dewar and with addition of glycerol (1 drop) to the solutions (9 drops) measured at room temperature. Glycerol used for low temperature experiments was of >99.9% purity.

Lifetimes were recorded 0.05 ms after pulsed excitation at the excitation maxima (λ_{ex}) of the ligand by measuring the decay of the lanthanide main emission peak (Eu(III) 616 nm) The increments after the initial delay were adjusted between 0.2–20 μs depending on the lifetime in order to have a good sampling of the decay. The obtained data were fitted by single and double exponential decay models in OriginPro 9, and the most reliable value was chosen according to the adjusted R^2 value and the shape of the residuals. A relative error of 10% is typically found among a series of measurements on the same sample.

The fluorescence lifetime decays in the nanosecond range were measured on Spectrofluorometer FS5 system from Edinburgh Instruments. The system was equipped with picosecond pulsed light emitting diode EPLED-340 with excitation wavelength at 341.5 nm. The data were acquired in the 50 ns measurement range with peak preset at 10^4 counts in 1024 channels. The repetition rate of the excitation source was 10 MHz, and the synchronization delay was 80 s. The scatter light profile (prompt signal, black in the decay figures) was recorded for each experiment individually in the same quartz cuvette using diluted Ludox solution in HPLC water at 341.5 nm emission wavelength with similar parameters as were used for the measured sample (red in the decay figures). The obtained data were fitted in the Fluoracle software (green trace in the decay figures) using mono and biexponential reconvolution fit model in Equations S2 and S3, where τ_1 is the sample lifetime, t is time represented in ns, B_1 is the population (100% in all cases) (Table S45).

$$R(t) = B_1 * \exp(-t/\tau_1) \quad \text{(S2)}$$

$$R(t) = A + B_1 * \exp\left(-\frac{t}{\tau_1}\right) + B_2 * \exp\left(-\frac{t}{\tau_2}\right) \quad \text{(S3)}$$

Photostability experiments were performed on Horiba FluoroMax-4P at room temperature with A = 0.10 in DMF unless otherwise noted. Each sample was prepared in a 1 cm screw-capped quartz cuvette (3 mL) and was continuously irradiated for 2 hours with emission spectrum recorded every 15 minutes. The front slit was 3 nm, the exit slit was 3 nm. Each sample evolution upon light irradiation was tested via absorbance spectroscopy before and after being

irradiated. No major differences were noticed in the samples. The signal of the solvent recorded in the same conditions was subtracted from the recorded emission spectra (Figures S25, S27, S29) which were then integrated. The resulting integrated intensity values were divided by that at t_0 and multiplied by 100% (Figures S26, S28, S30).

Fourier Transform Infrared Spectroscopy (FTIR). The measurement was done on Perkin Elmer Spectrum One instrument. IR spectra was recorded on a dry sample by making a pellet using KBr with the ligand or complex (100:1). Blank was recorded with only KBr pellet. The main infrared bands of the ligand and their complexes are shown (Figures S38, S39, S40, S41, S42, S43, S44 and S45) and reported (Table S46).

Electrochemistry. Cyclic voltammograms (CV) were obtained at room temperature (~20 °C) using an AUTOLAB PGSTAT 100 potentiostat, or an AUTOLAB PGSTAT 204N potentiostat. The experiments were performed in an Argon atmosphere glovebox with level of O_2 (<0.5 ppm) and H_2O (<0.5 ppm). The setup was equipped with a 3 mm glassy carbon (GC) working electrode, a Pt wire auxiliary electrode, and an Ag wire as a reference electrode with ferrocene as an internal standard. Here, voltammograms are shown and values are reported for Fc/Fc^+ (Table S48). Measurements were done in anhydrous DMF with NBu_4PF_6 (0.1 M) as the supporting electrolyte. The solvent was degassed prior to bringing it into the glovebox by three freeze-pump-thaw cycles. The electrolyte solution was prepared in the glovebox. The voltammograms were recorded by scanning first toward more negative potential values (reduction). A step-potential of –0.9 mV was used for 100 mV/s scan rates.

A solution of NBu_4PF_6 (0.1 M) in DMF (2 mL) was added to the electrochemical cell. The working electrode was polished with 0.05 µm alumina on a polishing pad, washed with water and ethanol, and dried with air before bringing into the glovebox. This was repeated before each new sample. The three electrodes (GC working electrode, platinum wire auxiliary electrode, and silver wire reference electrode) were inserted into the cell setup and a background scan was recorded with a scan rate of 100 mV/s, and two sweeps. The complexes were added to the solution (2 mM). At the end of the experiment, ferrocene was added to the solution, and cyclic voltammograms were recorded again with scan rates 100 mV/s with one sweep.

EPR Spectroscopy. EPR measurements at room temperature were performed using a Bruker EMX Micro spectrometer, equipped with an ER 4119HS resonator. EPR samples were prepared in a 1 mm capillary. EPR parameters: Microwave frequency, 9.86 GHz; modulation frequency, 100 kHz. EPR measurements at 10 K were performed using a Bruker ESR-500 spectrometer, equipped with an ER 4122SHQ resonator, an ESR900 cryostat, and an Oxford

ITC503 temperature controller. EPR parameters: Microwave frequency, 9.41 GHz; modulation frequency, 100 kHz.

General information on Ln(II) catalysis

Reaction setup

Photoreactors. (1) RPR-100 and 200 Rayonet Photochemical Chamber Reactors were used for photoreactions (Figure S1a). A typical setup has 16 x 24W UV lamp of 365 nm (Southern New England Ultraviolet Company). (2) 40 W blue LED lamp (Kessil A160WE Tuna Blue, λ_{max} = 463 nm, set highest blue color and intensity) was also used for irradiation (Figure S1b and S1c). Reactions were stirred at 600−1000 rpm. For the full emission spectrum of the A160WE Tuna Blue light source see Reference [5].

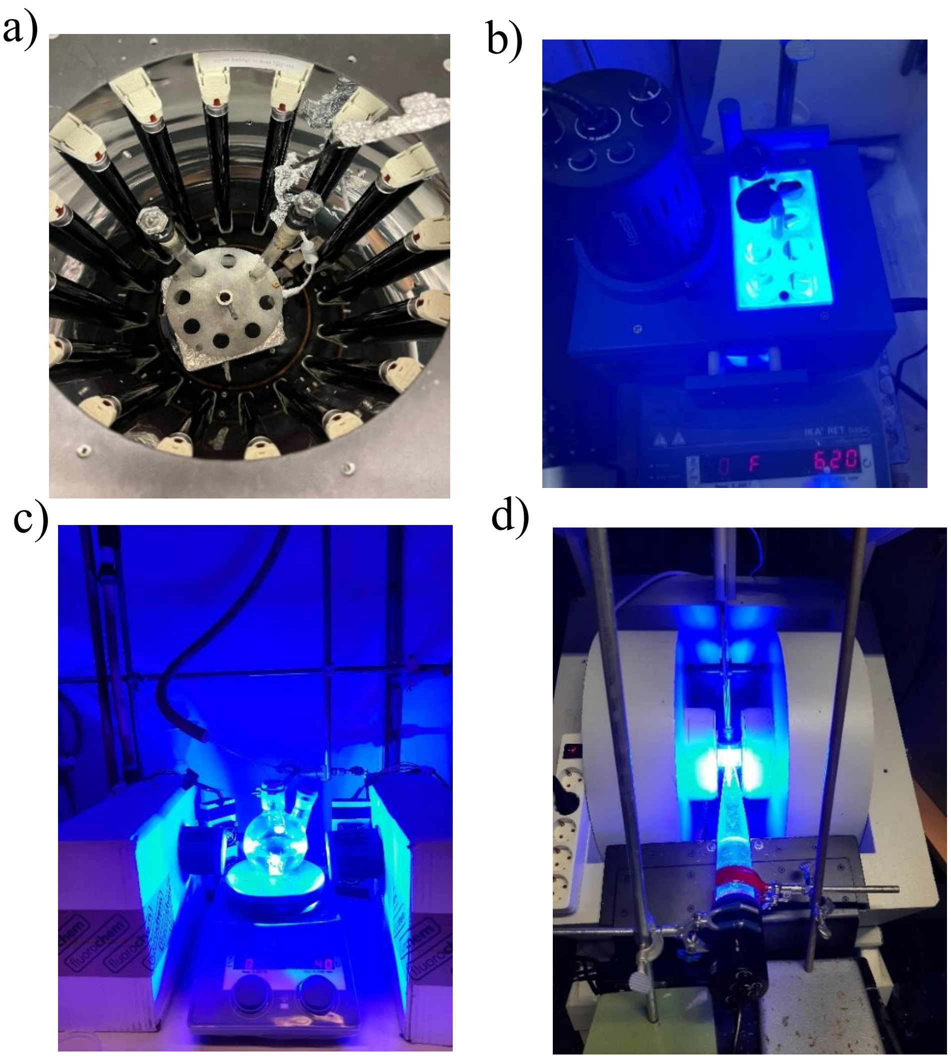

Figure S1. a) Reaction setup in Rayonet photoreactor, b) Kessil lamp setup for small scale reaction, and c) Kessil lamp setup for scale-up reaction, d) Irradiation set up in EPR instrument.

General reaction setup. All reactions were performed in Microwave vials (5–30 mL) or quartz tubes (20 mL) equipped with a stirring bar, in a dry glovebox [O_2 (<0.5 ppm), H_2O (<0.5 ppm)] with an Ar atmosphere. The quartz tubes and the vials were sealed with an electric black tape.

Optimization studies for reductive dehalogenation

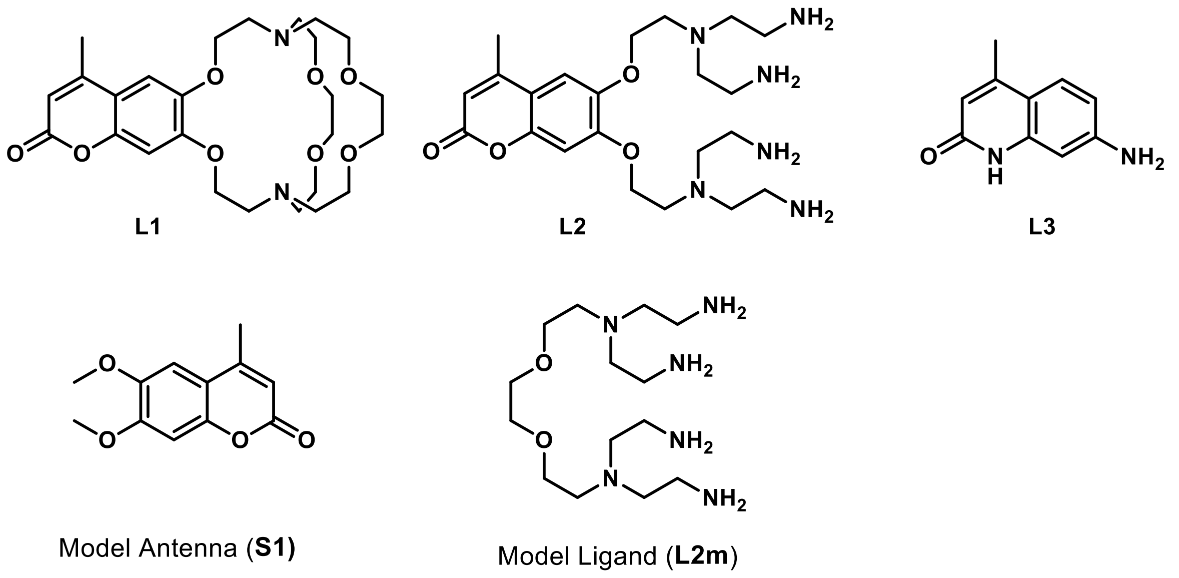

Figure S2. Structures of ligands.

Table S1. Optimization study of benzyl halide reduction.[a]

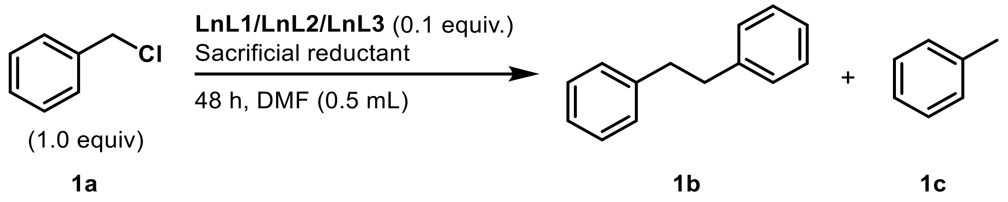

Entry	LnL	Sacrificial reductant (equiv.)	Light Source	Yield (%)[b]	
				**1b**	**1c**
1	**EuL1**	Zn (1)	365 nm	60	-
2	**EuL2**	Zn (1)	Blue LED	92	-
3	**EuL3**	Zn (1)	Blue LED	-	79
4	**EuL (L= L1, L2, L3)**	DIPEA (1)	Blue LED/365 nm	-	-
5 [c]	**EuL1**	DIPEA:HCO_2H (1:1)	365 nm	18	-
6	**EuL1**	DIPEA:HCO_2H (1:0.5)	365 nm	46	-
7 [c]	**EuL1**	DIPEA:HCO_2H (1:2)	365 nm	10	

8	**EuL1**	DIPEA:Hantzsch ester (1:1)	365 nm	trace	-
9	**EuL1**	Et_3N/Ethanolamine/*n*-Bu_4NCl	365 nm	-	-
10	**EuL2**	DIPEA:HCO_2H (1:1)	365 nm	-	-
11	**EuL2**	DIPEA:$Zn(OAc)_2$ (1:1)	Blue LED	70	-
12	**EuL2**	DIPEA:LiCl (1:1)	365 nm	trace	-
13	**EuL2**	DIPEA:LiCl:H_2O (1:1:1)	Blue LED	70	30
14	**EuL2**	DIPEA:LiCl (1:1), H_2O (20%)	Blue LED	97	-
15	**EuL2**	Zn, H_2O (10%)	Blue LED	-	91
16	**EuL3**	DIPEA:HCO_2H (1:1)	365 nm	-	70
17	**SmL1**	Zn	365 nm	53	-
18	**SmL2**	Zn	Blue LED	10	30
20	**DyL1**	Zn	Blue LED	44	-
21	**DyL2**	Zn	Blue LED	31	56

[a] **1a** (0.015–0.03 mmol), sacrificial reductant (1–10 equiv), **LnL1, LnL2 or LnL3** (0.0015–0.003 mmol), DMF (0.5 mL), 48 h, light source.

[b] GCMS yield determined using a calibration curve prepared from integrated peak areas of **1a** (1.1–28 μM), **1b** (0.3–8 μM), and **1c** (0.8–16 μM) solutions.

[c] for entries 5 and 7 the formation of benzyl formate side product in varying amounts was seen. The amount of side-product formed is dependent on the number of equivalents of formic acid used (see **Figure S5**).

Summary of optimization studies

Based on the results of optimization studies, three different conditions using different sacrificial reductants were selected for the exploration of the substrate scope.

Condition A. Eu(III)L (**L** = **L1**, **L2**, or **L3**, 0.1 equiv.), Zn (1.0 equiv.), Blue LED, 24 h, DMF.

Condition B. Eu(III)L1/**Eu(III)L3** (0.1 equiv.), DIPEA (1.0 equiv.), HCO_2H (0.5 equiv.), 365 nm, 48 h, DMF.

Condition C. Eu(III)L2 (0.1 equiv.), DIPEA (10.0 equiv.), LiCl (10.0 equiv.), H_2O (20%), Blue LED, 24 h, DMF.

Table S2. Control experiments for benzyl halide reduction.

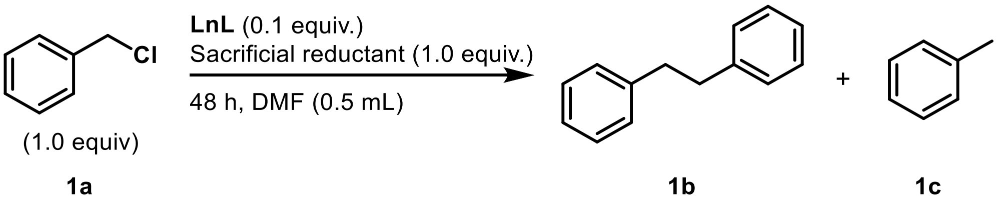

Entry	LnL	Condition	Light source	Yield (%)	
				**1b**	**1c**
1	**GdL1/L3**	**B**	365 nm	< 4	-
2	**GdL1/GdL2/GdL3**	**A**	Blue LED	NR[a]	NR
3	**GdL2**	**C**	Blue LED	NR	NR
4	**EuL1**	**B**	DARK	NR	NR
5	**EuL1/EuL2/EuL3**	**A**	DARK	NR	NR

[a] NR: no reaction, i.e. no consumption of starting material.

Table S3. Control experiments with Eu(III) complex lacking the sensitizing antenna.

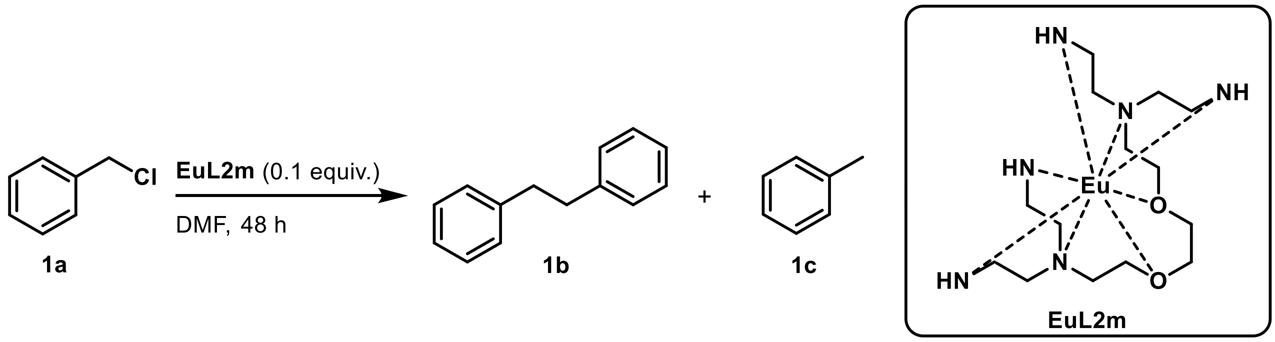

Entry	Condition	Light source	Yield (%)	
			**1b**	**1c**
1	**A**	Blue LED	NR[a]	NR
2	**C**	Blue LED	NR	NR

[a] NR: no reaction, i.e. no consumption of starting material.

Representative GCMS chromatograms of the reactions of 1a, 1b and 1c:

Method A.

Photoreactions were monitored by GC-MS (Agilent 7890A GC and 5975 MSD system). Samples were injected using split injection (1 µL injection volume; split ratio: 100:1; 250 °C inlet temperature; flow rate: 120 mL/min). The temperature rate was set to 50 °C/min resulting in a 12.5 min total run time. He was used as a carrier gas at a flow rate of 1.2 mL/min. The

column used was an Agilent 19091S-433: 325 °C: 30 m x 250 µm x 0.25 µm (front SS-inlet: He; out: vacuum). Mass spectrometer: Source temperature: 230 °C, Quad-temperature 150 °C.

Method B.

Samples were injected using split injection (1µL injection volume; split ratio: 100:1; 250 °C inlet temperature; flow rate: 120 mL/min). The temperature rate was set to 20 °C/min resulting in a 12.5 min total run time. He was used as a carrier gas at a flow rate of 1.2 mL/min. The column used was an Agilent 19091S-433: 325 °C: 30 m x 250 µm x 0.25 µm (front SS-inlet: He; out: vacuum). Mass spectrometer: Source temperature: 230 °C, Quad-temperature 150 °C.

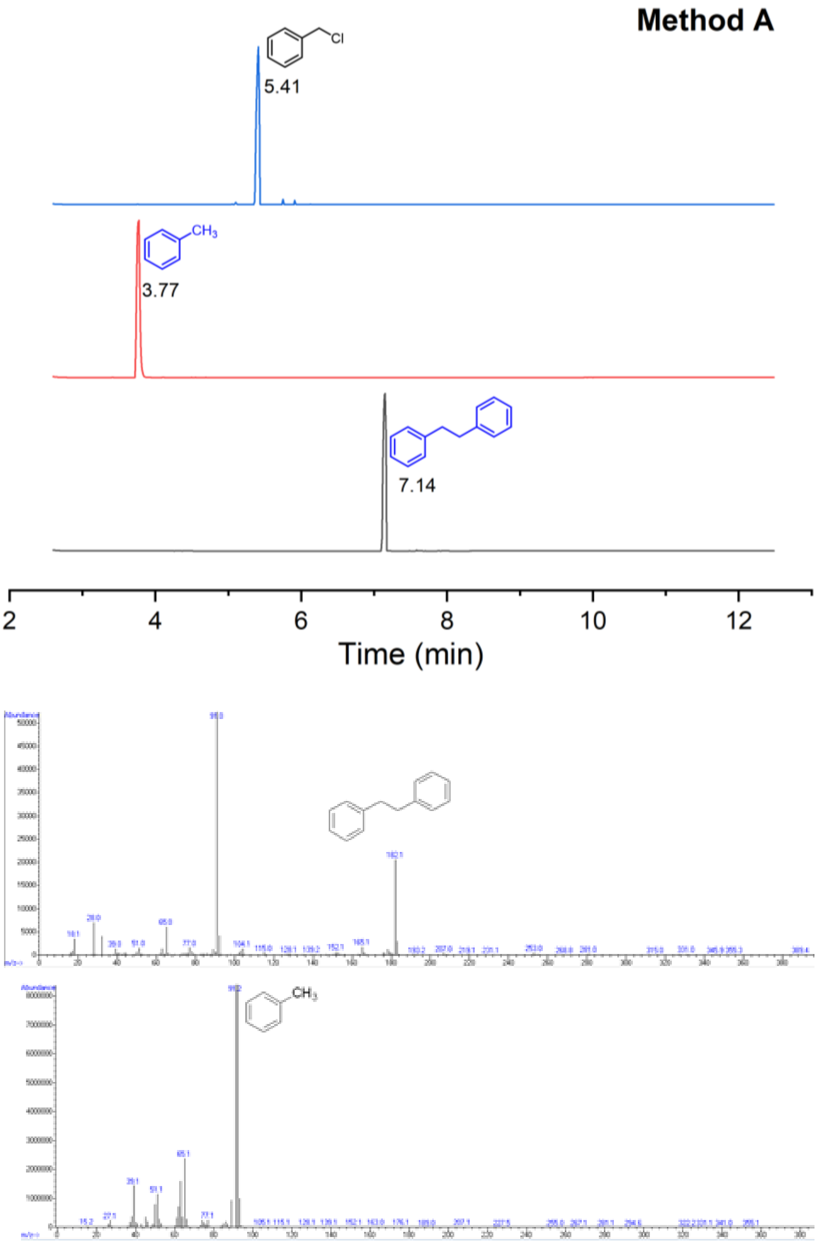

Figure S3. Representative GC traces of standards of **1a**, **1b**, and **1c** in **Method A**.

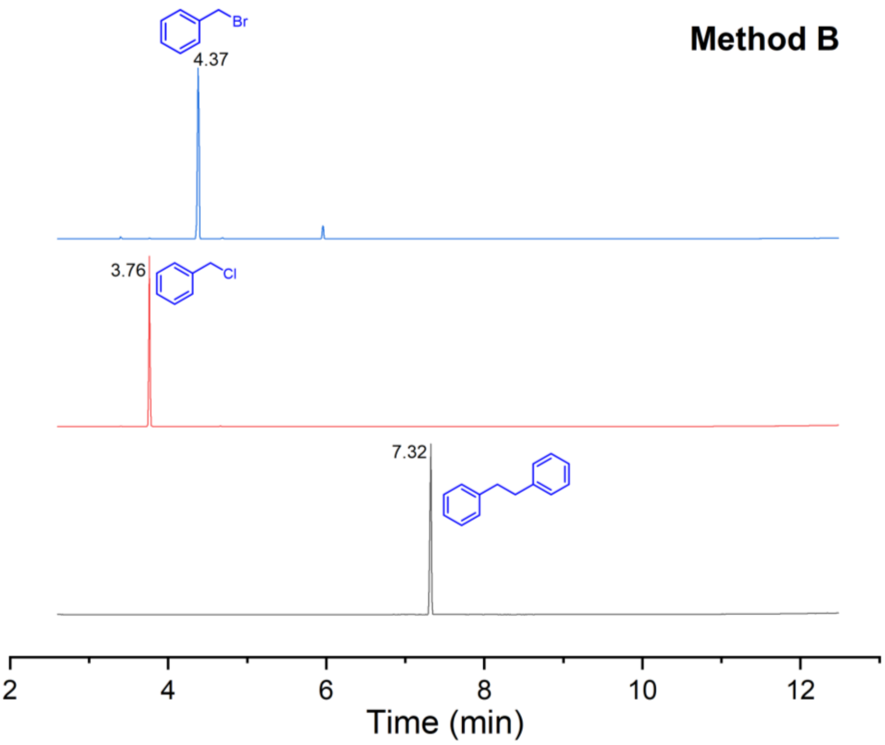

Figure S4. Representative GC traces of standards of **1a** and **1c** in **Method B**.

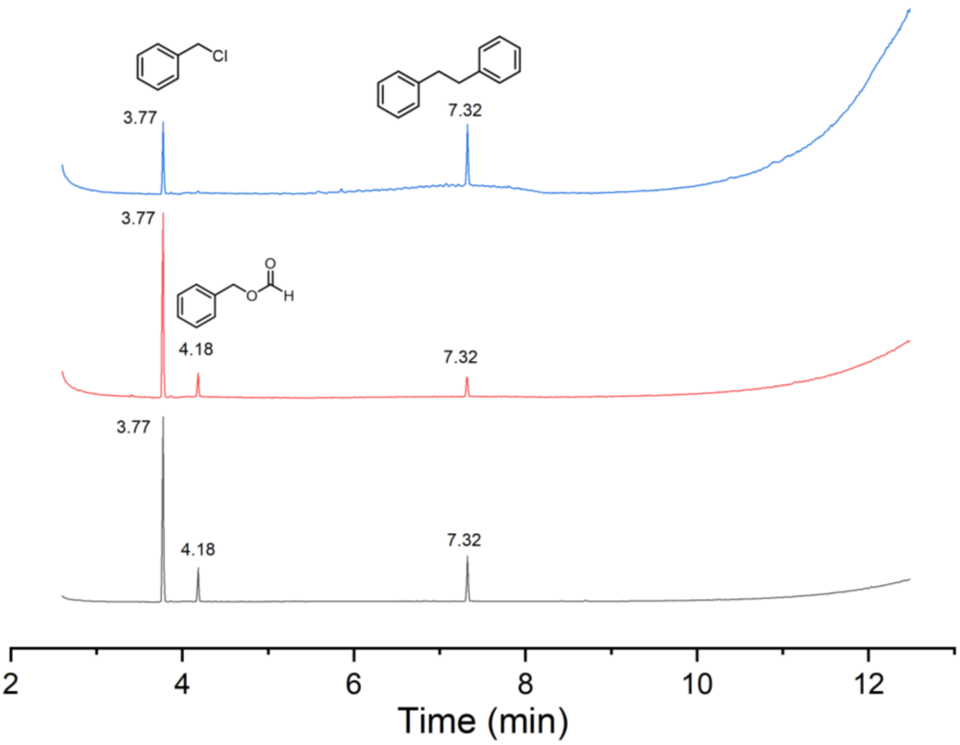

Figure S5. Overlapping GC chromatograms of entries 6 (blue), 7 (red), and 5 (black) (Table S1) in **Method B** showing the formation of benzyl formate side product when using 0.5, 1, 2 equiv. of formic acid in the reaction.

Note: This side product formation is responsible for the low yield of the reactions using **4a**, **6a**, and **7a** when reacting under **Condition B**.

Stoichiometric benzyl halide reduction reaction

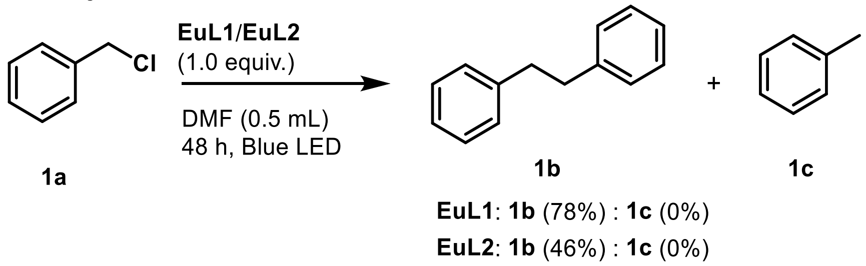

Scheme S1. Stoichiometric benzyl chloride reduction using **EuL1**, **EuL2**.

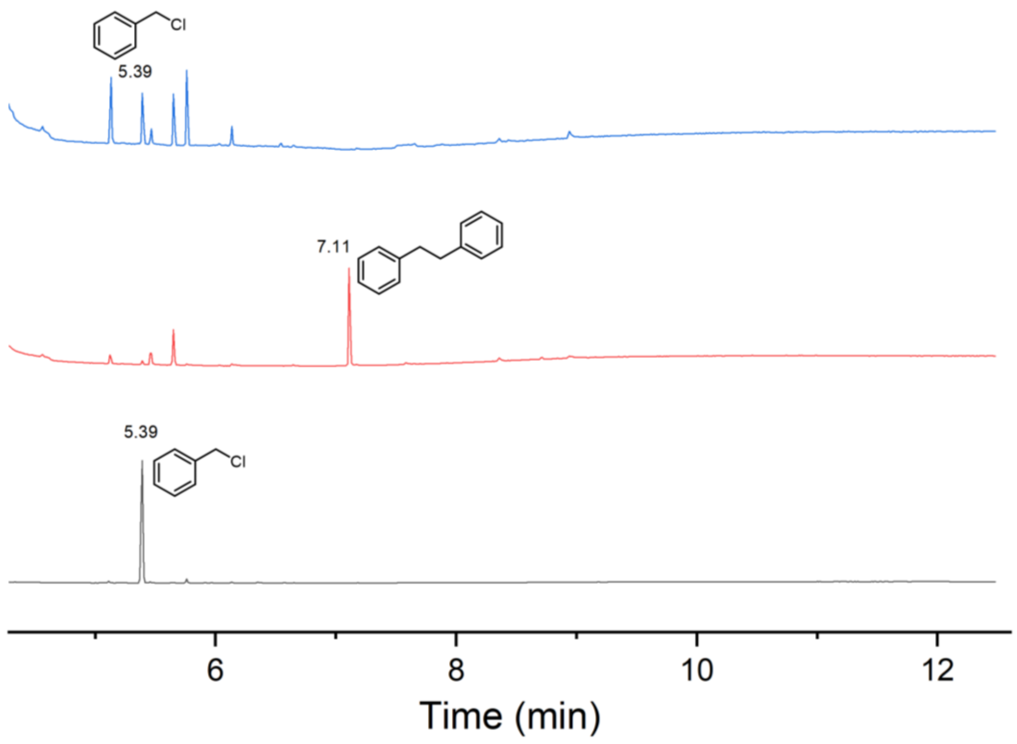

Figure S6. Representative GC chromatograms (**Method A**) of benzyl chloride reduction using 2.0 (blue), 1.0 (red), and 0.5 equiv. (black) of **EuL1**. Note: Region of 2.5–4.0 min is not shown due to the strong DMF signal.

Benzyl chloride reduction with Eu(III) salt and L1–L3.

Table S4.

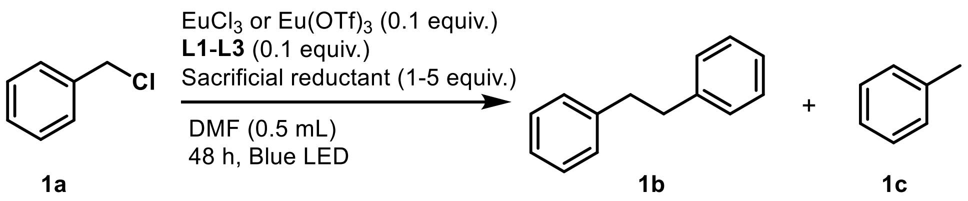

Entry	EuX_3	Ligand	Conditions	GC-Yield (%)	
				**1b**	**1c**
**1**	$Eu(OTf)_3$	**L1**	**B**	31	9
**2**	$EuCl_3$	**L2**	**A**	71	0
**3**	$EuCl_3$	**L2**	**C**	56	13
**4**	$EuCl_3$	**L3**	**A**	0	93
**5**	$Gd(OTf)_3$	**L1**	**B**	6	0
**6**	$GdCl_3$	**L2**	**A**	0	0
**7**	$GdCl_3$	**L2**	**C**	0	0
**8**	$GdCl_3$	**L3**	**A**	0	0

Benzyl chloride reduction with different EuL2 loading.

Table S5.

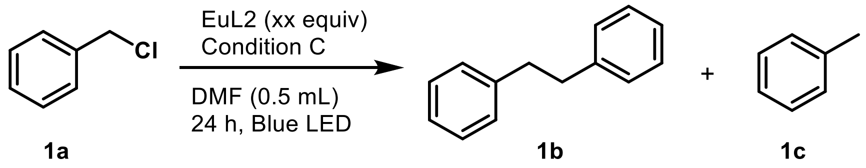

Entry	Condition	EuL2	Light source	GC-Yield	
				**1b**	**1c**
1	**C**	2%	BlueLED	0	1
2	**C**	5%	BlueLED	20%	2
3	**C**	20%	BlueLED	95%	3

Catalytic benzyl halide reduction using Eu(II)L1.

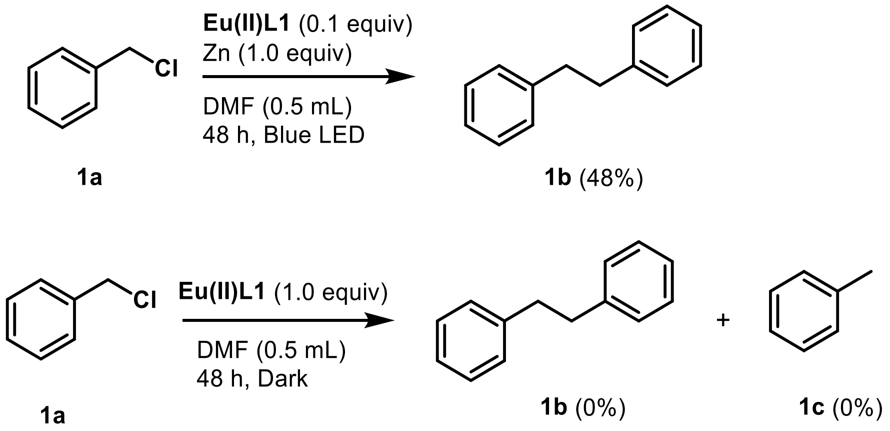

Scheme S2. Benzyl chloride reduction using **Eu(II)L1** and stoichiometric dark reaction.

Table S6. Radical quenching experiments.

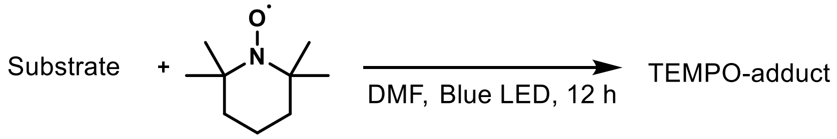

Entry	Substrate	TEMPO-adduct (%LC)
1	**24a**	8%
2	**EuL1**	~1%
3	**GdL1**	0

Ligand and LnL syntheses

Synthesis of L1

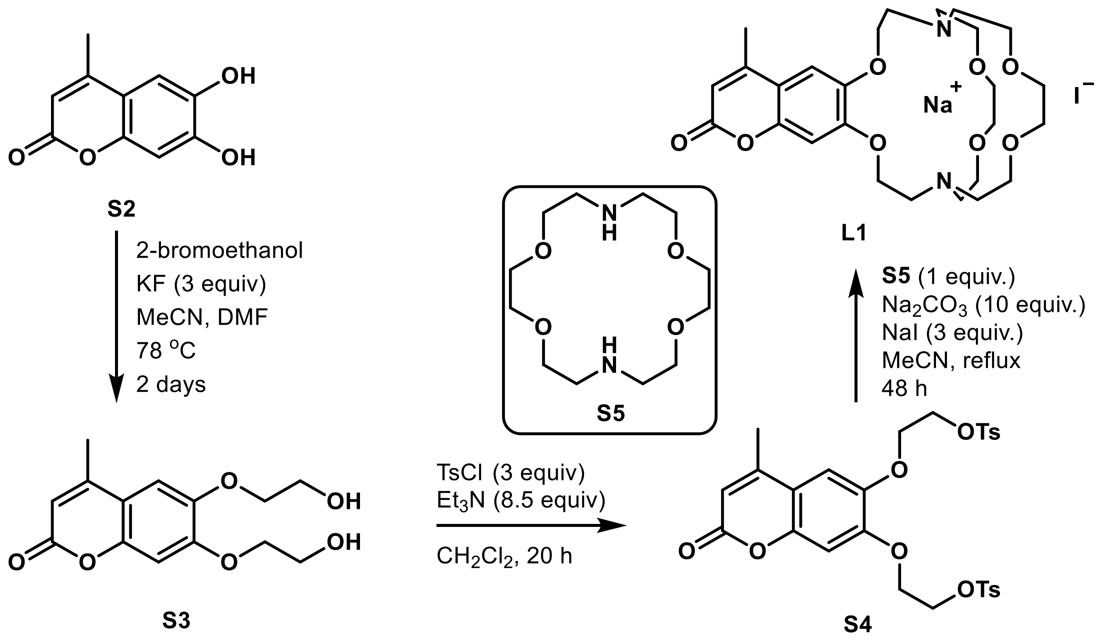

Scheme S3. Synthesis of **L1**.

S3. Known compound, prepared using a modified procedure.[6] To a solution of 6,7-dihydroxy-4-methyl coumarin (**S2**, 4.00 g, 20.8 mmol, 1.0 equiv) and 2-bromoethanol (4.43 mL, 62.4 mmol, 3.0 equiv)) in a mixture of anhydrous MeCN (160 mL) and DMF (32 mL), anhydrous KF (3.63 g, 62.4 mmol, 3.0 equiv) was added under a nitrogen atmosphere. The resulting mixture was heated at 78 °C for 24 h. The reaction was monitored by TLC and LCMS analysis. Another two portions of KF (1.813 g, 31.21 mmol, 1.5 equiv) and bromoethanol (2.21 mL, 31.21 mmol, 1.5 equiv) were added each after 24 h, and stirred for another two days. After 3 days, the orange-brownish mixture was filtered through filter paper. The filtrate was evaporated to dryness yielding an orange-brownish powder that was purified by column chromatography on silica gel (0→10% MeOH in CH_2Cl_2) yielding a white solid (4.08 g, 70%): R_f (5% MeOH:CH_2Cl_2) = 0.35; HPLC: Method LC1, t_r = 2.59 min; ^{1}H NMR (400 MHz, DMSO-d_6) δ 7.19 (s, 1H), 7.06 (s, 1H), 6.19 (d, J = 1.5 Hz, 1H), 4.92 (s, 2H), 4.11–4.07 (m, 4H), 3.74 (q, J = 5.5 Hz, 4H), 2.40 (s, 3H); MS (ESI, *m/z*): calcd for $C_{14}H_{17}O_6$ [M+H$^+$] 281.1, found 281.1.

S4. To a solution of **S3** (2.30 g, 8.20 mmol, 1.0 equiv.) in CH_2Cl_2 (30 mL), triethylamine (10.0 mL, 69.7 mmol, 8.5 equiv) was added, and the solutions was stirred for 10 mins under and Ar atmosphere. Then a solution *p*-TsCl (4.69 g, 24.6 mmol, 3.0 equiv) in CH_2Cl_2 (15 mL) was added. The reaction mixture immediately turned into a clear yellow solution, which then became cloudy after 20–30 mins. Stirring was continued for 20 h at r.t. The reaction was

monitored by TLC and LCMS analysis, which showed the consumption of the starting material after 20 h. LCMS analysis showed the presence of a mixture of mono- and dialkylated products. The reaction mixture was poured directly onto a silica gel-packed chromatography column, and the crude product was purified using a FlashMaster system and EtOAc:heptane (1:19→1:0) as the eluent. Evaporation of the solvents and drying of the solid residue under vacuum yielded a yellowish crystalline powder (2.03 g, 42%): R_f (EtOAc:heptane, 6:4) = 0.42; HPLC: Method LC1, t_r = 7.00 min; ^{1}H NMR (400 MHz, DMSO-d_6) δ 7.78–7.75 (m, 4H), 7.40 (dd, J = 8.5, 3.5 Hz, 4H), 7.11 (s, 1H), 6.97 (s, 1H), 6.21 (d, J = 1.5 Hz, 1H), 4.36–4.24 (m, 8H), 2.36 (s, 6H), 2.35 (s, 3H); ^{13}C NMR (101 MHz, DMSO-d_6) δ 160.2, 153.4, 151.1, 148.9, 145.0, 144.0, 145.0, 132.1, 132.0, 130.1, 127.6, 112.5, 111.7, 109.6, 101.6, 69.0, 68.6, 67.0, 66.4, 21.1, 18.3; HRMS (ESI, *m/z*) calcd for $C_{28}H_{28}O_{10}S_2Na$ [M+Na$^+$]: 611.1016, found 611.1014.

L1. Known compound prepared using a modified procedure.[6] To a solution of **S4** (1.00 g, 1.69 mmol, 1.0 equiv) in dry acetonitrile (10.0 mL) Na_2CO_3 (1.79 g, 16.9 mmol, 10 equiv) and NaI (759 mg, 5.07 mmol, 3.0 equiv) were added, followed by a single portion of Kryptofix-22 (714 mg, 1.69 mmol, 1.0 equiv). The resulting mixture was heated at reflux for 48 h. When TLC and LCMS analyses indicated the completion of the reaction the solvent was evaporated to yield a pale yellow solid, which was suspended in chloroform. The suspension was filtered, resulting filtrate was dried and purified by column chromatography on silica gel (0→2% MeOH in CH_2Cl_2) to give **L1** (615 mg, 55%) as a pale yellow crystalline product (614 mg, 55%). R_f (5% MeOH:CH_2Cl_2) = 0.32; HPLC: Method LC2, t_r = 0.50 min; ^{1}H NMR (400 MHz, $CDCl_3$) δ 7.29 (s, 1H), 6.83 (s, 1H), 6.19 (d, J = 1.0 Hz, 1H), 4.47–4.44 (m, 2H), 4.29–4.27 (m, 2H), 3.73–3.64 (m, 8H), 3.60–3.55 (m, 6H), 3.42–3.36 (m, 2H), 2.97–2.95 (m, 2H), 2.89–2.86 (m, 2H), 2.81–2.72 (m, 6H), 2.70–2.67 (m, 2H), 2.52 (d, J = 1.0 Hz, 3H); MS (ESI, *m/z*) calcd for $C_{26}H_{39}N_2O_8$ [M+H$^+$]: 507.2, found 507.2.

General procedure for LnL1 synthesis

To a solution of **L1** (1.0 equiv) in acetonitrile Ln(OTf)$_3$ (1.0 equiv) was added as a single portion. The resulting yellow solution was heated at 80 °C for 4–5 days. During this time the solution turned to reddish brown. The solvent was evaporated at reduced pressure yielding colored powders.

Eu(III)L1/ EuL1: crystalline reddish brown powder (700 mg, 80%): ^{1}H NMR (400 MHz, D_2O) δ 25.6, 24.4, 15.5, 14.1, 13.0, 12.0, −2.0, −2.5, −4.7, −13.8, −21.5; HRMS (ESI, *m/z*) calcd

for $C_{26}H_{38}N_2O_8EuI$ $[M+I]^{2+}$ 393.0437, found 393.0438; λ_{max}(DMF) = 339 nm, λ_{em}(DMF) = 412, 616 nm.

SmL1: reddish powder, (100 mg, 65%): HRMS (ESI, *m/z*) calcd for $C_{26}H_{38}N_2O_8SmI$ $[M+I]^{2+}$ 392.5429, found 392.5429; λ_{max}(DMF) = 339 nm, λ_{em}(DMF) = 412 nm.

GdL1: yellow-reddish powder, (50 mg, 70%): HRMS (ESI, *m/z*) calcd for $C_{26}H_{38}N_2O_8SmI$ $[M+I]^{2+}$ 392.5451, found 395.5454; λ_{max}(DMF) = 339 nm, λ_{em}(DMF) = 415 nm.

DyL1: reddish powder, (20 mg, 78%): λ_{max}(DMF) = 339 nm, λ_{em}(DMF) = 416 nm.

Eu(II)L1: In an Ar-filled glove box a solution of **L1** (50 mg, 0.094 mmol, 1.0 equiv) in anhydrous THF (2.0 mL) was added to a clear solution of EuI_2 (38 mg, 0.094 mmol, 1.0 equiv) in THF (2.0 mL) in a microwave vial. The vial was sealed, taken out of the glove box, and the reaction mixture was heated at 80 °C for 4–5 days. The reaction mixture turned reddish-brown during this time, and a precipitate formed on the wall of the vial. The solvent was removed with the help of syringe from the reaction mixture, the solid residue was washed with THF thrice, and was then dried under vacuum yielding a reddish powder was obtained (55 mg, 87%): HRMS (ESI, *m/z*) calcd for $C_{26}H_{38}N_2O_8EuI$ $[M+I]^{2+}$ 393.0437, found 393.0438; λ_{max} (DMF) = 350 nm, λ_{em}(DMF) = 434 nm.

Synthesis of L2

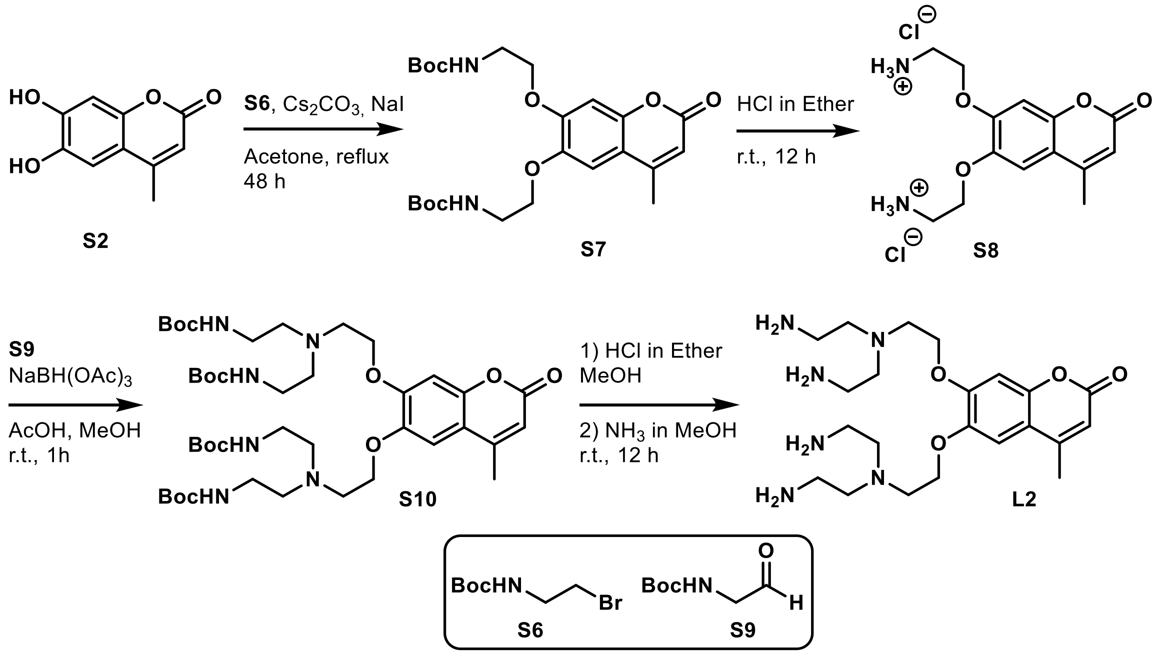

Scheme S4. Synthesis of **L2**.

S7. A 250-mL two-necked round-bottomed flask was charged with **S2** (2.00 g, 10.4 mmol, 1.0 equiv), **S6** (11.7 g, 52.0 mmol, 5.0 equiv), Cs_2CO_3 (16.9 g, 52.0 mmol, 5.0 equiv) and NaI

(780 mg, 5.2 mmol, 0.5 equiv), followed by acetone (100 mL). The resulting suspension was heated at 80 °C for 48 h. When TLC and LC-MS analyses indicated the completion of the reaction the mixture was diluted with water (50 mL) and the mixture was extracted twice with EtOAc. The combined organic layer was dried over Na_2SO_4, filtered, and the filtrate was concentrated under reduced pressure. The solid residue was purified using silica gel column chromatography with EtOAc:heptane (2:8) as the eluent to yield a white solid (3.28 g, 66%): R_f (40% EtOAc in heptane) = 0.32; HPLC: Method LC1, t_r = 5.89 min. ^{1}H NMR (400 MHz, $CDCl_3$) δ 7.07 (s, 1H), 6.83 (s, 1H), 6.15 (s, 1H), 5.22 (d, *J* = 20.0 Hz, 2H), 4.11–4.07 (m, 4H), 3.56 (dd, *J* = 20.0, 5.0 Hz, 4H), 2.37 (s, 3H), 1.45 (s, 18H); ^{13}C NMR (101 MHz, $CDCl_3$) δ 161.3, 156.0, 152.7, 152.2, 150.0, 145.3, 113.4, 112.9, 110.6, 102.2, 79.9, 70.4, 69.0, 40.3, 40.0, 28.5, 18.9; HRMS (ESI, *m/z*) calcd for $C_{24}H_{34}N_2O_8Na$ [M+Na$^+$] 501.2207, found 501.2207.

S8. To a stirred solution of **S7** (3.46 g, 6.27 mmol, 1.0 equiv) in MeOH (30 mL) a solution of HCl in ether (2.0 M, 7.8 mL, 2.5 equiv) was added at room temperature. The reaction mixture was stirred for 16 h during which a white suspension was formed. The reaction mixture was concentrated under reduced pressure and washed with diethyl ether (10 mL) to get an off-white/yellowish solid (1.78 g, 81%): HPLC: Method LC2, t_r = 0.50 min. ^{1}H NMR (400 MHz, DMSO-d_6) δ 8.43 (s, 6H), 7.32 (s, 1H), 7.17 (s, 1H), 6.26 (s, 1H), 4.32–4.28 (m, 4H), 3.37–3.23 (m, 4H), 2.42 (s, 3H); ^{13}C NMR (101 MHz, DMSO-d_6) δ 160.8, 153.9, 152.0, 149.7, 144.8, 113.2, 112.3, 110.3, 102.1, 79.9, 79.5, 66.8, 66.2, 19.0; HRMS (ESI, *m/z*) calcd for $C_{14}H_{19}N_2O_4$ [M+H$^+$] 279.1339, found 279.1339.

S10. The reaction was carried out in triplicate on the scale indicated in the protocol (0.86 mmol), as reactions carried out on a larger scale yielded unacceptable quantities of side products. To a stirred solution of **S8** (302 mg, 0.86 mmol, 1.0 equiv) in MeOH (10 mL), triethylamine (432 mg, 0.62 mL, 4.28 mmol, 5.0 equiv) was added. The mixture was stirred for 15 mins, after which **S9** (411 mg, 2.58 mmol, 3.0 equiv) and $NaBH(OAc)_3$ (547 mg, 2.58 mmol, 3.0 equiv) were added. After 1 h a second portion of **S10** (684 mg, 4.30 mmol, 5.0 equiv) and $NaBH(OAc)_3$ (912 mg, 4.30 mmol, 5.0 equiv) were added, and stirring was continued at room temperature for 48 h. When TLC analysis indicated the completion of the reaction the three parallel reaction mixtures were combined, and were concentrated under reduced pressure. The crude product was purified using silica gel column chromatography using MeOH in CH_2Cl_2 (2%) as the eluent. Evaporation of the solvents and drying the residue under vacuum yielded a pale yellow solid (724 mg, 33%): R_f (5% MeOH in CH_2Cl_2) = 0.63; HPLC: Method LC1, t_r = 4.60 min. ^{1}H NMR (400 MHz, $CDCl_3$) δ 6.95 (s, 1H), 6.81 (s, 1H), 6.15 (s, 1H), 5.21 (s, 4H),

4.08–4.05 (m, 4H), 3.19–3.15 (m, 8H), 2.96–2.94 (m, 4H), 2.68 (d, J = 6.5 Hz, 8H), 2.39 (d, J = 1.0 Hz, 3H), 1.36 (s, 36H); ^{13}C NMR (101 MHz, $CDCl_3$) δ 161.5, 156.3, 152.3, 152.03, 149.4, 145.4, 112.6, 112.5, 106.4, 100.9, 79.1, 68.0, 54.5, 52.8, 52.5, 38.6, 32.0, 29.7, 28.4, 22.7, 18.9, 14.2; HRMS (ESI, *m/z*) calcd for $C_{42}H_{70}N_6O_{12}$ Na [M+Na$^+$] 873.4943, found 873.4943.

L2. To a stirred solution of **S10** (724 mg, 0.850 mmol, 1.0 equiv) in MeOH (10.0 mL), a solution of HCl in ether (2.0 M, 4.24 mL, 10 equiv) was added at room temperature. The reaction mixture was stirred overnight, during which a white precipitate formed. The mixture was concentrated under reduced pressure. Ammonia (4% in MeOH, 10.0 mL) was added, and the mixture was stirred overnight. The mixture was filtered, and the filtrate was washed with diethyl ether (5.0 mL) to yield a yellow-brown solid (364 mg, 95%): HPLC: Method LC2, t_r = 0.39 min; ^{1}H NMR (400 MHz, D_2O) δ 7.52 (s, 1H), 7.14 (s, 1H), 7.01 (s, 1H), 6.16 (s, 1H), 4.19–4.18 (m, 4H), 3.04–3.03 (m, 12H), 2.87–2.86 (m, 8H), 2.33 (s, 3H); ^{13}C NMR (101 MHz, D_2O) δ 164.7, 156.3, 148.8, 144.5, 111.0, 108.0, 101.4, 86.05, 77.7, 66.0, 57.7, 50.7, 43.8, 38.6, 36.3, 34.1, 29.6, 22.7, 18.2, 16.7; HRMS (ESI, *m/z*) calcd for $C_{22}H_{39}N_6O_4$ [M+H$^+$] 451.3027, found 451.3029.

General procedure for synthesis of LnL2.

To a vial containing a solution of **L2** (0.18 g, 0.40 mmol, 1.0 equiv) in MeOH: H_2O (2:4 mL) anhydrous $LnCl_3$ (0.40 mmol, 1.0 equiv) was added. The resulting brown solution was heated at 60 °C for 72 h, during which a precipitate usually formed. The solid was collected by filtration, and was washed with CH_2Cl_2 (2×5 mL) and MeOH (2×5 mL) to remove the excess ligand. The resulting brown solid was dried under reduced pressure. On a smaller scale instead of precipitate formation the solution became cloudy. The sample was then concentrated, and the residue was washed with CH_2Cl_2 and MeOH to remove excess ligand.

EuL2. Brown solid (40 mg, 13%): ^{1}H NMR (400 MHz, DMSO-d_6) δ −1.61, −3.63, −25.46; HRMS (ESI, *m/z*) calcd for $C_{26}H_{38}N_2O_8EuCl_2$ [M+2Cl]$^+$ 673.1538, found 673.1532; λ_{max} (DMF) = 341 nm, λ_{em} (DMF) = 418, 616, 701 nm.

SmL2. Brown solid (22 mg, 8%): HRMS (ESI, *m/z*) calcd for $C_{26}H_{38}N_2O_8SmCl_2$[M+2Cl]$^+$ 672.1529, found 672.1528; λ_{max} (DMF) = 339 nm, λ_{em} (DMF) = 417 nm, 601.

GdL2. Pale yellow solid (85 mg, 30%): λ_{max} (DMF) = 340 nm, λ_{em} (DMF) = 416 nm.

DyL2. Brown solid (25 mg, 9%): λ_{max} (DMF) = 339 nm, λ_{em} (DMF) = 415 nm.

General procedure for synthesis of LnL3.

To a vial containing solution of **L3** (0.20 g, 1.1 mmol, 1.0 equiv) in MeOH (1 mL), anhydrous $LnCl_3$ (1.1 mmol, 1.0 equiv) was added. The resulting yellow solution was heated at 60 °C for 24 h during which it turned greenish brown. Then, reaction mixture was concentrated under reduced pressure and the resulting greenish brown residue was washed with CH_2Cl_2 (2×20 mL) to remove excess of ligand, dried under reduced pressure.

EuL3. Brown solid (74 mg, 15%): ^{1}H NMR (400 MHz, DMSO-d_6) δ 11.12, −1.61, −3.63, −25.46; HRMS (ESI, *m/z*) calcd for $(C_{10}H_9N_2O)_4EuH_3$ $[M+3H]^{2+}$ 424.1147, found 424.1149; λ_{max}(DMF) = 338, 353 nm, λ_{em}(DMF) = 385 nm.

GdL3. Greenish solid (25 mg, 5%): HRMS (ESI, *m/z*) calcd for $(C_{10}H_9N_2O)_4GdH_3$ $[M+3H]^{2+}$ 426.6162, found 426.6166; λ_{max}(DMF) = 338, 353 nm, λ_{em}(DMF) = 385 nm.

Synthesis of model ligand (L2m)

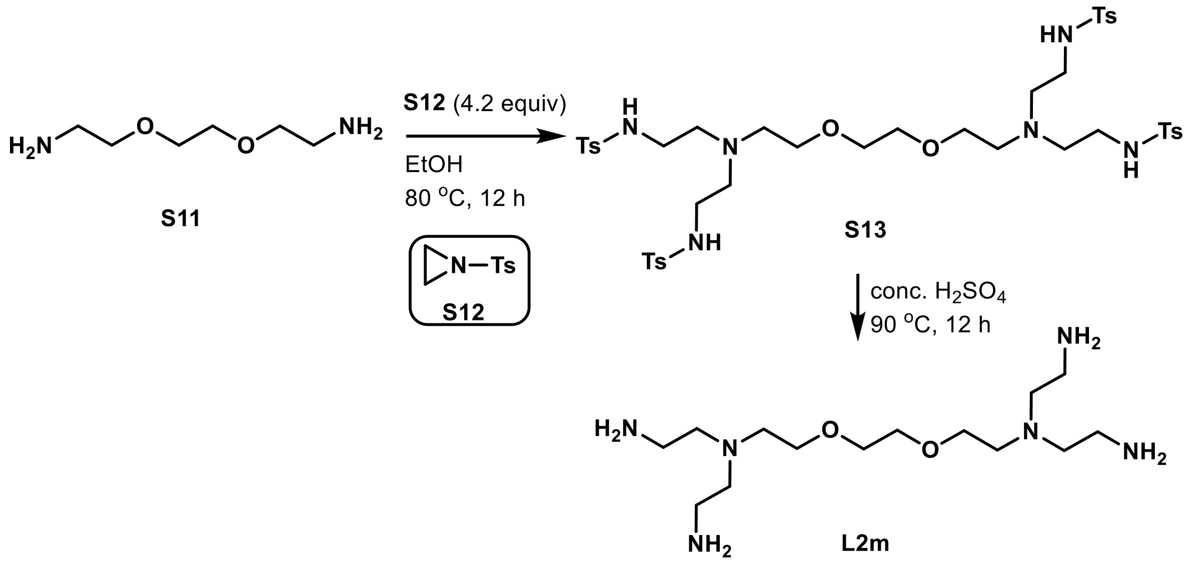

Scheme S5. Synthesis of model ligand (**L2m**).

S13. To a 10-mL two-necked round-bottomed flask containing **S11** (100 g, 0.67 mmol, 1.0 equiv) in EtOH (5.0 mL), *N*-tosyl aziridine (664 mg, 3.37 mmol, 5.0 equiv) was added. The resulting solution was heated at 80 °C for 12 h, and the reaction was monitored using TLC and LC-MS analysis. Upon completion of reaction, it was allowed to cool to room temperature and concentrated under reduced pressure. The resulting solid mixture was washed with Et_2O (2×5 mL), dried under reduced pressure to obtain as a white solid (402 mg, 64%); R_f (2% MeOH in CH_2Cl_2) = 0.6; HPLC: Method LC1, t_r = 5.31 min; ^{1}H NMR (400 MHz, $CDCl_3$) δ 7.62 (d, J = 8.0 Hz, 8H), 7.32 (d, J = 8.0 Hz, 8H), 3.20 (m, 3H), 2.67–2.62 (m, 6H), 2.39–2.28 (m, 22H); ^{13}C NMR (101 MHz, $CDCl_3$) δ 143.2, 136.9, 129.8, 127.2, 70.3, 68.9, 66.9, 53.6, 53.0, 40.7, 21.6; HRMS (ESI, *m/z*) calcd for $C_{42}H_{60}N_6O_{10}S_4$ $[M+Na]^+$ 959.3146, found 959.3146.

L2m. Known compound prepared using a modified procedure.[7] To a 10-mL two-necked round-bottomed flask containing **S13** (0.10 g, 0.38 mmol, 1.0 equiv) conc. H_2SO_4 (0.5 mL) was added. Then, resulting mixture was stirred at 90 °C for 24 h, and the reaction was monitored using LCMS analysis. Upon completion of reaction, the reaction mixture was diluted with Et_2O (2×5 mL) to obtain yellow precipitates. The ether layer was decanted and solid residue was neutralized with methanolic ammonia solution (conc. 4%). The resulting solution dried under reduced pressure to obtain yellow liquid (31 mg, 90%): ^1H NMR (400 MHz, D_2O) δ 3.56–3.49 (m, 8H), 3.00–2.97 (m, 8H), 2.76–2.70 (m, 8H), 2.67–2.64 (m, 4H). HPLC: Method LC2, t_r = 0.39 min.

EuL2m: To a vial containing a solution of **L2m** (0.03 g, 0.096 mmol, 1.0 equiv) in water (4 mL), anhydrous $EuCl_3$ (0.025 g, 0.096 mmol, 1.0 equiv) was added. The resulting brown solution was heated at 60 °C for 72 h, during which complex usually precipitated. Then, precipitates were filtered and washed with MeOH (2×2 mL) and water (2×2 mL) to remove excess of ligand. The resulting brown solid was dried under reduced pressure to obtained the desired complex as a colorless solid (37 mg, 67%): ^1H NMR (400 MHz, D_2O) δ –11.41, –10.34; λ_{max} (DMF) = 280 nm, λ_{em} (DMF) = 613, 701 nm.

Synthesis of starting materials

Procedure for the synthesis of 12a

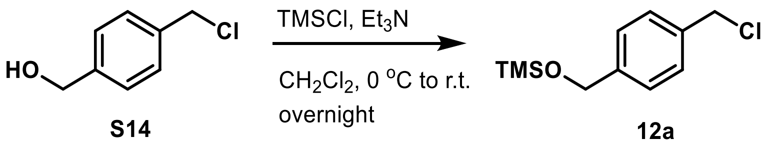

Scheme S6. Synthesis of **12a**.

12a. Known compound, previously reported without characterization.[8] Following a literature procedure, a vial containing **S14** (0.21 g, 1.36 mmol, 1.0 equiv) and triethylamine (0.30 mL, 2.01 mmol, 1.5 equiv) in CH_2Cl_2 (5.0 mL), a single portion of TMSCl (0.19 mL, 1.48 mmol, 1.1 equiv) was added at 0 °C. The resulting solution was stirred at room temperature for 16 h, and the reaction progress was monitored using TLC. Upon completion of the reaction the precipitate that formed was filtered out. The filtrate was concentrated under reduced pressure. The resulting solid was purified using silica gel column chromatography with heptane as the eluent to obtain a colorless liquid (0.23 g, 77%): R_f (EtOAc:heptane, 10%) = 0.7; ^{1}H NMR (400 MHz, $CDCl_3$) δ 7.33 (m, 4H), 4.68 (s, 2H), 4.57 (s, 2H), 0.15 (s, 9H); HRMS (ESI, *m/z*) calcd for $C_{44}H_{30}BrN_4$ $[M+H]^+$ 229.0810, found 229.0810.

Procedure for the synthesis of 17a.

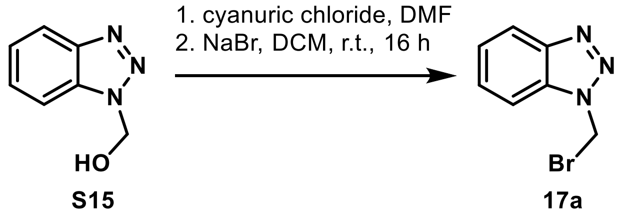

Scheme S7. Synthesis of **17a**.

Known compound, modified procedure.[9] To a 50-mL round-bottomed flask cyanuric chloride (0.92 g, 5.2 mmol, 1.1 equiv), DMF (1.0 mL) was added at r.t. The mixture was stirred until TLC analysis indicated the consumption of cyanuric chloride. The reaction mixture was diluted with CH_2Cl_2 (15.0 mL), and NaBr (0.98 g, 9.4 mmol, 2.0 equiv) was added. The reaction mixture was stirred for 10 h and then **S15** (0.70 g, 4.7 mmol, 1.0 equiv) was added. The resulting suspension was stirred at r.t. until TLC analysis showed the disappearance of the starting material. Upon completion of the reaction the mixture was diluted with water (10 mL). The layers were separated, and the organic layer was concentrated under reduced pressure. The crude product was purified using silica gel column chromatography (1.2:8.8 = EtOAc:heptane).

A colorless solid was obtained (0.48 g, 49%): R_f (20% EtOAc:heptane) = 0.41; ^{1}H NMR (400 MHz, $CDCl_3$) δ 8.11 (d, J = 8.0 Hz, 1H), 7.67 (m, 1H), 7.60 (m, 1H), 7.48–7.44 (m, 1H), 6.40 (s, 2H).

General procedure for the syntheses of 27a and 28a.

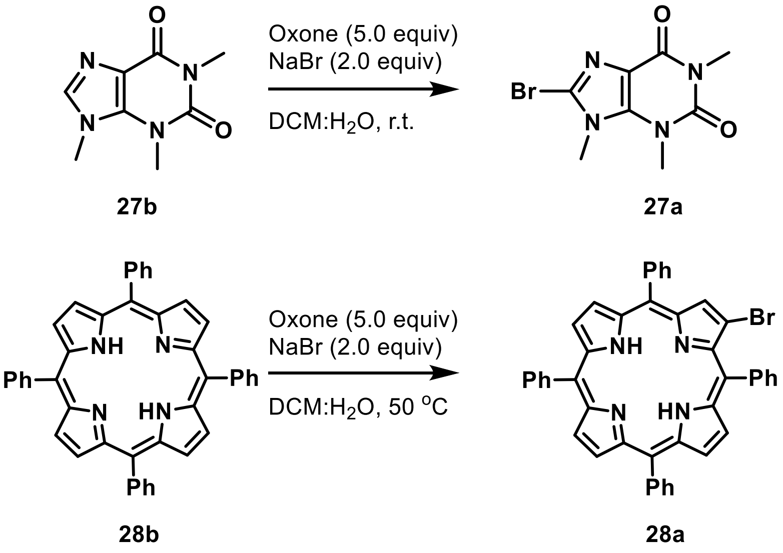

Scheme S8. Synthesis of **27a** and **28a**.

To a vial containing **27b-28b** (1.0 equiv) in CH_2Cl_2 (3.0–15.0 mL), oxone (5.0 equiv) and NaBr (2.0 equiv) in water (1.0 mL) was added at r.t. The resulting solution was stirred at room temperature or at 50 °C overnight. The reaction progress was monitored by TLC analysis. Upon completion of the reaction the solvent layers were separated, and the CH_2Cl_2 layer was concentrated under reduced pressure.

27a. Known compound.[10] The resulting crude mixture was purified using silica gel column chromatography (MeOH:CH_2Cl_2, 2:98) to obtain **27a** as a colorless solid (0.49 g, 85%): R_f (EtOAc:heptane, 4:6) = 0.42; ^{1}H NMR (400 MHz, DMSO-d_6) δ 3.80 (s, 3H), 3.34 (s, 3H), 3.17 (s, 3H).

28a. Known compound, new characterization data.[11] The resulting crude mixture was purified using silica gel column chromatography (CH_2Cl_2:pentane, 2:98) to obtain **28a** as a purple solid (9 mg, 41%): R_f (EtOAc:heptane, 1:9) = 0.55; ^{1}H NMR (400 MHz, $CDCl_3$) δ 8.89–8.75 (m, 7H), 8.20–8.15 (m, 5H), 8.09–8.05 (m, 3H), 7.79–7.69 (m, 12H), –2.92 (brs, 2H); HRMS (ESI, m/z) calcd for $C_{44}H_{30}BrN_4$ $[M+H]^+$ 695.1633, found 695.1637.

Synthetic procedures and product characterization of Ln(II)-catalyzed reductions

1. General procedures for small scale (0.015 mmol–0.4 mmol) reactions

Procedure for Conditions A and B: In an Ar-filled glovebox **LnL1, LnL2 or LnL3** (0.05–0.1 equiv.), substrate (1.0 equiv.), sacrificial reductant or additive (1.0 equiv.) were added to a vial, followed by anhydrous DMF (28−32 mM). The vial was sealed and covered with an electrical tape, taken out of the glovebox, and was placed in a photoreactor. The mixture was stirred for the specified time. Often the reaction mixture changed colour from colourless/light yellow to green/dark brown. The solutions were then analysed using GCMS or TLC analysis.

Note: Performing the same procedure with Schlenk techniques instead of in a glovebox gave similar results.

Procedure for Condition C: In an Ar-filled glovebox **LnL1, LnL2 or LnL3** (0.1 equiv), substrate (1.0 equiv), DIPEA (10.0 equiv), and LiCl (10.0 equiv) were added to a vial, followed by anhydrous DMF (28−32 mM). The vial was sealed with electrical tape. The vial was brought out of the glovebox. H_2O (20% with respect to DMF) was added to the reaction mixture and the sample was degassed by bubbling a stream of Ar through it for 20 mins. The vial was placed in a photoreactor, and the reaction mixture was stirred for the specified time. The crude products were analysed using GCMS or TLC analysis.

Note: Performing the same procedure with Schlenk technique instead of glovebox also produces similar results. Degassing with Ar is absolutely necessary for good results.

2. Procedure for scale-up reaction of Brittonin A (4.3 mmol)

In an Ar-filled glovebox **EuL1** (0.05 equiv.), **18a** (1.0 equiv.), DIPEA (10.0 equiv), and LiCl (10.0 equiv) were added to a two-neck round-bottom flask, followed by anhydrous DMF (140 mL). It was taken out of the glovebox and H_2O (20% v/v with respect to DMF) was added to the reaction mixture. The sample was degassed by bubbling a stream of Ar through it for 20 mins. The vial was placed in a photoreactor under Ar atmosphere, and the reaction mixture was stirred for 12 h. The crude products were analysed using GCMS or TLC analysis.

3. General procedure for stoichiometric reaction with SmI_2

In an Ar-filled glovebox the substrate (1.0 equiv.) and SmI_2 dissolved in THF (0.1 M, 2 equiv.) were added to a vial. In the case of aromatic dehalogenation ^{i}PrOH (20 equiv.) was also added. The mixture was stirred at room temperature for 30 min. The mixture changed colour from

bluish-green to light yellow within 5–20 mins. The samples were then analysed using GCMS or TLC analyses.

4. Characterization data

All reactions were carried out in duplicates. Isolated yields are the average from the two separate batches of the same scale (0.166–0.4 mmol) performed simultaneously.

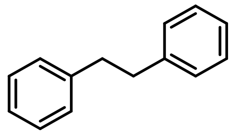

1,1'-(Ethane-1,2-diyl)dibenzene (1b). Known compound.[12] (Table S1, entry 14). The reaction mixture was purified using silica-gel column chromatography using EtOAc:heptane (2:98) as an eluent. A white powder was obtained. R_f (EtOAc:heptane, 1:9) = 0.74. (60 mg, 90%); ^{1}H NMR (400 MHz, $CDCl_3$) δ 7.30–7.25 (m, 4H), 7.21–7.17 (m, 6H), 2.92 (s, 4H).

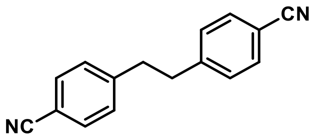

4,4'-(Ethane-1,2-diyl)dibenzonitrile (2b). Known compound.[13] The reaction mixture was purified using silica-gel column chromatography using EtOAc:heptane (3:97) as an eluent. An off-white powder was obtained. R_f (EtOAc:heptane, 1:9) = 0.70; ^{1}H NMR (400 MHz, $CDCl_3$) δ 7.57 (d, J = 8.0 Hz, 4H), 7.21 (d, J = 8.0 Hz, 4H), 2.99 (s, 4H); MS (EI, *m/z*) calcd for $C_{16}H_{12}N_2$ = 232.1 $[M]^+$, found 232.1.

Table S7. Synthesis of **2b** under **Conditions B** and **C**.

Entry	LnL	Condition	Light source	2b (%)
1	**EuL1**	**B**	365 nm	42 (8 mg)
2	**EuL2**	**C**	Blue LED	80 (15 mg)
3	**SmL1**	**B**	365 nm	88 (17 mg)
4	**SmL2**	**C**	Blue LED	73 (14 mg)
5	**GdL1**	**B**	365 nm	0
6	**GdL2**	**C**	Blue LED	0

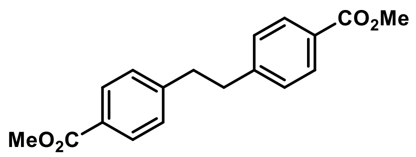

Methyl 4,4'-(ethane-1,2-diyl)dibenzoate (3b). Known compound.[13] The crude product was purified using silica gel column chromatography using EtOAc:heptane (20:80) as the eluent. An off-white powder was obtained. R_f (EtOAc:heptane, 2:8) = 0.35; ^{1}H NMR (400 MHz, $CDCl_3$) δ 7.93 (d, *J* = 8.0 Hz, 4H), 7.18 (d, *J* = 8.0 Hz, 4H), 3.89 (s, 6H), 2.98 (s, 4H); MS (EI, m/z) calcd for $C_{18}H_{18}O_4$ = 298.1 $[M]^+$, found 298.1.

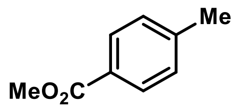

Methyl 4-methylbenzoate (3c). Known compound.[13,14] The crude product was purified using silica gel column chromatography using EtOAc:heptane (5:95) as the eluent. A colorless liquid was obtained. R_f (EtOAc:heptane, 2:8) = 0.62; ^{1}H NMR (400 MHz, $CDCl_3$) δ 7.93–7.91 (m, 2H), 7.22 (dd, *J* = 8.5, 1.0 Hz, 2H), 3.89 (s, 3H), 2.39 (s, 3H); MS (EI, *m/z*) calcd for $C_9H_{10}O_2$ = 150.0 $[M]^+$, found 150.0.

Table S8. Synthesis of **3b** and **3c** under **Conditions A**, **B** and **C**.

Entry	LnL	Condition	Light Source	3b (%)	3c (%)
1	**EuL1**	**B**	365 nm	0	0
2	**EuL2**	**C**	Blue LED	42 (10 mg)	0
3	**EuL2**	**A**	Blue LED	0	53 (13 mg)

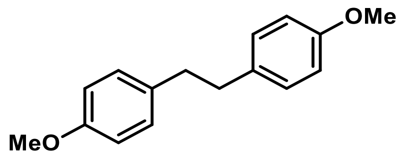

1,1'-(Ethane-1,2-diyl)bis(4-methoxybenzene) (4b). Known compound.[12] The crude product was purified using silica gel column chromatography using EtOAc:heptane (3:97) as the eluent. An off-white powder was obtained. R_f (EtOAc:heptane, 1:9) = 0.53; ^{1}H NMR (400 MHz, $CDCl_3$) δ 7.08 (d, *J* = 8.5 Hz, 4H), 6.82 (d, *J* = 8.5 Hz, 4H), 3.79 (s, 6H), 2.83 (s, 4H); MS (EI, *m/z*) calcd for $C_{16}H_{18}O_2$ = 242.1 $[M]^+$, found 242.1.

Table S9. Synthesis of **4b** under **Conditions A**, **B** and **C**.

Entry	LnL	Condition	Light source	4b (%)
1	**EuL1**	**B**	365 nm	16 (3 mg)
2	**EuL1**	**A**	Blue LED	35 (7 mg)
3	**EuL2**	**C**	Blue LED	10 (2 mg)

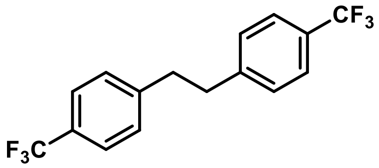

1,1'-(Ethane-1,2-diyl)bis(4-(trifluoromethyl)benzene) (5b). Known compound.[15] The crude product was purified using silica gel column chromatography using EtOAc:heptane (3:97) as the eluent. An off-white powder was obtained. R_f (EtOAc:heptane, 5:95) = 0.43; ^{1}H NMR (400 MHz, CD_2Cl_2) δ 7.54 (d, J = 8.0 Hz, 4H), 7.29 (d, J = 8.0 Hz, 4H), 3.01 (s, 4H); ^{19}F NMR (376 MHz, CD_2Cl_2) δ –62.55; MS (EI, *m/z*) calcd for $C_{16}H_{12}F_6$ = 318.0 $[M]^+$, found 318.0.

Table S10. Synthesis of **5b** under **Conditions A**, **B** and **C**.

Entry	LnL	Condition	Light source	5b (%)
1	**EuL1**	**A**	Blue LED	80 (21 mg)
2	**EuL1**	**B**	365 nm	10 (3 mg)
3	**EuL2**	**C**	Blue LED	62 (16 mg)

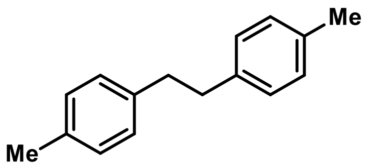

1,1'-(Ethane-1,2-diyl)bis(4-methylbenzene) (6b). Known compound.[12] The crude product was purified using silica gel column chromatography using EtOAc:heptane (3:97) as the eluent. An off-white powder was obtained. R_f (EtOAc:heptane, 1:9) = 0.43; ^{1}H NMR (400 MHz, $CDCl_3$) δ 7.10 (s, 8H), 2.86 (s, 4H), 2.33 (s, 6H); MS (EI, *m/z*) calcd for $C_{16}H_{18}$ = 210.1 $[M]^+$, found 210.1.

Table S11. Synthesis of **6b** under **Conditions A**, **B** and **C**.

Entry	LnL	Condition	Light Source	6b (%)

1	**EuL1**	**B**	365 nm	38 (7 mg)
2	**EuL1**	**A**	Blue LED	33 (6 mg)
3	**EuL2**	**A**	365 nm	0
4	**EuL2**	**C**	Blue LED	49 (9 mg)

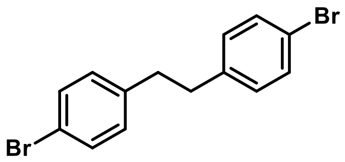

1,1'-(Ethane-1,2-diyl)bis(4-bromobenzene) (7b). Known compound.[13] The crude product was purified using silica gel column chromatography using EtOAc:heptane (3:97) as the eluent. An off-white powder was obtained. R_f (EtOAc:heptane, 1:9) = 0.43; ^{1}H NMR (400 MHz, $CDCl_3$) δ 7.38 (d, *J* = 8.5 Hz, 4H), 6.99 (d, *J* = 8.5 Hz, 4H), 2.84 (s, 4H); MS (EI, *m/z*) calcd for $C_{14}H_{12}Br_2$ = 339.9 $[M]^+$, found 339.9.

Table S12. Synthesis of **7b** under **Conditions A**, **B** and **C**.

**Entry**	**LnL**	**Condition**	**Light Source**	**7b (%)**
1	**EuL1**	**B**	365 nm	25 (7 mg)
2	**EuL1**	**A**	Blue LED	33 (9 mg)
3	**EuL2**	**C**	365 nm	0
4	**EuL2**	**A**	Blue LED	55 (16 mg)

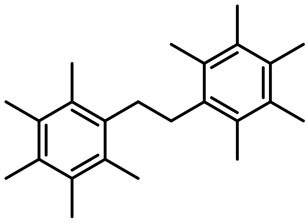

1,1'-(Ethane-1,2-diyl)bis(2,3,4,5,6-pentamethylbenzene) (8b). Known compound.[16] The crude product was purified using silica gel preparative column chromatography using EtOAc:heptane (3:97) as the eluent. A pale yellow powder was obatined. R_f (EtOAc:heptane, 1:9) = 0.48; ^{1}H NMR (400 MHz, $CDCl_3$) δ 2.40 (s, 4H), 2.28 (s, 12H), 2.24 (s, 18H). MS (EI, *m/z*) calcd for $C_{24}H_{34}$ = 322.2 $[M]^+$, found 322.2.

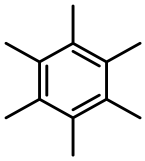

1,2,3,4,5,6-Hexamethylbenzene (8c). Known compound.[17] The reaction mixture was purified using silica-gel column chromatography using heptane as an eluent to obtain **8c** as a colorless solid. R_f (EtOAc:heptane, 1:9) = 0.5; ^{1}H NMR (400 MHz, $CDCl_3$) δ 2.24 (s, 1H); MS (EI, *m/z*) calcd for $C_{12}H_{18}$ = 162.1 $[M]^+$, found 162.1.

Table S13. Synthesis of **8b** under **Conditions A**, **B** and **C**.

Entry	LnL	Condition	Light source	8b (%)	8c (%)
1	**EuL1**	**B**	365 nm	20 (5 mg)	0
2	**EuL1**	**A**	Blue LED	40 (10 mg)	0
3	**EuL2**	**C**	Blue LED	0	30 (8 mg)

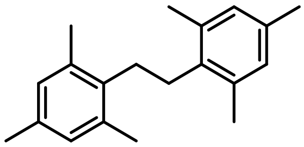

1,1'-(Ethane-1,2-diyl)bis(2,4,6-trimethylbenzene) (9b). Known compound.[18] The crude product was purified using silica gel column chromatography using EtOAc:heptane (3:97) as the eluent. An off-white powder was obtained. R_f (EtOAc:heptane, 1:9) = 0.46; ^{1}H NMR (400 MHz, $CDCl_3$) δ 6.86 (s, 4H), 2.77 (s, 4H), 2.36 (s, 12H), 2.27 (s, 6H); MS (EI, *m/z*) calcd for $C_{20}H_{26}$ = 266.2 $[M]^+$, found 266.2.

Table S14. Synthesis of **9b** under **Conditions A**, **B** and **C**.

Entry	LnL	Condition	Light source	9b (%)	9c (%)
1	**EuL1**	**B**	365 nm	21 (5 mg)	0
2	**EuL1**	**A**	Blue LED	80 (GC)	0
3	**EuL2**	**C**	Blue LED	62 (14 mg)	0
5	**SmL2**	**C**	Blue LED	45 (10 mg)	0
6	**GdL1**	**B**	365 nm	0	0
7	**GdL2**	**C**	Blue LED	0	0

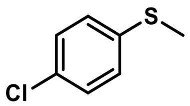

1-chloro-4-(methylsulfanyl)benzene (10c). Known compound.[19] The crude product was purified using silica gel column chromatography using EtOAc:heptane (3:97) as the eluent. A colorless oil was obtained. R_f (EtOAc:heptane, 1:9) = 0.58; ^{1}H NMR (400 MHz, $CDCl_3$) δ 7.25 (d, J = 8.0 Hz, 2H), 7.18 (d, J = 8.0 Hz, 2H), 2.47 (s, 3H); MS (EI, *m/z*) calcd for C_7H_7ClS = 158.0 $[M]^+$, found 158.0.

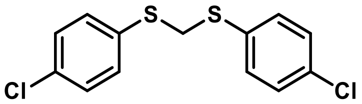

Impurity. Known compound.[20] The crude product was purified using preparative silica gel thin layer chromatography using EtOAc:heptane (10:90) as the eluent. A white solid was obtained. R_f (10% EtOAc:heptane) = 0.48. ^{1}H NMR and MS data are in accordance with the reported values.

Table S15. Synthesis of **10c** under **Conditions A**.

Entry	LnL	Condition	Light source	10c (%)
1	**EuL1**	**A**	Blue LED	38 (10 mg)<br>impurity (28%, 7 mg)
2	**EuL2**	**A**	Blue LED	70 (18 mg)

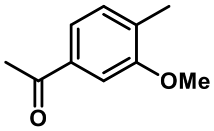

1-(3-Methoxy-4-methylphenyl)ethan-1-one (11c). Known compound.[21] The crude product was purified using silica gel column chromatography using EtOAc:heptane (8:92) as the eluent. A white powder was obtained. R_f (EtOAc:heptane, 2:8) = 0.45. ^{1}H NMR (400 MHz, $CDCl_3$) δ 7.81 (dd, J = 8.5, 2.0 Hz, 1H), 7.76 (d, J = 1.0 Hz, 1H), 6.83 (d, J = 8.5 Hz, 1H), 3.88 (s, 3H), 2.54 (s, 3H), 2.23 (s, 3H).

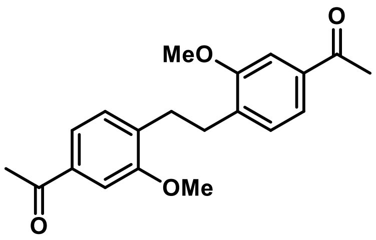

1,1'-(Ethane-1,2-diylbis(3-methoxy-4,1-phenylene))di(ethan-1-one) (11b). The crude product was purified using silica gel column chromatography using EtOAc:heptane (20:80) as the eluent. An off-white powder was obatined. R_f (EtOAc:heptane, 2:8) = 0.22. ^{1}H NMR (400 MHz, $CDCl_3$) δ 7.82 (dd, *J* = 8.5, 2.5 Hz, 2H), 7.71 (d, *J* = 2.5 Hz, 2H), 6.85 (d, *J* = 8.5 Hz, 2H), 3.88 (s, 6H), 2.92 (s, 4H), 2.52 (s, 6H); ^{13}C NMR (101 MHz, $CDCl_3$) δ 197.2, 161.7, 130.3, 128.9, 109.6, 55.7, 30.3, 26.4. HRMS (ESI, *m/z*) calcd for $C_{20}H_{22}O_4$ $[M+Na]^+$ 349.1410 found 349.1415.

Table S16. Synthesis of **11b** and **11c** under **Conditions A** and **C**.

**Entry**	**LnL**	**Condition**	**Light Source**	**11b (%)**	**11c (%)**
1	**EuL1**	**A**	Blue LED	30 (8 mg)	60 (16 mg)
3	**EuL2**	**A**	Blue LED	0	70 (19 mg)
4	**EuL2**	**C**	Blue LED	79 (21 mg)	0

(**Note**: Reaction was followed using TLC analysis as **11b** is not visible on GCMS.)

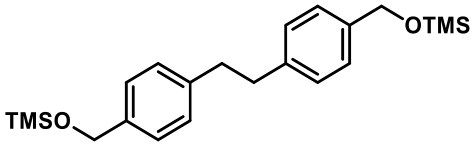

1,1'-(Ethane-1,2-diylbis(4,1-phenylenemethyleneoxy))bis(trimethylsilane) (12b). The crude product was purified using silica-gel column chromatography using EtOAc:heptane (5:95) as an eluent. A white powder was obtained. R_f (EtOAc:heptane, 5:95) = 0.45; ^{1}H NMR (400 MHz, $CDCl_3$) δ 7.22 (d, *J* = 7.5 Hz, 4H), 7.13 (d, *J* = 7.5 Hz, 4H), 4.65 (s, 4H), 2.88 (s, 4H), 0.14 (s, 18H); ^{13}C NMR (101 MHz, $CDCl_3$) δ 140.8, 138.6, 128.5, 126.8, 64.7, 37.7, –0.3; HRMS (ESI, *m/z*) calcd for $C_{22}H_{34}O_2$ $[M+Na]^+$ 265.1199, found 265.1204. Note: This product is susceptible to desilylation upon storage in solution. In HRMS only the desilylated product mass was observed.

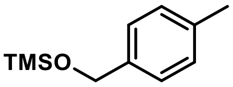

Trimethyl((4-methylbenzyl)oxy)silane (12c). Known compound.[22] The crude product was purified using silica gel column chromatography using EtOAc:heptane (2:98) as the eluent. A white powder was obtained. R_f (EtOAc:heptane, 5:95) = 0.61; ^{1}H NMR (400 MHz, $CDCl_3$) δ 7.20 (d, *J* = 8.0 Hz, 2H), 7.12 (d, *J* = 7.5 Hz, 2H), 4.64 (s, 2H), 2.32 (s, 3H), 0.13 (s, 9H).

Table S17. Synthesis of **12b** and **12c** under **Condition A**.

Entry	LnL	Condition	Light source	12b (%)	12c (%)
1	**EuL1**	**A**	Blue LED	5 (2 mg)	70 (22 mg)
2	**EuL2**	**A**	Blue LED	30 (9 mg)	58 (19 mg)

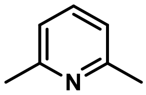

2,6-Dimethylpyridine (13c). The reaction mixture was subjected to GCMS analysis and yields were calculated from integrated area of the calibration curve prepared. MS (EI, *m/z*) calcd for C_7H_9N = 107.1 $[M]^+$, found 107.1.

Table S18. Synthesis of **13c** under **Conditions B** and **C**.

Entry	LnL	Condition	Light source	13c (%)
1	**EuL1**	**B**	365 nm	70 (GC)
2	**EuL2**	**B**	Blue LED	85 (GC)
3	**EuL1**	**B (with DMF-d_7)**	365 nm	51 (GC)
4	**EuL1**	**B (with HCO_2H-d_2)**	365 nm	44 (GC)

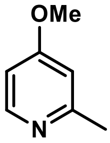

4-Methoxy-2-methylpyridine (14c). The reaction mixture was subjected to GCMS analysis and yields were calculated from integrated area of the calibration curve prepared. MS (EI, *m/z*) calcd for C_7H_9NO = 123.1 $[M]^+$, found 123.1.

Table S19. Synthesis of **14c** under **Conditions A** and **B**.

Entry	LnL	Condition	Light source	14c (%)
1	**EuL1**	**B**	365 nm	50 (GC)
2	**EuL2**	**A**	Blue LED	10 (GC)
3	**GdL1**	**B**	365 nm	0
4	**GdL2**	**A**	Blue LED	0

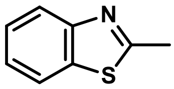

2-Methyl-1,3-benzothiazole (15c). Known compound.[23] The crude product was purified using silica gel column chromatography using EtOAc:heptane (8:92) as the eluent. A colorless oil was obtained. R_f (EtOAc:heptane, 1:9) = 0.4; ^{1}H NMR (400 MHz, $CDCl_3$) δ 7.95 (d, J = 8.0 Hz, 1H), 7.82 (d, J = 8.0 Hz, 1H), 7.46–7.42 (m, 1H), 7.34–7.32 (m, 1H), 2.84 (s, 3H).

Table S20. Synthesis of **15c** under **Conditions A** and **C**.

Entry	LnL	Condition	Light source	15c (%)
1	**EuL1**	**A**	Blue LED	58 (16 mg)
2	**EuL2**	**C**	Blue LED	67 (19 mg)

(**Note**: Reaction was followed using TLC analysis.)

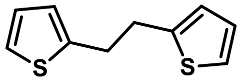

2,2'-(Ethane-1,2-diyl)bis(thiophene) (16b). Known compound.[24] The crude product was purified using silica gel column chromatography using EtOAc:heptane (1:99) as the eluent. A colorless oil was obtained. R_f (EtOAc:heptane, 1:9) = 0.7; MS (EI, *m/z*) calcd for $C_{10}H_{10}S_2$ = 194.0 $[M]^+$, 194.0. ^{1}H NMR (400 MHz, $CDCl_3$) δ 7.12 (d, J = 5.0 Hz, 2H), 6.91 (m, 2H), 6.79 (d, J = 2.5 Hz, 2H), 3.19 (s, 4H).

Table S21. Synthesis of **16b** under **Conditions A** and **C**.

Entry	LnL	Condition	Light Source	16b (%)
1	**EuL1**	**A**	Blue LED	77 (23 mg)
2	**EuL2**	**C**	Blue LED	56 (16 mg)

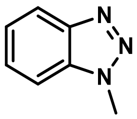

1-Methyl-1*H*-benzotriazole (17c). Known compound.[25] The crude product was purified using silica gel column chromatography using EtOAc:heptane (20:80) as the eluent. A reddish oil was obtained. R_f (EtOAc:heptane, 2:8) = 0.3; ^{1}H NMR (400 MHz, $CDCl_3$) δ 8.06 (d, *J* = 8.0 Hz, 1H), 7.53–7.48 (m, 2H), 7.39–7.35 (m, 1H), 4.30 (s, 3H); MS (EI, *m/z*) calcd for $C_{17}H_7N_3$ = 133.0 $[M]^+$, 133.0.

Table S22. Synthesis of **17c** under **Conditions A** and **C**.

Entry	LnL	Condition	Light source	17c (%)
1	**EuL1**	**A**	Blue LED	62% (13 mg)
2	**EuL2**	**A**	Blue LED	80% (17 mg)
3	**EuL2**	**C**	Blue LED	5% (1 mg)

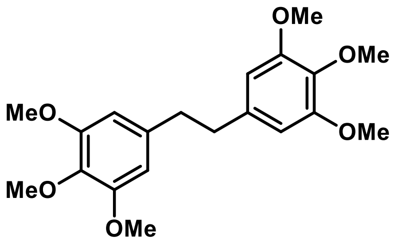

Brittonin A (18b). Known compound.[13] (807 mg, 40%). The crude product was purified using silica gel column chromatography using EtOAc:heptane (1:9) as the eluent. A pale yellow solid was obtained. R_f (EtOAc:heptane, 1:9) = 0.4; ^{1}H NMR (400 MHz, $CDCl_3$) δ 6.35 (s, 4H), 3.82 (s, 18H), 2.84 (s, 4H).

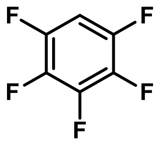

Pentafluorobenzene (19b). Known compound.[26] The reaction mixture was subjected to ^{19}F NMR analysis and yields were calculated using hexafluorobenzene as an internal standard. ^{19}F NMR (376 MHz, Benzene-d_6) δ –139.7, –156.4, –163.7.

Table S23. Synthesis of **19b** under **Conditions B** and **C**.

Entry	LnL	Condition	Light source	19b (%)
1	**EuL1**	**B**	Blue LED	60 (NMR)
2	**EuL2**	**C**	Blue LED	85 (NMR)
3	**GdL1**	**B**	Blue LED	0

4	**GdL2**	**C**	Blue LED	0

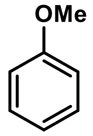

Anisole (20b). Known compound.[27] The crude product was purified using silica gel column chromatography using EtOAc:pentane (1:99) as the eluent. A colorless liquid was obtained. ^{1}H NMR (400 MHz, $CDCl_3$) δ 7.29 (dd, *J* = 8.5, 7.5 Hz, 2H), 6.93 (m, 3H), 3.81 (s, 3H); MS (EI, *m/z*) calcd for C_7H_8O = 108.0 $[M]^+$, found 108.0.

Table S24. Synthesis of **20b** under **Conditions B** and **C**.

**Entry**	**LnL**	**Condition**	**Light Source**	**20b (%)**
1	**EuL1**	**B**	Blue LED	35 (GC)
2	**EuL2**	**C**	Blue LED	30 (5 mg)

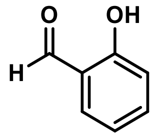

Salicylaldehyde (21b). Known compound.[28] Note: The reaction was followed using TLC analysis. The crude product was purified using silica gel column chromatography using EtOAc: pentane (20:80) as the eluent. A colorless liquid was obtained. ^{1}H NMR (400 MHz, $CDCl_3$) δ 11.01 (s, 1H), 9.89 (d, *J* = 1.0 Hz, 1H), 7.53 (m, 2H), 7.00 (m, 2H).

Table S25. Synthesis of **21b** under **Conditions A** and **C**.

**Entry**	**LnL**	**Condition**	**Light source**	**21b (%)**
1	**EuL1**	**A**	Blue LED	47 (9 mg)
2	**EuL2**	**C**	Blue LED	80 (16 mg)
3	**SmI_2**	**Stoichiometric**	None	0

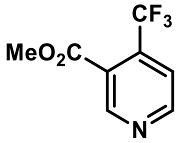

Methyl 4-trifluoromethylpyridine-3-carboxylate (22b). Known compound.[29] The crude product was purified using silica-gel column chromatography using EtOAc:heptane (20:80) as the eluent. A colorless liquid was obtained. ^{1}H NMR (400 MHz, $CDCl_3$) δ 9.10 (s, 1H), 8.91 (d, *J* = 5.0 Hz, 1H), 7.64 (d, *J* = 5.0 Hz, 1H), 3.97 (s, 3H); MS (EI, *m/z*) calcd for $C_8H_6F_3NO_2$ = 205.0 $[M]^+$, found 205.0.

Table S26. Synthesis of **22b** under **Conditions B** and **C**.

Entry	LnL	Condition	Light source	22b (%)
1	**EuL1**	**B**	365 nm	32 (11 mg)
2	**EuL2**	**C**	Blue LED	30 (10 mg)

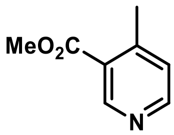

Methyl 4-methylpyridine-3-carboxylate (23b). Known compound.[30] The crude product was purified using silica gel column chromatography using EtOAc:heptane (20:80) as the eluent. A colorless liquid was obtained. ^{1}H NMR (400 MHz, $CDCl_3$) δ 9.08 (s, 1H), 8.57 (s, 1H), 7.19 (d, *J* = 5.0 Hz, 1H), 3.94 (s, 4H), 2.63 (s, 3H); MS (EI, *m/z*) calcd for $C_8H_9NO_2$ = 151.0 $[M]^+$, found 151.0.

Table S27. Synthesis of **23b** under **Conditions B** and **C**.

Entry	LnL	Condition	Light source	23b (%)
1	**EuL1**	**B**	365nm	0
2	**EuL2**	**C**	Blue LED	40 (9 mg)

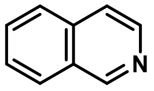

Isoquinoline (24b). Known compound.[31] The crude product was purified using silica gel column chromatography using EtOAc:pentane (12:88) as the eluent. A colorless liquid was obtained. R_f (EtOAc:heptane, 1:9) = 0.32; ^{1}H NMR (400 MHz, $CDCl_3$) δ 9.26 (s, 1H), 8.53 (d, *J* = 6.0 Hz, 1H), 7.98 (d, *J* = 8.0 Hz, 1H), 7.84–7.81 (m, 1H), 7.72–7.59 (m, 3H); MS (EI, *m/z*) calcd for C_9H_7N = 129.0 $[M]^+$, found 129.0.

Table S28. Synthesis of **24b** under **Conditions A** and **C**.

Entry	LnL	Condition	Light Source	24b (%)
1	**EuL1**	**A**	Blue LED	82 (19 mg)
2	**EuL2**	**C**	Blue LED	50 (12 mg)
3	**SmL1**	**A**	Blue LED	85 (20 mg)
4	**SmL2**	**C**	Blue LED	78 (18 mg)
5	**GdL1**	**A**	Blue LED	0
6	**GdL2**	**C**	Blue LED	0

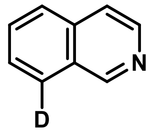

24c. The crude product was purified using silica gel column chromatography using EtOAc:pentane (12:88) as the eluent. A colorless liquid was obtained. ^{1}H NMR (400 MHz, $CDCl_3$) δ 9.28 (s, 1H), 8.54 (d, *J* = 5.8 Hz, 1H), 7.85 (d, *J* = 8.2 Hz, 1H), 7.71 (m, 2H), 7.63 (m, 1H); ^{2}H NMR (61 MHz, $CDCl_3$) δ 8.25 (s, 1H). MS (ESI, *m/z*) calcd for C_9H_6DN = 130.1 $[M+H]^+$, found 130.1.

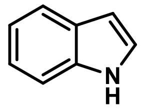

Indole (25b). Known compound.[32] The reaction mixture was purified using silica gel column chromatography using EtOAc:heptane (20:80) as the eluent. A brown solid was obtained. ^{1}H NMR (400 MHz, $CDCl_3$) δ 8.11 (s, 1H), 7.66 (m, 1H), 7.40 (m, 1H), 7.21 (m, 2H), 7.13 (m, 1H). MS (EI, *m/z*) calcd for C_8H_7N = 117.0 $[M]^+$, 117.0.

Table S29. Synthesis of **25b** under **Conditions A** and **C**.

Entry	LnL	Condition	Light Source	25b (%)
1	**EuL1**	**A**	Blue LED	0
2	**EuL2**	**C**	Blue LED	53 (10 mg)

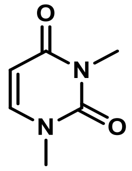

1,3-dimethyluracil (26b). Known compound.[33] The crude product was purified using silica gel column chromatography using MeOH: CH_2Cl_2 (3:97) as the eluent. A colorless solid was

obtained. R_f (EtOAc:heptane, 1:1) = 0.21; ^{1}H NMR (400 MHz, $CDCl_3$) δ 7.11 (d, *J* = 8.0 Hz, 1H), 5.73 (d, *J* = 8.0 Hz, 1H), 3.39 (s, 3H), 3.34 (s, 3H).

Table S30. Synthesis of **26b** under **Conditions B** and **C**.

Entry	LnL	Condition	Light source	26b (%)
1	**EuL1**	**B**	365 nm	79 (18 mg)
2	**EuL2**	**C**	Blue LED	67 (15 mg)

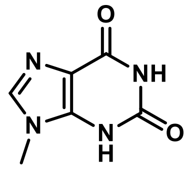

Caffeine (27b). Known compound.[34] Note: Reaction was followed using TLC analysis. The crude product was purified using silica gel column chromatography using MeOH:CH_2Cl_2 (3:97) as the eluent. A colorless solid was obtained. R_f (MeOH:CH_2Cl_2, 2:98) = 0.49; ^{1}H NMR (400 MHz, $CDCl_3$) δ 7.49 (s, 1H), 3.98 (s, 3H), 3.58 (s, 3H), 3.40 (s, 3H).

Table S31. Synthesis of **27b** under **Conditions B** and **C**.

Entry	LnL	Condition	Light source	27b (%)
1	**EuL1**	**B**	Blue LED	47 (13 mg)
2	**EuL2**	**C**	Blue LED	81 (22 mg)

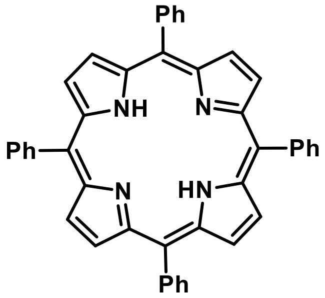

***Meso* tetraphenylporphyrin (28b).** Known compound.[35] Note: Reaction was followed using TLC analysis. The crude product was purified using silica gel column chromatography using CH_2Cl_2:pentane (5:95) as the eluent. A purple solid was obtained. ^{1}H NMR (400 MHz, $CDCl_3$) δ 8.83 (s, 8H), 8.21 (dd, *J* = 7.5, 2.0 Hz, 8H), 7.74 (m, 12H).

Table S32. Synthesis of **28b** under **Conditions A** and **C**.

Entry	LnL	Condition	Light source	28b (%)

1	**EuL1**	**A**	Blue LED	76 (8 mg)
2	**EuL2**	**C**	Blue LED	91 (9 mg)
3	**GdL1**	**A**	Blue LED	0
4	**GdL2**	**C**	Blue LED	0

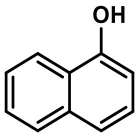

1-naphthol (29b). Known compound.[36] The crude product was purified using preparative silica-gel chromatography using EtOAc:heptane (20:80) as an eluent to obtain **29b** as a white solid (26 mg, 74%). R_f (EtOAc:heptane, 2:8) = 0.3; ^{1}H NMR (400 MHz, $CDCl_3$) δ 8.18 –8.16 (m, 1H), 7.82–7.79 (m, 1H), 7.50–7.45 (m, 2H), 7.45–7.42 (m, 1H), 7.30 (dd, *J* = 8.0, 7.5 Hz, 1H), 6.81 (dd, *J* = 7.5, 1.0 Hz, 1H), 5.19 (s, 1H).

Table S33. Synthesis of **29b**.

Entry	LnL	Condition	Time (h)	Light Source	29b (%)
1	**EuL2**	**A**	10	Blue LED	74 (18 mg)
2	**GdL2**	**A**	10	Blue LED	0

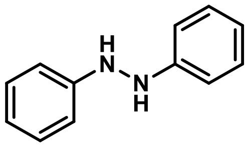

1,2-diphenylamine (30b). Known compound.[37] The crude product was purified using silica gel chromatography using EtOAc:heptane (20:80) as the eluent. A white solid was obtained. R_f (EtOAc:heptane, 2:8) = 0.52; ^{1}H NMR (400 MHz, $CDCl_3$) δ 7.23–7.18 (m, 4H), 6.87–6.80 (m, 6H), 5.63 (brs, 2H).

Table S34. Synthesis of **30b**.

Entry	LnL	Condition	Time (h)	Light Source	30b (%)
1	**EuL2**	**C**	10	Blue LED	70 (21 mg)
2	**GdL2**	**C**	10	Blue LED	0

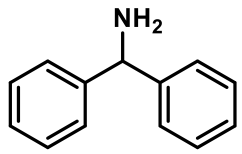

Diphenylmethanamine (31b). Known compound.[38] The crude product was purified using silica gel chromatography using EtOAc:Pentane (15:85) as the eluent. A yellow semi-solid was obtained. ^{1}H NMR (400 MHz, $CDCl_3$) δ 7.38–7.29 (m, 8H), 7.23 (t, *J* = 7.0 Hz, 2H), 5.23 (s, 1H), 2.60 (s, 2H).

Table S35. Synthesis of **31b**.

Entry	LnL	Condition	Time (h)	Light Source	31b (%)
**1**	**SmL2**	**C**	24	Blue LED	89 (27 mg)
2	**EuL2**	**C**	24	Blue LED	0
3	**GdL2**	**C**	24	Blue LED	0

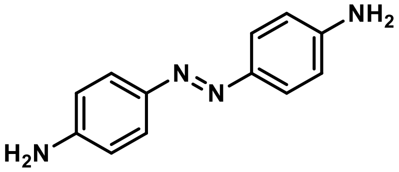

4,4'-Diaminoazobenzene (32b). Known compound.[39] The crude product was purified using silica gel chromatography using EtOAc:heptane (2:8) as the eluent. A yellow solid was obtained. R_f (EtOAc:heptane, 1:9) = 0.6; ^{1}H NMR (400 MHz, DMSO-d_6) δ 7.58 (d, *J* = 8.7 Hz, 4H), 6.75 (d, *J* = 8.6 Hz, 4H).

Table S36. Synthesis of **32b**.

Entry	LnL	Condition	Time (h)	Light Source	32b (%)
1	**EuL2**	**C**	4	Blue LED	69 (24 mg)
2	**GdL2**	**C**	4	Blue LED	0

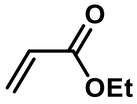

Ethylacrylate (33b). The crude product was subjected to GCMS analysis, and yield was determined based on standard calibration curve.

Table S37. Synthesis of **33b**.

Entry	LnL	Condition	Time (h)	Light Source	33b (%)
1	**EuL2**	**C**	4	Blue LED	97 (GC)
2	**GdL2**	**C**	4	Blue LED	0

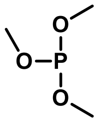

Trimethylphosphite (34b). Known compound.[40] The crude product was purified using silica gel chromatography using CH_2Cl_2:Pentane (20:80) as the eluent. A colorless liquid was obtained. ^{1}H NMR (400 MHz, Benzene-d_6) δ 3.28 (dd, J = 10.5, 1.0 Hz, 9H); ^{31}P NMR (162 MHz, Benzene-d_6) δ 140.8, MS (EI, *m/z*) calcd for $C_3H_9O_3P$ =124.0 $[M]^+$, 124.0.

Table S38. Synthesis of **34b**.

Entry	LnL	Condition	Time (h)	Light Source	34b (%)
1	**EuL2**	**A**	34	Blue LED	37 (22 mg)
2	**GdL2**	**A**	34	Blue LED	0

Table S39. Optimization studies for aldehyde or ketone reduction.

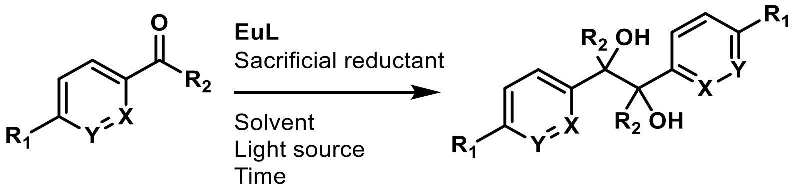

Entry	LnL	X	Y	R_1	R_2	Sacrificial reductant (equiv.)	Light source	Yield (%)
1	**EuL**	CH	CH	H	H	Condition B or C	Blue LED	0
2	**EuL2/EuL3**	CH	CH	CF_3	H	Condition B or C	Blue LED	0
3	**EuL2/EuL3**	CH	CH	OMe	H	Condition B or C	Blue LED	0
4	**EuL2/EuL3**	S	-	H	H	Condition B or C	Blue LED	0
5	**EuL2/EuL3**	O	-	H	H	Condition B or C	Blue LED	0
5	**EuL2/EuL3**	N	CH	H	H	Condition B or C	Blue LED	0
6	**EuL1/EuL2**	CH	CH	OMe	Me	DIPEA (1)	Blue LED	0
7	**EuL3**	CH	CH	OMe	Me	DIPEA:HCO_2H (1:1)	Blue LED	41 (23 mg)

8	**EuL3**	CH	CH	OMe	Me	DIPEA:HCO_2H (5:5):H_2O (10%)	Blue LED	30
9	**GdL3**	CH	CH	OMe	Me	DIPEA:HCO_2H (1:1)	Blue LED	0

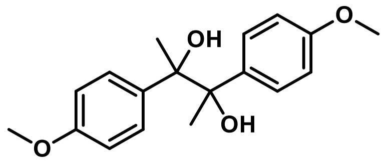

2,3-bis(4-methoxyphenyl)butane-2,3-diol (35b). Known compound.[41] The crude product was purified using silica gel column chromatography using EtOAc:heptane (18:82) as the eluent. An off-white solid was obtained. R_f (EtOAc:heptane, 2:8) = 0.3; ^{1}H NMR (400 MHz, DMSO-d_6) δ 7.30 (d, J = 8.0 Hz, 4H), 6.96 (d, J = 8.0 Hz, 4H), 6.76 (d, J = 8.0 Hz, 4H), 6.65 (d, J = 8.0 Hz, 4H), 4.82 (s, 2H), 4.71 (s, 2H), 3.72 (s,6), 3.68 (s, 6H), 1.40 (s, 6H), 1.24 (s, 6H).

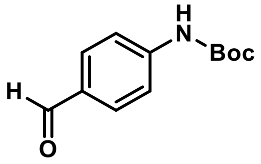

4-((tert-butoxycarbonyl)amino)benzaldehyde (36b) Known compound.[42] The product is highly susceptible to degradation in solution under air, therefore, its N-Boc protected derivative was isolated after treatment with Boc_2O (1.5 equiv.). The crude product was purified using silica gel column chromatography using EtOAc:heptane (20:80) as the eluent. A yellow solid was obtained. R_f (EtOAc:heptane, 2:8) = 0.60; ^{1}H NMR (400 MHz, $CDCl_3$) δ 9.94 (s, 1H), 7.86 (d, J = 8.5 Hz, 2H), 7.63 (d, J = 8.5 Hz, 2H), 1.54 (s, 9H).

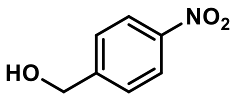

4-nitrobenzyl alcohol (36c). Known compound.[43] The crude product was purified using silica gel column chromatography using EtOAc:heptane (2:8) as the eluent. A yellow solid was obtained. R_f (EtOAc:heptane, 2:8) = 0.54; ^{1}H NMR (400 MHz, $CDCl_3$) δ 8.21 (d, J = 8.5 Hz, 2H), 7.53 (d, J = 8.5 Hz, 2H), 4.83 (s, 2H).

Table S40. Synthesis of **36b** and **36c**.

Entry	LnL	Condition	Time (h)	Light source	36b (%)	36c (%)

1	**EuL2**	DIPEA, LiCl (1:1), H_2O (10 eq)	15	BlueLED	85 (31 mg)	0
2	**EuL2**	**C**	15	BlueLED	0	32 (8 mg)
3	**GdL2**	**C**	15	BlueLED	0	0

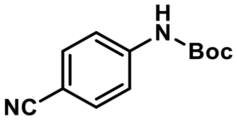

tert-butyl N-(4-cyanophenyl)carbamate (37b) Known compound.[44] N-Boc protected derivative was isolated after treatment with Boc_2O (1.5 eq). The crude product was purified using silica gel column chromatography using EtOAc:heptane (10:90) as the eluent. A colorless liquid was obtained (92%, 33 mg). R_f (EtOAc:heptane, 2:8) = 0.40; ^{1}H NMR (400 MHz, $CDCl_3$) δ 7.62 (d, J = 9.0 Hz, 2H), 7.58 (d, J = 9.0 Hz, 2H), 1.53 (s, 9H).

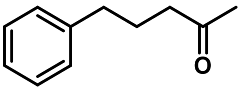

5-Phenylpentan-2-one (38b). Known compound.[45] The crude product was purified using silica gel column chromatography using EtOAc:heptane (12:88) as the eluent. An off-white solid was obtained. R_f (EtOAc:heptane, 5:95) = 0.32; ^{1}H NMR (400 MHz, $CDCl_3$) δ 7.32–7.12 (m, 5H), 2.61 (t, J = 7.5 Hz, 2H), 2.43 (t, J = 7.5 Hz, 2H), 2.12 (s, 3H), 1.96–1.83 (m, 2H); MS (EI, *m/z*) calcd for $C_{11}H_{14}O$ = 162.1 $[M]^+$, 162.1.

Table S41. Synthesis of **38b** under **Conditions A**.

Entry	LnL	Condition	Light source	38b (%)
1	**EuL1**	**A**	Blue LED	30 (8 mg)
2	**EuL2**	**A**	Blue LED	60 (16 mg)

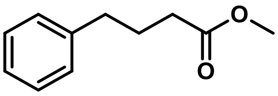

Methyl 4-phenylbutanoate (39b). Known compound.[46] The crude product was purified using silica gel column chromatography using EtOAc:heptane (12:88) as the eluent. An off-white solid was obtained. R_f (EtOAc:heptane, 5:95) = 0.4; ^{1}H NMR (400 MHz, $CDCl_3$) δ 7.30–

7.17 (m, 5H), 3.66 (s, 3H), 2.64 (t, J = 9.0 Hz, 2H), 2.32 (t, J = 7.5 Hz, 2H), 1.97–1.94 (m, 2H); MS (EI, m/z) calcd for $C_{11}H_{14}O_2$ = 178.1 $[M]^+$, found 178.1.

Table S42. Synthesis of **39b** under **Conditions A**.

Entry	LnL	Condition	Light source	39b (%)
1	**EuL1**	**A**	Blue LED	35 (10 mg)
2	**EuL2**	**A**	Blue LED	85 (25 mg)

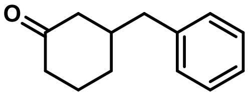

3-Benzylcyclohexan-1-one (40b). Known compound.[47] The crude product was purified using silica gel column chromatography using EtOAc:heptane (5:95) as the eluent. An off-white solid was obtained. R_f (EtOAc:heptane, 5:95) = 0.4; ^{1}H NMR (400 MHz, $CDCl_3$) δ 7.30–7.24 (m, 2H), 7.20 (d, J = 7.5 Hz, 1H), 7.13–7.06 (m, 2H), 2.61 (dd, J = 6.5, 3.5 Hz, 2H), 2.44–2.32 (m, 2H), 2.30–2.21 (m, 1H), 2.12–1.98 (m, 3H), 1.91–1.82 (m, 1H), 1.68–1.54 (m, 2H), 1.44–1.31 (m, 1H); MS (EI, m/z) calcd for $C_{13}H_{16}O$ = 188.1 $[M]^+$, 188.1.

Table S43. Synthesis of **40b** under **Conditions A**.

Entry	LnL	Condition	Light Source	40b (%)
1	**EuL1**	**A**	Blue LED	48 (15 mg)
2	**EuL2**	**A**	Blue LED	30 (9 mg)

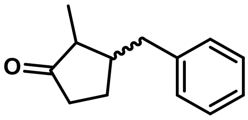

3-Benzyl-2-methylcyclopentan-1-one (41b). The crude product was purified using silica gel column chromatography using EtOAc:heptane (5:95) as the eluent. A pale yellow oil was obtained. R_f (EtOAc:heptane, 5:95) = 0.4; ^{1}H NMR (400 MHz, $CDCl_3$) δ 7.34–7.11 (m, 5H), 3.00 (dd, J = 13.5, 4.5 Hz, 1H), 2.44–2.18 (m, 1H), 2.43–2.14 (m, 1H), 2.13–1.63 (m, 4H), 1.54–1.37 (m, 1H), 1.07 (d, J = 7.0 Hz, 3H); ^{13}C NMR (101 MHz, $CDCl_3$) δ 173.7, 136.7, 129.2, 128.7, 128.5, 126.3, 96.7, 50.0, 46.6, 40.5, 37.3, 29.8, 12.8. HRMS (EI, m/z) calcd for $C_{22}H_{34}O_2$ 188.1196 $[M]^+$, found 188.1201. GC-HRMS and ^{1}H NMR shows **41b** as mixture of two diastereomers. In the case of the stoichiometric reaction of **41a**, **1a** with SmI_2, GCMS analysis shows complete disappearance of **41a** within 30 mins. No **41b** formation was observed, and **1a** remained intact (see GCMS traces below).

Table S44. Synthesis of **41b** under **Conditions A** and stoichiometric SmI_2.

Entry	LnL	Condition	Light Source	41b (%)
1	**EuL1**	**A**	Blue LED	41 (13 mg)
2	**EuL2**	**A**	Blue LED	80 (25 mg)
3	**SmI_2**	**Stoichiometric**	None	0

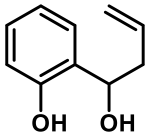

2-(1-Hydroxybut-3-en-1-yl)phenol (42c). Known compound.[48,49] The crude product was purified using preparative silica gel chromatography using EtOAc:heptane (10:90) as the eluent. A colorless solid was obtained (95%, 7.7 mg). R_f (EtOAc:heptane, 1:1) = 0.4; ^{1}H NMR (400 MHz, $CDCl_3$) δ 7.18 (m, 1H), 6.98 (dd, *J* = 7.5, 1.5 Hz, 1H), 6.90–6.82 (m, 2H), 5.91–5.81 (m, 1H), 5.26–5.22 (m, 2H), 4.89 (dd, *J* = 8.5, 5.0 Hz, 1H), 2.65–2.60 (m, 3H).

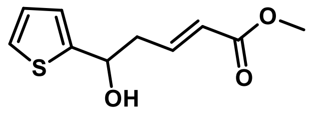

5-hydroxy-5-[2]thienyl-pent-2t-enoic acid methyl ester (43c). The crude product was purified using preparative silica gel chromatography using EtOAc:heptane (1:9) as the eluent. A colorless oil was obtained (30%, 11 mg). R_f (EtOAc:heptane, 2:8) = 0.5; ^{1}H NMR (400 MHz, $CDCl_3$) δ 6.96 (m, 3H), 5.93 (d, *J* = 15.0 Hz, 1H), 5.08 (t, *J* = 6.0 Hz, 1H), 3.72 (s, 3H), 2.78–2.75 (m, 2H); ^{13}C NMR (101 MHz, $CDCl_3$) δ 166.4, 146.9, 143.9, 126.6, 124.9, 123.9, 123.8, 77.1, 76.8, 76.5, 68.8, 51.4, 41.7. HRMS (ESI, *m/z*) calcd for $C_{10}H_{12}O_3SNa$ [M+Na]$^+$ 235.0399, found 235.0399.

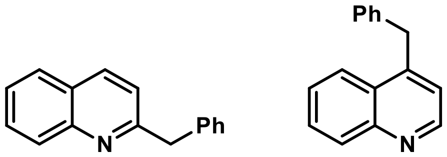

44b, 44c. Known compounds.[49] The crude product was purified using silica gel column chromatography using EtOAc:heptane (8:92→15:85) as the eluent.

2-Benzylquinoline (44b). Yellow solid (45%, 16 mg). ^{1}H NMR (400 MHz, $CDCl_3$) δ 8.08 (d, *J* = 8.5 Hz, 1H), 8.01 (d, *J* = 8.5 Hz, 1H), 7.76–7.74 (m, 1H), 7.69–7.67 (m, 1H), 7.51–7.46 (m, 1H), 7.27–7.20 (m, 5H), 4.34 (s, 2H).

4-Benzylquinoline (44c). Yellow solid (22%, 8 mg). ^{1}H NMR (400 MHz, $CDCl_3$) δ 8.81 (d, *J* = 4.5 Hz, 1H), 8.13 (dd, *J* = 8.5, 1.5 Hz, 1H), 8.03 (dd, *J* = 8.5, 1.5 Hz, 1H), 7.70 (t, *J* = 8.5 Hz, 1H), 7.53 (t, *J* = 8.5 Hz, 1H), 7.32–7.28 (m, 2H), 7.25–7.20 (m, 3H), 7.13 (dd, *J* = 4.5, 1.0 Hz, 1H), 4.44 (s, 2H).

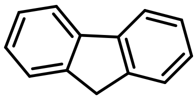

9H-Fluorene (45b). Known compound.[50] The crude product was purified using preparative silica gel thin layer chromatography using EtOAc:heptane (10:90) as the eluent. Colorless solid (22%, 6 mg). ^{1}H NMR (400 MHz, $CDCl_3$) δ 7.80 (d, *J* = 7.5 Hz, 2H), 7.56 (d, *J* = 7.5 Hz, 2H), 7.39 (m, 2H), 7.31 (td, *J* = 7.5, 1.0 Hz, 2H), 3.91 (s, 2H).

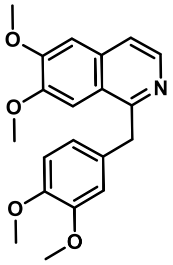

Papaverine (46c). Known compound.[51] (3 mg, 56%). The crude product was purified using silica gel column chromatography using EtOAc:heptane (50:50) as the eluent. A yellow solid was obtained. ^{1}H NMR (400 MHz, $CDCl_3$) δ 8.32 (t, *J* = 5.5 Hz, 1H), 7.81 (d, *J* = 6.0 Hz, 1H), 7.58 (s, 1H), 7.22 (s, 1H), 6.90 (d, *J* = 8.0 Hz, 1H), 6.76 (d, *J* = 8.0 Hz, 1H), 4.96 (s, 2H), 4.10 (d, *J* = 1.5 Hz, 3H), 4.01 (d, *J* = 1.5 Hz, 3H), 3.87 (d, *J* = 1.5 Hz, 3H), 3.81 (d, *J* = 1.5 Hz, 3H).

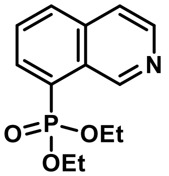

Diethyl isoquinolin-8-ylphosphonate (47b). The crude product was purified using silica gel column chromatography using MeOH:CH_2Cl_2 (7:93) as the eluent. A yellowish liquid was obtained. ^{1}H NMR (400 MHz, $CDCl_3$) δ 9.99 (s, 1H), 8.69 (d, *J* = 6.0 Hz, 1H), 8.35 (dd, *J* = 16.0, 7.0 Hz, 1H), 8.03 (d, *J* = 8.0 Hz, 1H), 7.80 (td, *J* = 6.0, 4.0 Hz, 2H), 4.31–4.25 (m, 2H), 4.17–4.11 (m, 2H), 1.32 (t, *J* = 7.0 Hz, 6H); ^{13}C NMR (101 MHz, $CDCl_3$) δ 151.4, 142.2, 136.5, 135.9, 131.6, 129.9, 127.5, 125.7, 121.7, 76.8, 62.7, 16.2; ^{31}P NMR (162 MHz, $CDCl_3$) δ 16.6. MS (ESI, *m/z*) calcd for $C_{13}H_{16}NO_3P$ = 266.2 $[M+H]^+$, found 266.2.

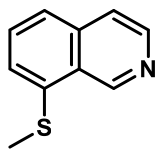

8-(Methylsulfanyl)isoquinoline (47c). The crude product was purified using silica gel column chromatography using EtOAc:pentane (35:65) as the eluent. A yellowish liquid was obtained. ^{1}H NMR (400 MHz, DMSO-d_6) δ 9.45 (s, 1H), 8.52 (d, J = 5.5 Hz, 1H), 7.81 (d, J = 5.5 Hz, 1H), 7.73–7.67 (m, 2H), 7.50 (d, J = 6.0 Hz, 1H), 2.61 (s, 3H); ^{13}C NMR (101 MHz, DMSO-d_6) δ 148.3, 144.0, 137.4, 136.4, 131.2, 126.1, 124.1, 123.9, 121.4, 15.0. MS (ESI, *m/z*) calcd for $C_{10}H_9NS$ = 176.2 $[M+H]^+$, found 176.2.

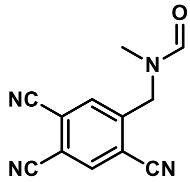

***N*-methyl-*N*-(2,4,5-tricyanobenzyl)formamide (48b).** The crude product was purified using silica gel chromatography using EtOAc:Pentane (24:76) as the eluent. A colorless solid was obtained. ^{1}H NMR (400 MHz, $CDCl_3$) δ 8.23 (s, 1H), 8.08 (s, 1H), 7.87 (s, 1H), 4.78 (s, 2H), 3.08 (s, 3H); ^{13}C NMR (101 MHz, $CDCl_3$) δ 163.3, 146.4, 137.0, 134.0, 120.1, 117.0, 116.1, 114.1, 113.9, 113.4, 77.4, 77.1, 76.8, 46.5, 35.4; MS (ESI, *m/z*) calcd for $C_{12}H_8O_4N$ = 225.2 $[M+H]^+$, 225.2. Note: The ^{1}H NMR spectrum shows the presence of trace amounts of an inseparable rotamer, in accord with the literature report[52] of a similar transformation.

Table S45. Synthesis of **48b**.

Entry	LnL	Condition	Time (h)	Light Source	48b (%)
1	**EuL2**	**C**	24	Blue LED	65 (2 mg)
2	**GdL2**	**C**	24	Blue LED	0
3	**EuL1** (1 equiv)	-	48	Blue LED	94 (4 mg)

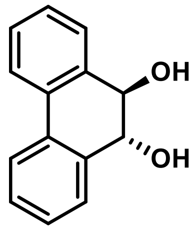

2,3-bis(4-methoxyphenyl)butane-2,3-diol (49b). Known compound.[53] The crude product was purified using silica gel column chromatography using EtOAc:heptane (18:82) as the eluent. An off-white solid was obtained. ^{1}H NMR (400 MHz, $CDCl_3$) δ 7.75–7.74 (m, 2H), 7.68–7.66 (m, 2H), 7.43–7.36 (m, 4H), 4.76 (s, 2H), 2.09 (s, 3H).

Table S46. Synthesis of **49b**.

Entry	LnL	Condition	Time (h)	Light Source	49b (%)
**1**	**SmL2**	**C**	24	Blue LED	74 (5 mg)
2	**EuL2**	**C**	24	Blue LED	41 (3 mg)
3	**GdL2**	**C**	24	Blue LED	0

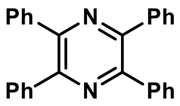

2,3,5,6-tetraphenyl-1,4-diazine (50b). Known compound.[54] The crude product was purified using silica gel chromatography using EtOAc:Pentane (2:88) as the eluent. A white solid was obtained. ^{1}H NMR (400 MHz, $CDCl_3$) δ 7.65–7.63 (m, 8H), 7.34–7.32 (m, 12H); MS (ESI, *m/z*) calcd for $C_{28}H_{21}N_2$ = 385.4 $[M+H]^+$, 385.4.

Table S47. Synthesis of **50b**.

Entry	LnL	Condition	Time (h)	Light Source	50b (%)
**1**	**SmL2**	**C**	24	Blue LED	39 (3 mg)
2	**EuL2**	**C**	24	Blue LED	10
3	**GdL2**	**C**	24	Blue LED	0

Limitations of the catalytic procedure

Figure S7. Attempted aromatic dehalogenations.

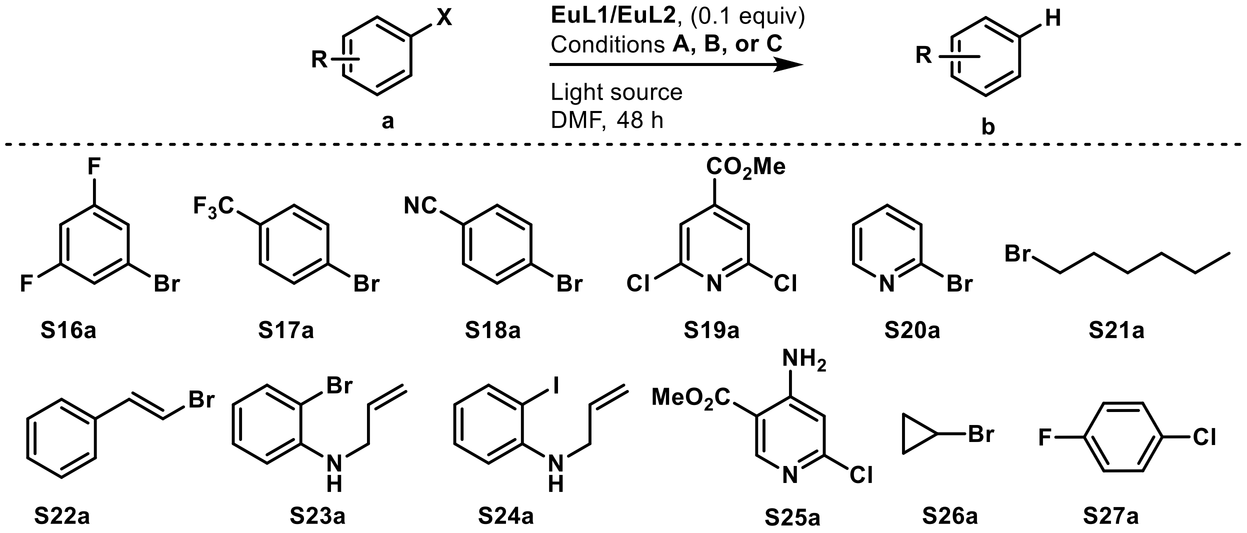

Table S48. Attempted cross-coupling reactions.

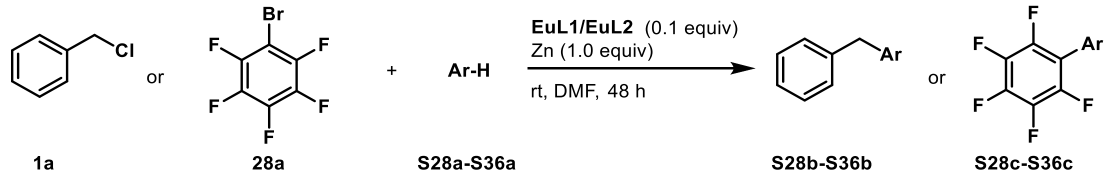

Entry	Ar-H		Light Source	Yield (%)
1	N-methylpyrrole	**1a**	Blue LED	0
2	N-methylpyrazole	**1a**	Blue LED	0
3	1,3-dimethoxybenzene	**1a**	Blue LED	0
4	Ph_2IBF_4	**1a**	Blue LED	<10%
5	**25b**	**1a**	Blue LED	0
5	Benzimidazole	**1a**	Blue LED	0
6	Benzene	**1a**	Blue LED	0
7	**19a**	**1a**	Blue LED	0
8	**25a**	**1a**	Blue LED	0

Table S49. Attempted addition of benzyl chloride to alkyne substrates.

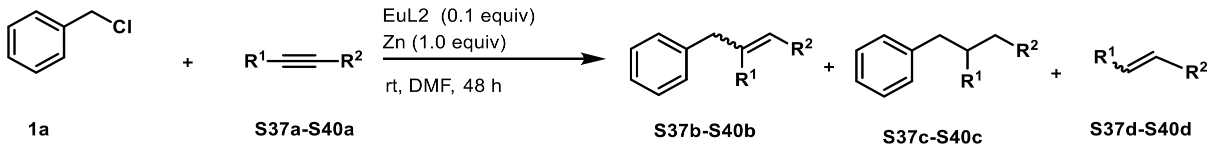

Entry	R[1]	R^2	Light Source	S28b–S31b (%)	S28c–S31c (%)	S28d–S31d (%)
1	Ph	Ph	Blue LED	0	0	10 (cis:trans)
2	Pyridyl	H	Blue LED	0	0	0
3	-CO_2Et	H	Blue LED	0	10	0
4	Ph	H	Blue LED	0	0	0

Table S50. Attempted addition of benzyl chloride to carbonyl substrates.

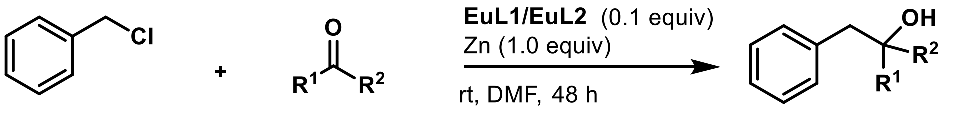

1a S41a-S45a S41b-S45b

Entry	R^1	R^2	Light source	S32b–S36b (%)
1	Ph	Ph	Blue LED	0
2	OMePh	CH_3	Blue LED	0
3	Ph	H	Blue LED	0
4	Pyridyl	H	Blue LED	0
5	C_6F_5	C_6F_5	Blue LED	0

Table S51. Attempted dehalogenation of benzoyl chlorides.

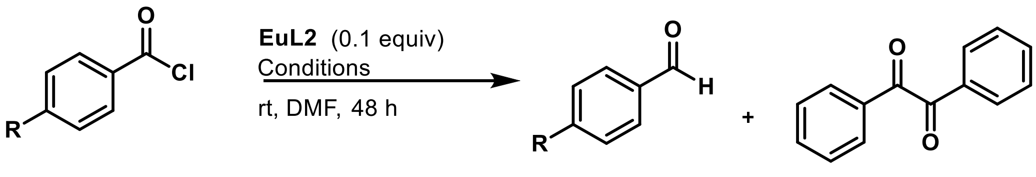

S46a-S49a S46b-S9b S46c-S49c

Entry	R	Light Source	Yield (%)
1	H	Blue LED	0
2	NO_2	Blue LED	0
3	CN	Blue LED	0
4	Pyrdiyl	Blue LED	0

Photophysical characterization

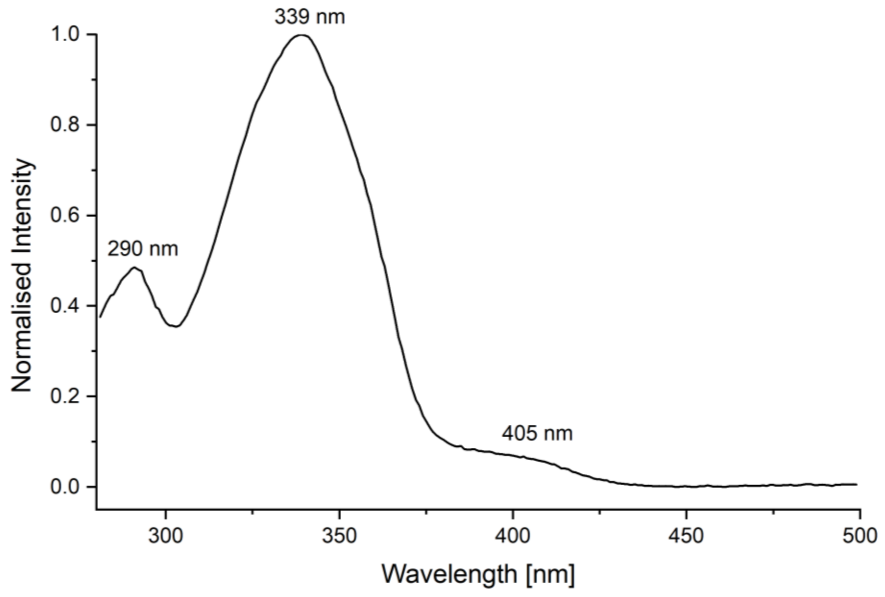

Figure S8. Normalized UV absorption spectrum of **EuL1** (16.7 µM) in DMF. Black numbers indicate the local maxima of the spectra.

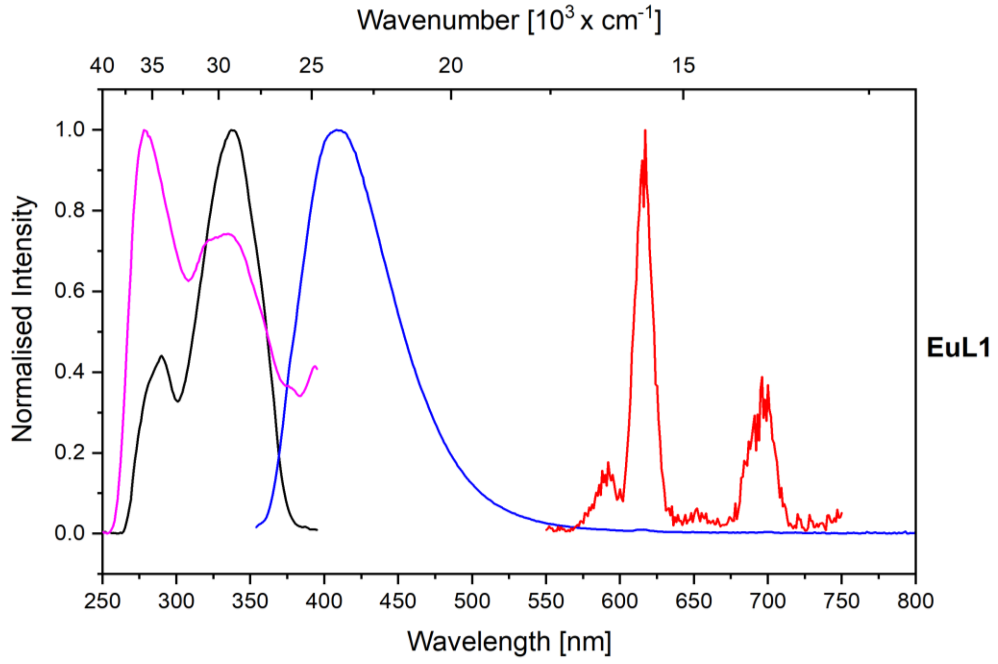

Figure S9. The excitation spectra of ligand fluorescence and **EuL1** emission (black and magenta lines, left, λ_{em} = 412 nm and 616 nm, respectively), steady-state emission spectra of **EuL1** at r.t. (blue lines, right, λ_{ex} = 339 nm). [**EuL1**] = 16.7 µM in DMF and time-resolved emission spectra of **EuL1** (red line, λ_{exc} = 339 nm), entry and exit slit width 15 nm, 15 nm; [**EuL1**] = 26.6 µM in DMF.

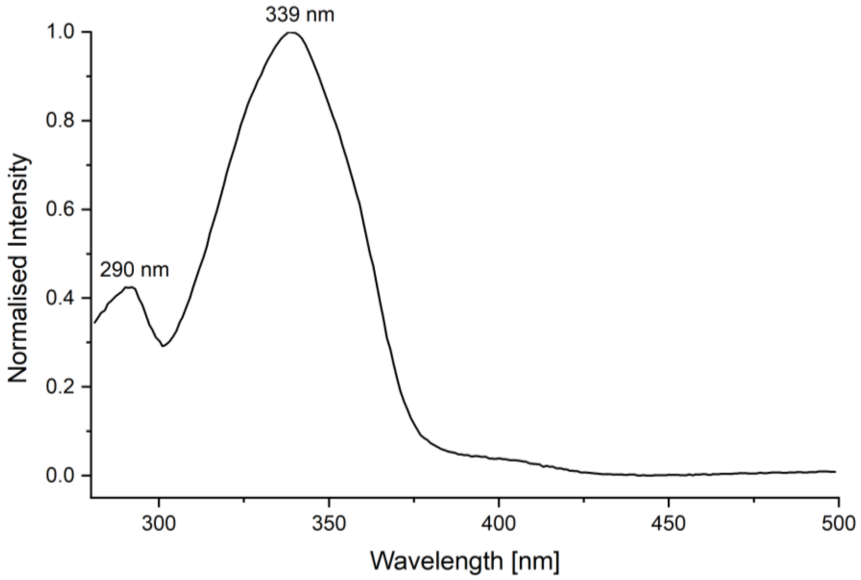

Figure S10. Normalized UV absorption spectrum of **SmL1** (13.6 µM) in DMF. Black numbers indicate the local maxima of the spectra.

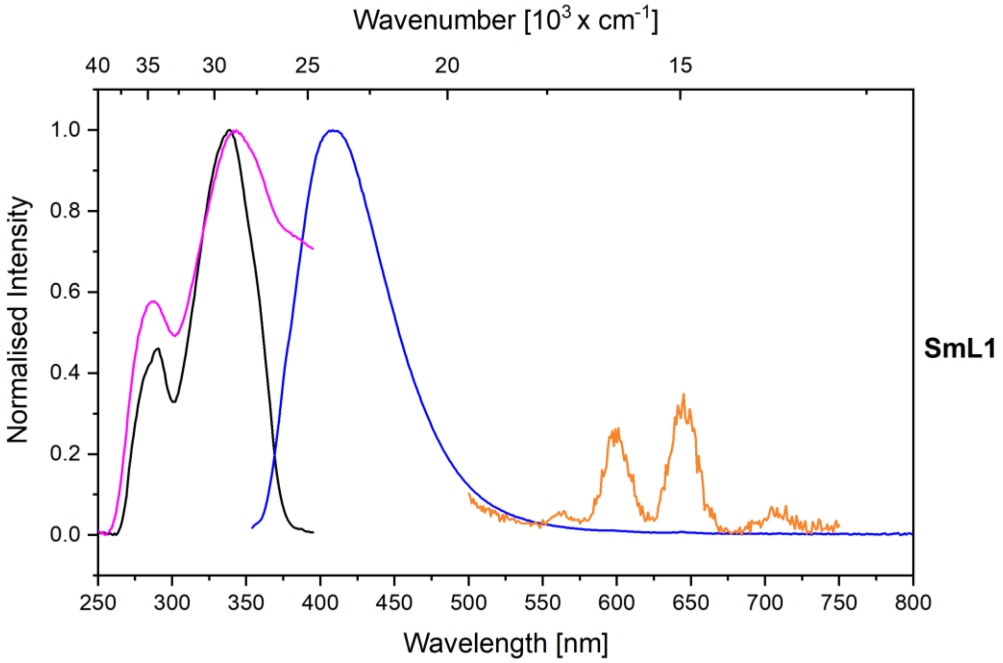

Figure S11. The excitation spectra of ligand fluorescence and **SmL1** emission (black and magenta lines, left, λ_{em} = 412 nm and 601 nm, respectively) and steady-state emission spectra of **SmL1** at r.t. (blue lines, right, λ_{ex} = 339 nm). [**SmL1**] = 13.6 µM and time-resolved emission of **SmL1** (orange line, λ_{exc} = 339 nm), entry and exit slit width 15 nm, 15 nm, [**SmL1**] =13.6 µM in DMF.

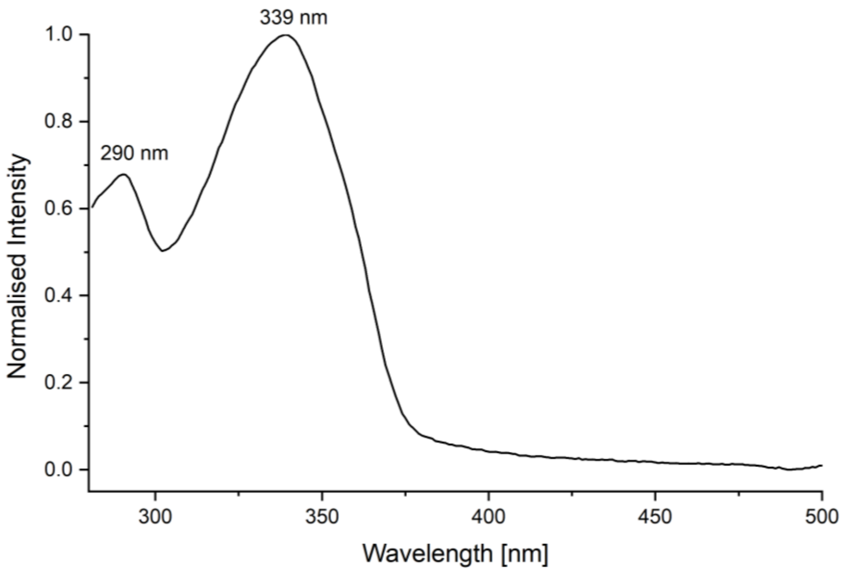

Figure S12. Normalized UV absorption spectrum of **DyL1** (10 µM) in DMF. Black numbers indicate the local maxima of the spectra.

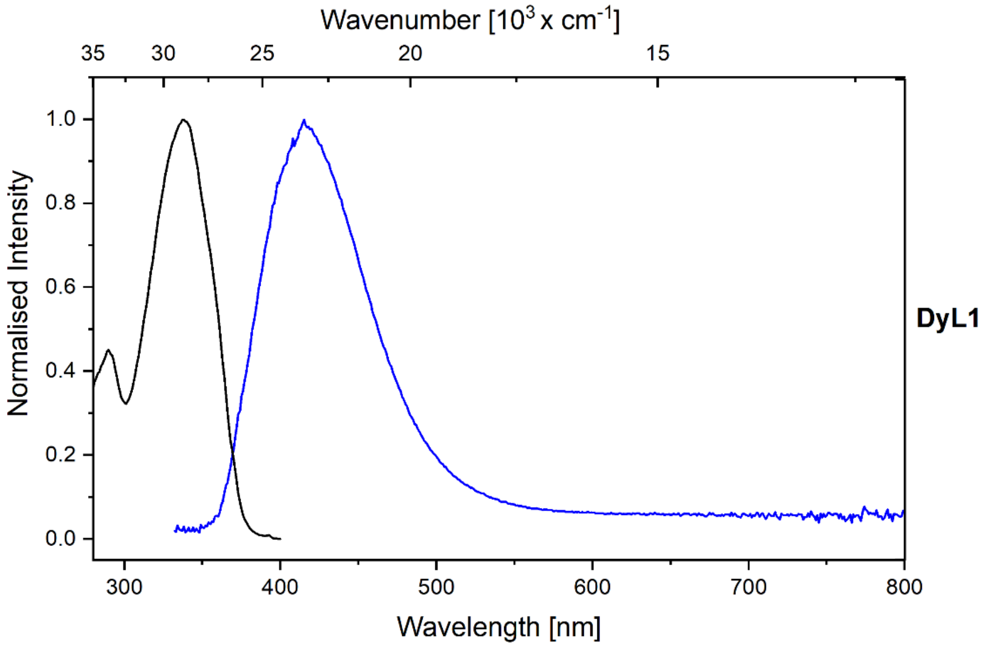

Figure S13. The excitation spectra of ligand fluorescence and **DyL1** emission (black, left, λ_{em} = 416 nm) and steady-state emission spectra of **DyL1** at r.t. (blue lines, right, λ_{ex} = 330 nm). [**DyL1**] = 10 µM in DMF.

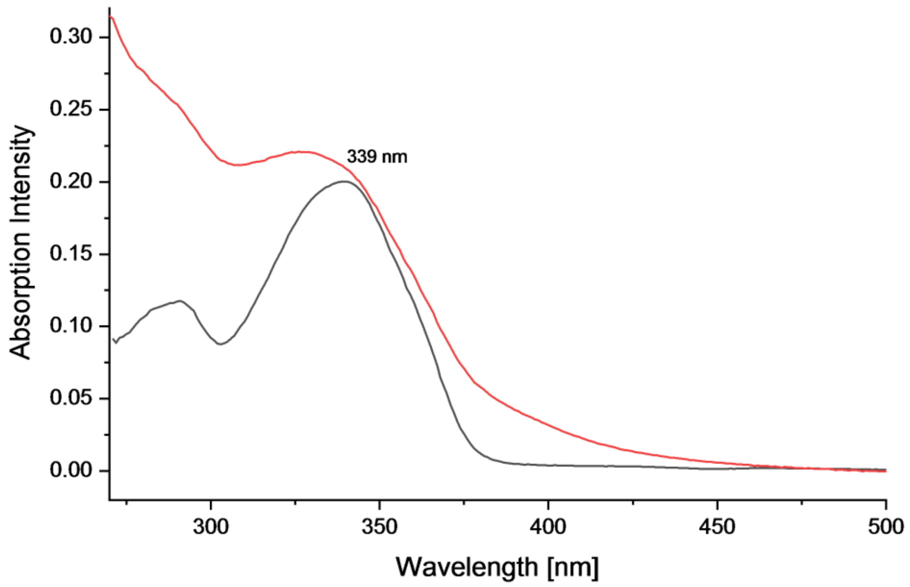

Figure S14. Normalized UV absorption spectrum of **EuL2** (58 µM, red line) and **GdL2** (210 µM, black line) in DMF. Black number indicates the local maximum of the **GdL2** spectrum.

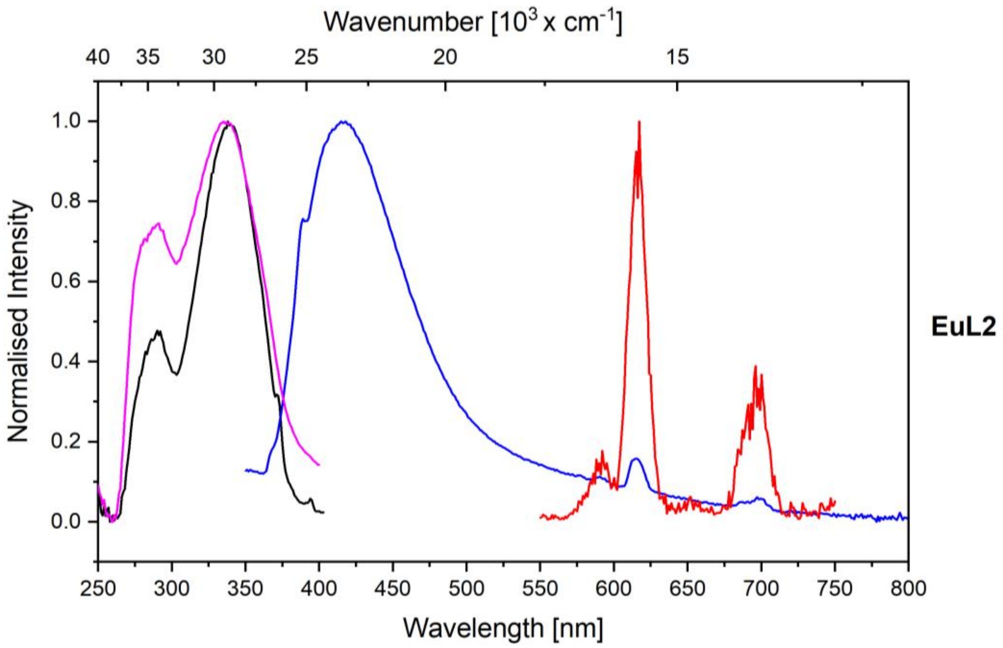

Figure S15. The excitation spectra of ligand fluorescence and **EuL2** emission (black and magenta lines, left, λ_{em} = 418 nm and 616 nm, respectively), steady-state emission spectra of **EuL2** at r.t. (blue lines, right, λ_{ex} = 348 nm). [**EuL2**] = 58 µM in DMF and time-resolved emission spectra of **EuL2** (red line, λ_{exc} = 348 nm) in DMF, entry and exit slit width 15 nm, 15 nm; [**EuL2**] = 100 µM in DMF.

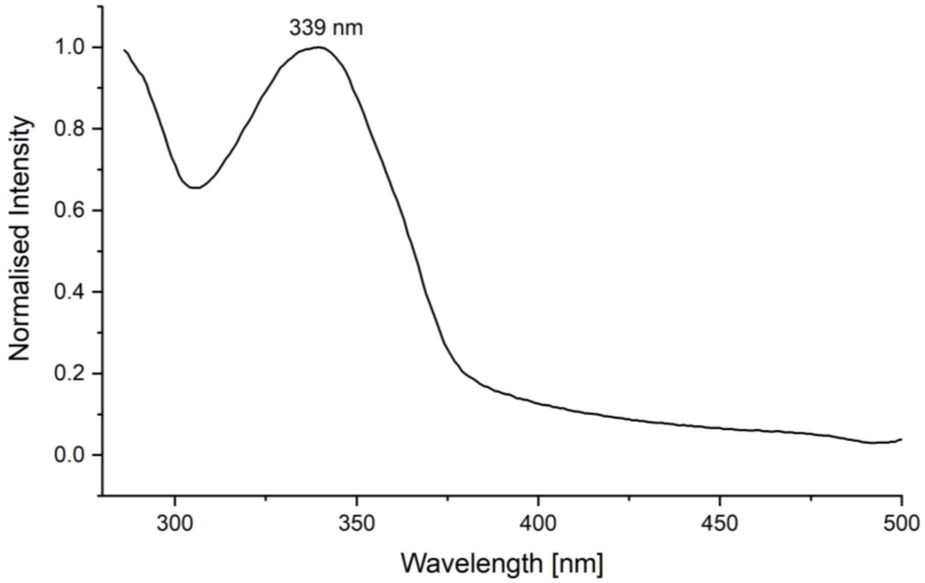

Figure S16. Normalized UV absorption spectrum of **SmL2** (17 µM) in DMF. Black numbers indicate the local maxima of the spectra.

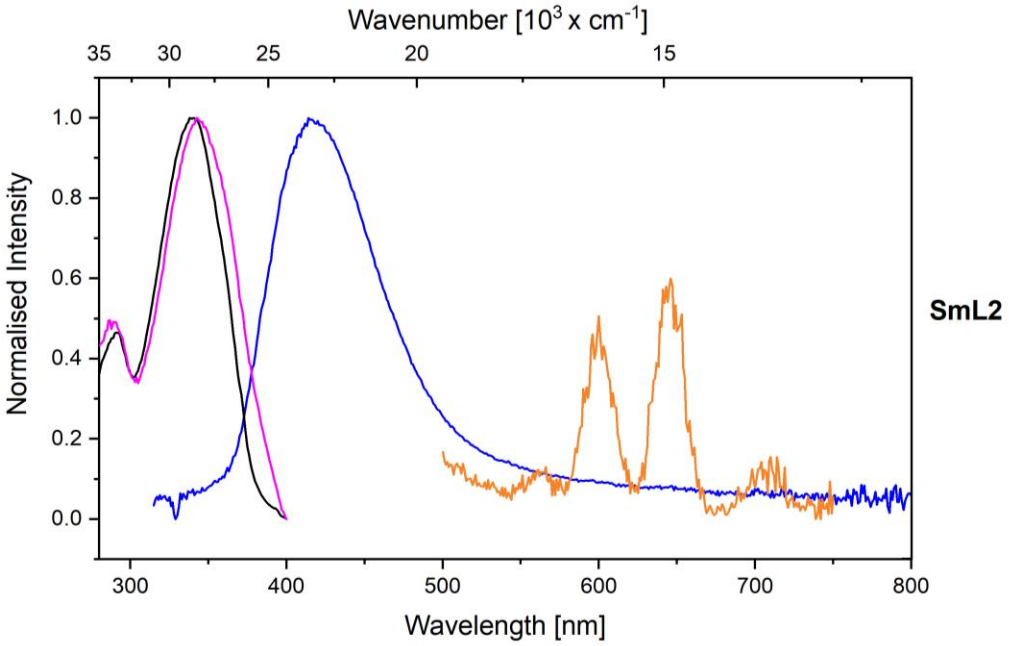

Figure S17. The excitation spectra of ligand fluorescence and **SmL2** emission (black and magenta lines, left, λ_{em} = 417 nm and 601 nm, respectively) and steady-state emission spectra of **SmL2** at r.t. (blue lines, right, λ_{ex} = 328 nm), [**SmL2**] = 17 µM in DMF and time-resolved emission of **SmL2** (orange line, λ_{exc} = 339 nm), entry and exit slit width 15 nm, 15 nm, [**SmL2**] = 51 µM in DMF.

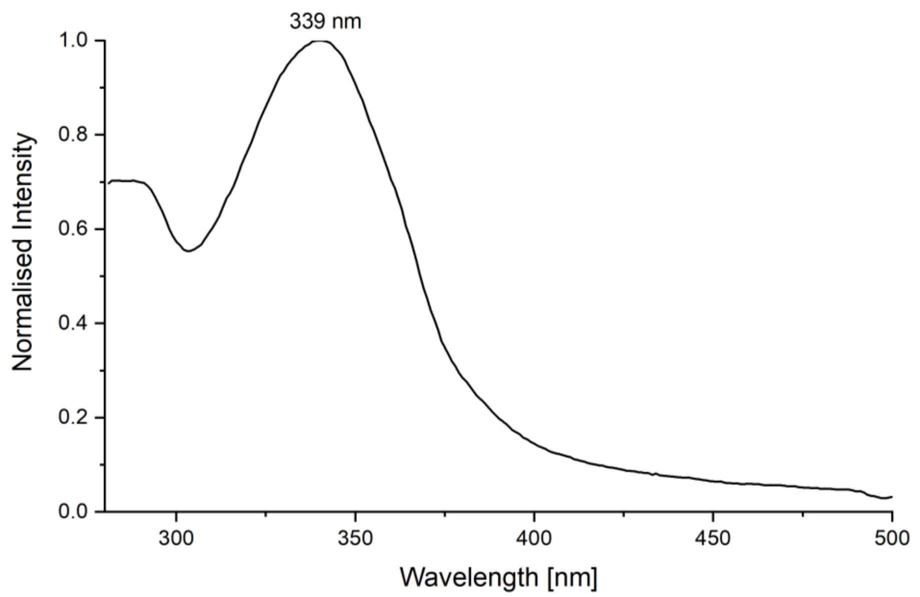

Figure S18. Normalized UV absorption spectrum of **DyL2** (20 µM) in DMF. Black numbers indicate the local maxima of the spectra.

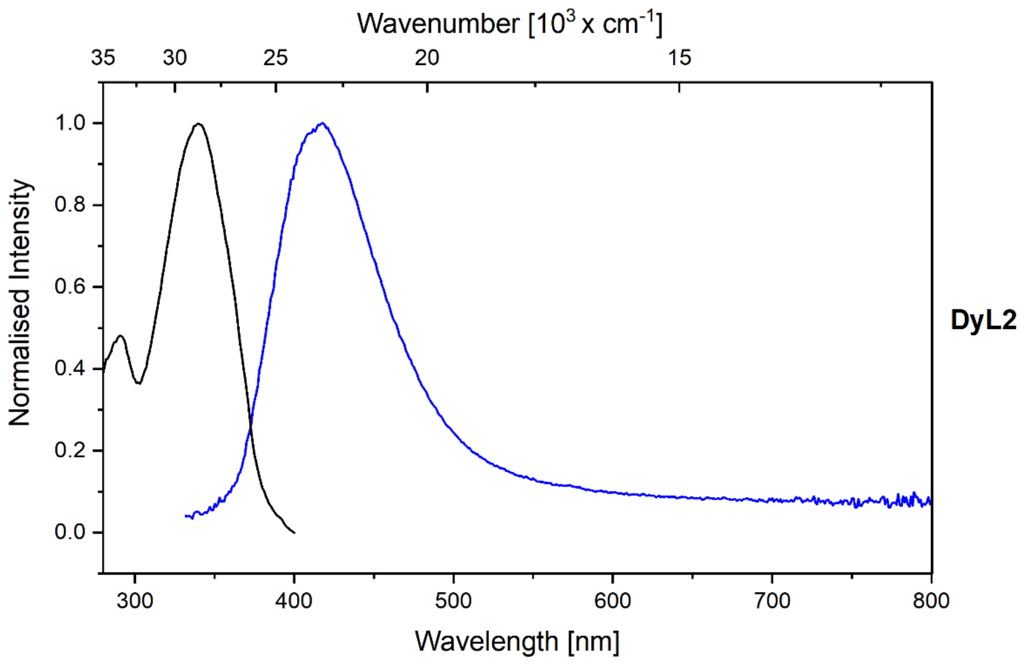

Figure S19. The excitation spectra of ligand fluorescence and **DyL2** emission (black, left, λ_{em} = 415 nm) and steady-state emission spectra of **DyL2** at r.t. (blue lines, right, λ_{ex} = 328 nm). [**DyL2**] = 20 µM in DMF.

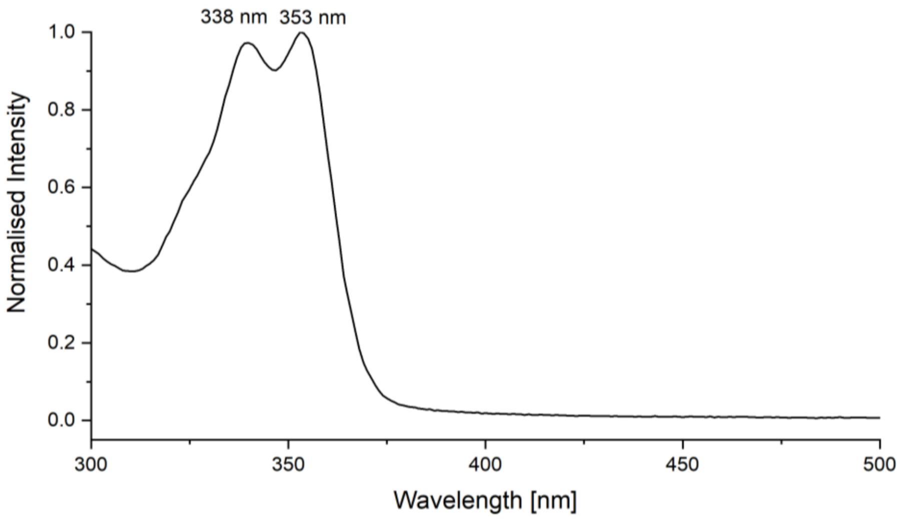

Figure S20. Normalized UV absorption spectrum of **EuL3** (5 µM) in DMF. Black numbers indicate the local maxima of the spectra.

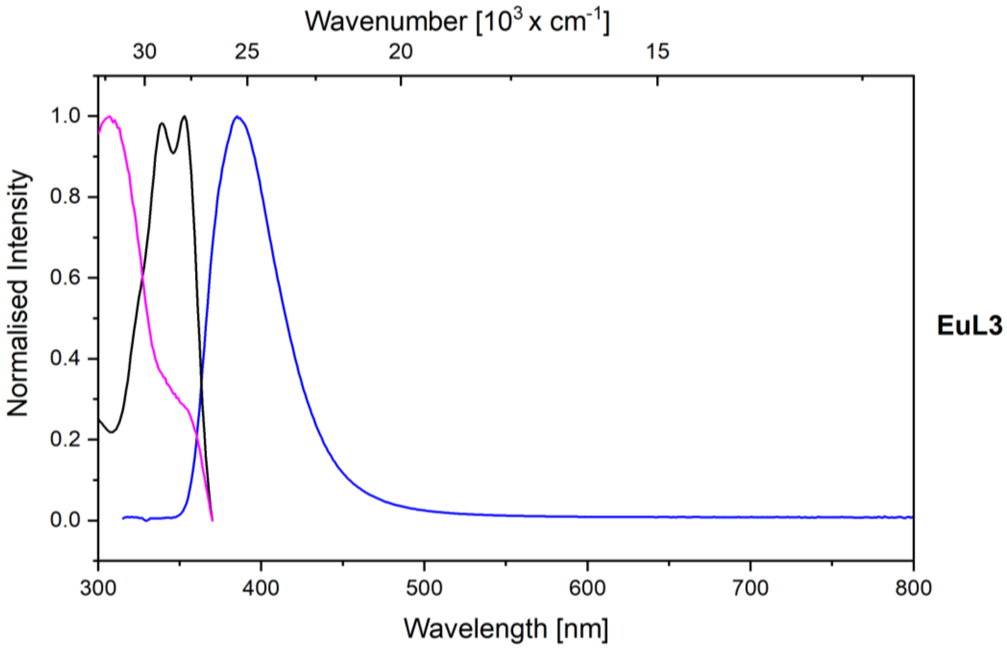

Figure S21. The excitation spectra of ligand fluorescence and **EuL3** emission (black and magenta lines, left, λ_{em} = 385 nm and 616 nm, respectively) and steady-state emission spectra of **EuL3** at r.t. (blue lines, right, λ_{ex} = 346 nm). [**EuL3**] = 15 µM in DMF.

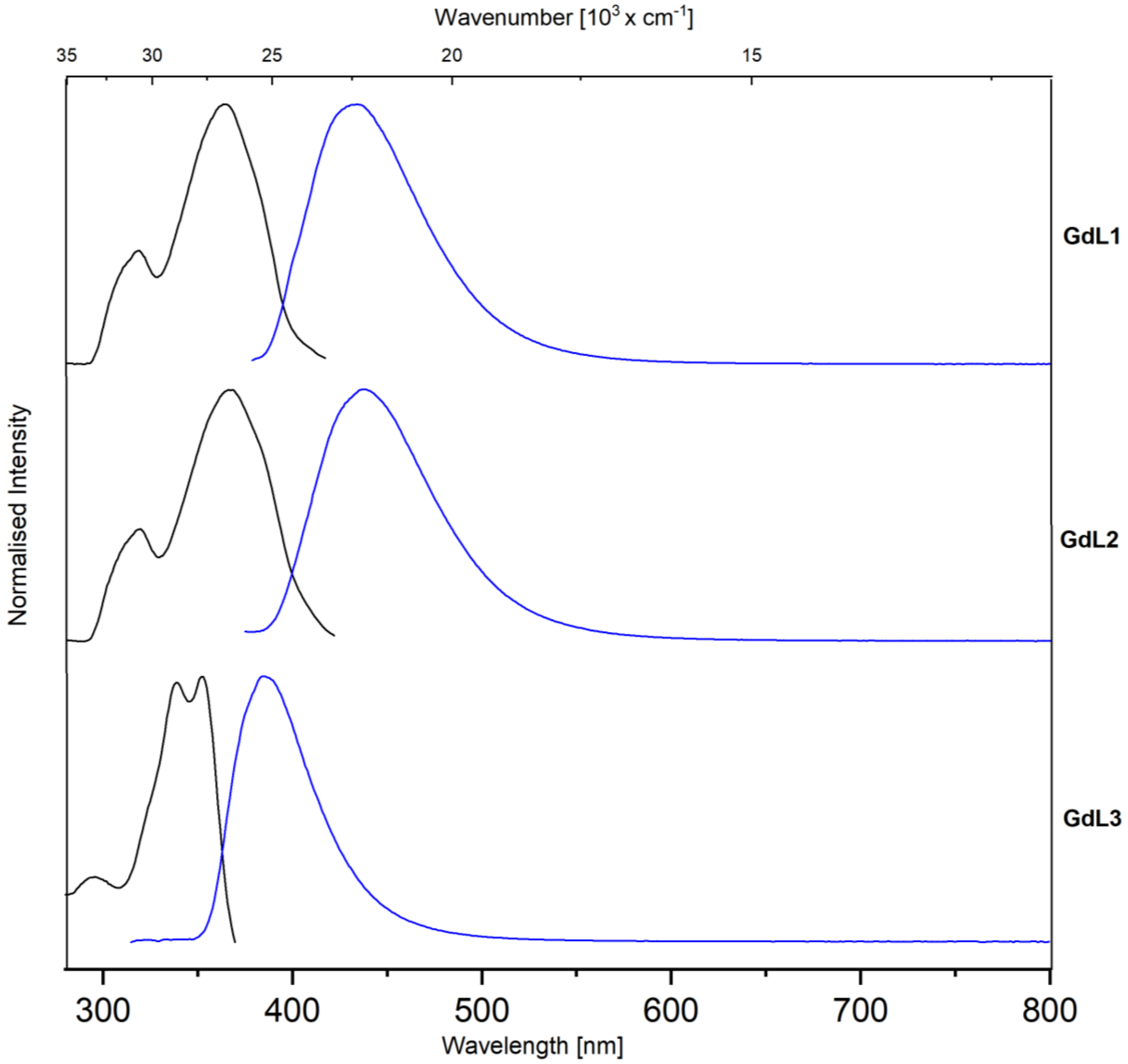

Figure S22. Ligand fluorescence of excitation (black, left, λ_{em} = 415 nm for **GdL1** and **GdL2**, 385 nm for **GdL3**) and steady-state emission spectra (blue lines, right, λ_{ex} = 339 nm for **GdL1**, 340 nm for **GdL2**, 346 nm for **GdL3**) of **GdL(L= L1, L2, L3)** in DMF [**GdL1**] = 14.1 µM, [**GdL2**] = 210 µM and [**GdL3**] = 13 µM

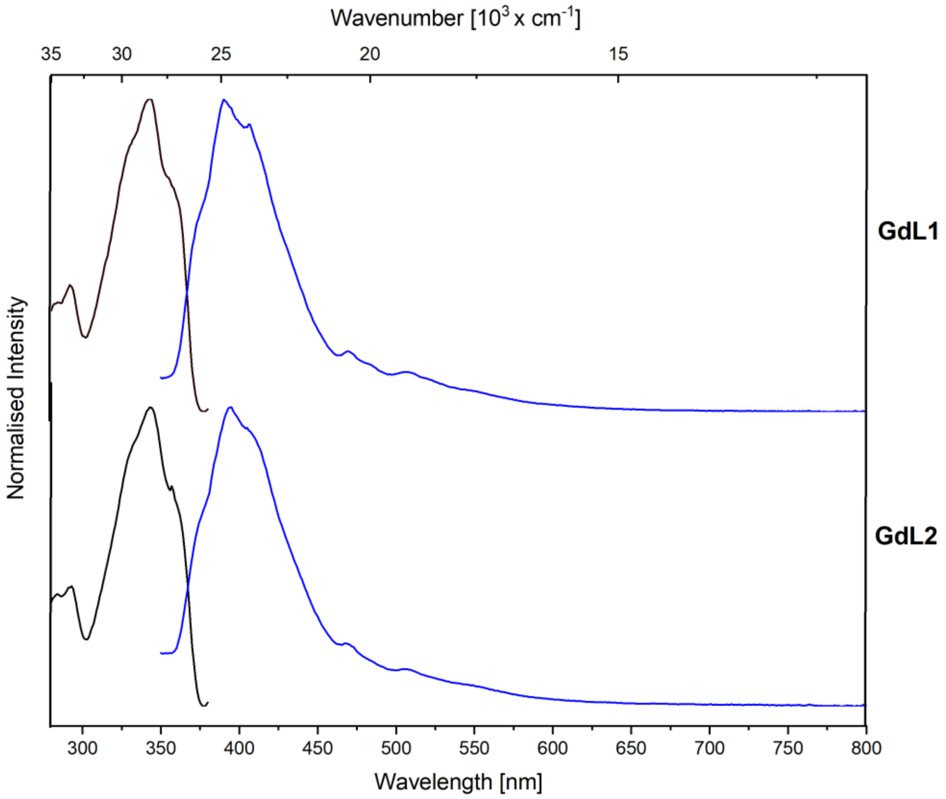

Figure S23. Ligand phosphorescence excitation (black, left) and steady-state emission spectra (blue, right) of **GdL1** and **GdL2** at 77 K with 10% glycerol added to DMF. [**GdL1**] = 14.1 µM, λ_{ex} = 339; λ_{em} = 390 nm [**GdL2**] = 210 µM, λ_{ex} = 340; λ_{em} = 394 nm.

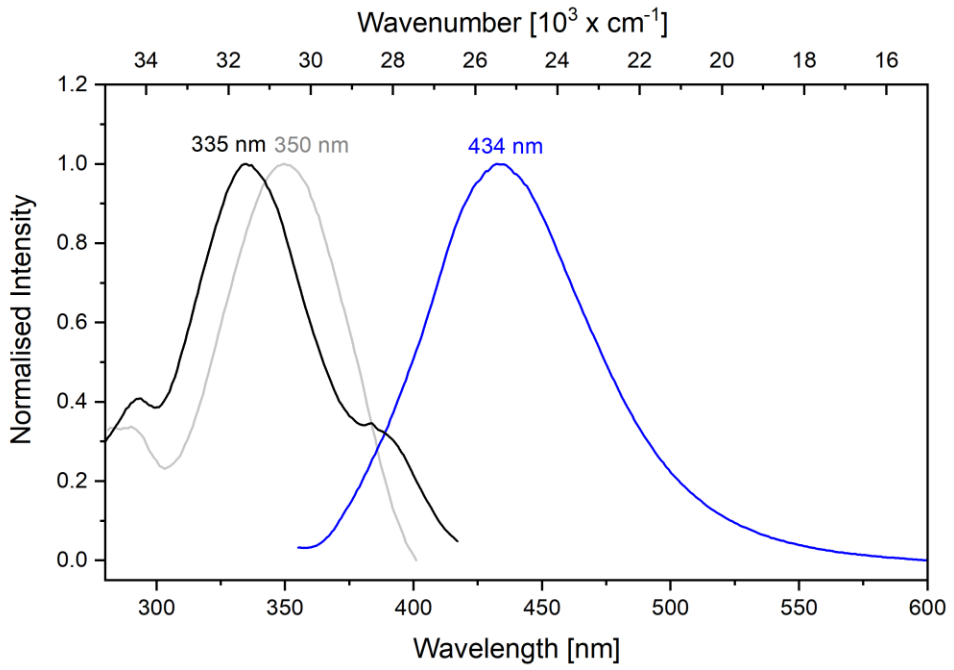

Figure S24. The absorption, excitation (black and blue lines, left, λ_{em} = 432 nm) and steady-state emission spectra (red lines, right, λ_{ex} = 350 nm) of **Eu(II)L1** in DMF. [**Eu(II)L1**] = 20 µM in DMF.

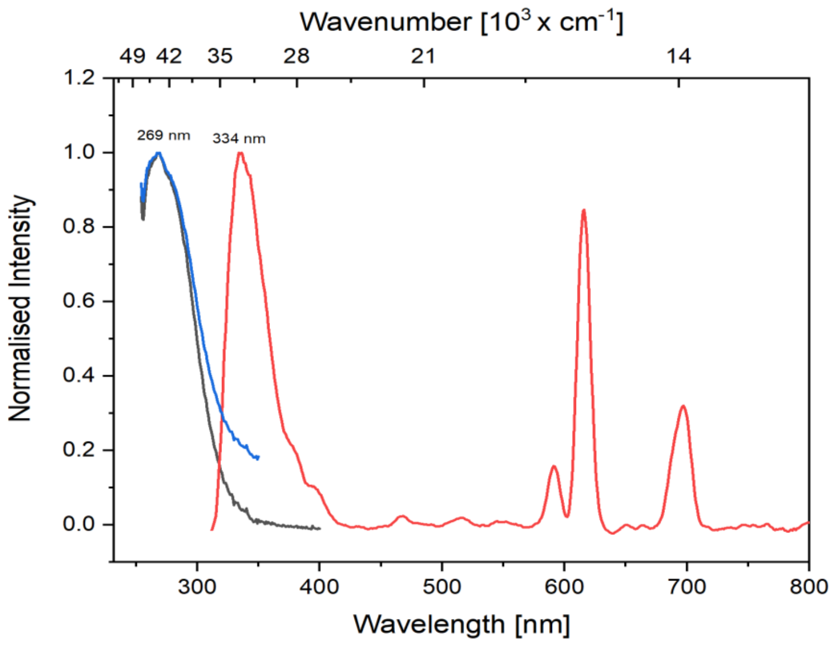

Figure S25. The absorption, excitation (black and blue lines, left, λ_{em} = 269 nm) and steady-state emission spectra (red lines, right, λ_{ex} = 616 nm) of **EuL2m** in DMF. [**EuL2m**] = 120 µM in DMF.

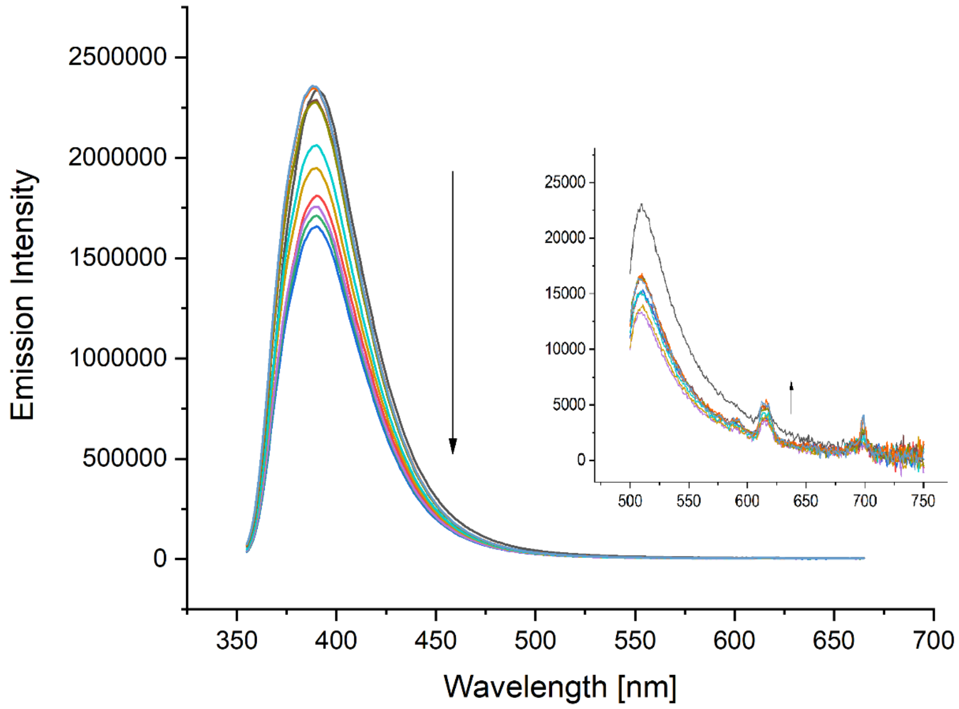

Figure S26. Titration of **L3** (100 µM in DMF) with $EuCl_3$ (100 µM in DMF).

Photostability Experiments

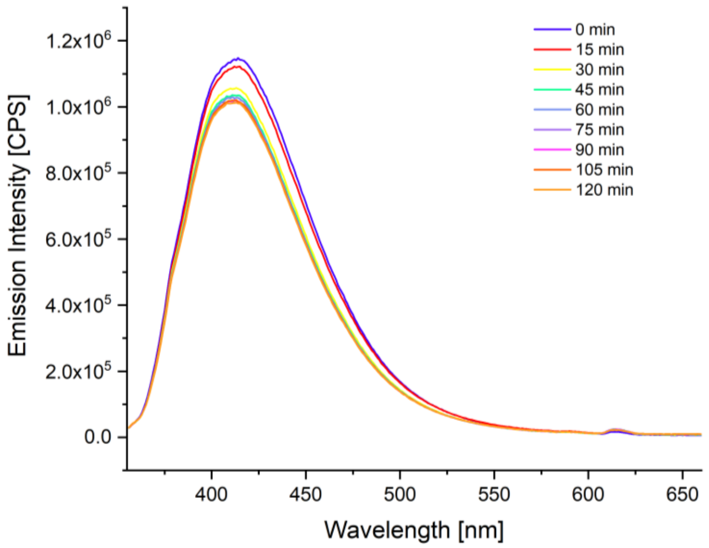

Figure S27. Steady-state fluorescence spectra of aerated **EuL1** upon continuous light irradiation under identical samples absorptions (A = 0.10) in DMF; λ_{ex} = 339 nm, front slit: 3 nm, exit slit: 2.5 nm.

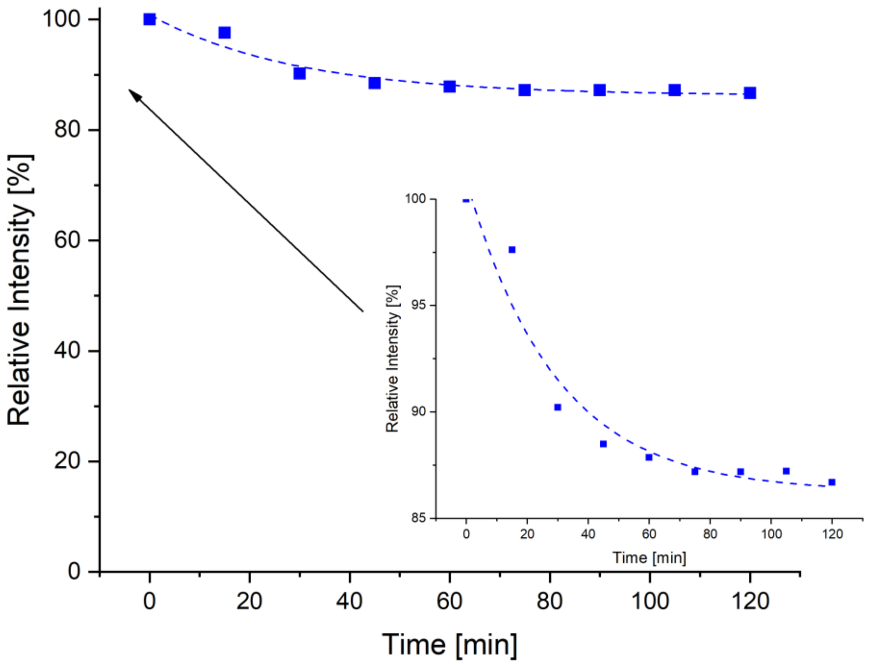

Figure S28. Relative emission intensity of **EuL1** upon continuous light irradiation under identical sample absorptions. After blank signal subtraction each spectrum was integrated (354–663 nm), and the integrated intensity was divided by that at t_0 and multiplied by 100%. The lines next to the data values are only to guide the eye

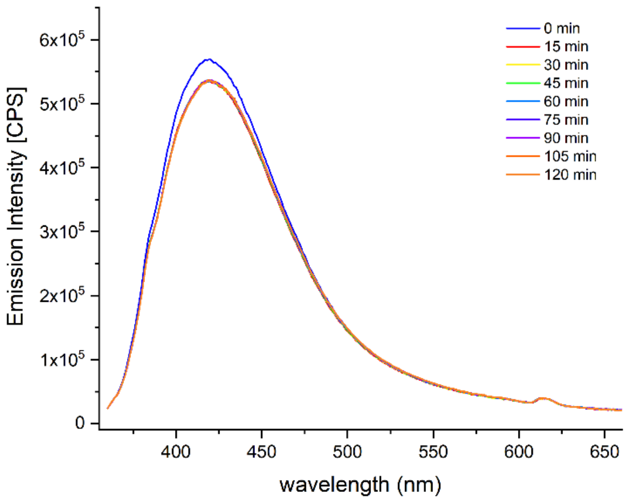

Figure S29. Steady-state fluorescence spectra of aerated **EuL2** upon continuous light irradiation under identical samples absorptions (A = 0.10) in H_2O: DMF; λ_{ex} = 339 nm, front slit: 3 nm, exit slit: 2.5 nm.

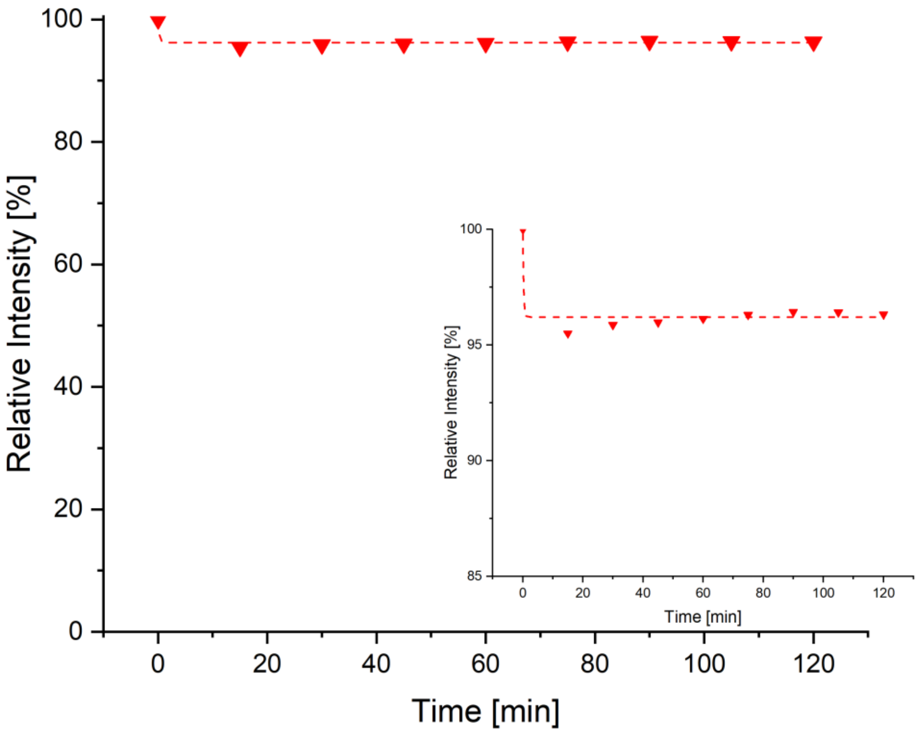

Figure S30. Relative emission intensity of **EuL2** upon continuous light irradiation under identical sample absorptions. After blank signal subtraction each spectrum was integrated (354–663 nm), and the integrated intensity was divided by that at t_0 and multiplied by 100%.

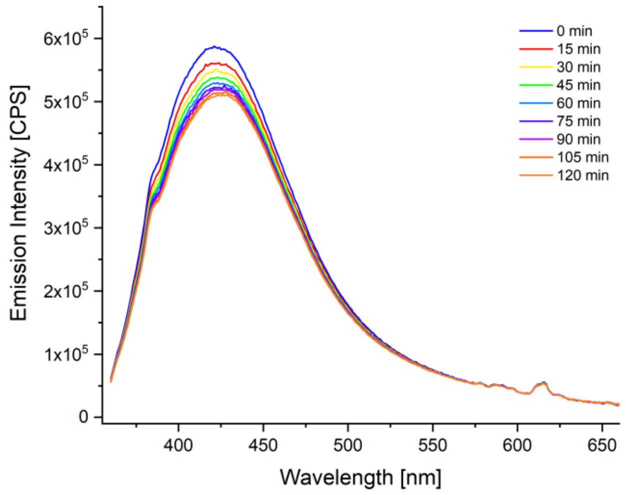

Figure S31. Steady-state fluorescence spectra of aerated **EuL2** upon continuous light irradiation under identical samples absorptions (A = 0.10) in H_2O:DMF with 10 equiv. DIPEA:LiCl; λ_{ex} = 339 nm, front slit: 3 nm, exit slit: 2.5 nm.

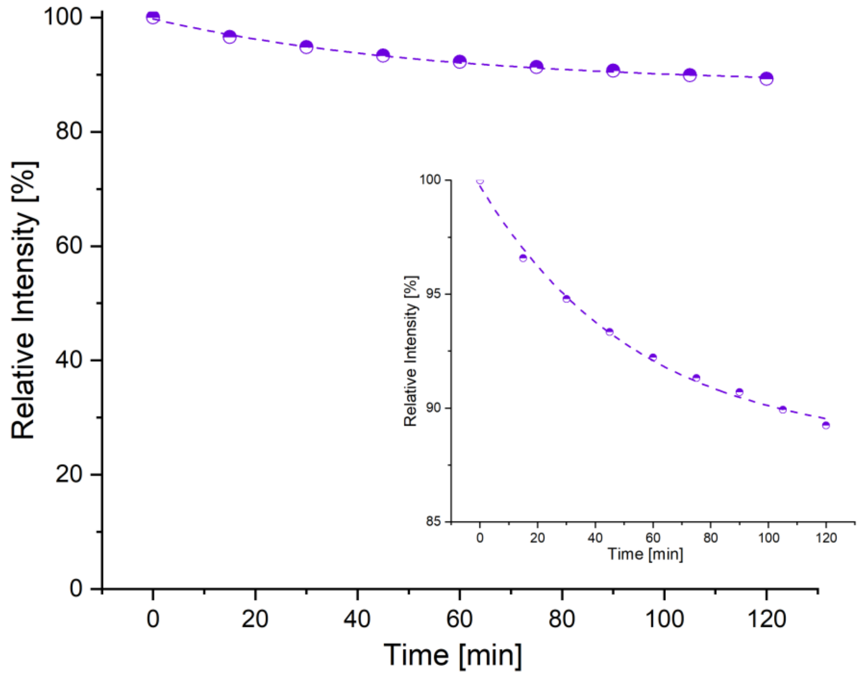

Figure S32. Relative emission intensity of **EuL2** upon continuous light irradiation under identical sample absorptions in H_2O:DMF with 10 equiv. DIPEA:LiCl. After blank signal subtraction each spectrum was integrated (354–663 nm), and the integrated intensity was divided by that at t_0 and multiplied by 100%.

Table S52. Antenna fluorescence quantum yields (Φ_L[%])and Ln(III) emission quantum yield (Φ_{Ln}[%]) of **LnL** (Ln= **Eu, Sm, Gd**; L= **L1, L2, L3**) in DMF.

Complex	Φ_L[%]	Φ_{Ln} [%]	$\Phi_{L,D}$ [%]	$\Phi_{Ln,D}$ [%]
**EuL1**	7.59	0.002	7.65	0.007
**SmL1**	6.56		-	-
**GdL1**	8.12	-	9.78	-
**EuL2**	1.45	0.015	2.61	0.188
**SmL2**	4.27		-	-
**GdL2**	7.19	-	5.07	-

Antenna (Φ_L) and Ln(III) (Φ_{Ln}) fluorescence quantum yields without (Φ_L, Φ_{Ln}) and with ($\Phi_{L,D}$, $\Phi_{Ln,D}$) sacrificial donor (DIPEA (1 equiv.): HCOOH (0.5 equiv.) with **LnL1** and (DIPEA (5 equiv.):LiCl (5 equiv.) with **LnL2**).

Table S53. Lifetimes ($\tau_{f,L}$) of **LnL** (Ln = **Eu, Sm, Gd**; L= **L1, L2, L3**) in DMF. Measured lifetimes ($\tau_{f,L}$) of **GdL, SmL, DyL** based on ligand fluorescence in DMF (λ_{ex} = 341.5 nm, λ_{em} = 412 nm for **SmL1**; 416 nm for **GdL1** and **DyL1**; 417 nm for **LnL2** (Ln = **Gd, Sm , Dy**); 385 nm for GdL3; monoexponential and biexponential reconvolution fit). The χ^2 value is the goodness of fit (χ^2 = 1 is the best fit).

Complex	$\tau_{f,L}$ (ms*/ns)	χ^2
**EuL1**	0.51 ms*	-
**SmL1**	t_1 = 0.59 ns (90.7%)<br>t_2 = 2.83 ns (9.2%)	1.28
**GdL1**	t_1 = 0.64 ns (97.1%)<br>t_2 = 3.1 ns (2.8%)	1.28
**DyL1**	t_1 = 0.68 ns (90.1%)<br>t_2 = 3.08 ns (9.9%)	1.31
**EuL2**	0.57 ms*	-
**SmL2**	t_1 = 0.79 ns (82.3%)<br>t_2 = 3.13 ns (17.6%)	1.54
**GdL2**	t_1 = 0.83 ns (67.8%)<br>t_2 = 2.4 ns (32.1%)	1.28
**DyL2**	t_1 = 0.80 ns (81.4%)<br>t_2 = 3.14 ns (18.5%)	1.45
**EuL3**	0.70 ms*	-
**GdL3**	t_1 = 1.3 ns	1.37

*Emission based lifetime. All other values are antenna fluorescence lifetimes.

Antenna fluorescence lifetime decays

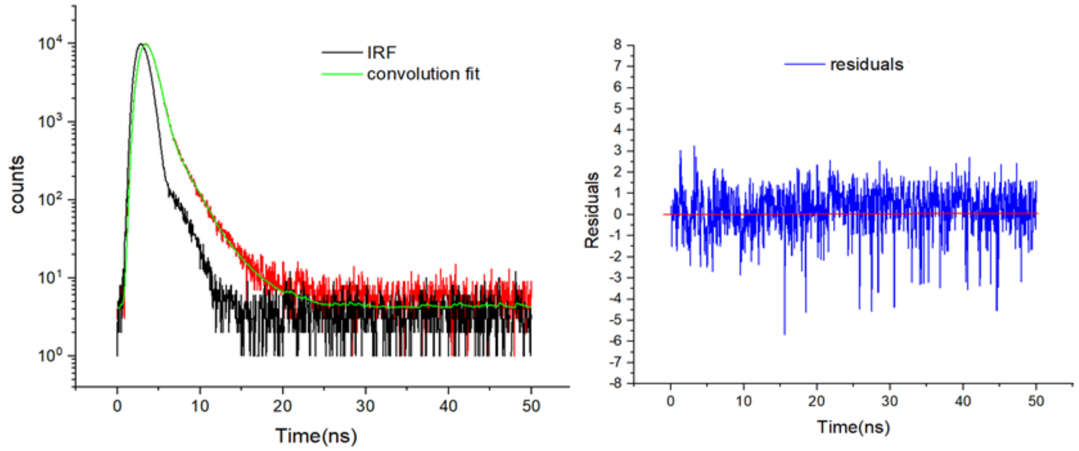

Figure S33. The fluorescence decay and reconvolution fit of **GdL1** in DMF (left, red line) and residuals of the fit (right).

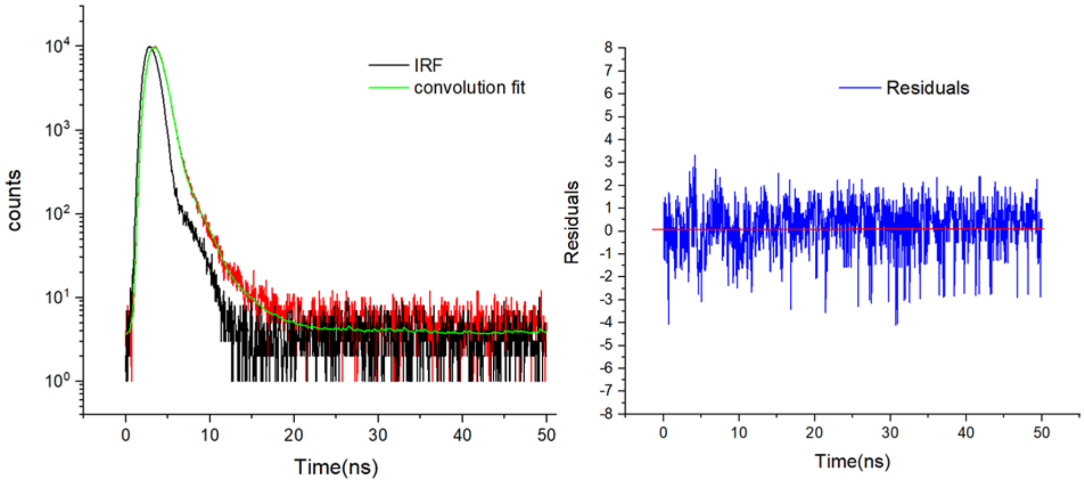

Figure S34. The fluorescence decay and reconvolution fit of **SmL1** in DMF (left, red line) and residuals of the fit (right).

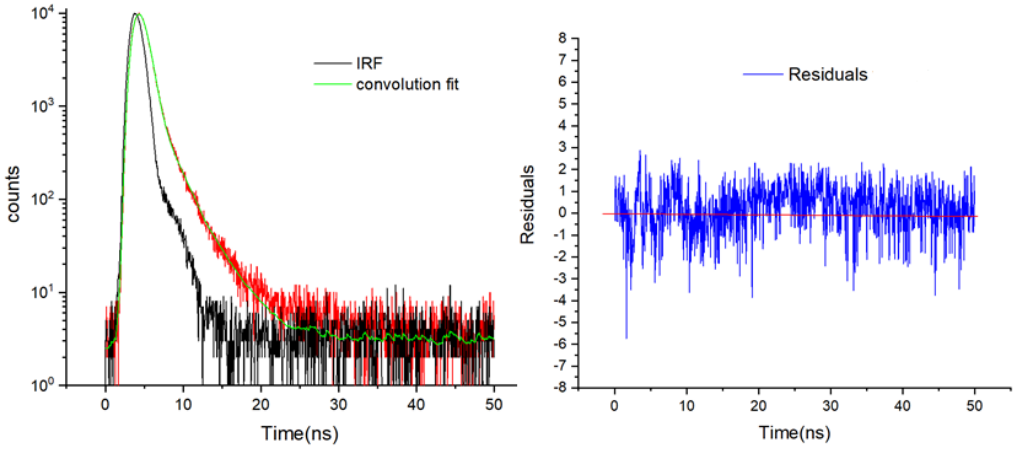

Figure S35. The fluorescence decay and reconvolution fit of **DyL1** in DMF (left, red line) and residuals of the fit (right).

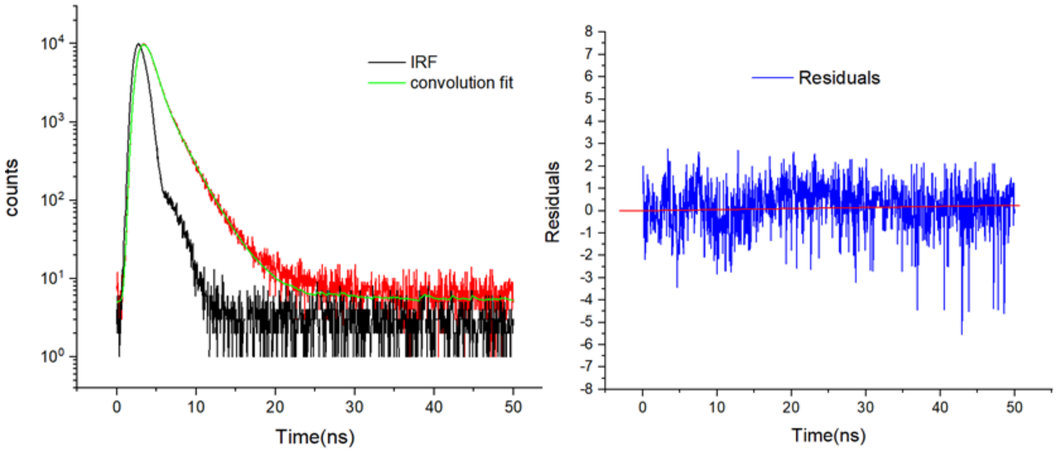

Figure S36. The fluorescence decay and reconvolution fit of **GdL2** in DMF (left, red line) and residuals of the fit (right).

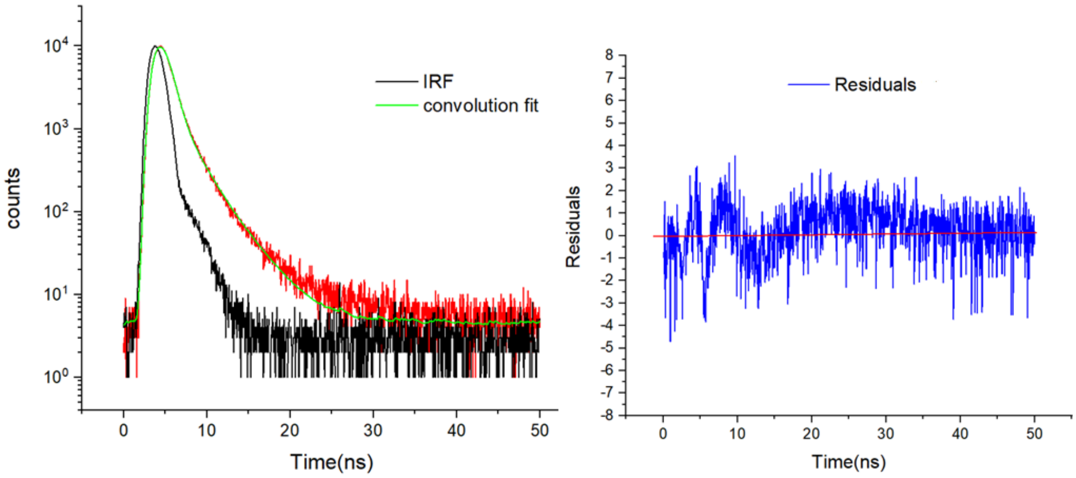

Figure S37. The fluorescence decay and reconvolution fit of **SmL2** in DMF (left, red line) and residuals of the fit (right).

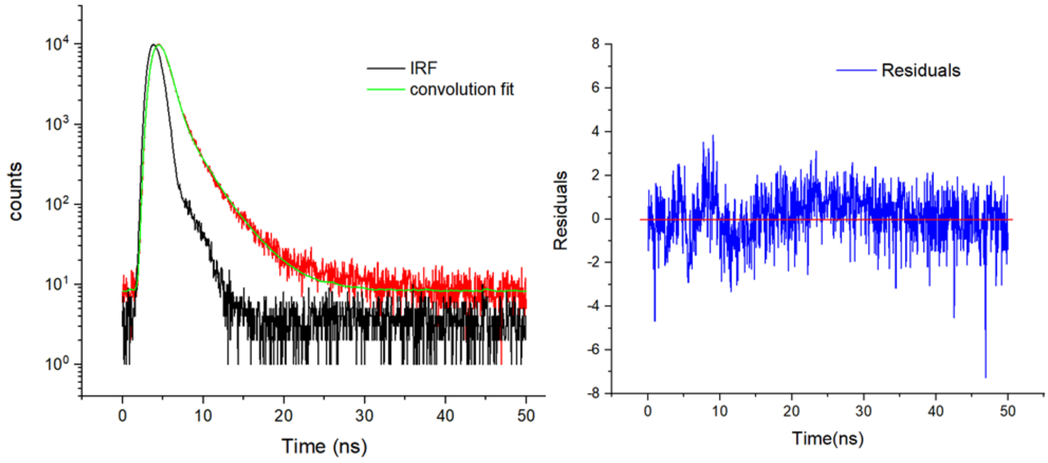

Figure S38. The fluorescence decay and reconvolution fit of **DyL2** in DMF (left, red line) and residuals of the fit (right).

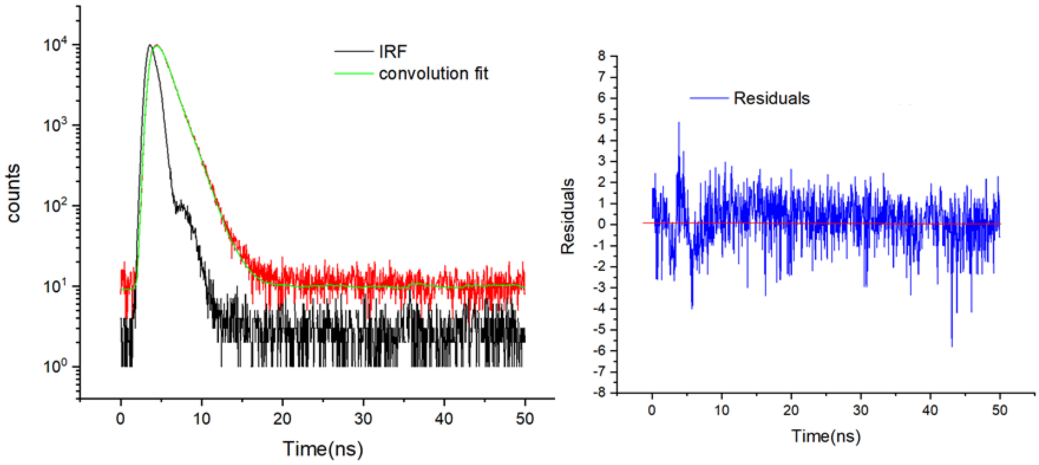

Figure S39. The fluorescence decay and reconvolution fit of **GdL3** in DMF (left, red line) and residuals of the fit (right).

FT-IR spectra

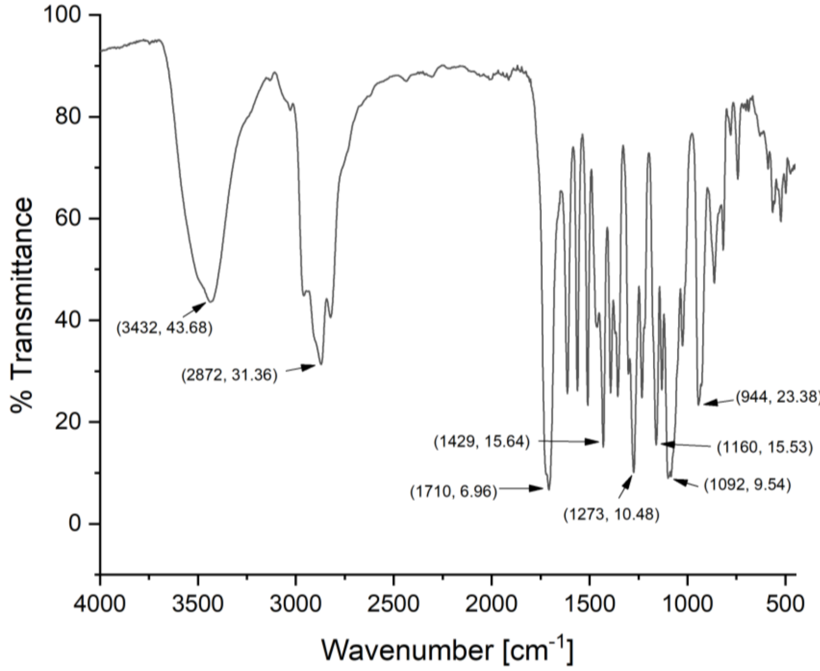

Figure S40. FT-IR spectrum of **L1**.

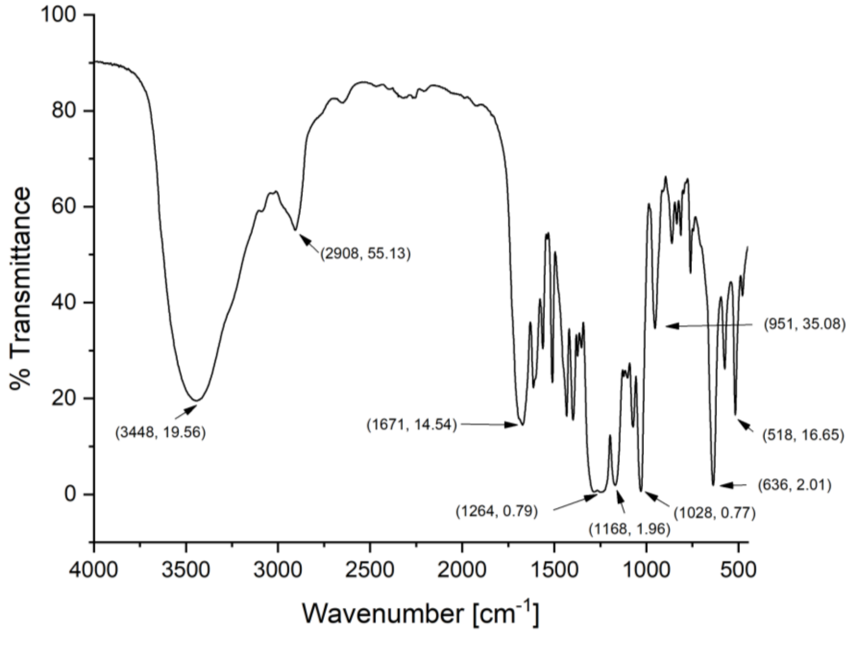

Figure S41. FT-IR spectrum of **EuL1**.

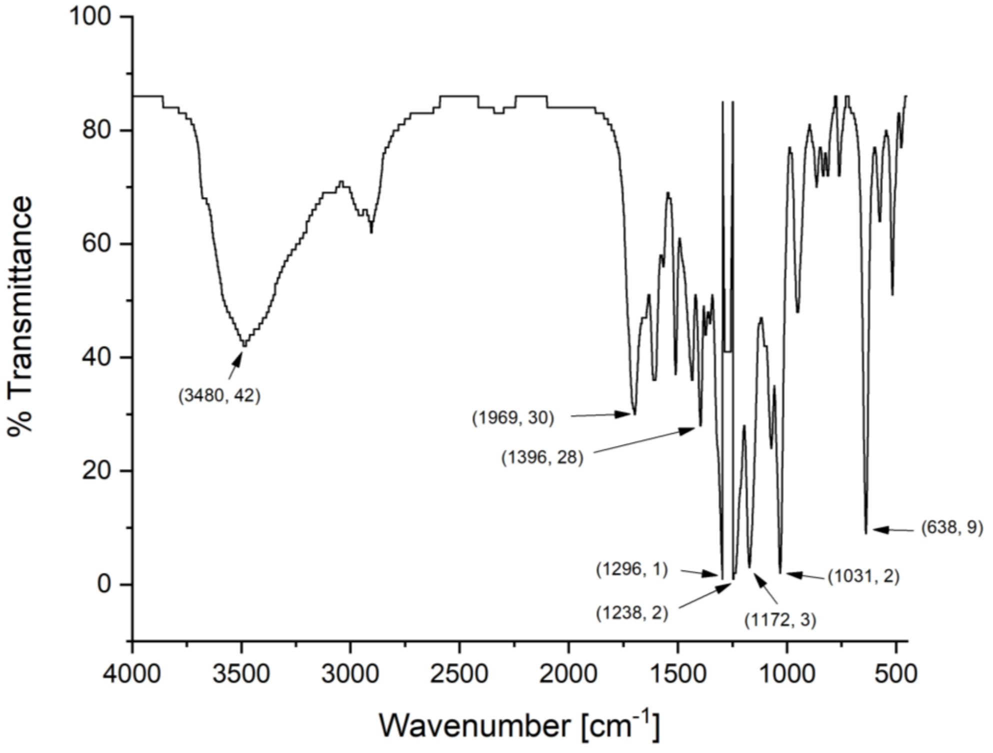

Figure S42. FT-IR spectrum of **SmL1.**

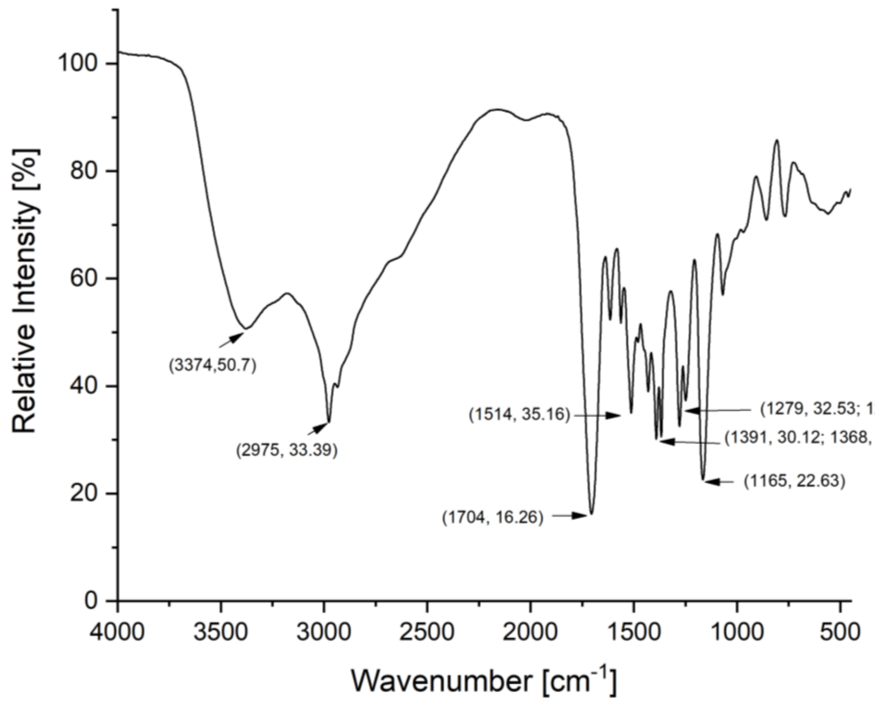

Figure S43. FT-IR spectrum of **L2**.

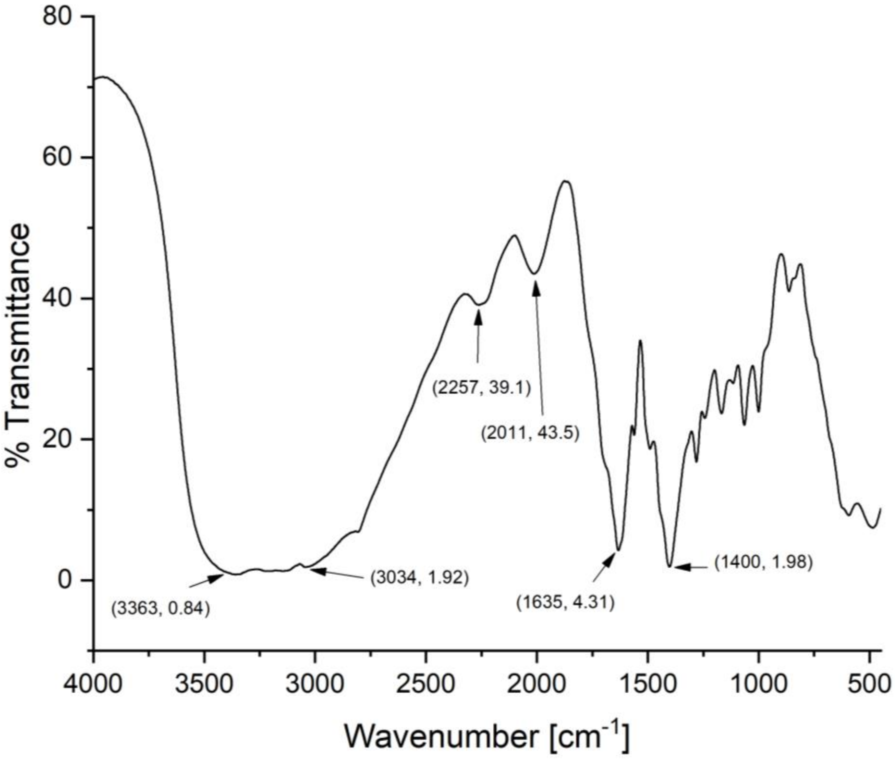

Figure S44. FT-IR spectrum of **EuL2.**

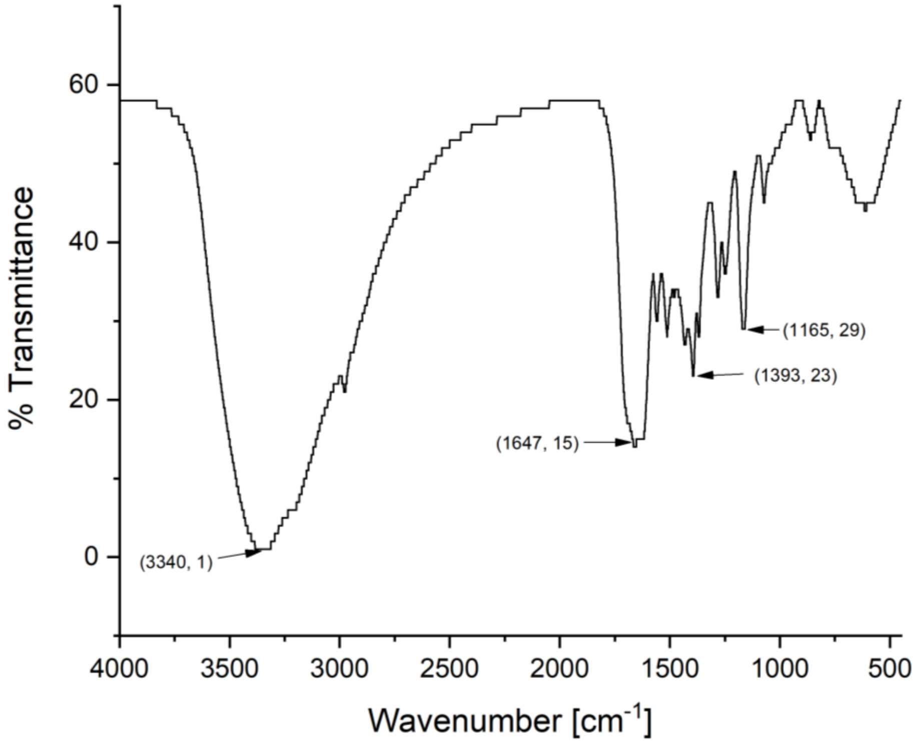

Figure S45. FT-IR spectrum of **SmL2.**

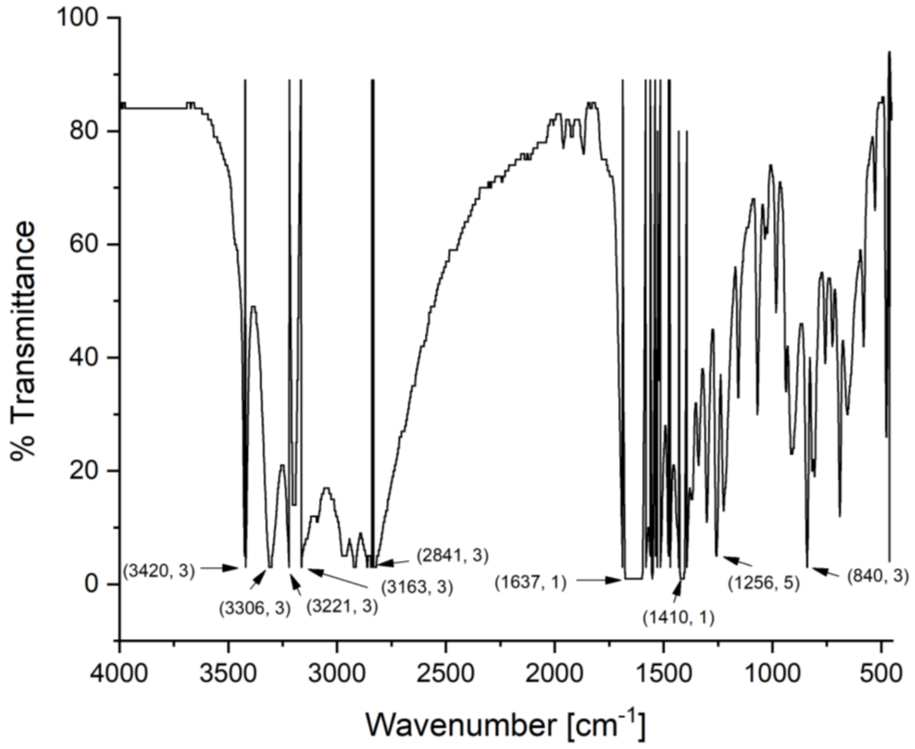

Figure S46. FT-IR spectrum of **L3**.

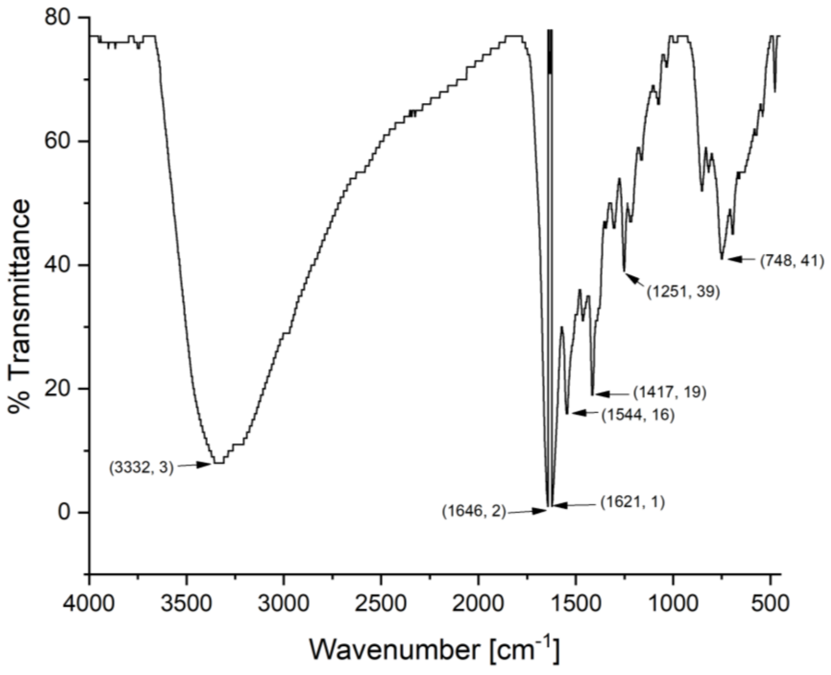

Figure S47. FT-IR spectrum of **EuL3.**

Table S54. Tabulated FT-IR data of **LnL** (Ln= **Eu, Sm, Gd**; L=**L1, L2, L3**).

Compound	Wavenumber (cm^{-1})
**L1**	2872, 1710, 1429, 1273, 1160, 1092, 944
**EuL1**	3448, 2908, 1671, 1264, 1168, 1028,951, 636, 518
**SmL1**	3480, 1969, 1396, 1296, 1238, 1172, 1031, 638
**L2**	3375, 2975, 1704, 1514, 1391, 1368, 1279, 1247, 1165
**EuL2**	3363, 3034, 2257, 2011, 1635, 1400
**SmL2**	3340, 1647, 1393, 1165
**L3**	3420, 3306, 3221, 3163, 2841, 1637, 1410, 1256, 840
**EuL3**	3332, 1646, 1621, 1544, 1417, 1251, 748

Electrochemical characterization

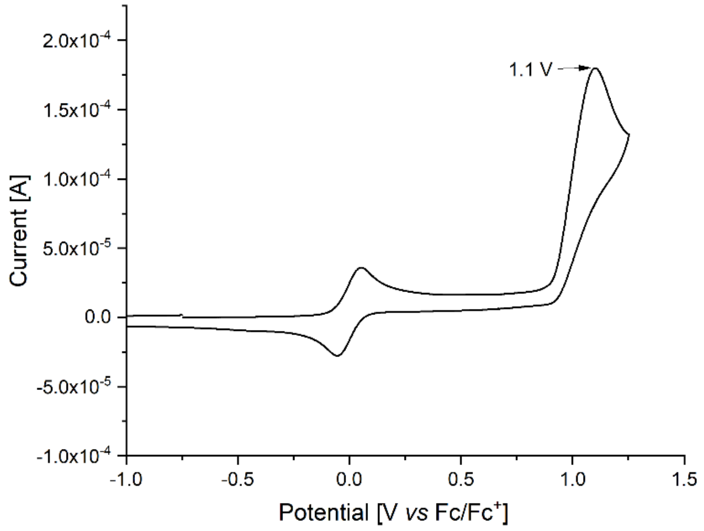

Figure S48. Cyclic voltammogram of model antenna (**S1**) in DMF (0.1 M $TBAPF_6$). [**S1**] = 5 mM; reference electrode, Ag wire; working electrode, GC electrode; counter electrode, Pt wire; scan rate, 0.1 V/s. All measurements were conducted in a glovebox.

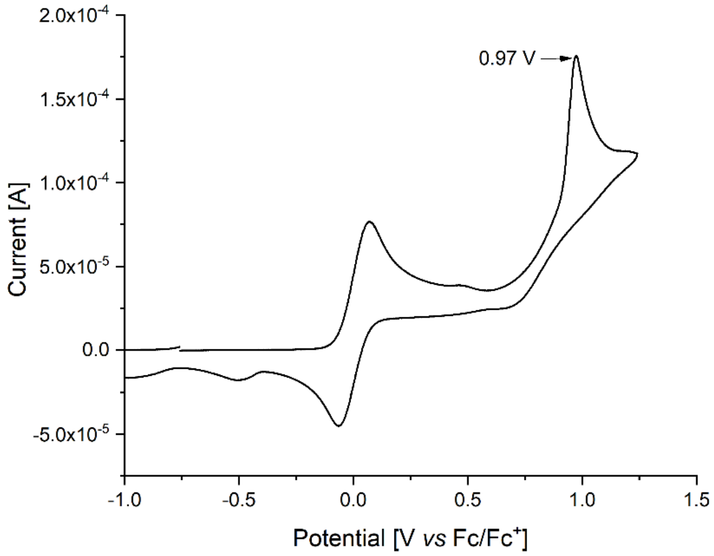

Figure S49. Cyclic voltammogram of **GdL3** in DMF (0.1 M $TBAPF_6$). [**GdL3**] = 2 mM; reference electrode, Ag wire; working electrode, GC electrode; counter electrode, Pt wire; scan rate, 0.1 V/s. All measurements were conducted in a glovebox.

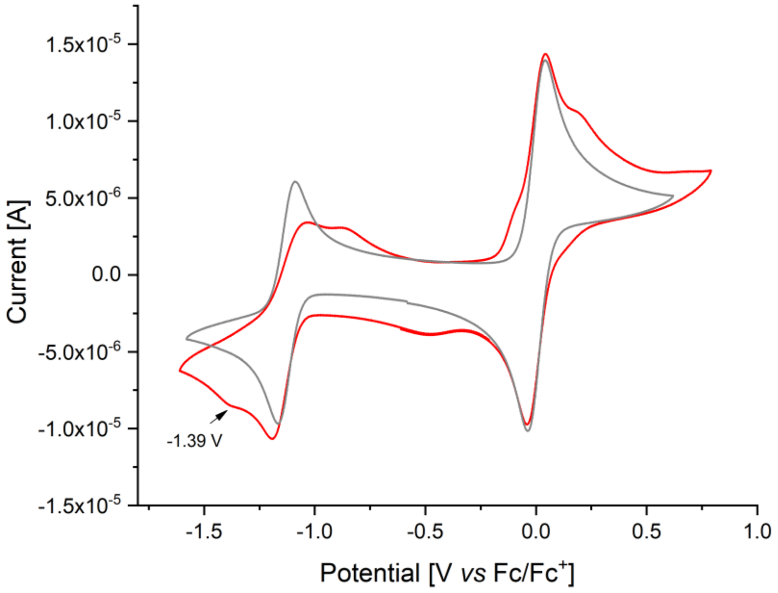

Figure S50. Cyclic voltammogram of **EuL1** and $Eu(OTf)_3$ in DMF (0.1 M $TBAPF_6$). [**EuL1** and $Eu(OTf)_3$] = 2 mM; reference electrode, Ag wire; working electrode, GC electrode; counter electrode, Pt wire; scan rate, 0.1 V/s. All measurements were conducted in a glovebox. Additional peaks are assigned to Fc^+/Fc and excess $Eu(OTf)_3$.

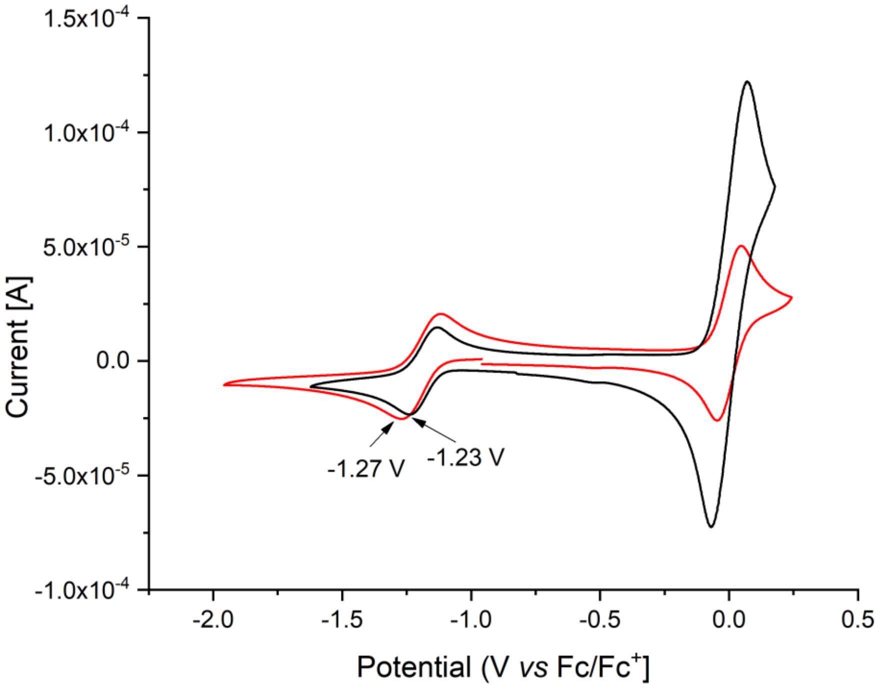

Figure S51. Cyclic voltammogram of **EuL2** (red line) and **EuL2m** (black line) in DMF (0.1 M $TBAPF_6$). [**EuL2** and **EuL2**$^{\mathbf{m}}$] = 2 mM; reference electrode, Ag wire; working electrode, GC

electrode; counter electrode, Pt wire; scan rate, 0.1 V/s. All measurements were conducted in a glovebox.

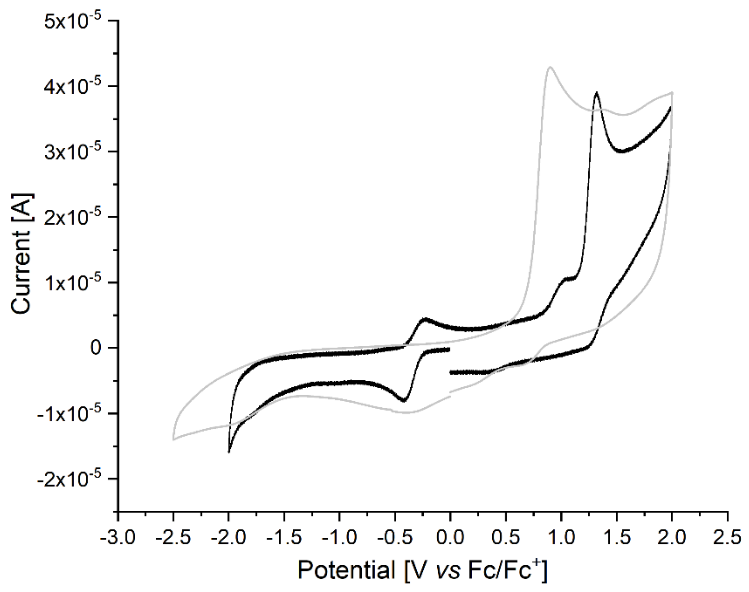

Figure S52. Cyclic voltammogram of **EuL3** (black line, first cycle, without Fc) and (grey line, last cycle with Fc) in DMF (0.1 M $TBAPF_6$). [**EuL3**] = 2 mM; reference electrode, Ag wire; working electrode, GC electrode; counter electrode, Pt wire; scan rate, 0.1 V/s. All measurements were conducted in a glovebox.

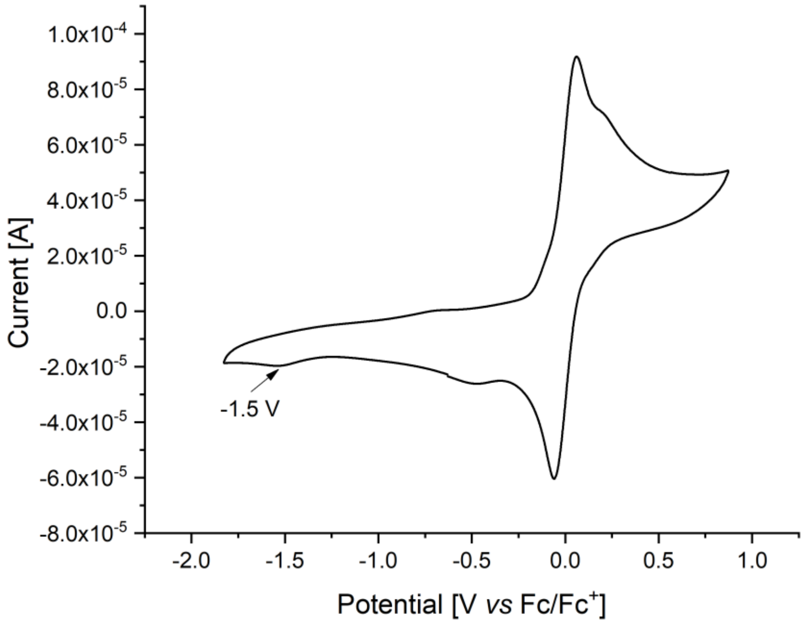

Figure S53. Cyclic voltammogram of **SmL1** in DMF (0.1 M $TBAPF_6$). [**SmL1**] = 2 mM; reference electrode, Ag wire; working electrode, GC electrode; counter electrode, Pt wire; scan rate, 0.1 V/s. All measurements were conducted in a glovebox.

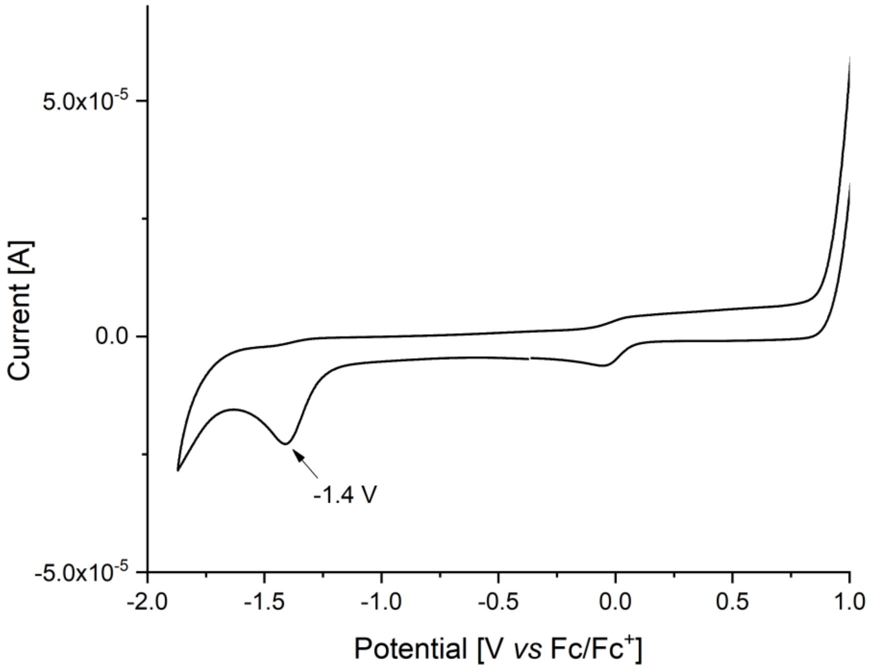

Figure S54. Cyclic voltammogram of **SmL2** in DMF (0.1 M $TBAPF_6$). [**SmL2**] = 2 mM; reference electrode, Ag wire; working electrode, GC electrode; counter electrode, Pt wire; scan rate, 0.1 V/s. All measurements were conducted in a glovebox.

Calculation of excited state potential

The excited-state oxidation potential ($E^*_{1/2}$) was calculated according to Rehm–Weller formalism equation (S4)

$$E^*_{1/2} = E_{1/2} - E_{0/0} \qquad \text{(S4)}^{55}$$

with the ground-state potential ($E_{1/2}$, Fig S46, S47) and the energy of the emission band ($E_{0,0}$), which is the energy of an electron in the excited state relative to the ground state as determined by the maximum emission wavelength (Fig S22). There is an additional work-function term that has been omitted from equation S4 because it was assumed to be negligibly small.

Calculated for model coumarin antenna (**S1**) and **L3**:

$$E(\boldsymbol{S1})^*_{1/2} = E_{1/2} - E_{0/0} = 1.10 - 2.98 = -1.88 \text{ V } vs \text{ Fc}^+/\text{Fc}$$

$$E(\boldsymbol{L3})^*_{1/2} = E_{1/2} - E_{0/0} = 0.97 - 3.22 = -2.25 \text{ V } vs \text{ Fc}^+/\text{Fc}$$

Table S55.

Compound	$E_{1/2}$ [V *vs* Fc^+/Fc]	λ_{em}, $E_{0/0}$	$E^*_{1/2}$ [V *vs* Fc^+/Fc]
**S1**	1.10*	415 nm, 2.98	–1.88
**GdL3**	0.97*	385 nm, 3.22	–2.25

* Irreversible antenna oxidation under experimental conditions, oxidation potential is used.

Calculation of driving force for photoinduced electron transfer

The driving force for photoinduced electron transfer from the excited coumarin antenna (coum) was calculated according to Eq (S5):[56]

$$\Delta G(eT) = (E_{ox}^{cou} - E_{red}^{LnL}) - E_{s}^{cou} - \frac{{e_0}^2}{\varepsilon r} \quad \text{(S5)}$$

with $\Delta G(eT)$ the free energy of electron transfer, E_{ox}^{cou} the oxidation potential of the ground state coumarin antenna (1.10 V *vs* Fc^{+}/Fc),[1] E_{red}^{LnL} the reduction potential of **LnL** complex (Ln = **Eu, Sm**; L= **L1, L2**), E_{s}^{cou} the singlet excited state of the antenna (3.17 eV for **Ln1** and 3.14 eV for **Ln2**), and $\frac{{e_0}^2}{\varepsilon r}$ the attraction between the radical ion pair (~0.15 eV for an exciplex).[57]

Table S56. Reduction potential of **LnL** and PeT driving force from the excited coumarin antenna.

Complex	E_{red}^{LnL} [V *vs* Fc/Fc^{+}]	$\Delta G(eT)$[eV]
**EuL1**	–1.39	–0.83
**SmL1**	–1.50	–0.72
**EuL2**	–1.27	–0.92
**SmL2**	–1.40	–0.79

$$\Delta G(eV) = \left(E_{ox}^{cou} - E_{red}^{EuL1}\right) - E_{s}^{cou} - \frac{{e_0}^2}{\varepsilon r} = \left(1.10\ eV - (-1.39\ eV)\right) - 3.17\ eV - 0.15\ eV = -0.83\ eV$$

Substrate binding study

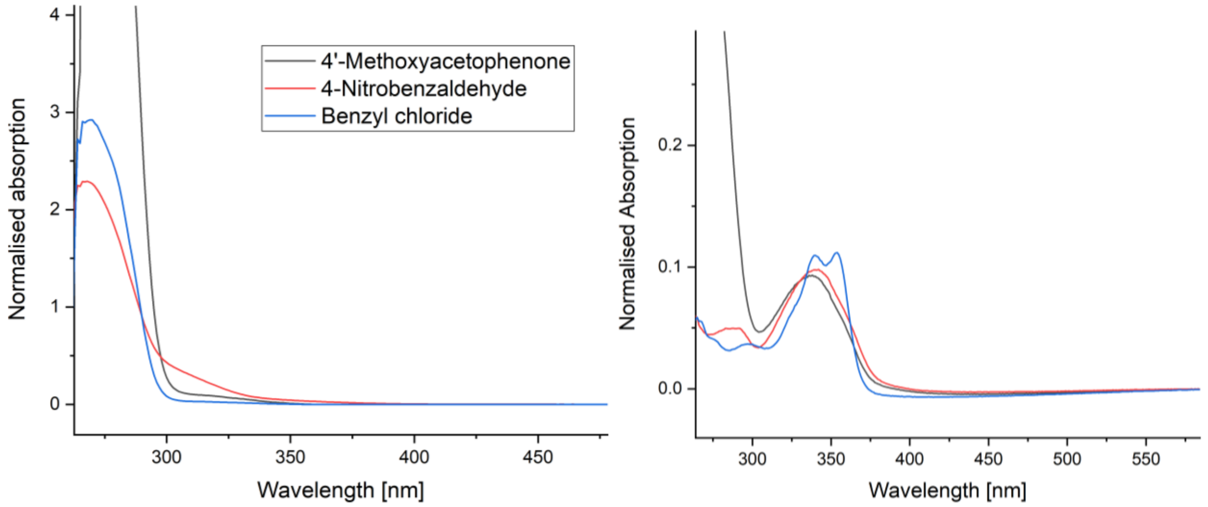

Figure S55. (Left) Normalised absorption spectra of **1a** (**BnCl**, blue), **35a** (4-methoxyacetophenone, black), **EuL1** and **36a** (4-nitrobenzaldehyde, red) in DMF; (right) Normalised absorption spectra of **EuL1** (black), **EuL2** (red), **EuL3** (blue) in DMF.

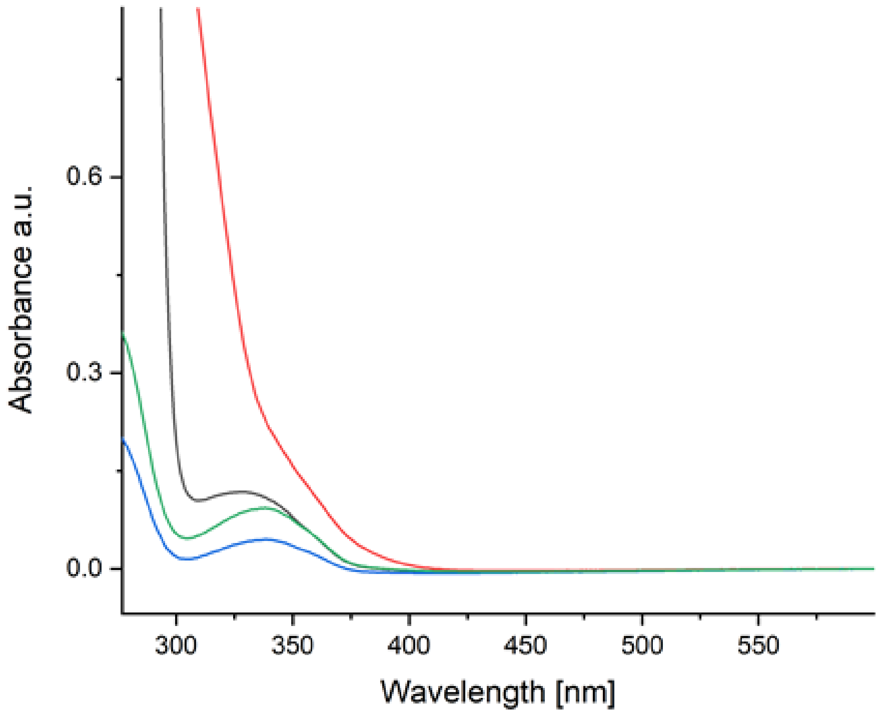

Figure S56. Effect of substrate binding on the absorption of **EuL1**. **EuL1** (green), **EuL1** + 10 equiv. of **1a** (blue), **EuL1** + 10 equiv. of **35a** (black), **EuL1** + 10 equiv. of **36a** (red) in DMF.

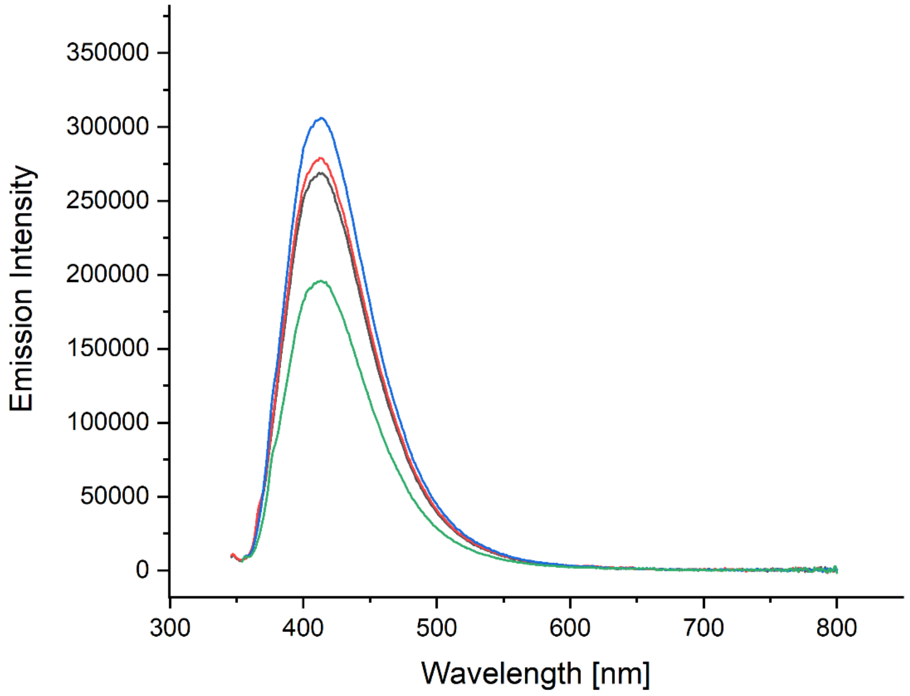

Figure S57. Effect of substrate binding on steady-state emission of **EuL1** in DMF. **EuL1** (black), **EuL1** + 10 equiv. of **1a** (red), **EuL1** + 10 equiv. of **35a** (blue), **EuL1** + 10 equiv. of **36a** (green), λ_{ex} = 339 nm.

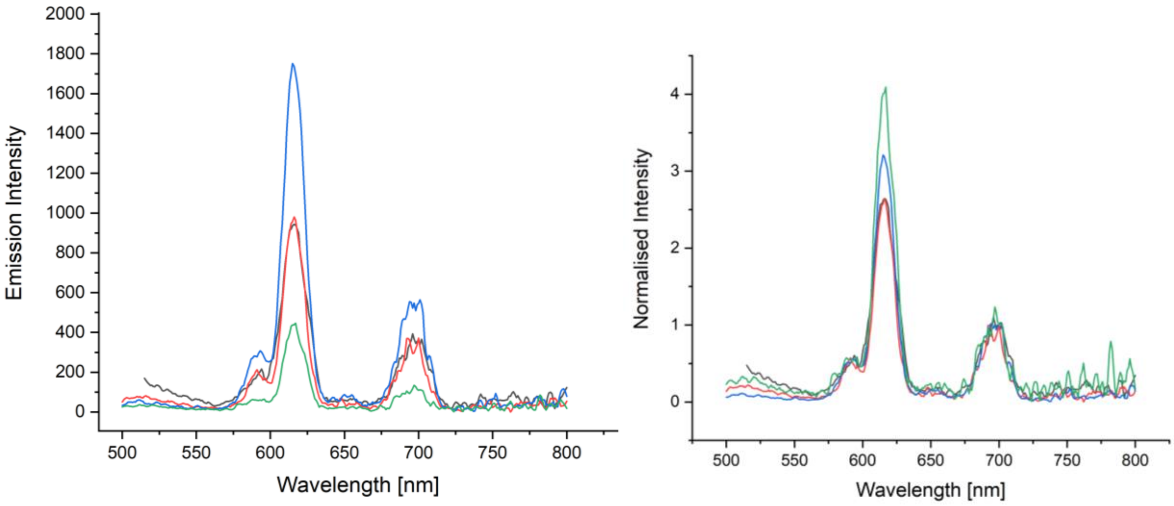

Figure S58. (left) Effect of substrate binding on the time-resolved emission of **EuL1** in DMF. **EuL1** (black), **EuL1** + 10 equiv. of **1a** (red), **EuL1** + 10 equiv. of **35a** (blue), **EuL1** + 10 equiv. of **36a** (green), (λ_{ex} = 339 nm). (right) Emission spectra of **EuL1** normalised at 700 nm.

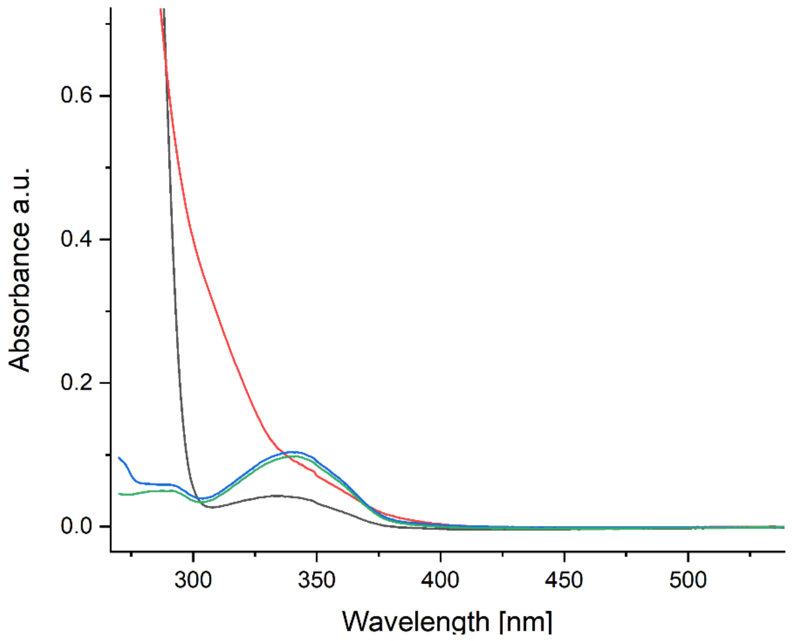

Figure S59. Effect of substrate binding on the absorption spectrum of **EuL2**. **EuL2** (green), **EuL2** + 10 equiv. of **1a** (blue), **EuL2** + 10 equiv. of **35a** (black), **EuL2** + 10 equiv. of **36a** (red).

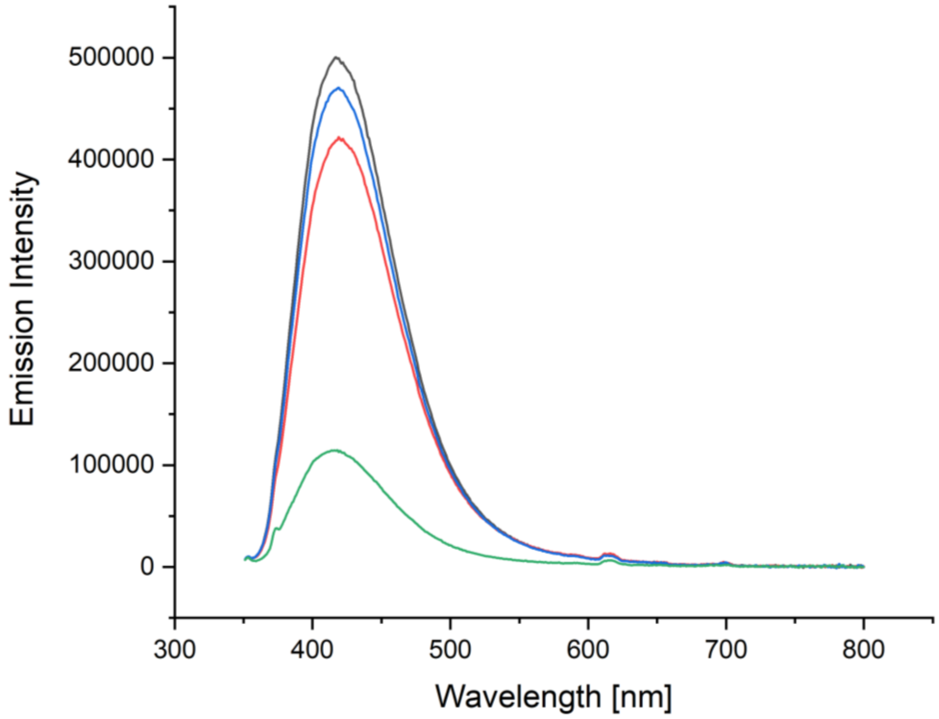

Figure S60. Effect of substrate binding on the steady-state emission of **EuL2**. **EuL2** (black), **EuL2** + 10 equiv. of **1a** (red), **EuL2** + 10 equiv. of **35a** (blue), **EuL2** + 10 equiv. of **36a** (green), λ_{ex} = 336 nm.

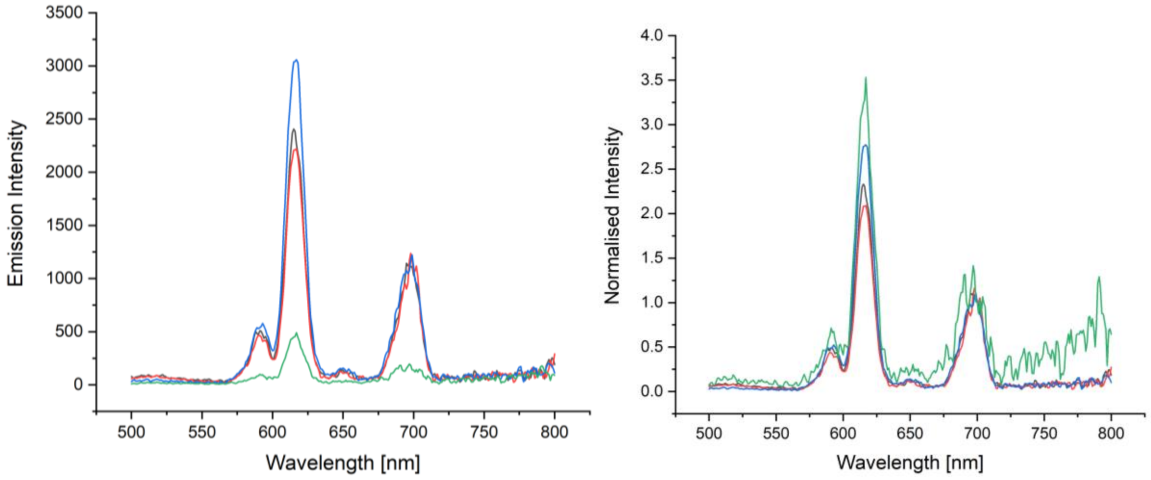

Figure S61. Effect of substrate binding on the time-resolved emission of **EuL2** in DMF. **EuL2** (black), **EuL2** + 10 equiv. of **1a** (red), **EuL2** + 10 equiv. of **35a** (blue), **EuL2** + 10 equiv. of **36a** (green), λ_{ex} = 336 nm. (right) Emission spectra of **EuL2** normalised at 700 nm.

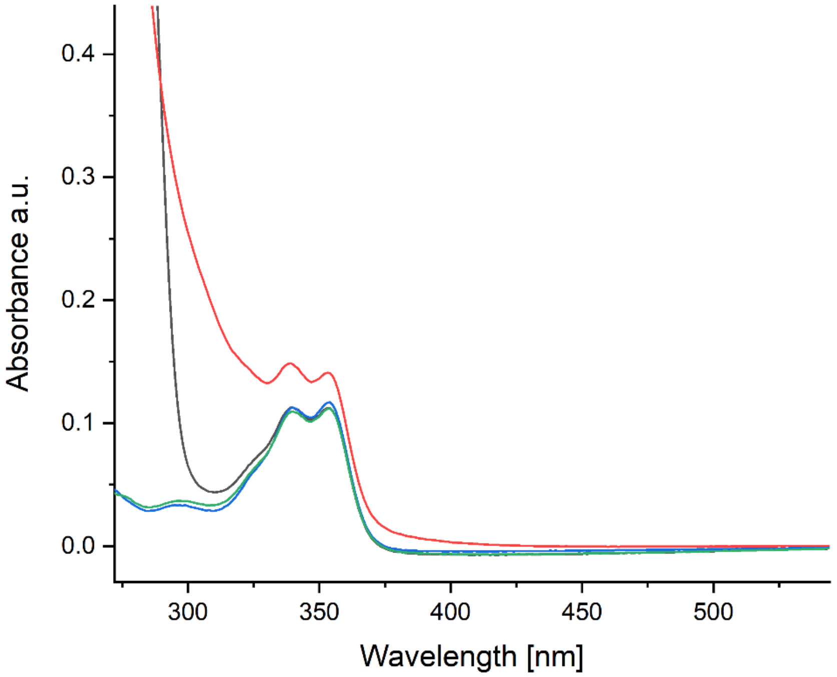

Figure S62. Effect of substrate binding on the absorption of **EuL3**. **EuL3** (green), **EuL3** + 10 equiv. of **1a** (blue), **EuL3** + 10 equiv. of **35a** (black), **EuL3** + 10 equiv. of **36a** (red).

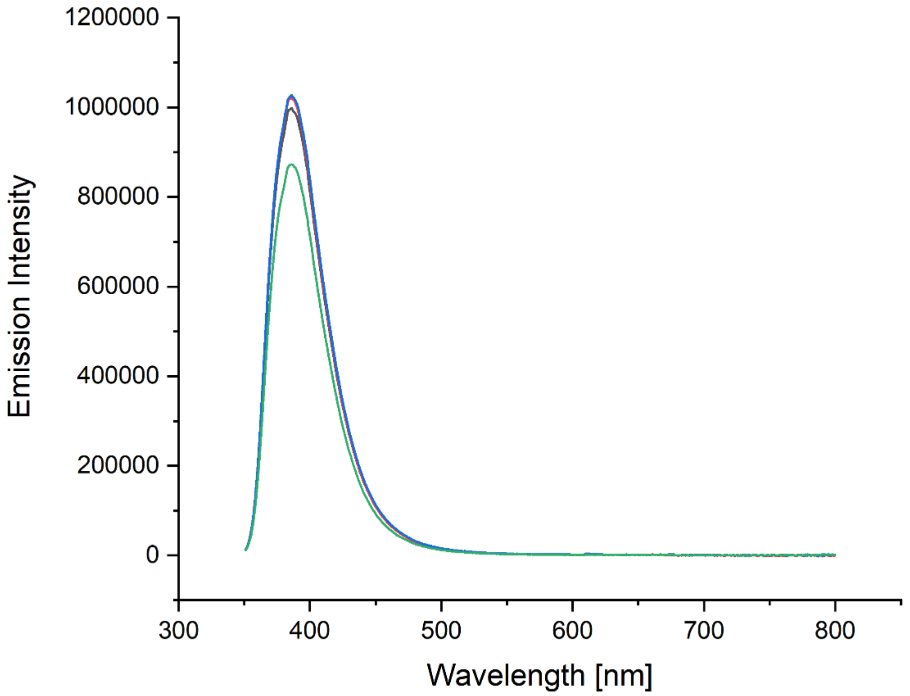

Figure S63. Effect of substrate binding on the steady-state emission of **EuL3**. **EuL3** (black), **EuL3** + 10 equiv. of **1a** (red), **EuL3** + 10 equiv. of **35a** (blue), **EuL3** + 10 equiv. of **36a** (green), λ_{ex} = 346 nm.

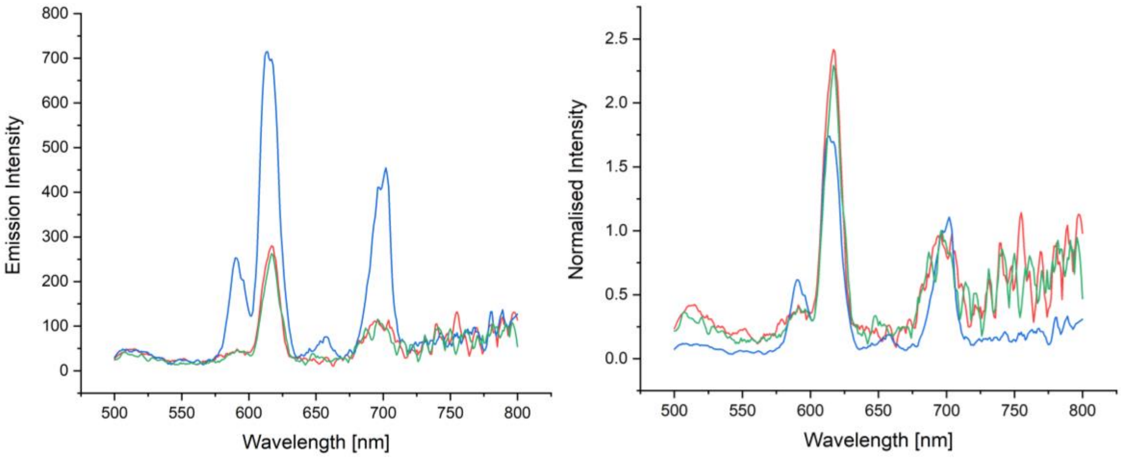

Figure S64. Effect of substrate binding on the time-resolved emission of **EuL3**. (left) **EuL3** + 10 equiv. of **1a** (red), **EuL3** + 10 equiv. of **35a** (blue), **EuL3** + 10 equiv. of **36a** (green), λ_{ex} = 346 nm. Emission spectra of **EuL3** normalised at 700 nm (right).

Table S57. Luminescence lifetimes (τ_{Eu}) of **EuL** (L= **L1, L2, L3**) in substrate-containing (10 equiv.) in DMF.

**Complexes**	τ_{Eu} / ms
**EuL1**	0.51
**EuL1** + **1a**	0.97
**EuL1** + **35a**	0.91 (53%) + 0.10 (47%)
**EuL1** + **36a**	0.89 (48%) + 0.10 (52%)
**EuL2**	0.57
**EuL2** + **1a**	1.05
**EuL2** + **35a**	1.10 (56%) + 0.24 (44%)
**EuL2** + **36a**	1.01 (56%) + 0.25 (44%)
**EuL3**	0.70
**EuL3** + **1a**	0.66
**EuL3** + **35a**	1.89
**EuL3** + **36a**	0.79 (49%) + 0.13 (51%)

Substrate binding study using ^{19}F NMR

Following a reported procedure,[58] pseudocontact shifts (PCS) were studied upon binding of substrate **22a** to **EuL1** or **EuL2** using ^{19}F NMR spectroscopy. To minimize error, another nonbinding compound (C_6F_6) was added as an internal standard. Various amounts of **22a** (1 equiv. to 10 equiv. with respect to **EuL1**/**EuL2**) were studied. The results indicate varying degrees of binding of **22a** to **EuL1** and **EuL2**.

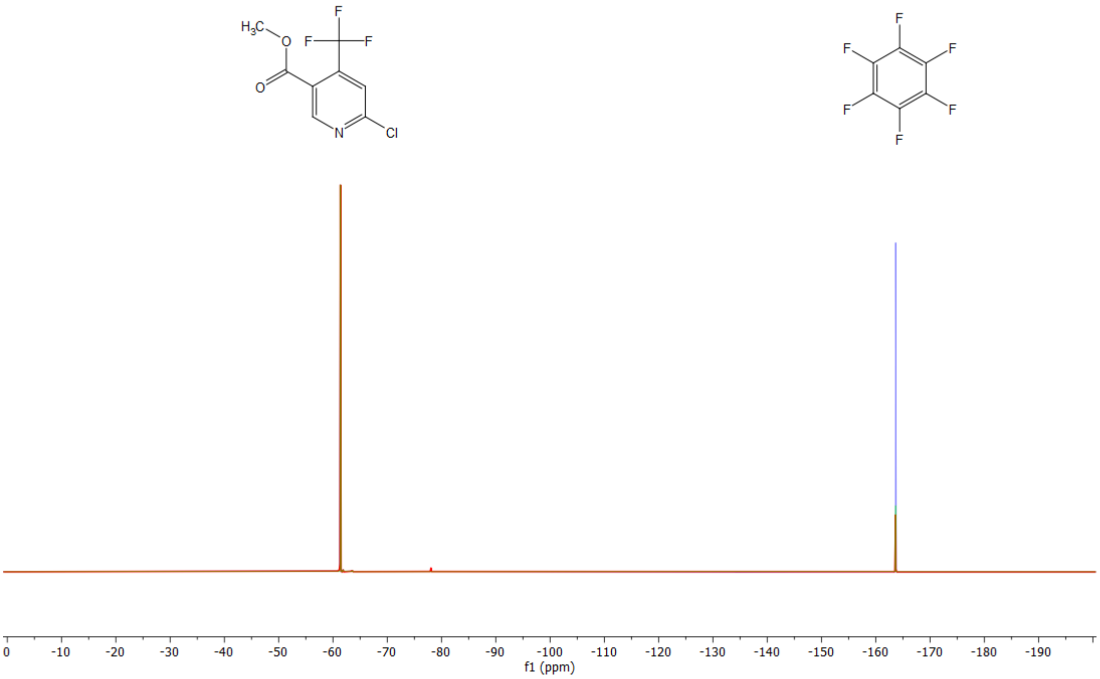

Figure 65. ^{19}F NMR spectra of solutions in DMF of **22a**, **EuL1**:**22a** (1:10), **EuL2**:**22a** (1:10) in the presence of internal standard hexafluorobenzene.

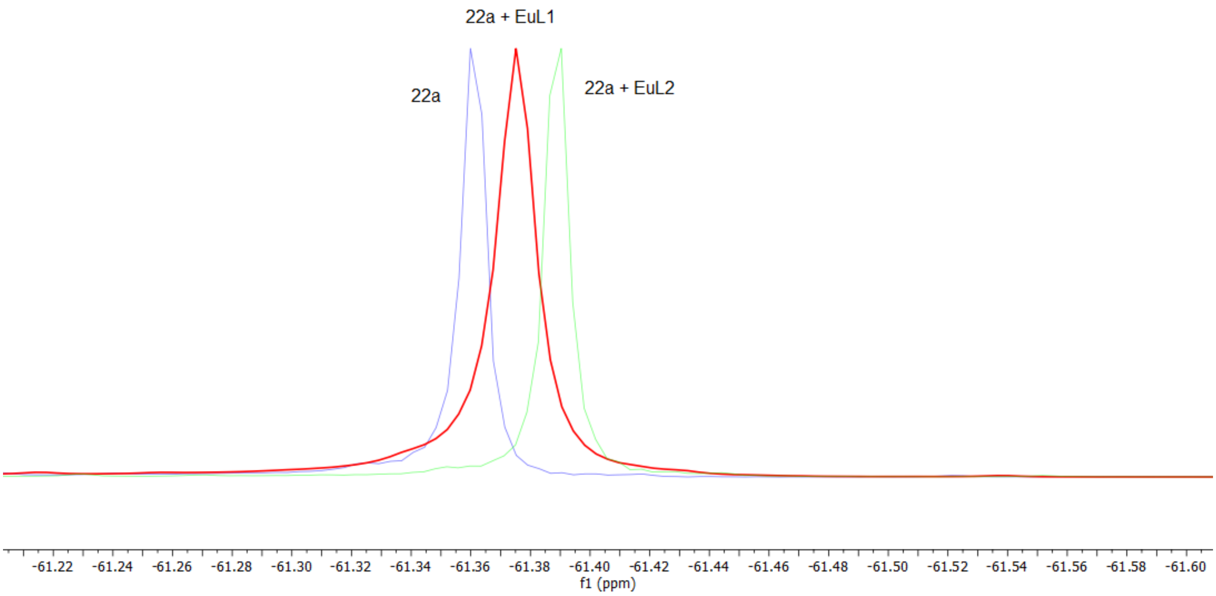

Figure S66. ^{19}F NMR spectra (expansion) of solutions in DMF of **22a**, **EuL1**:**22a** (1:10), **EuL2**:**22a** (1:10). Changes relative to **22a** are attributed to coordination to **EuL1** and **EuL2**.

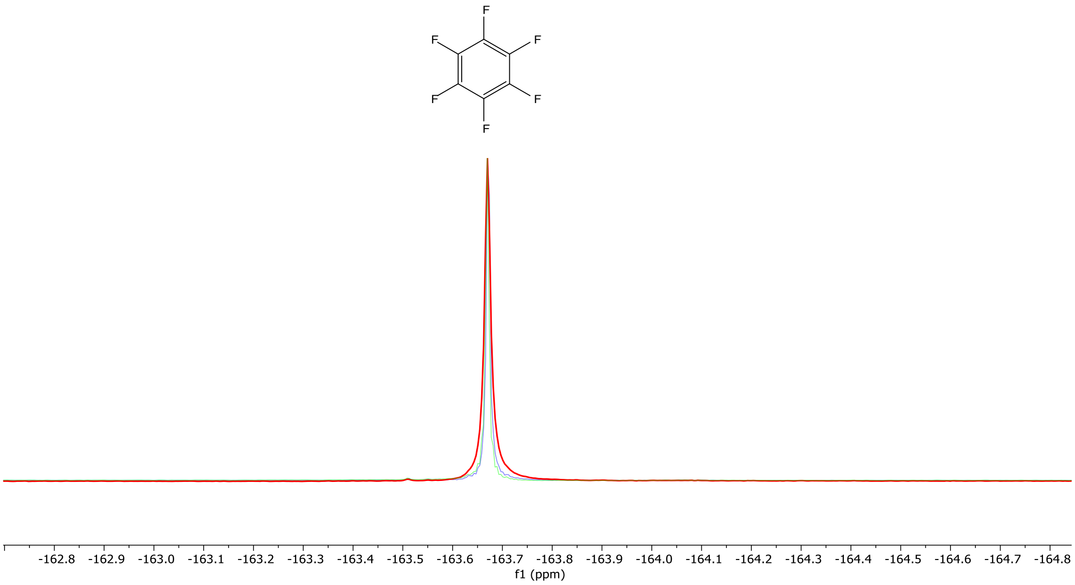

Figure S67. ^{19}F NMR spectral range of the internal standard of solutions in DMF of **22a**, **EuL1**:**22a** (1:10), **EuL2**:**22a** (1:10).

EPR study

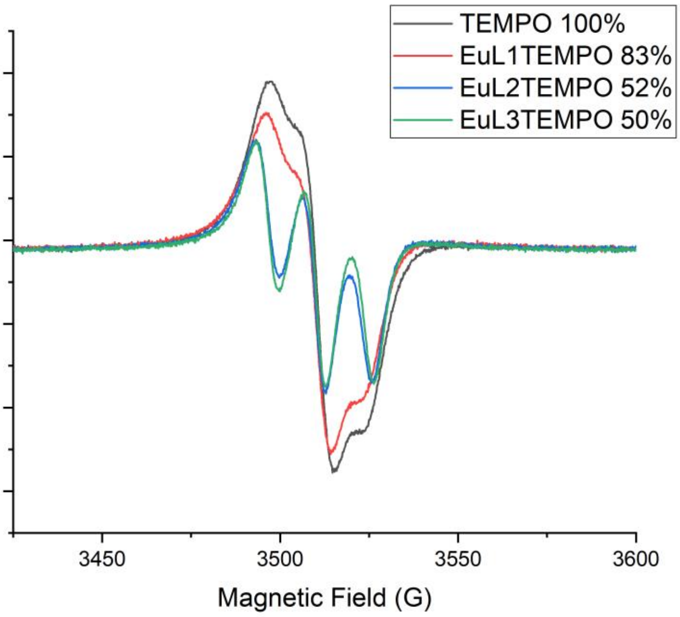

Figure S68. EPR spectra of TEMPO and **EuL1**/**EuL2**/**EuL3** containing TEMPO (1 mM in DMF) collected after 12 h irradiation with Blue LED under an Ar atmosphere. The integrated area of the radical signal (normalized to the TEMPO only sample) is shown in the figure. The presence of **EuL** is quenching the TEMPO radical, especially for **EuL2** and **EuL3**. EPR parameters: microwave power 2 µW, modulation amplitude 1 G, room temperature.

Note: All the spectra contain an EPR signal from an organic radical that fits with a broadened signal from the TEMPO radical. This broadening could potentially be due to the solvent or due to the concentration of TEMPO.

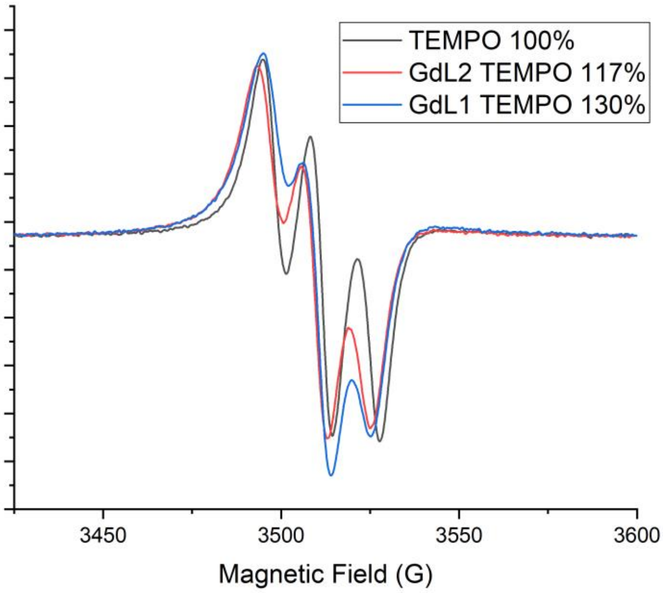

Figure S69. EPR spectra of TEMPO and **GdL1**/**GdL2** containing TEMPO (1 mM in DMF) collected after 12 h irradiation with Blue LED under an Ar atmopsphere. The integrated area of the radical signal (normalized to the TEMPO only sample) is shown in the figure. The presence of **GdL** is not quenching the TEMPO radical, the increased integrated area is likely caused by the presence of an additional very broad EPR-signal in the samples with **GdL** which affects the integration. EPR parameters: microwave power 2 μW, modulation amplitude 1 G, room temperature.

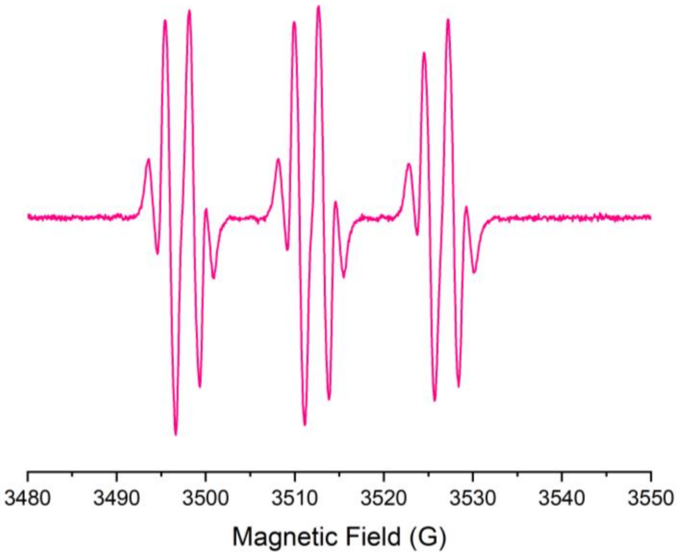

Figure S70. EPR spectrum of a solution of **EuL2** + N-tert-butyl-α-phenylnitrone (PBN) (1 mM in DMF) collected after 10 h irradiation with Blue LED under an Ar atmosphere showing the

formation of a N-based radical, EPR parameters: microwave power 2 mW, modulation amplitude 0.5 G, room temperature.

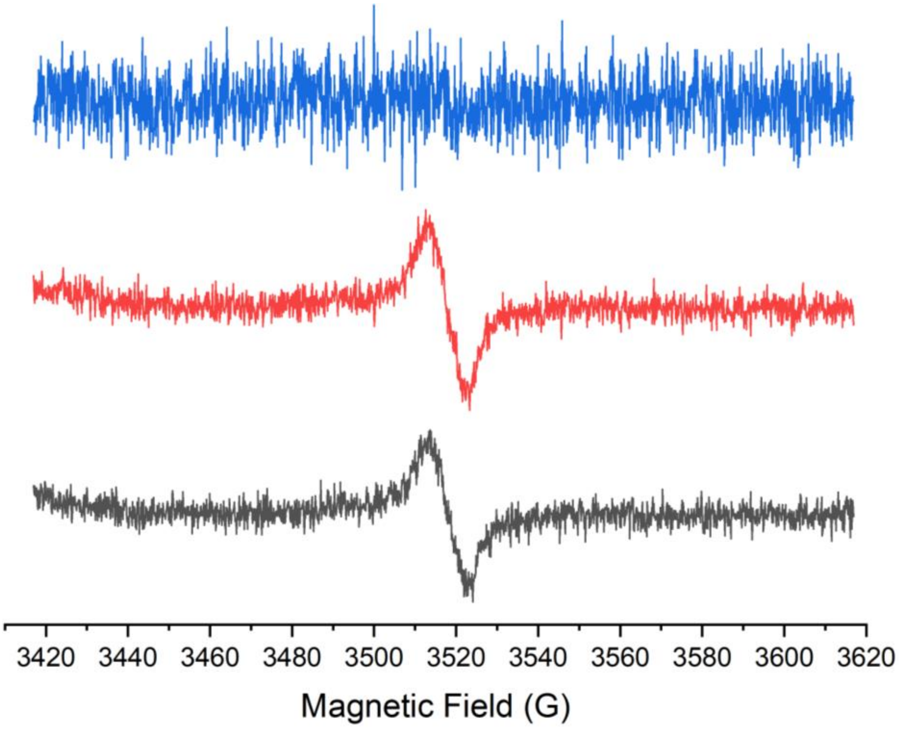

Figure S71. EPR spectra of **EuL2** before illumination (blue), during illumination with blue LED (red) and after illumination was stopped (black). The stable organic radical formed has a g-value of ~2.003. EPR parameters: microwave power 2 μW, modulation amplitude 1 G, room temperature.

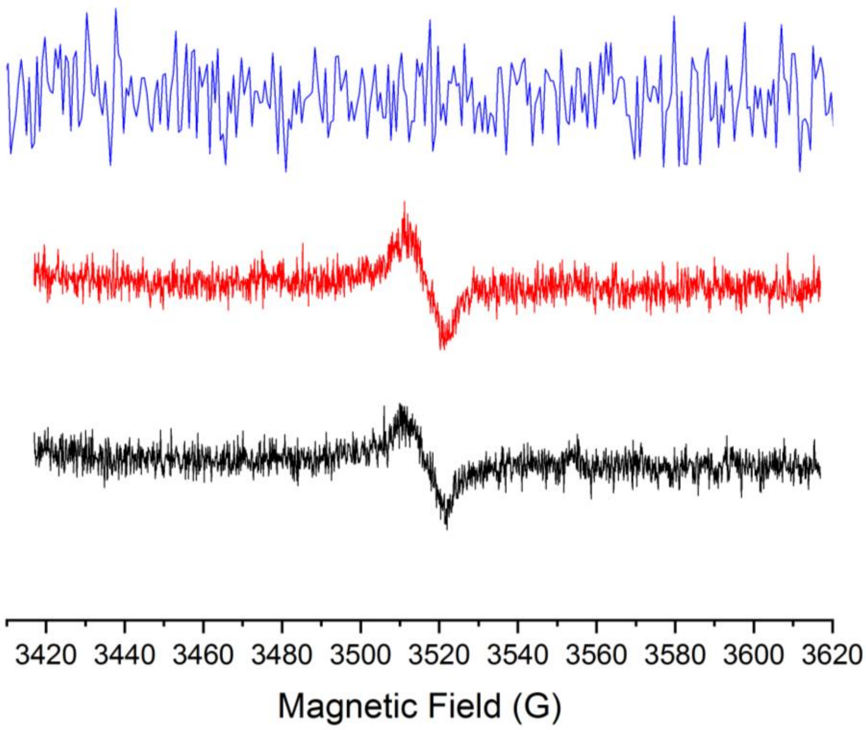

Figure S72. EPR spectra of **GdL2** before illumination (blue), during illumination with blue LED (red) and after illumination is stopped (black). The stable organic radical formed has a g-value of ~2.003. EPR parameters: microwave power 2 mW, modulation amplitude 5 G, room temperature.

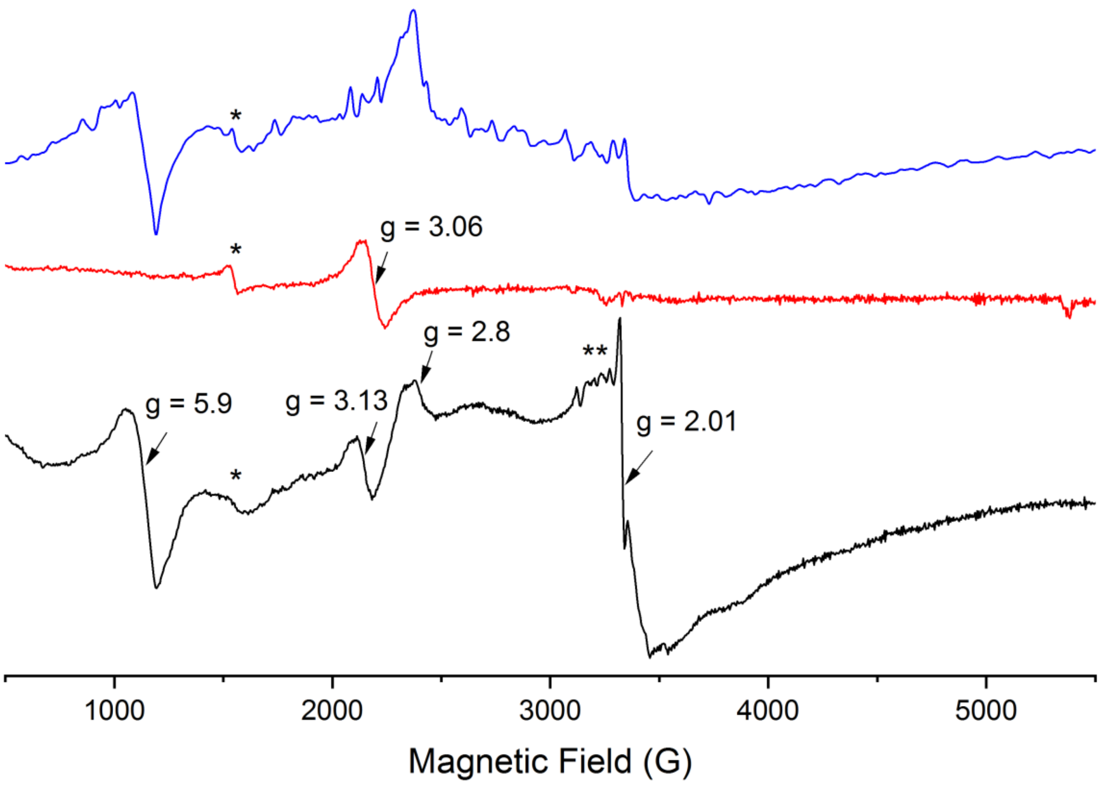

Figure S73. EPR spectra of **Gd(III)L1** (blue), **Eu(II)L1** (red) and **EuL2** after illumination (black, same as red in Figure S74). The blue spectrum is reduced by a factor of 25 for clarity. EPR parameters: microwave power 2µW (blue) or 2 mW (red and black), modulation amplitude 19.4 G, temperature: 10 K. The signal marked with * in the EPR spectra is a contamination from Fe(III), the signal marked with ** in the black spectrum is a contamination from a small amount of Mn(II). The cavity signal has been subtracted from the spectra and a baseline correction has been applied.

GCMS traces

GCMS traces for the reactions listed in Table S1.

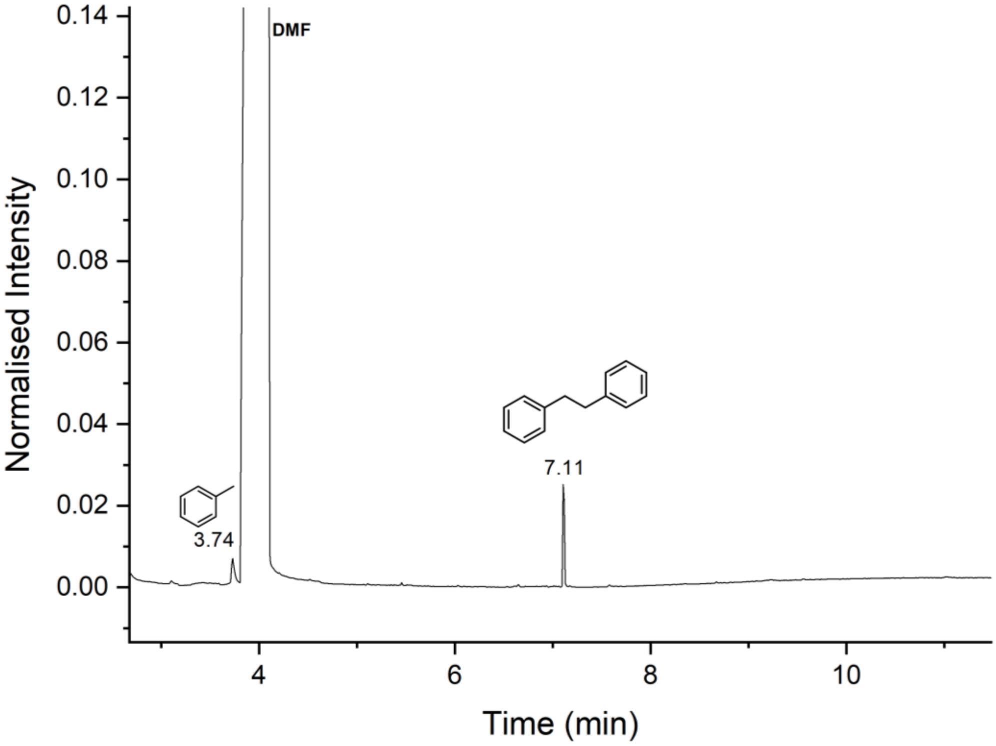

Figure S74. GC trace of the reaction mixture of **1a** described in Entry 1 (Table S1).

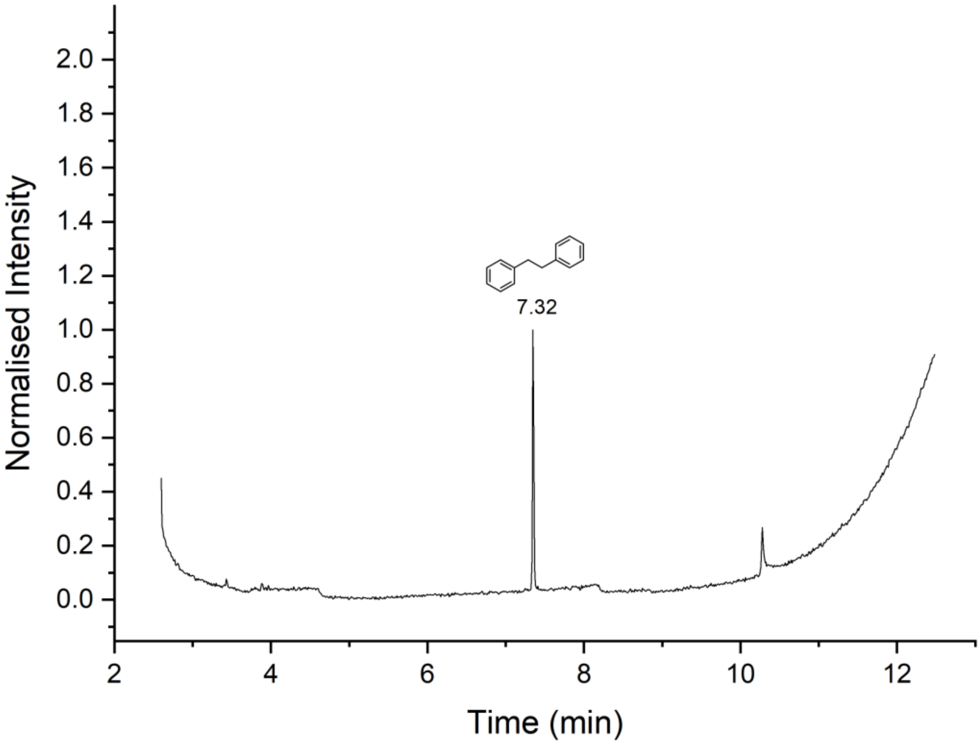

Figure S75. GC trace of the reaction mixture of **1a** described in Entry 2 (Table S1).

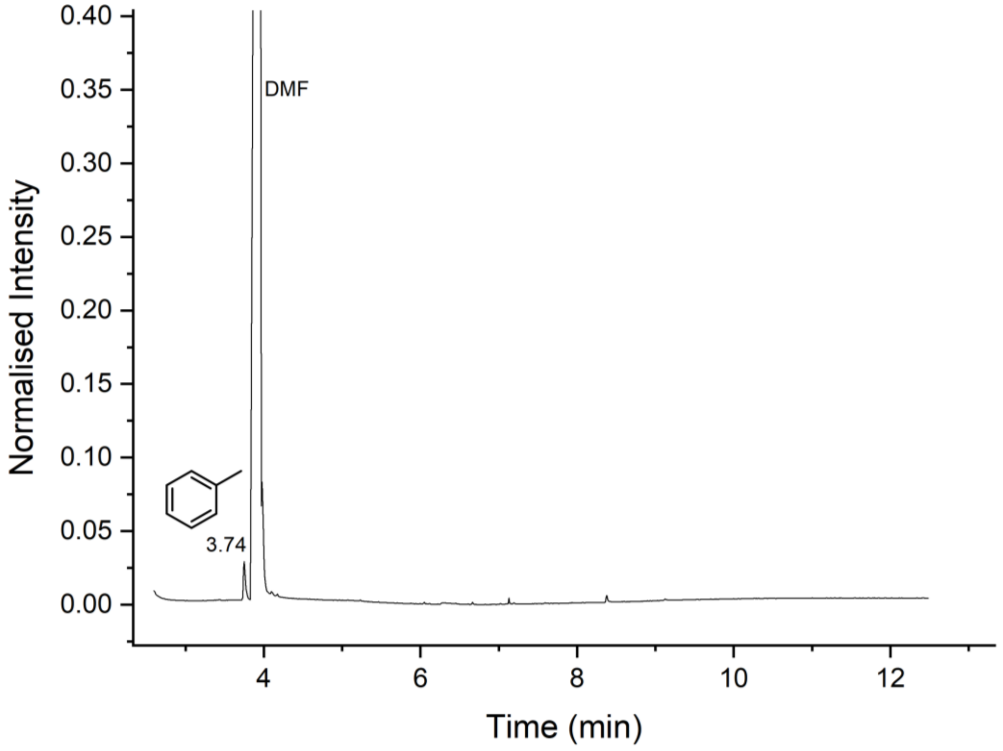

Figure S76. GC trace of the reaction mixture of **1a** described in Entry 3 (Table S1).

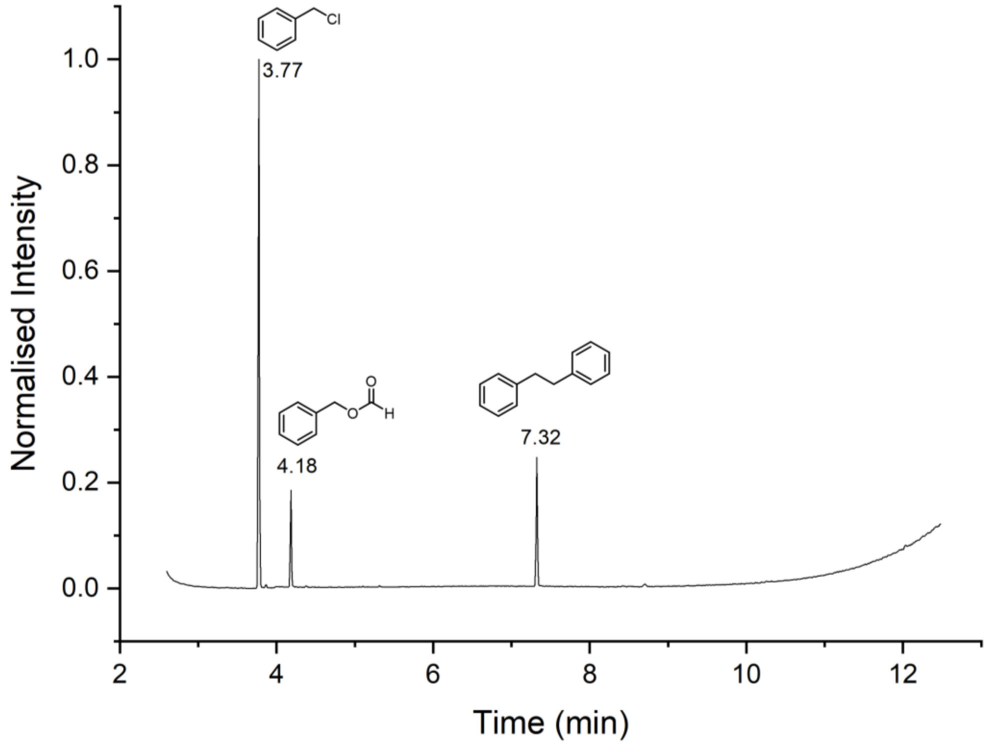

Figure S77. GC trace of the reaction mixture of **1a** described in Entry 5 (Table S1).

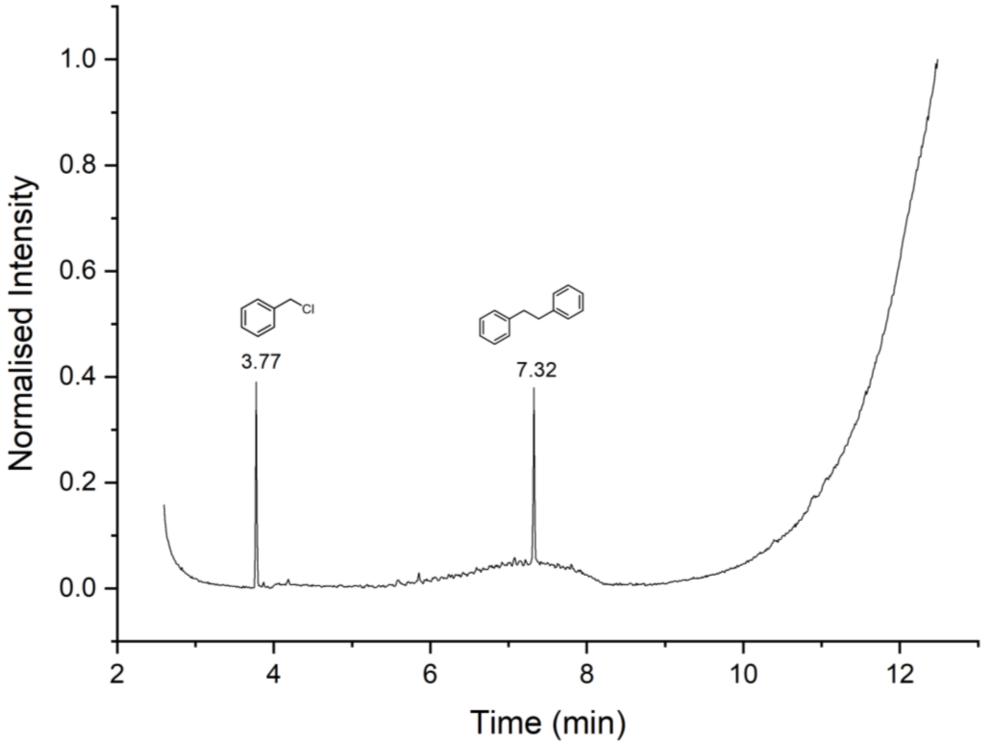

Figure S78. GC trace of the reaction mixture of **1a** described in Entry 6 (Table S1).

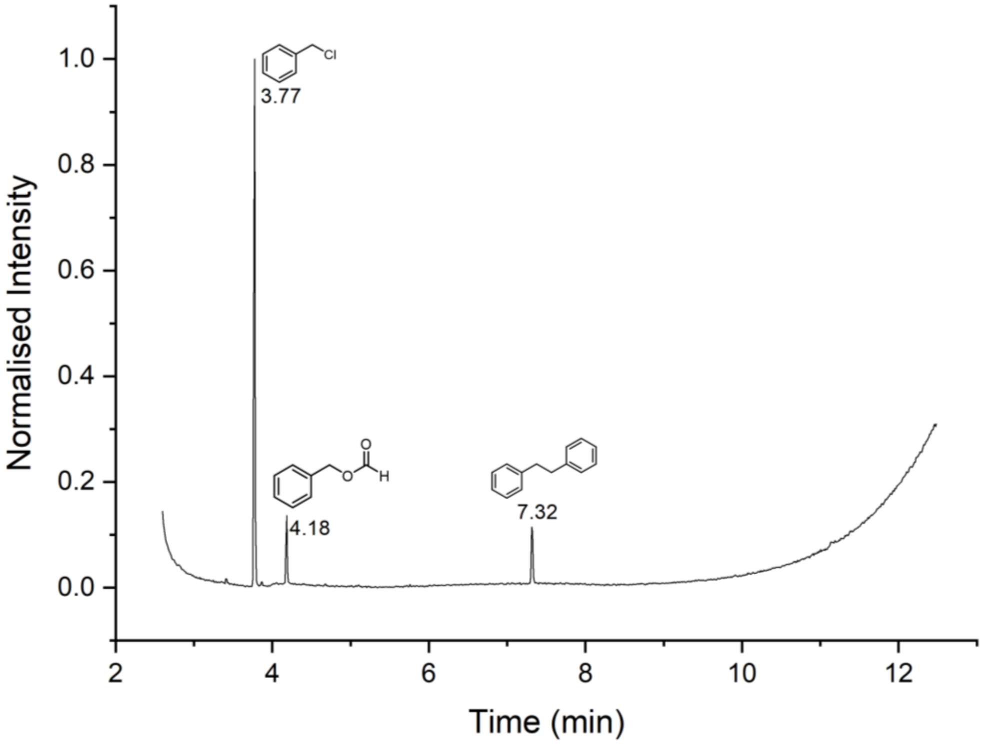

Figure S79. GC trace of the reaction mixture of **1a** described in Entry 7 (Table S1).

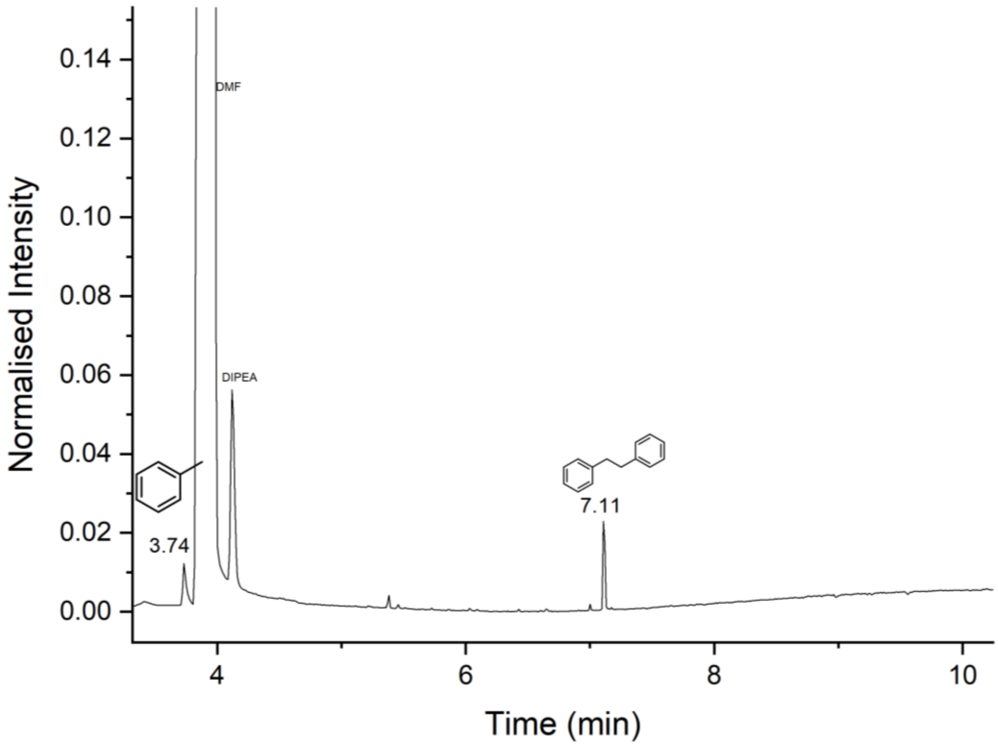

Figure S80. GC trace of the reaction mixture of **1a** described in Entry 13 (Table S1).

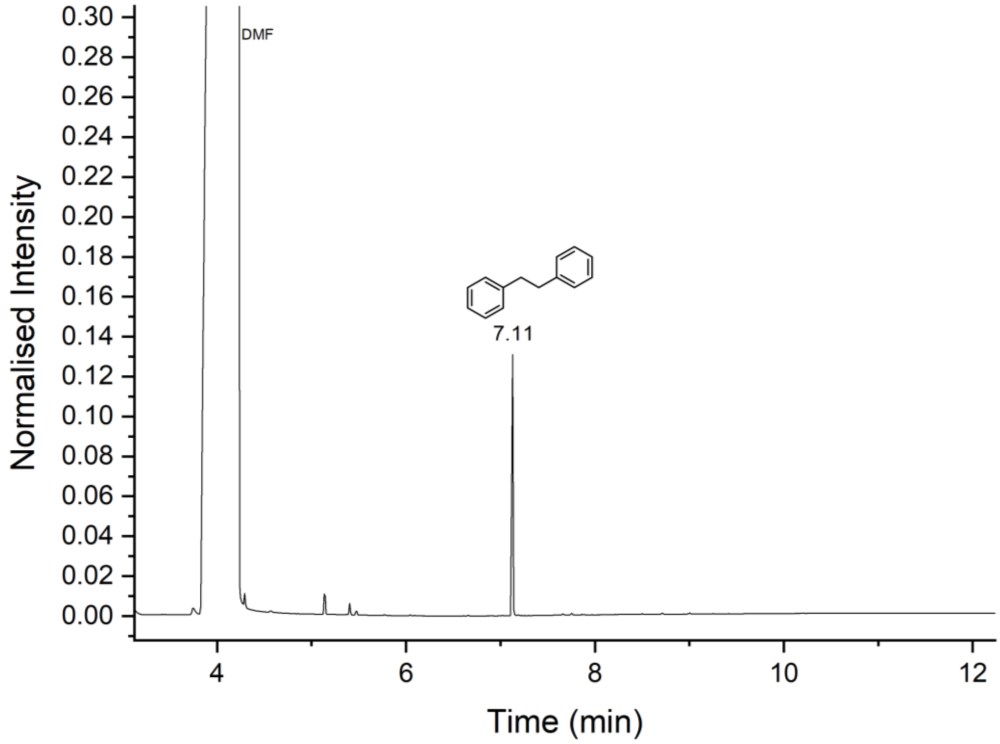

Figure S81. GC trace of the reaction mixture of **1a** described in Entry 14 (Table S1).

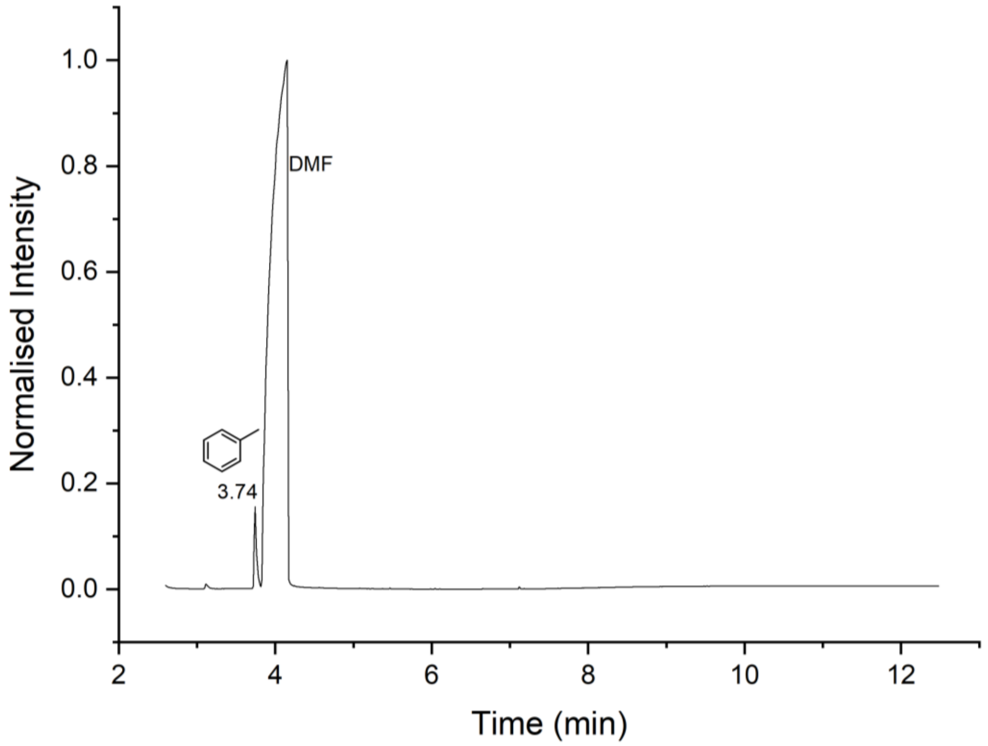

Figure S82. GC trace of the reaction mixture of **1a** described in Entry 15 (Table S1).

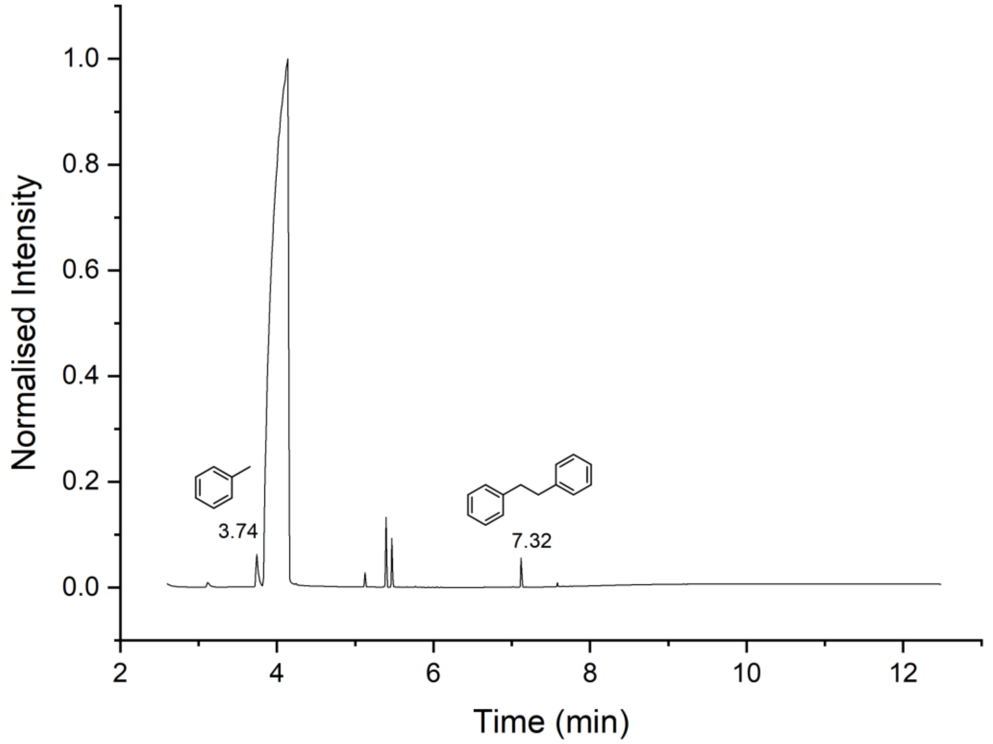

Figure S83. GC trace of the reaction mixture of **1a** described in Entry 18 (Table S1).

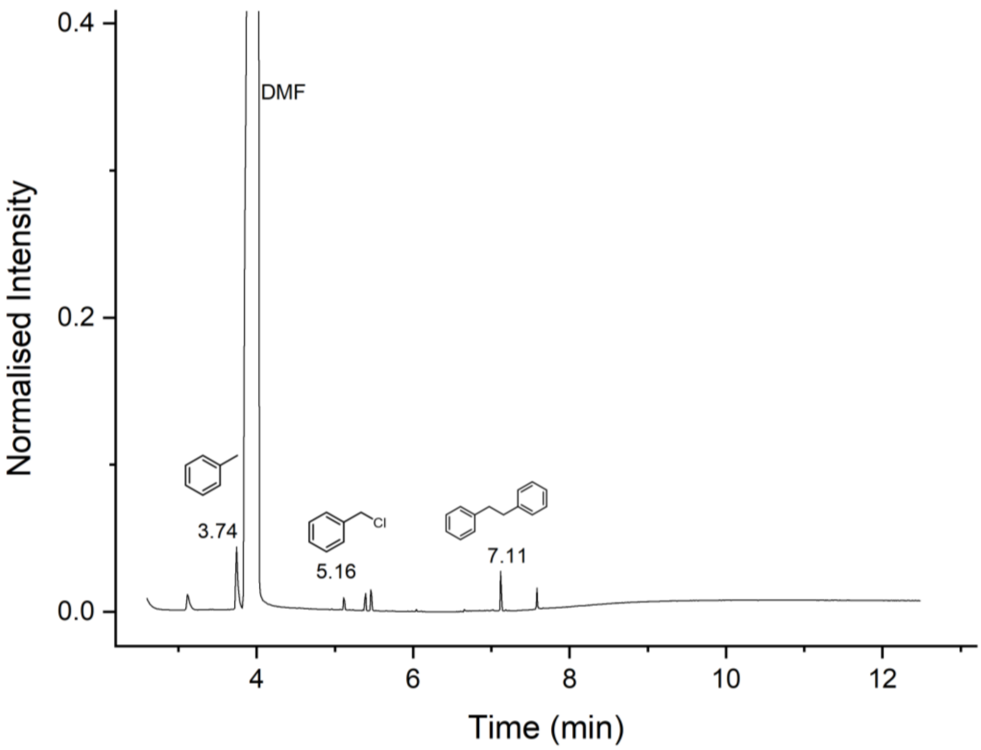

Figure S84. GC trace of the reaction mixture of **1a** described in Entry 21 (Table S1).

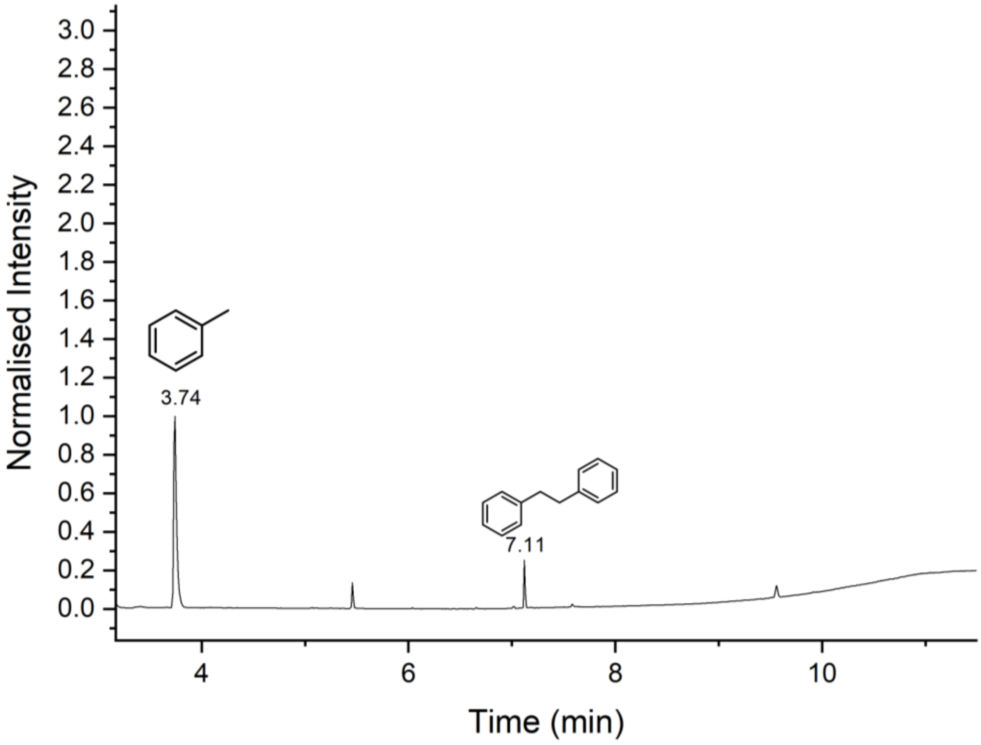

Figure S85. GC trace of the reaction mixture of **1a** described in Entry 22 (Table S1).

GCMS traces of the reactions listed in Table S2.

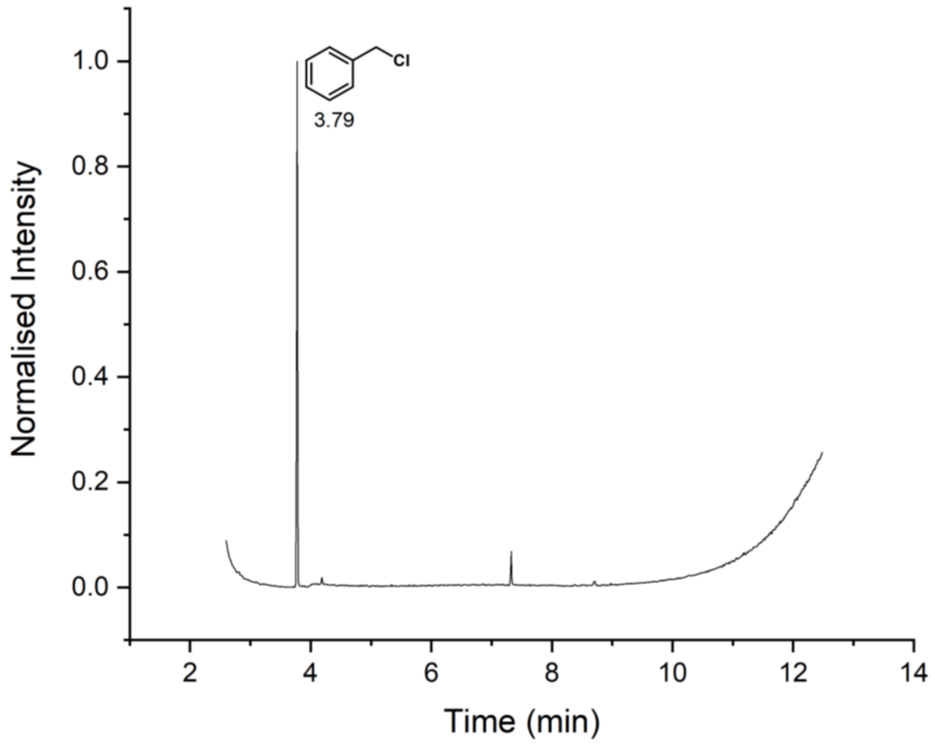

Figure S86. GC trace of the reaction mixture of **1a** described in Entry 1 (Table S2).

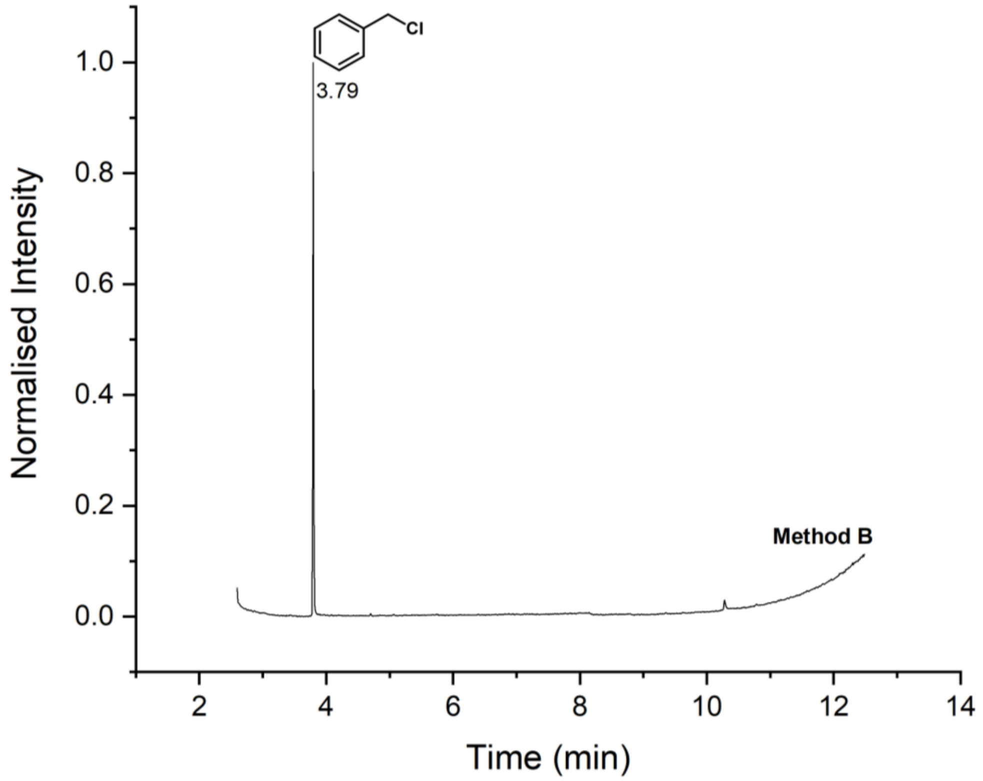

Figure S87. GC trace of the reaction mixture of **1a** described in Entry 2 (GdL2, Table S2).

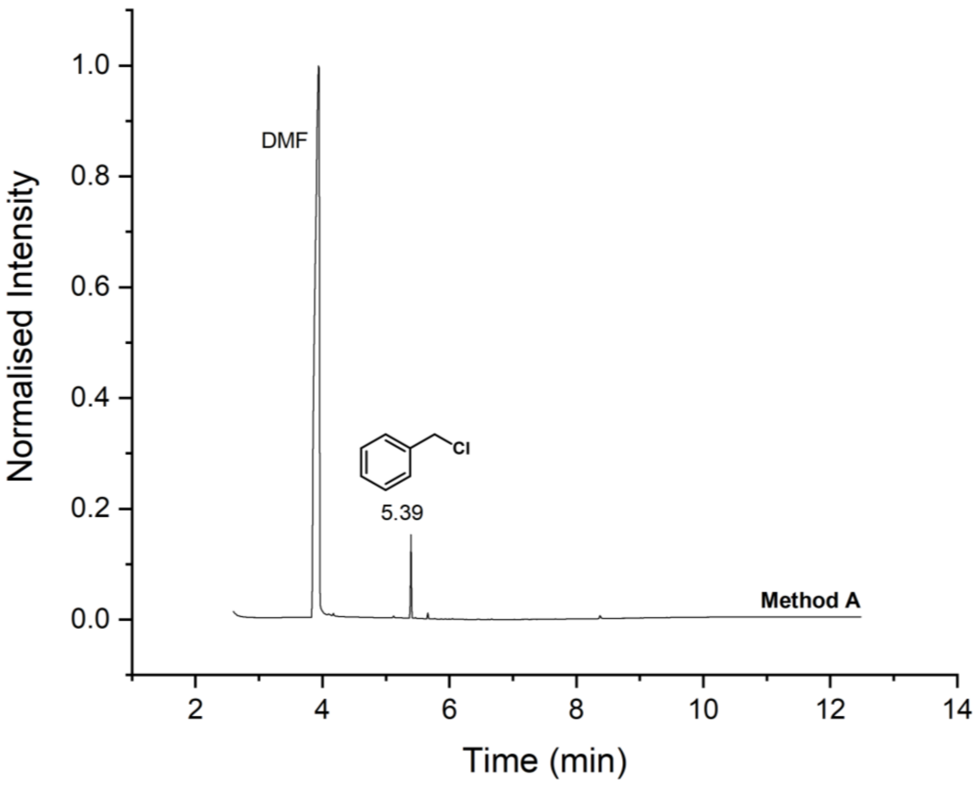

Figure S88. GC trace of the reaction mixtures **1a** described in Entry2 (GdL3, Table S2).

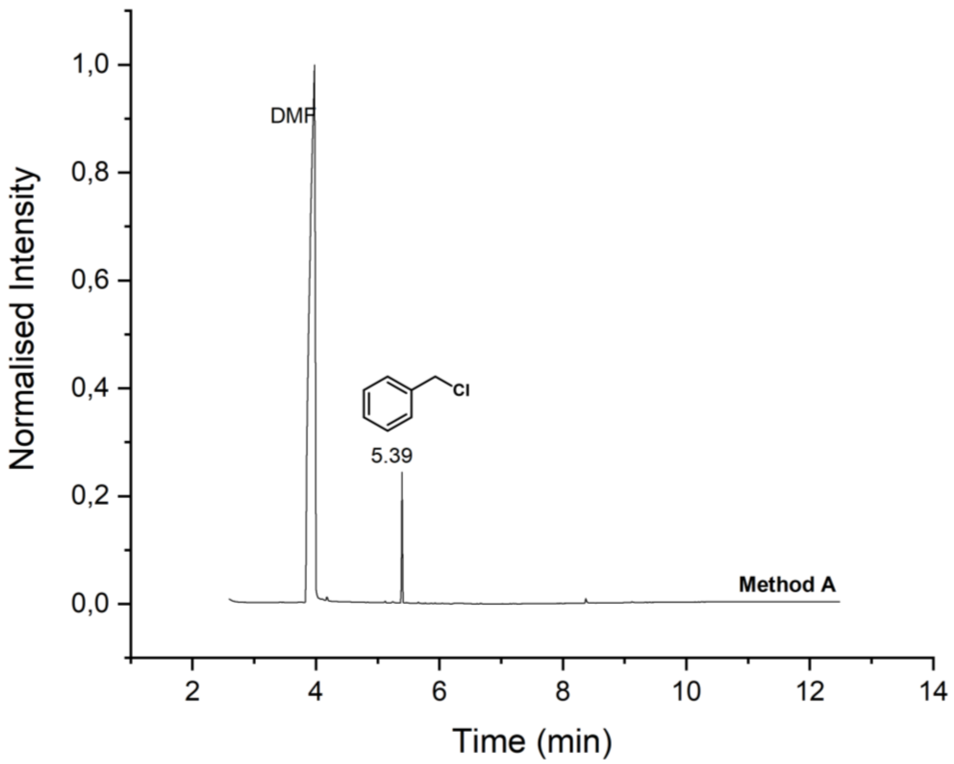

Figure S89. GC trace of the reaction mixtures **1a** described in Entry 2 (GdL1, Table S2).

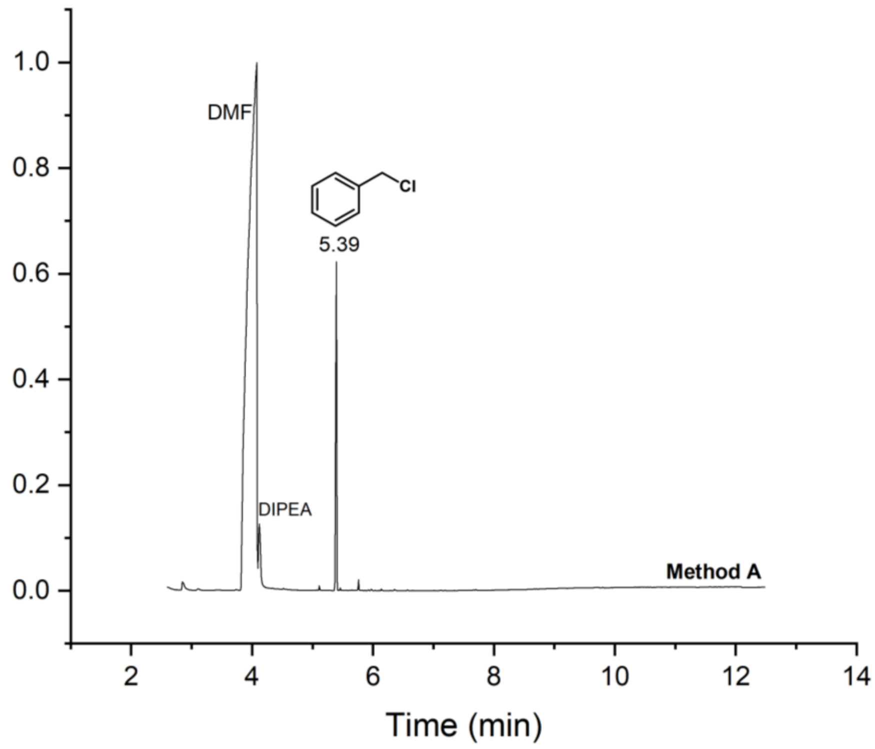

Figure S90. GC trace of the reaction mixtures **1a** described in Entry 3 (Table S2).

GCMS traces of the reaction mixtures of 2b–43b.

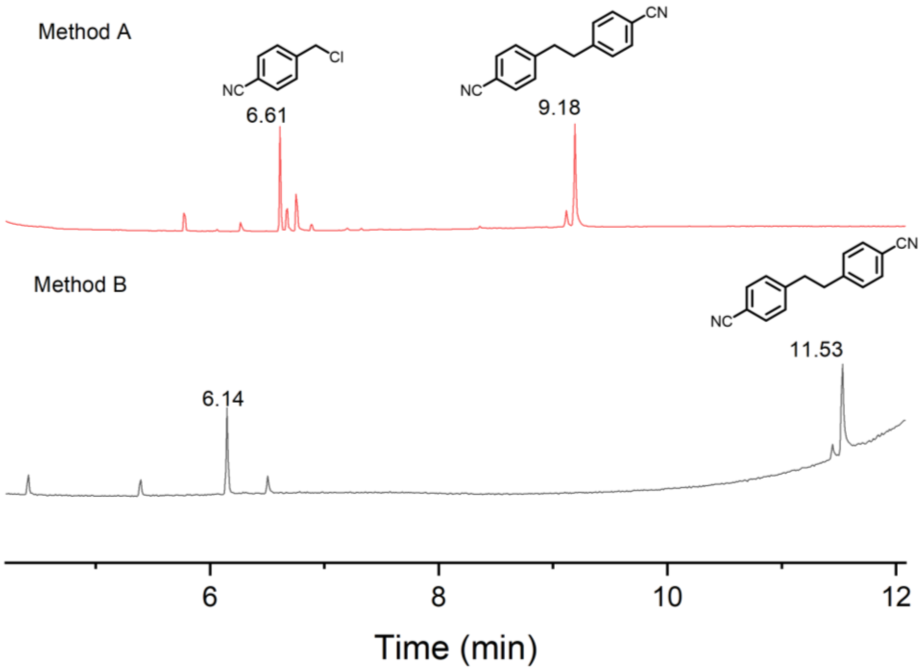

Figure S91. GC chromatograms of the reaction mixtures (**2b**) described in Entries 1 (grey) and 2 (red).

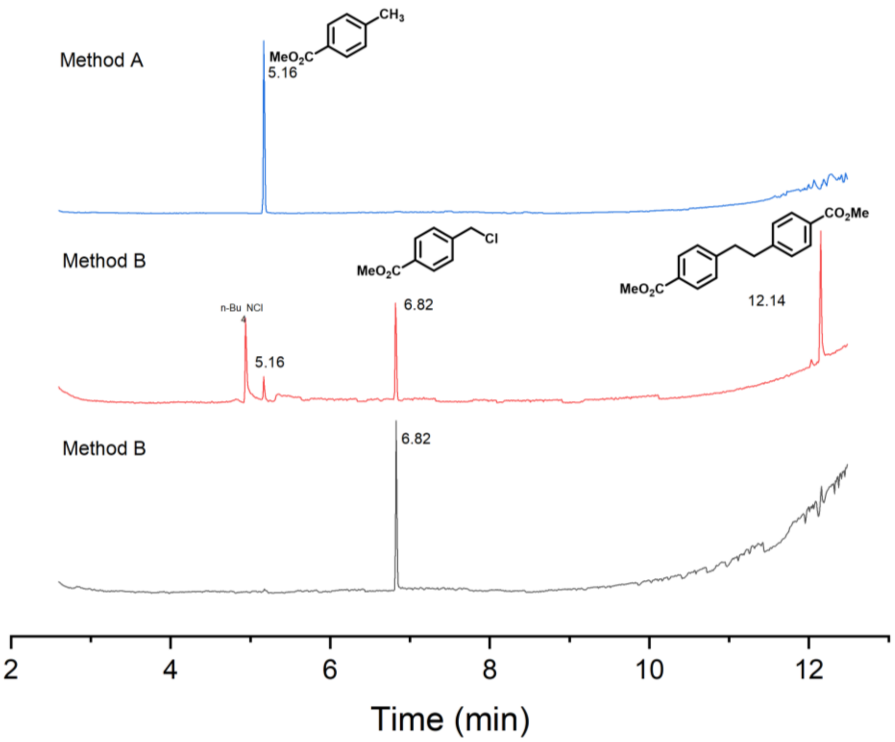

Figure S92. GC chromatograms of the reaction mixtures (**3b**) described in Entries 1 (grey) 2 (red) and 3 (blue).

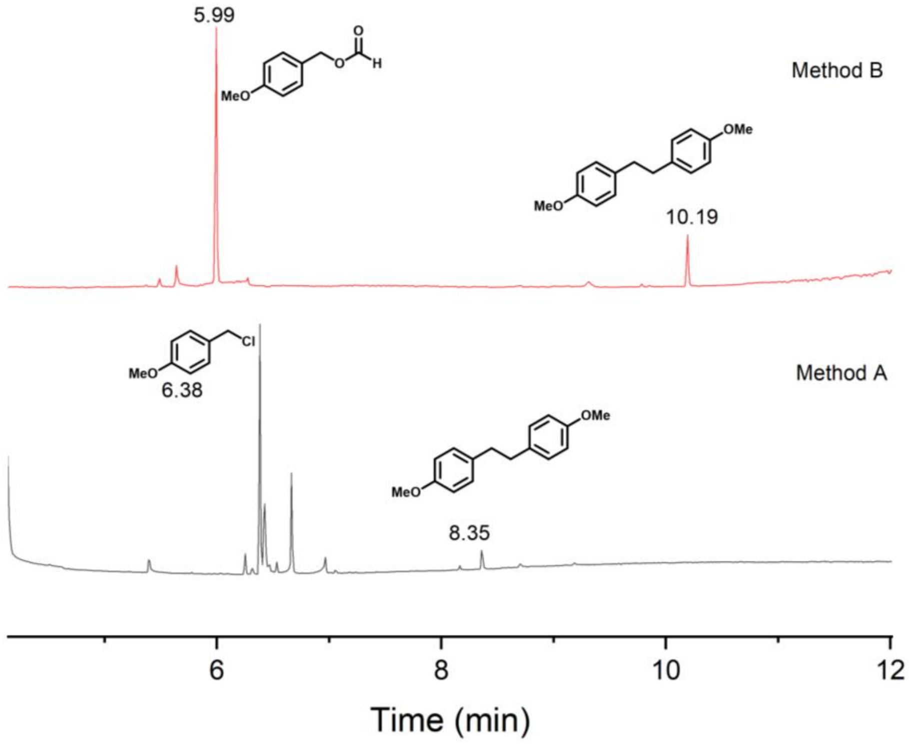

Figure S93. GC chromatograms of the reaction mixtures of **4b** described in Entries 2 (grey) and 1 (red).

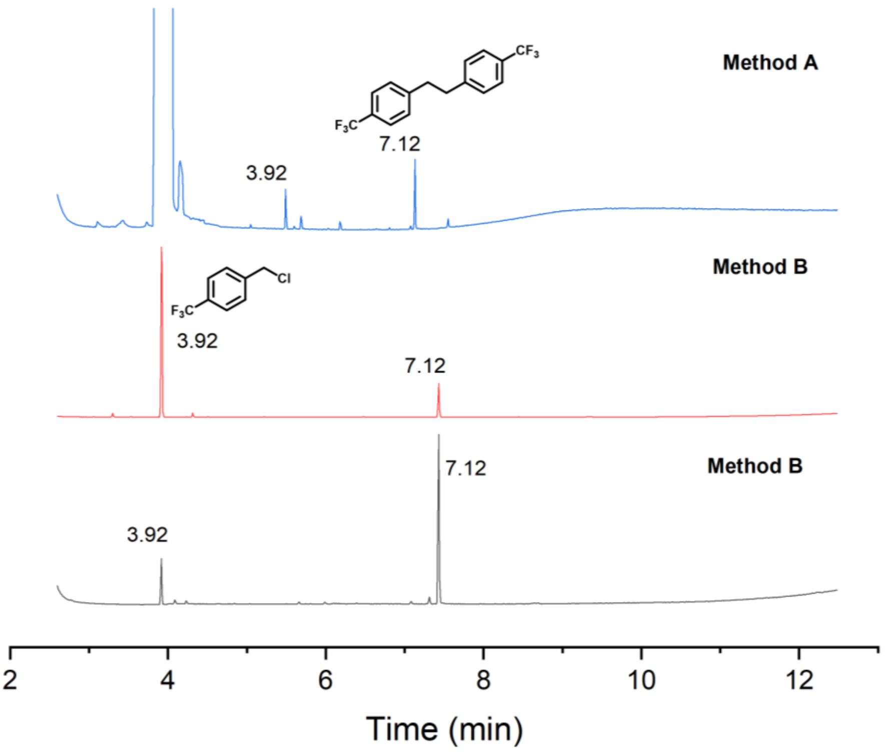

Figure S94. GC chromatograms of the reaction mixtures of **5a** described in Entries 3 (grey) 2, (red) and 1 (blue).

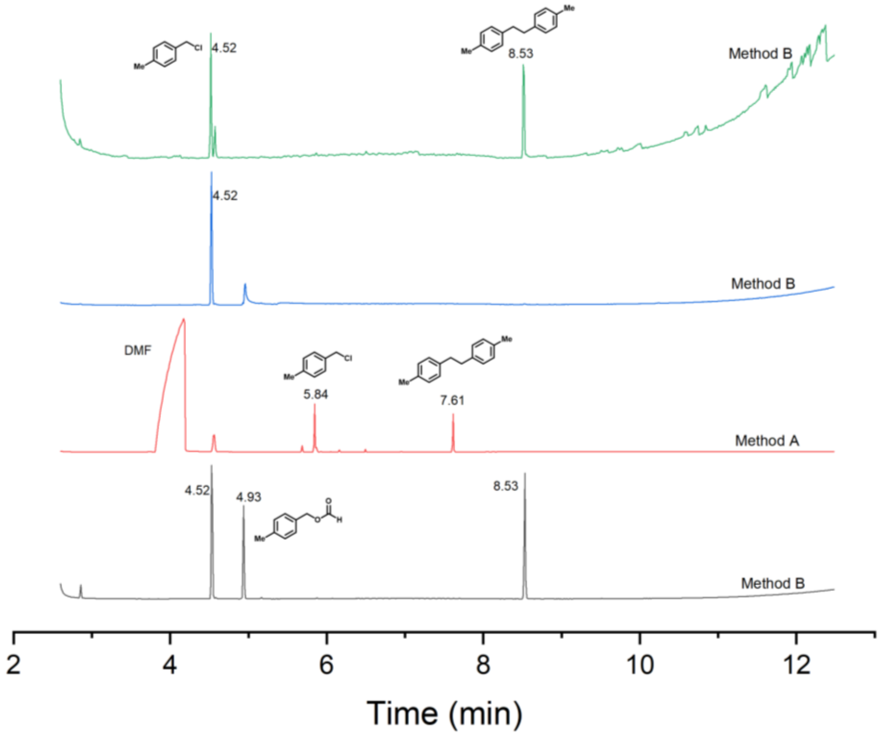

Figure S95. GC chromatograms of the reaction mixtures of **6a** described in Entries 1 (grey) 2 (red), 3 (blue) and 4 (green).

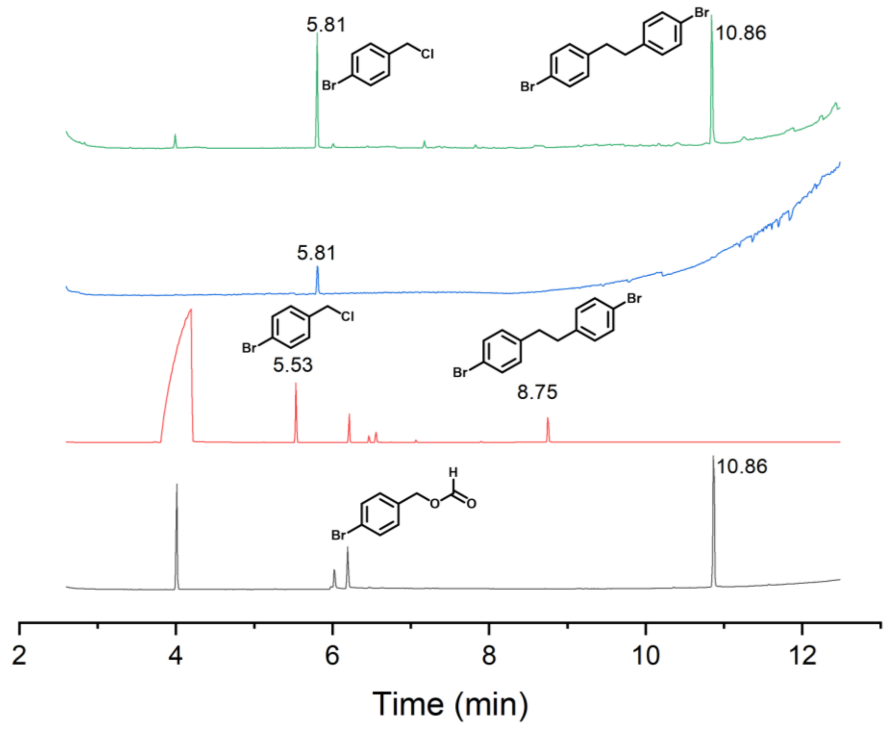

Figure S96. GC chromatograms of the reaction mixtures of **7a** described in Entries 1 (grey) 2 (red), 3 (blue) and 4 (green).

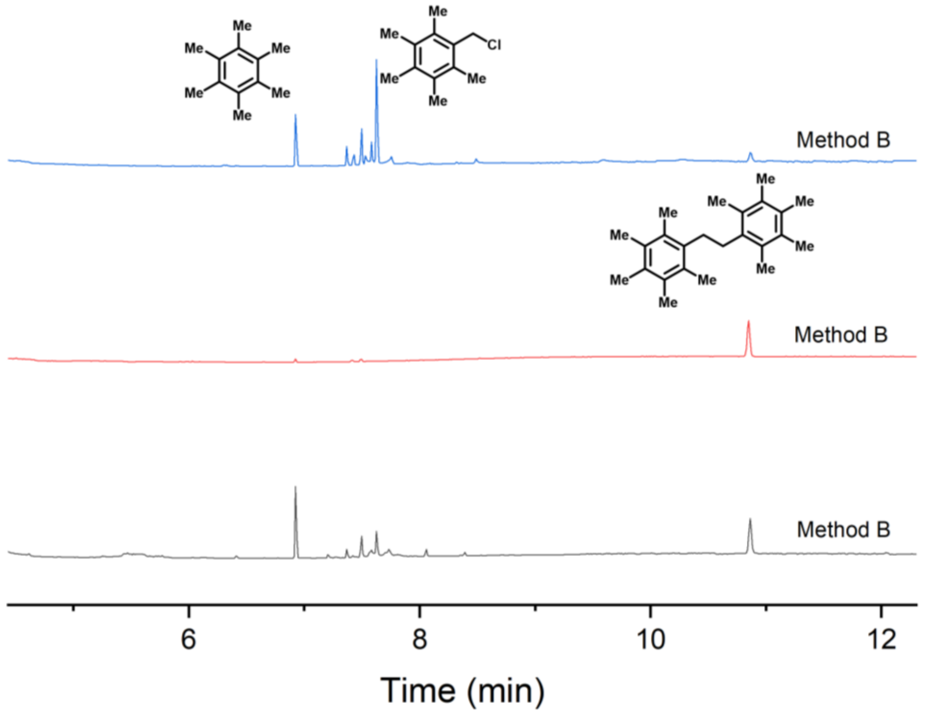

Figure S97. GC chromatograms of the reaction mixtures of **8a** described in Entries 1 (grey) 2, (red) and 3 (blue).

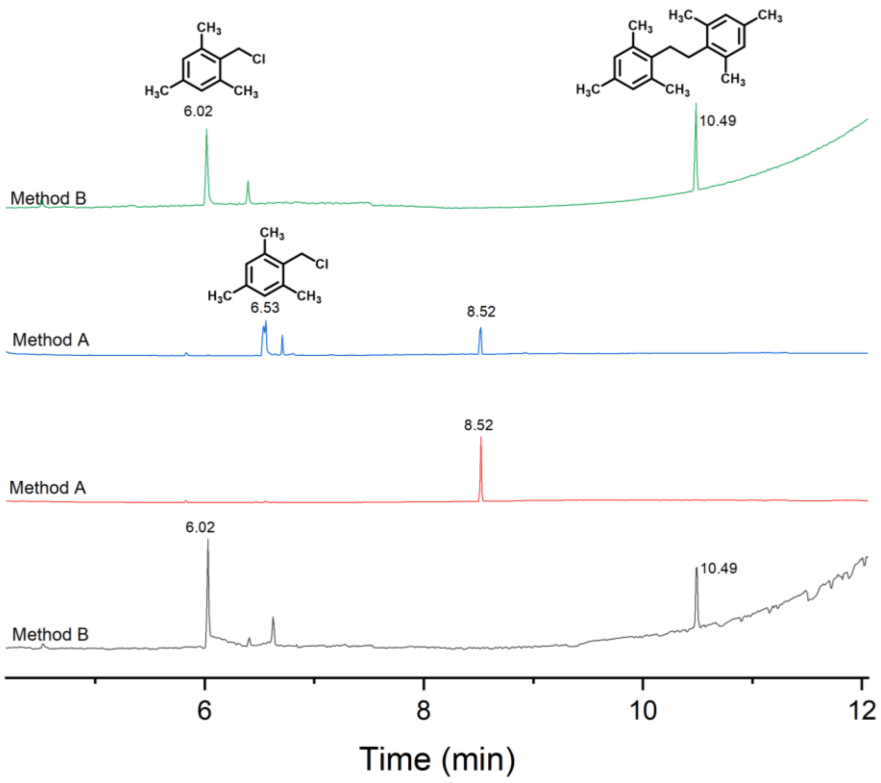

Figure S98. GC chromatograms of the reaction mixtures of **9a** described in Entries 1 (grey) 2, (red), 4 (blue) and 3 (green).

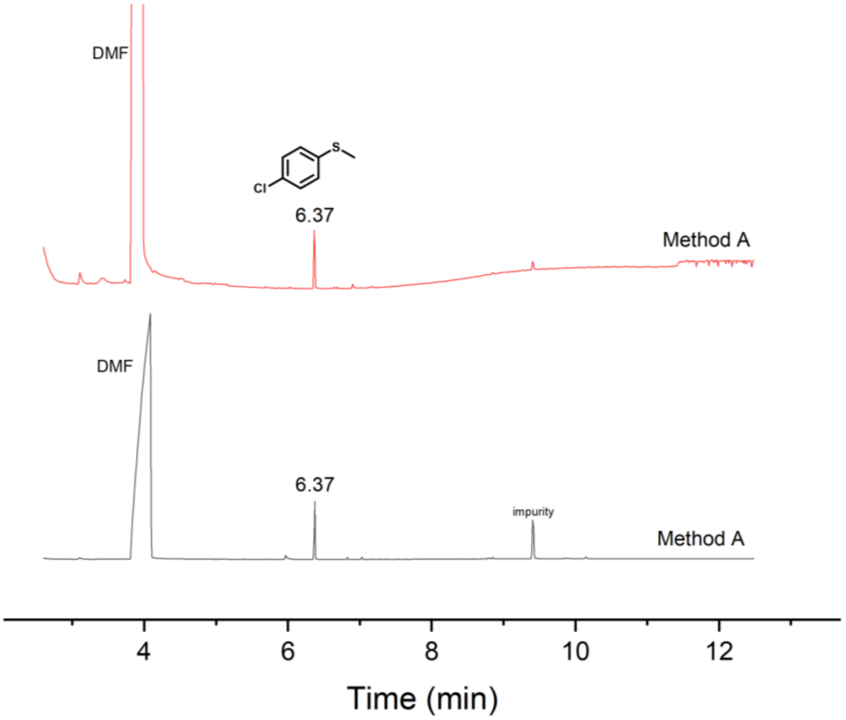

Figure S99. GC chromatograms of the reaction mixtures of **10a** described in Entries 1 (grey) and 2 (red).

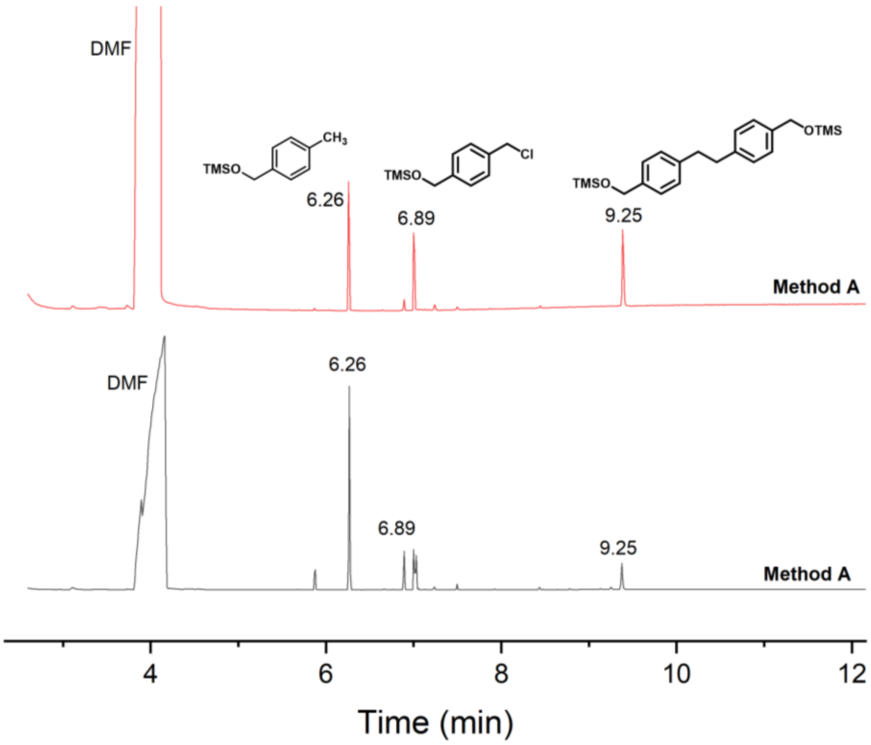

Figure S100. GC chromatograms of the reaction mixtures of **12a** described in Entries 1 (grey) and 2 (red).

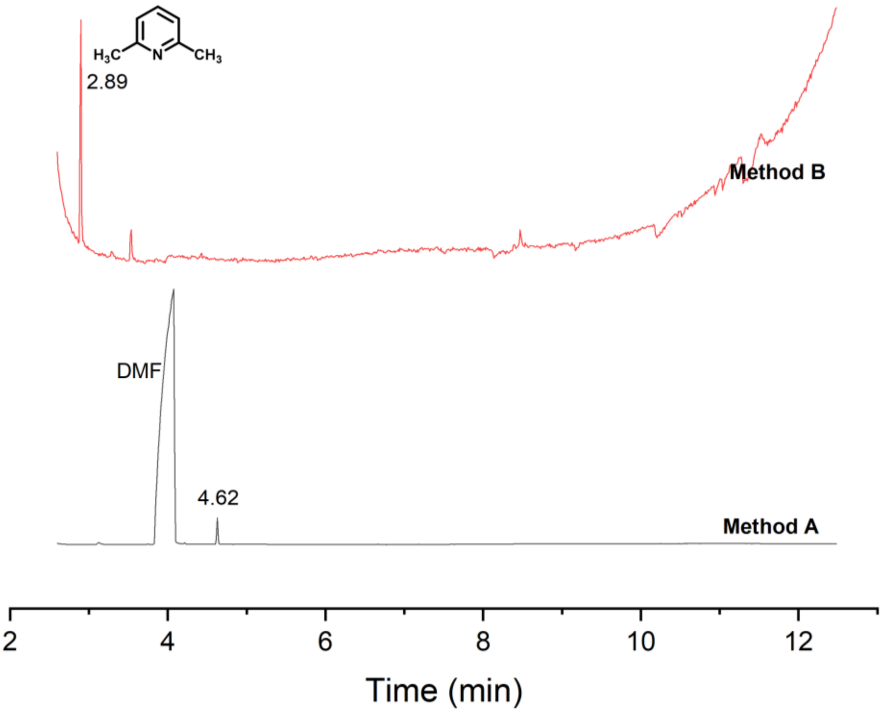

Figure S101. GC chromatograms of the reaction mixtures of **13a** described in Entries 1 (grey) and 2 (red).

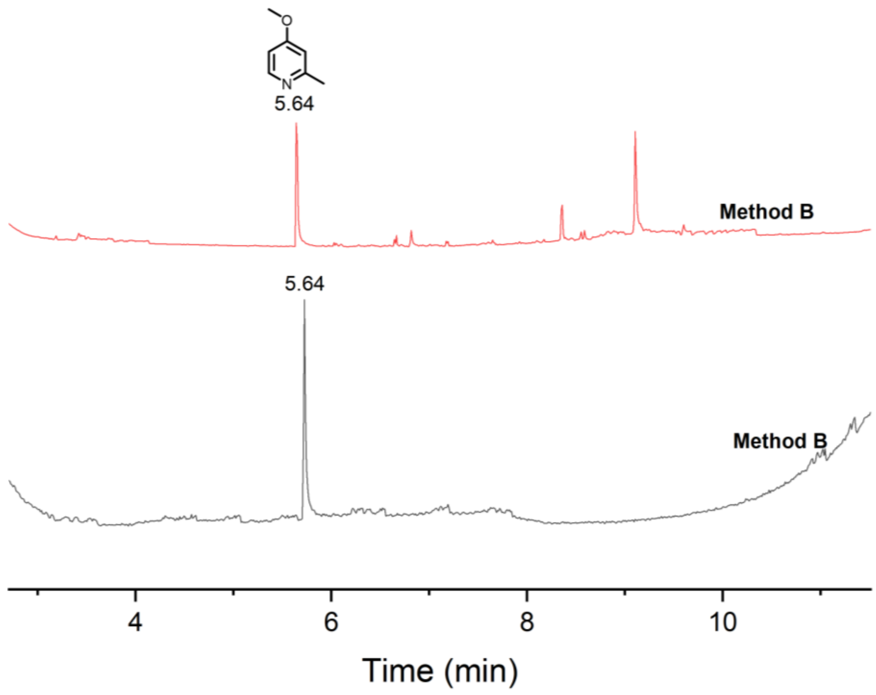

Figure S102. GC chromatograms of the reaction mixtures of **14a** described in Entries 1 (grey) and 2 (red).

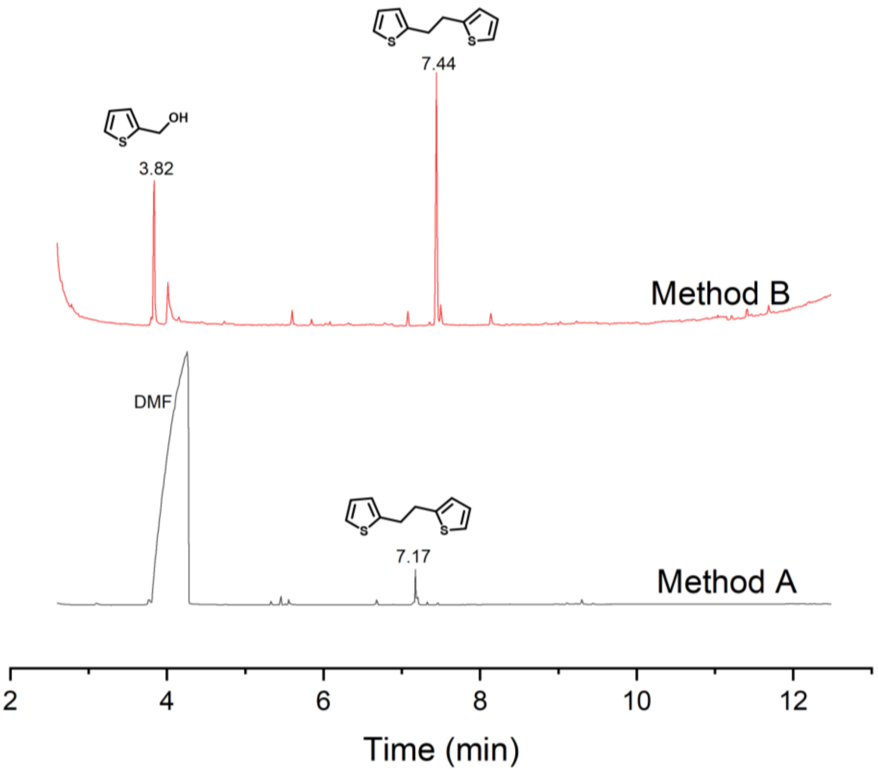

Figure S103. GC chromatograms of the reaction mixtures of **16a** described in Entries 1 (grey) and 2 (red).

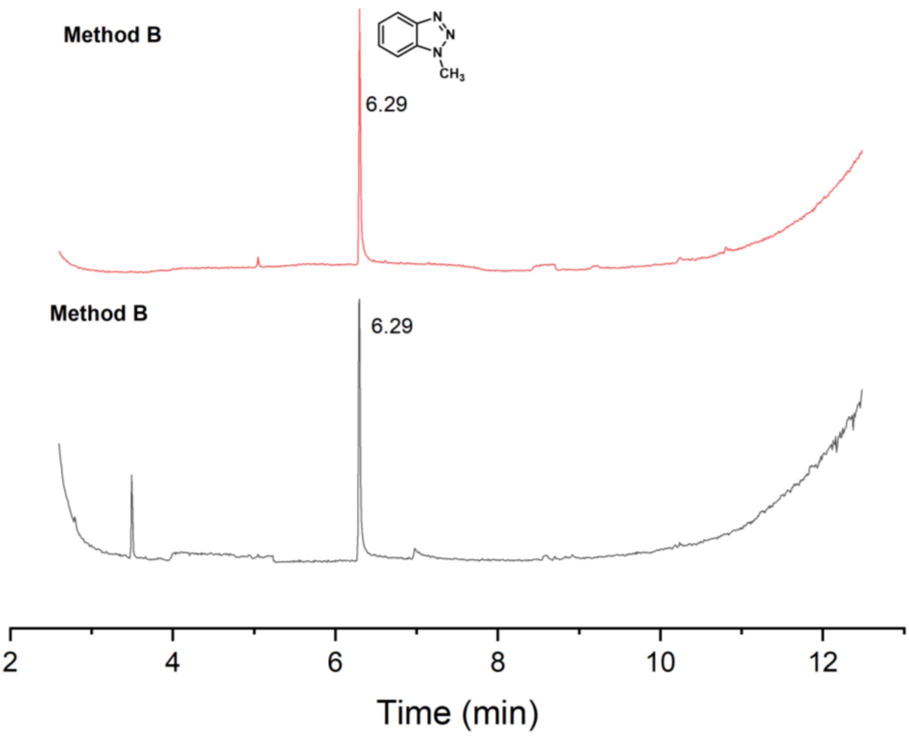

Figure S104. GC chromatograms of the reaction mixtures of **17a** described in Entries 2 (grey) and 1 (red).

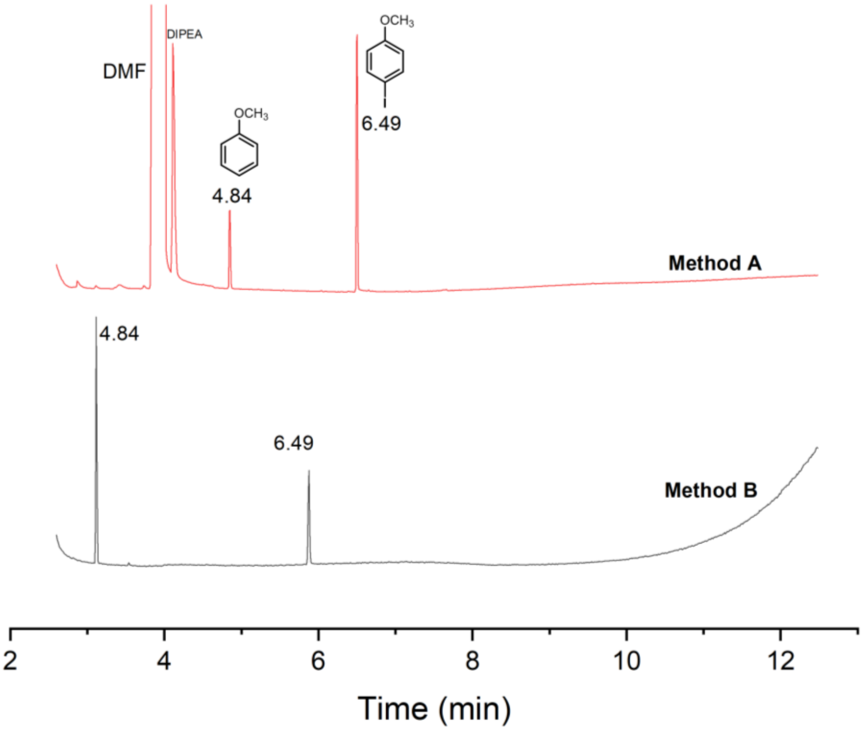

Figure S105. GC chromatograms of the reaction mixtures of **20a** described in Entries 1 (grey) and 2 (red).

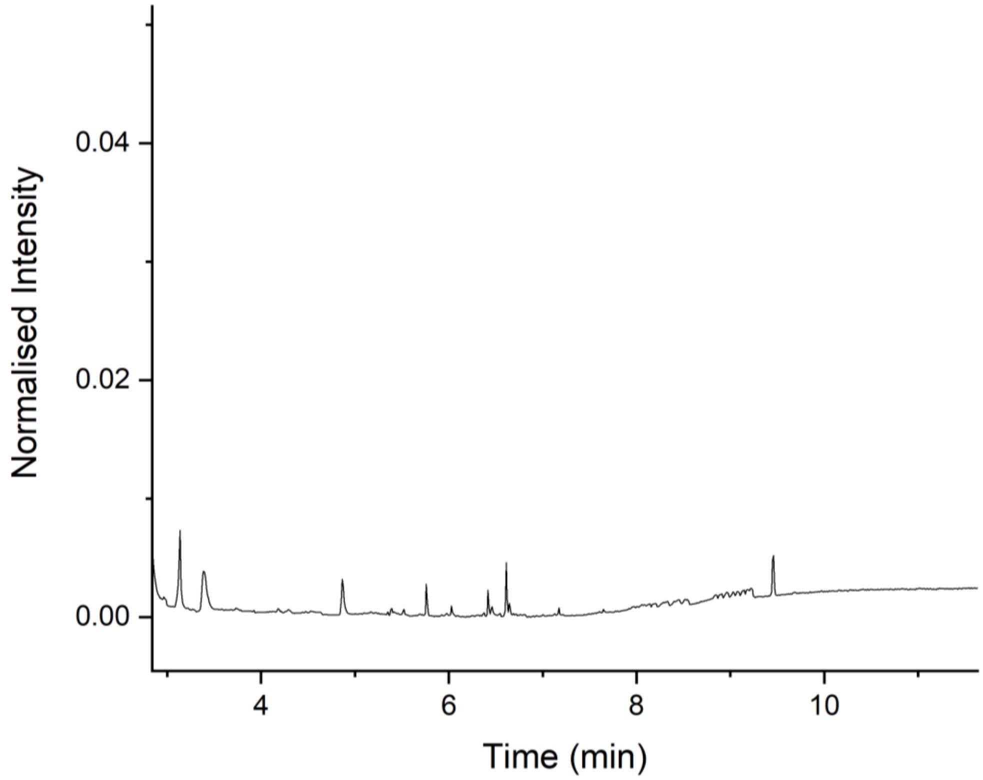

Figure S106. GC chromatogram of the reaction mixture of **21a** with stoichiometric SmI_2.

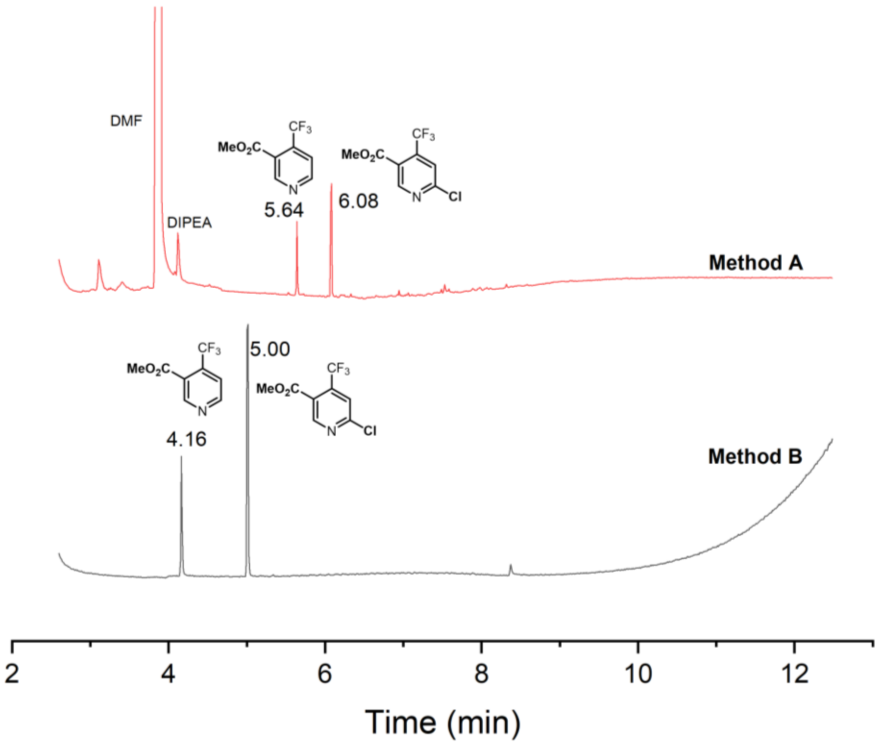

Figure S107. GC chromatograms of the reaction mixtures of **22a** described in Entries 1 (grey) and 2 (red).

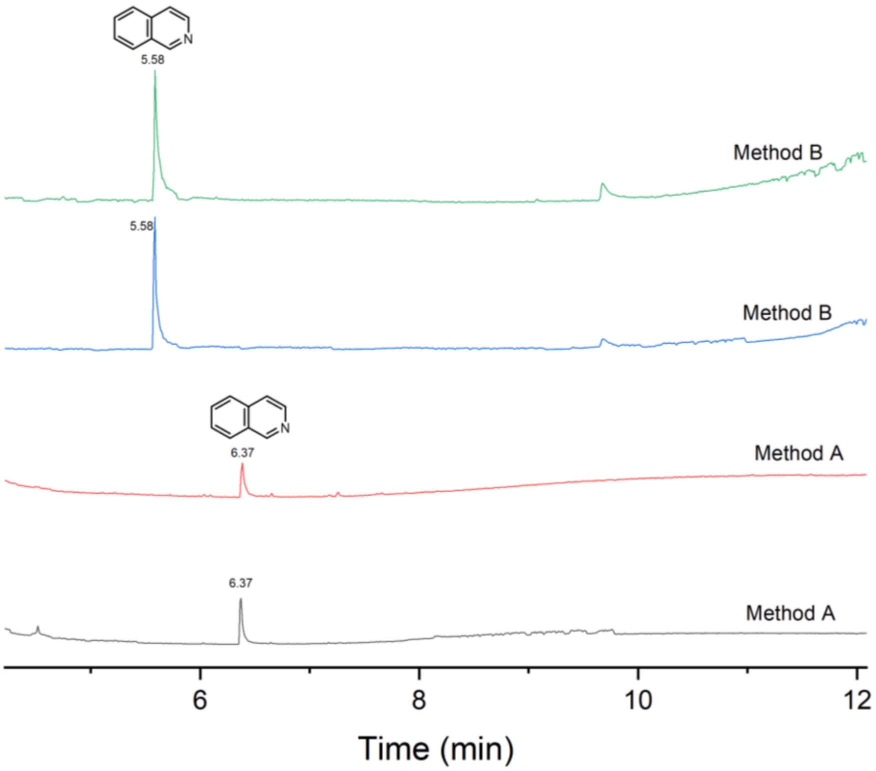

Figure S108. GC chromatograms of the reaction mixtures of **24a** described in Entries 1 (grey), 2 (red), 3 (blue) and 4 (green).

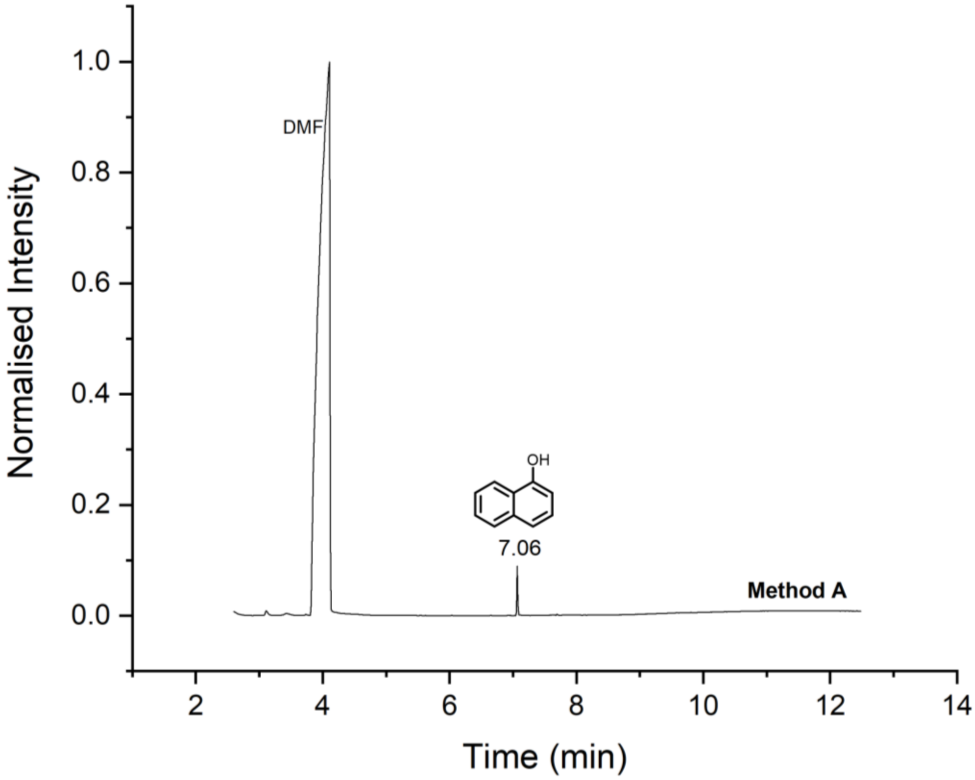

Figure S109. GC chromatograms of the reaction mixtures of **29a**.

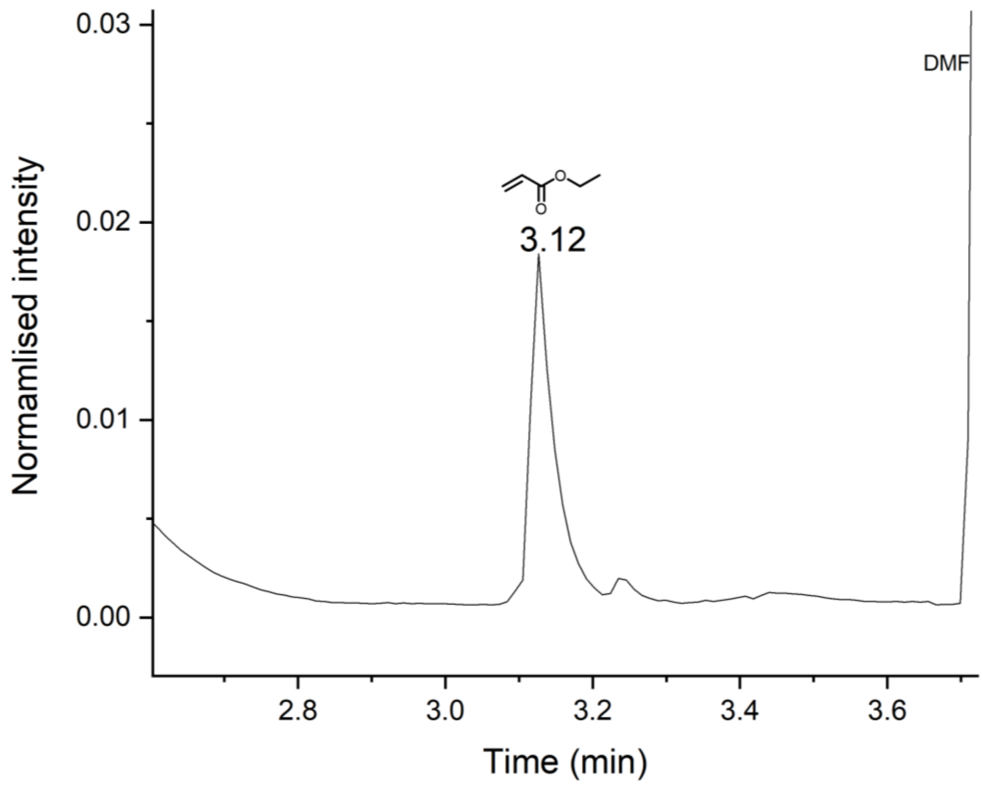

Figure S110. GC chromatograms of the reaction mixtures of **33a**.

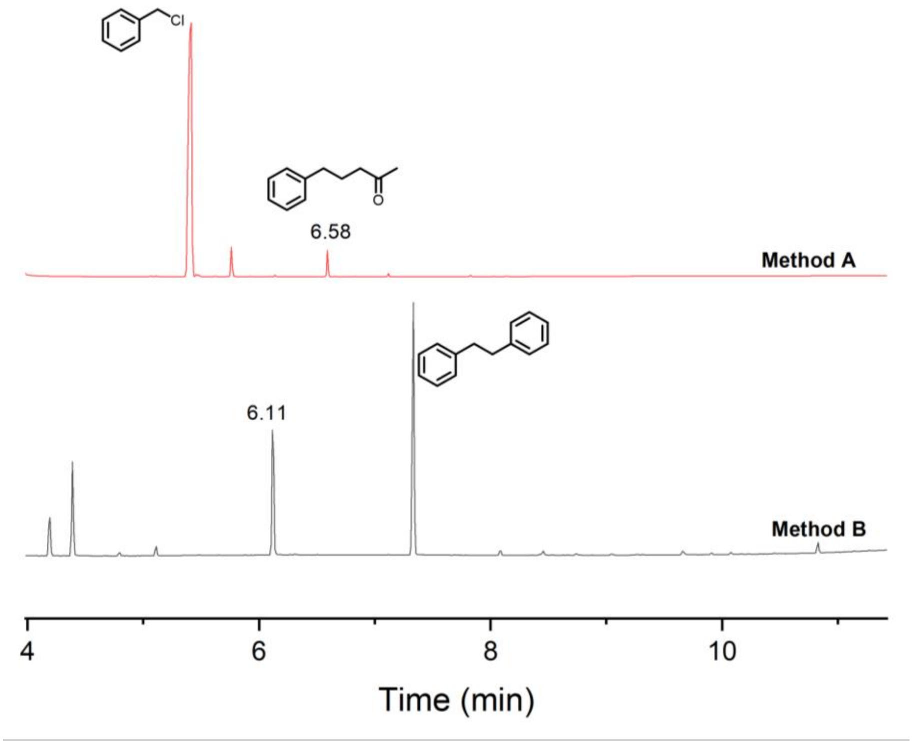

Figure S111. GC chromatograms of the reaction mixtures of **38a** described in Entries 1 (grey) and 2 (red).

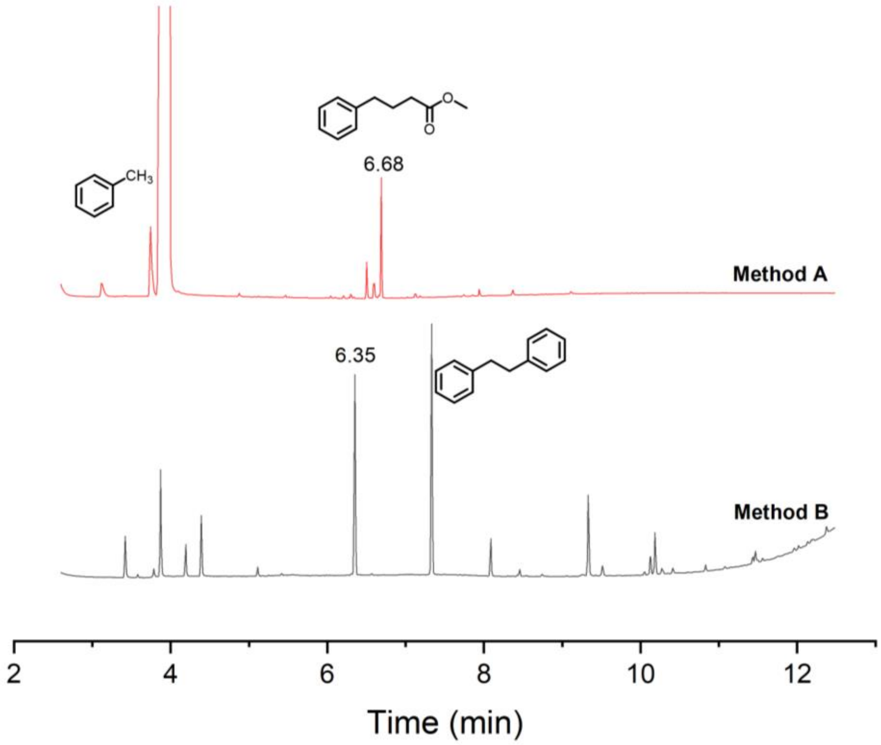

Figure S112. GC chromatograms of the reaction mixtures of **39a** described in Entries 1 (grey) and 2 (red).

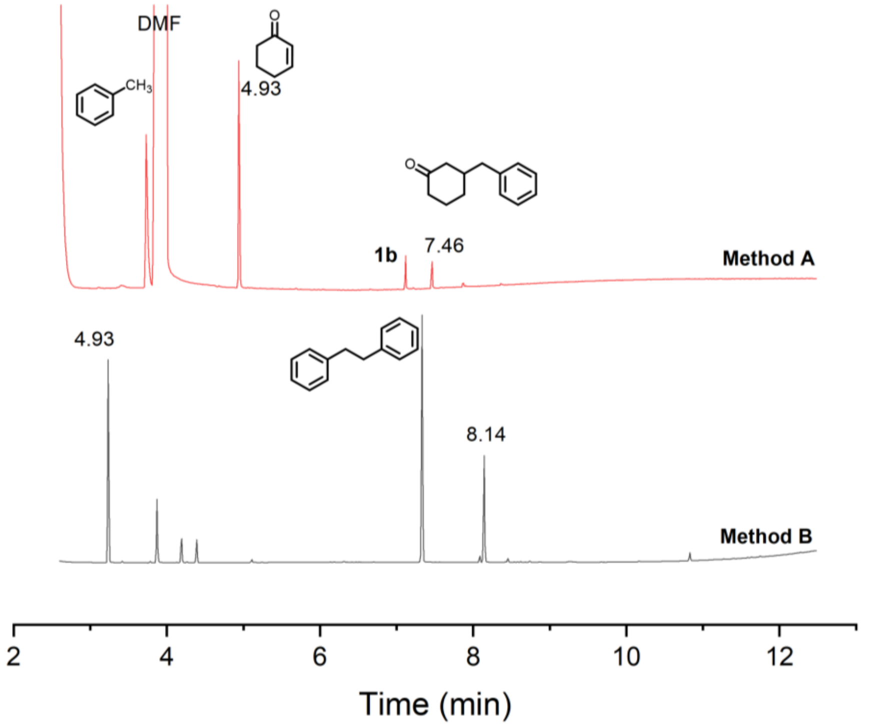

Figure S113. GC chromatograms of the reaction mixtures of **40a** described in Entries 1 (grey) and 2 (red).

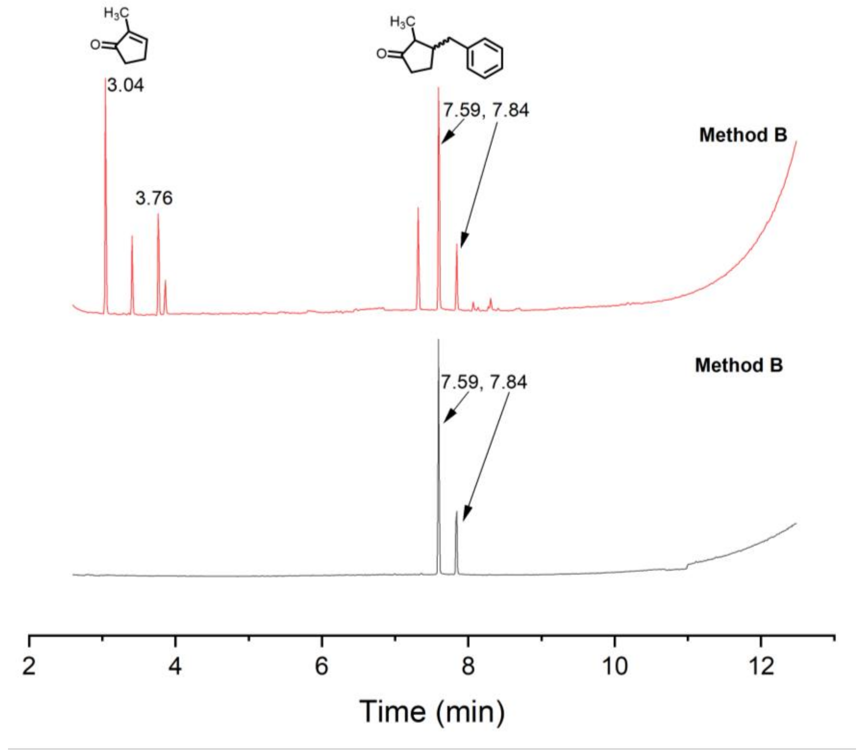

Figure S114. GC chromatograms of the reaction mixtures of **41a** described in Entries 1 (grey) and 2 (red).

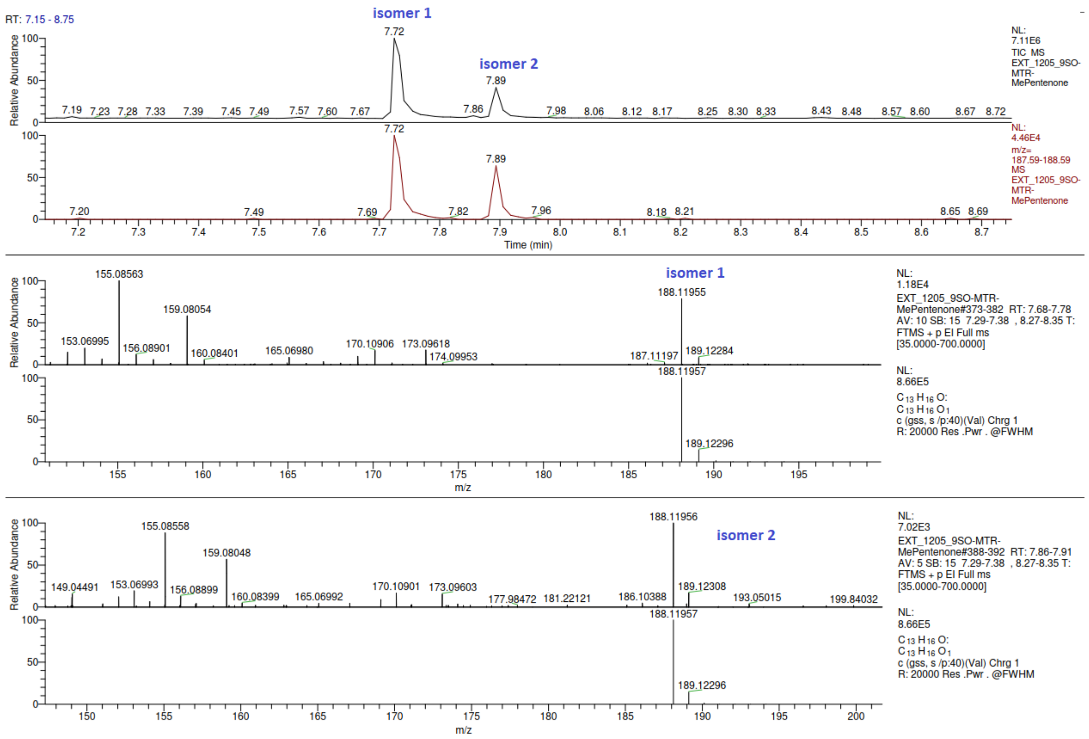

Figure S115. GC-HRMS chromatogram of the diastereomers of **41b**.

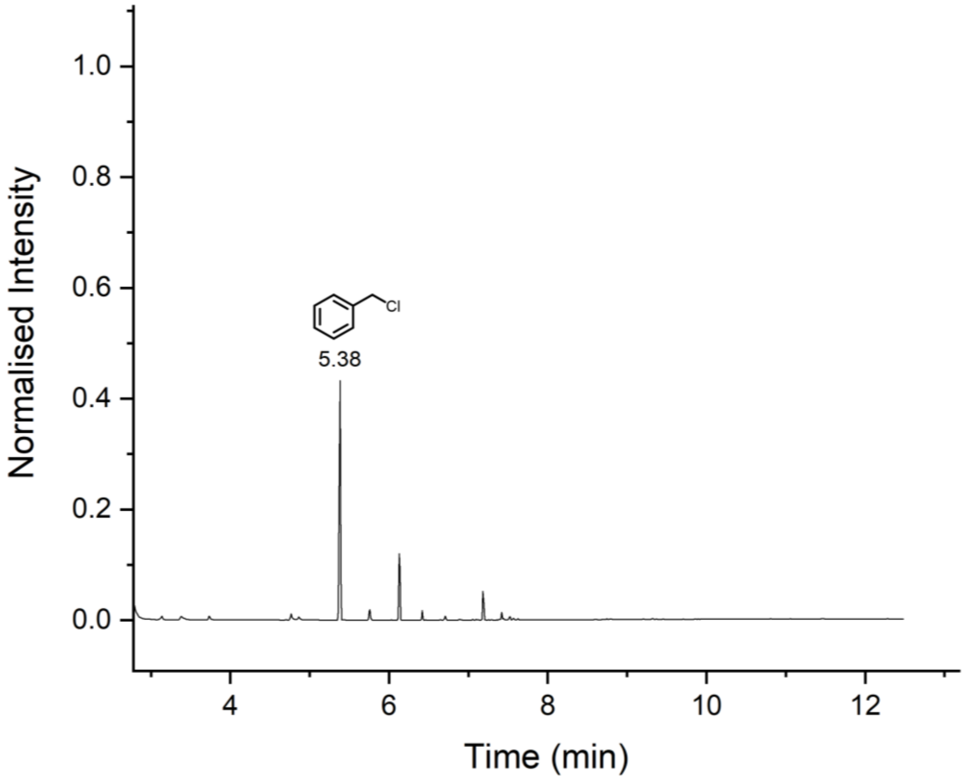

Figure S116. GC chromatogram of the reaction mixture of **41a** with stoichiometric SmI_2.

Mass traces of Radical-TEMPO quenching study

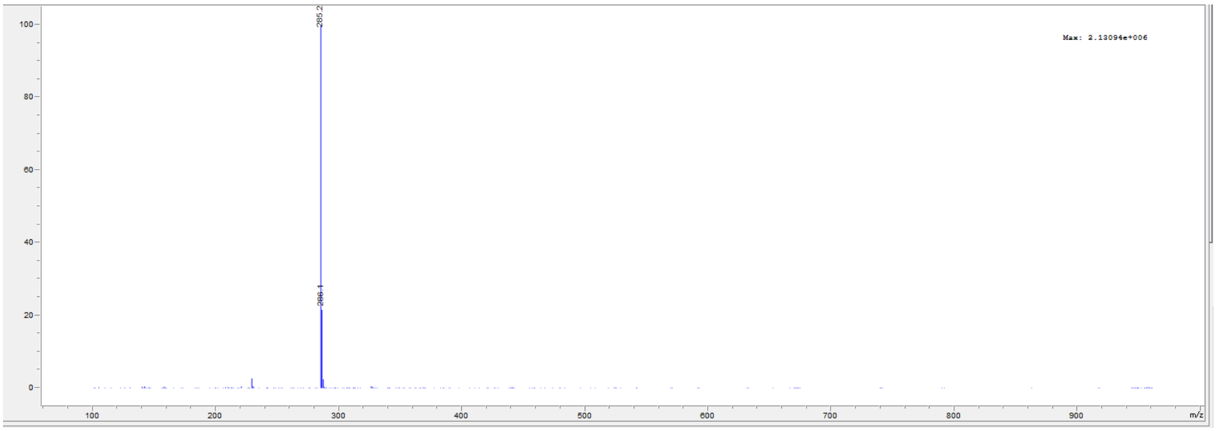

Figure S117. MS chromatogram of **24b-TEMPO** adduct, m/z = 285.2 $(M+H)^+$

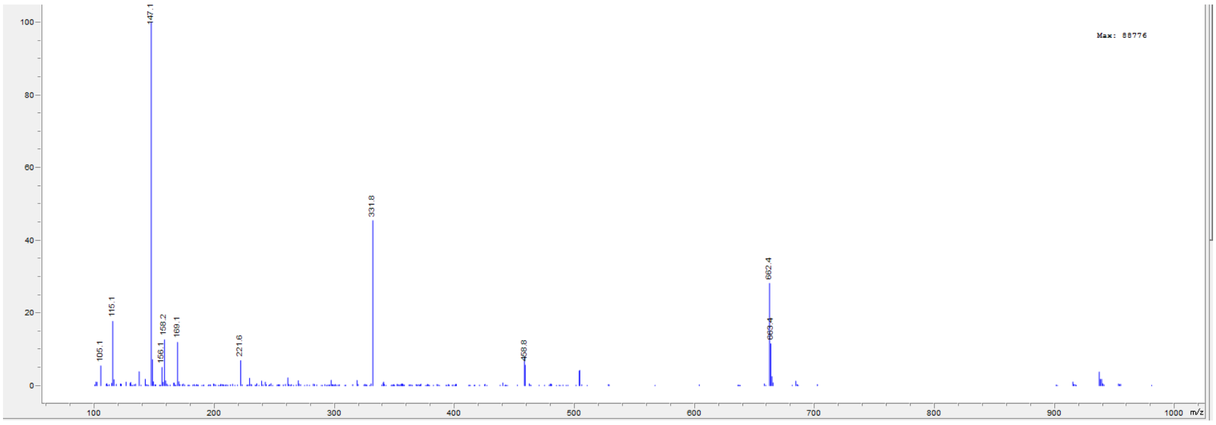

Figure S118. MS chromatogram of **L1-TEMPO** adduct, m/z = 662.2 $(M+H)^+$

^{1}H, ^{13}C, ^{19}F, and ^{31}P NMR spectra

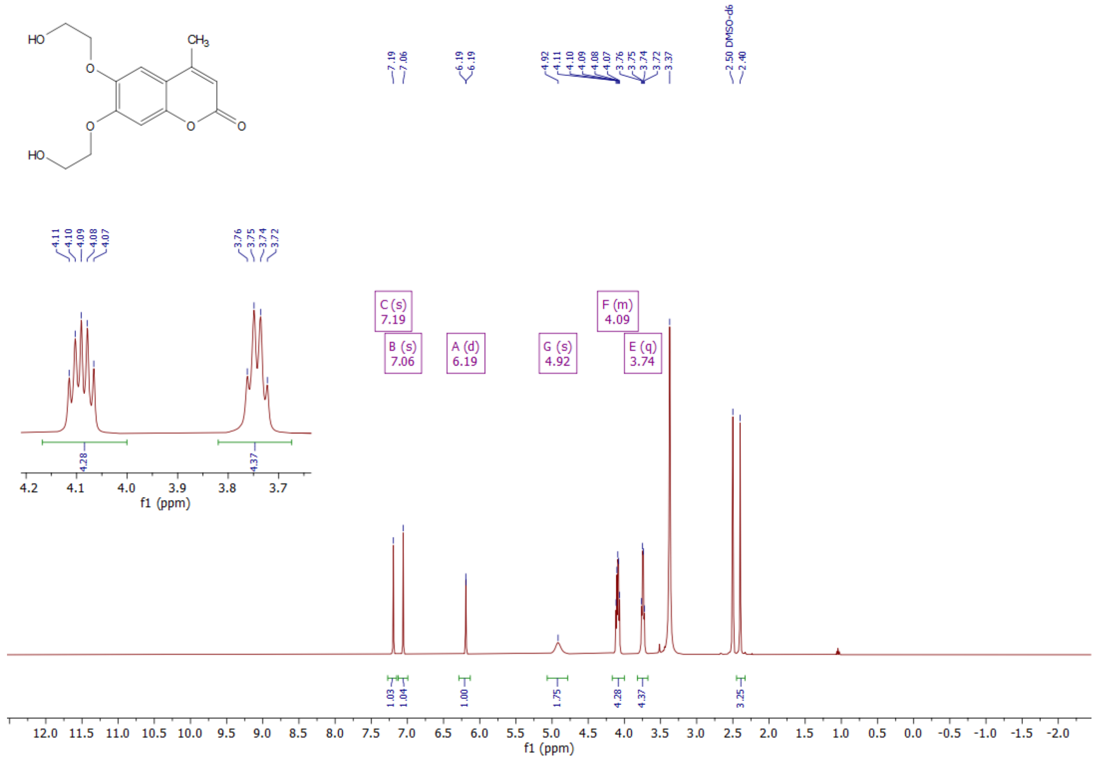

Figure S119. ^{1}H NMR spectrum of **S3** (400 MHz, DMSO-d_6).

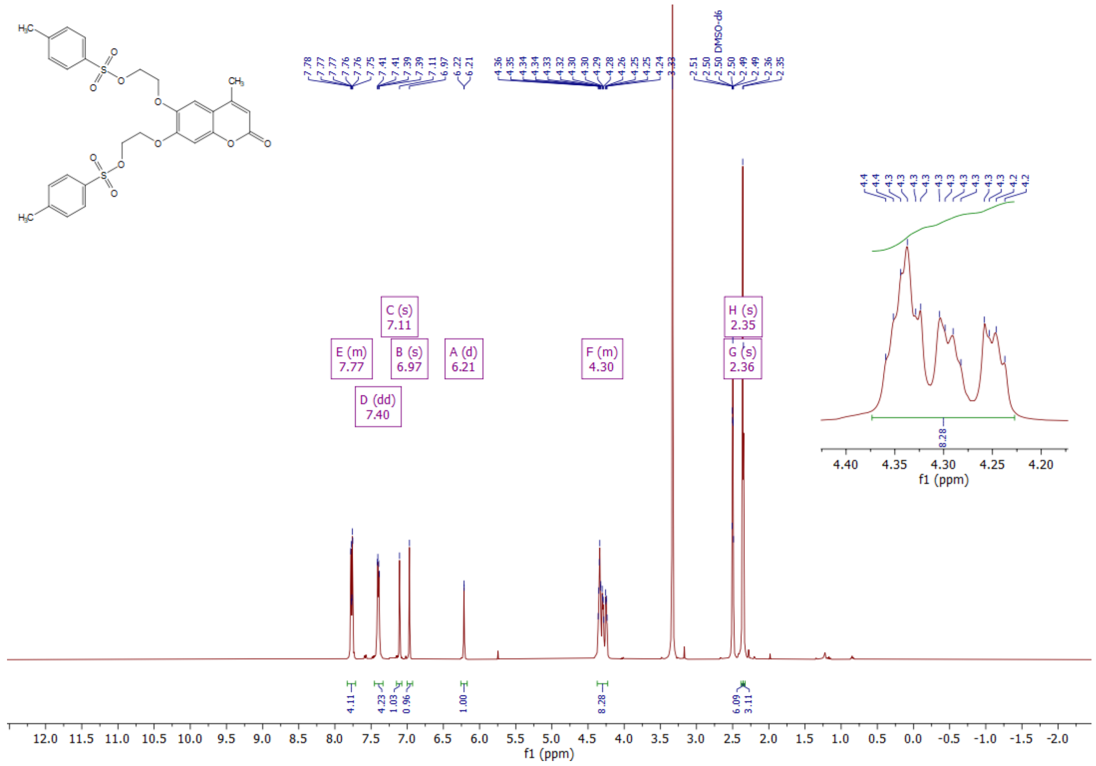

Figure S120. ^{1}H NMR spectrum of **S4** (400 MHz, DMSO-d_6).

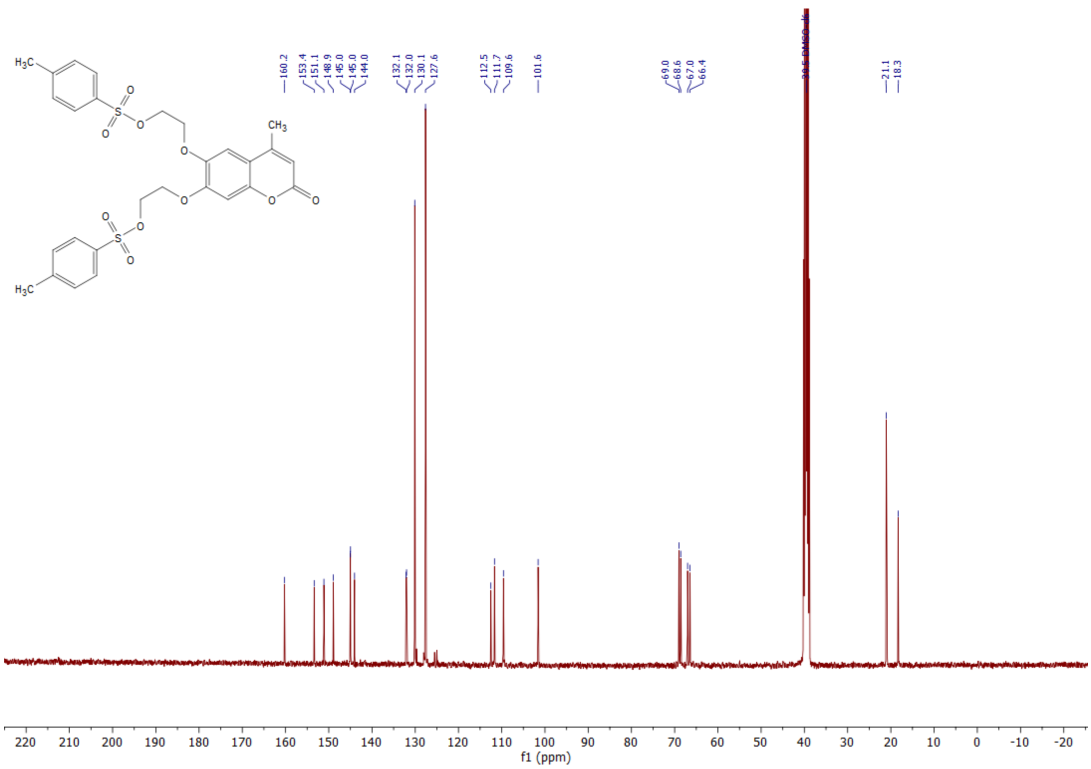

Figure S121. ^{13}C NMR spectrum of **S4** (101 MHz, DMSO-d_6).

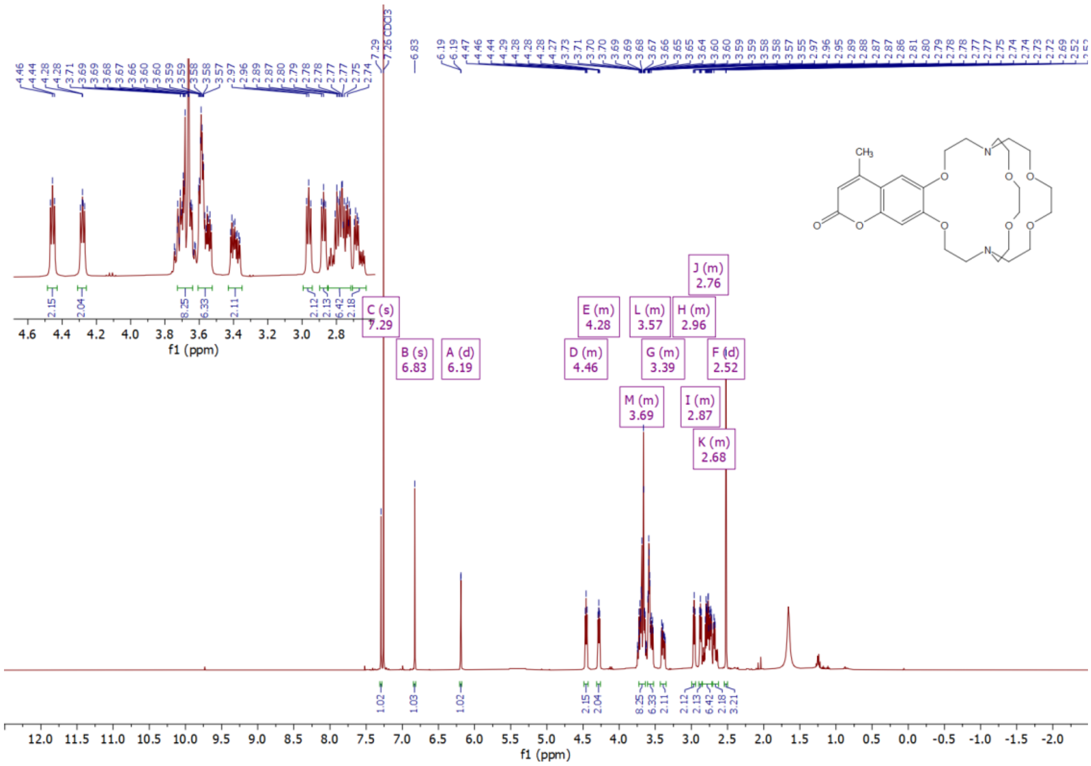

Figure S122. ^{1}H NMR spectrum of **L1** (400 MHz, $CDCl_3$).

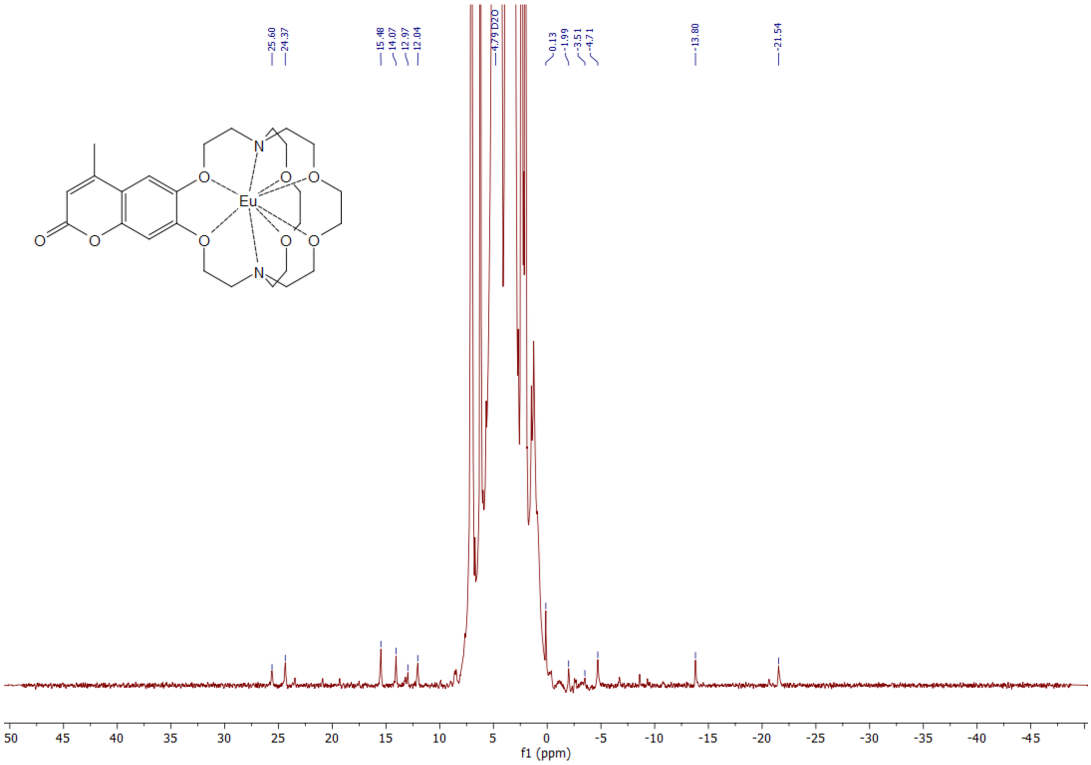

Figure S123. ^{1}H NMR spectrum of **EuL1** (400 MHz, D_2O).

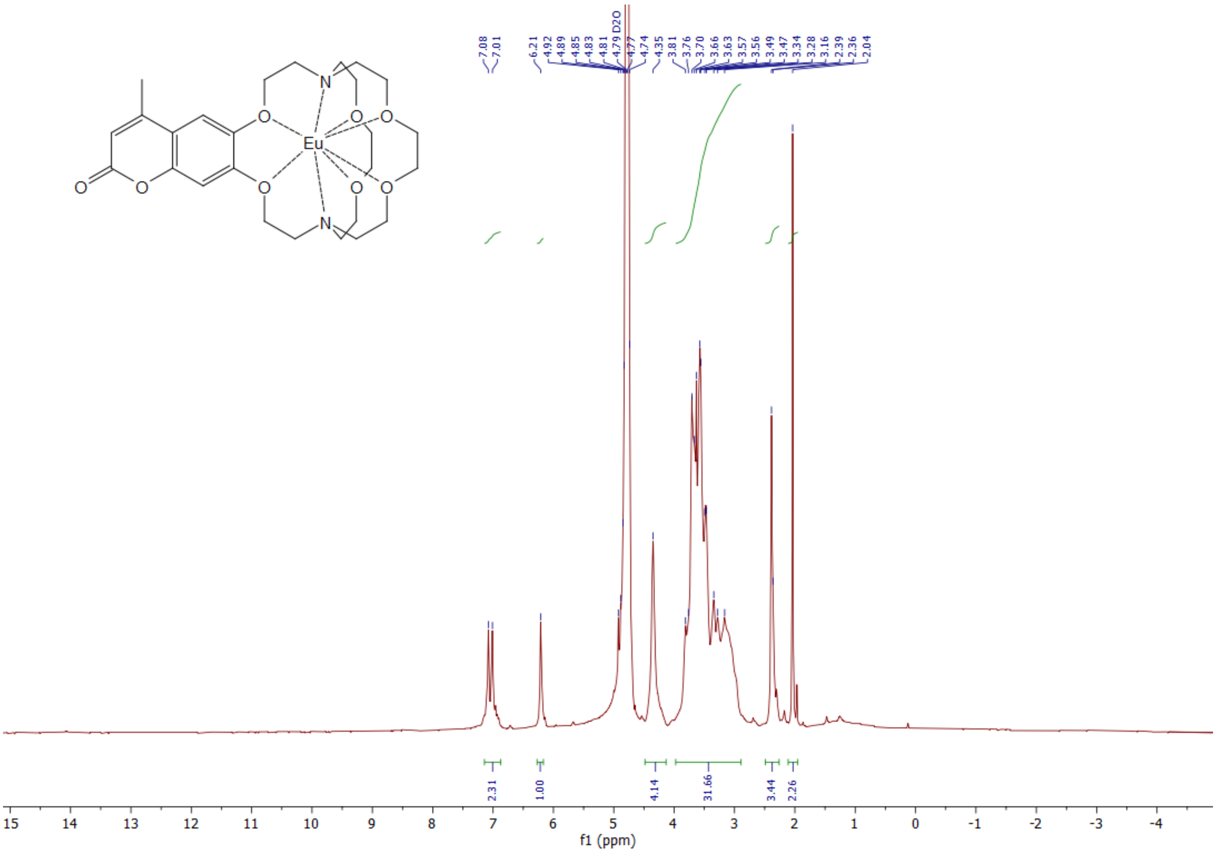

Figure S124. Expansion of ^{1}H NMR spectrum of **EuL1** (400 MHz, D_2O).

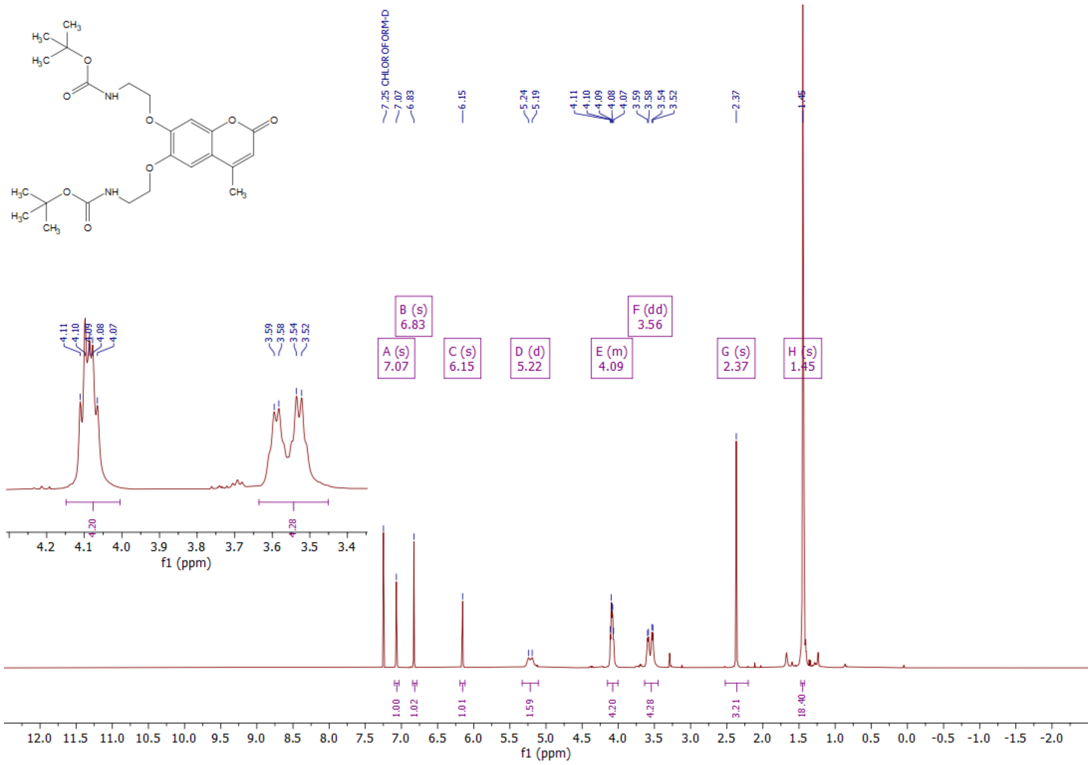

Figure S125. ^{1}H NMR spectrum of **S7** (400 MHz, $CDCl_3$).

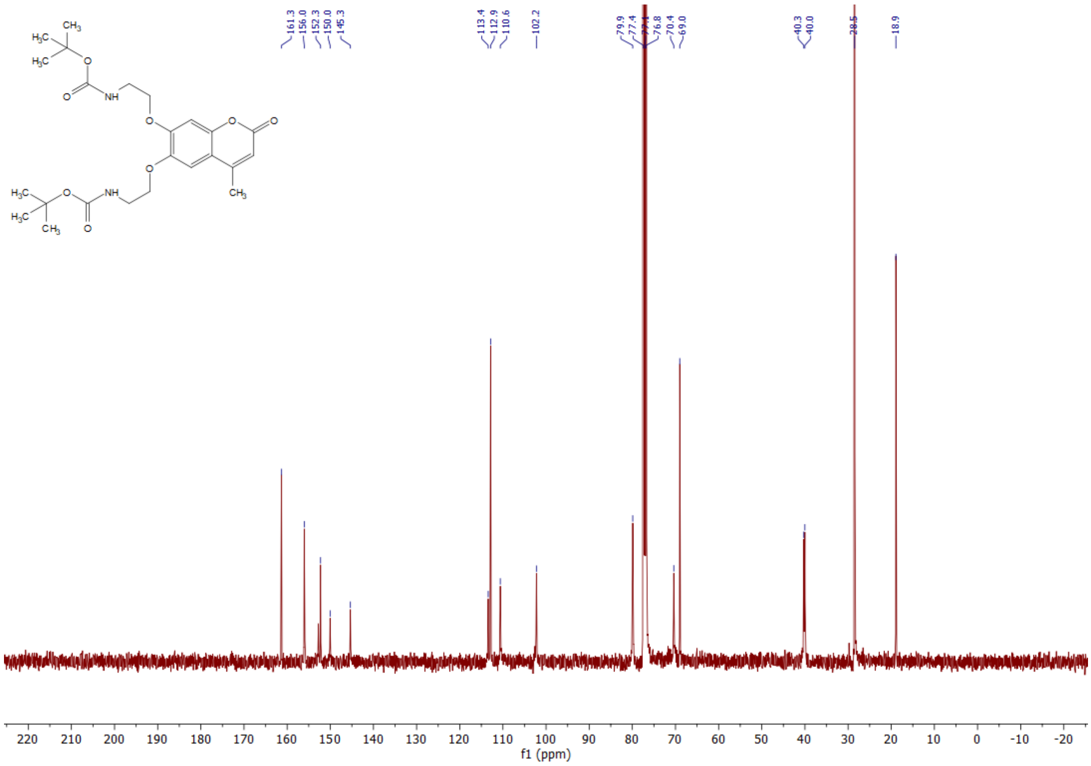

Figure S126. ^{13}C NMR spectrum of **S7** (101 MHz, $CDCl_3$).

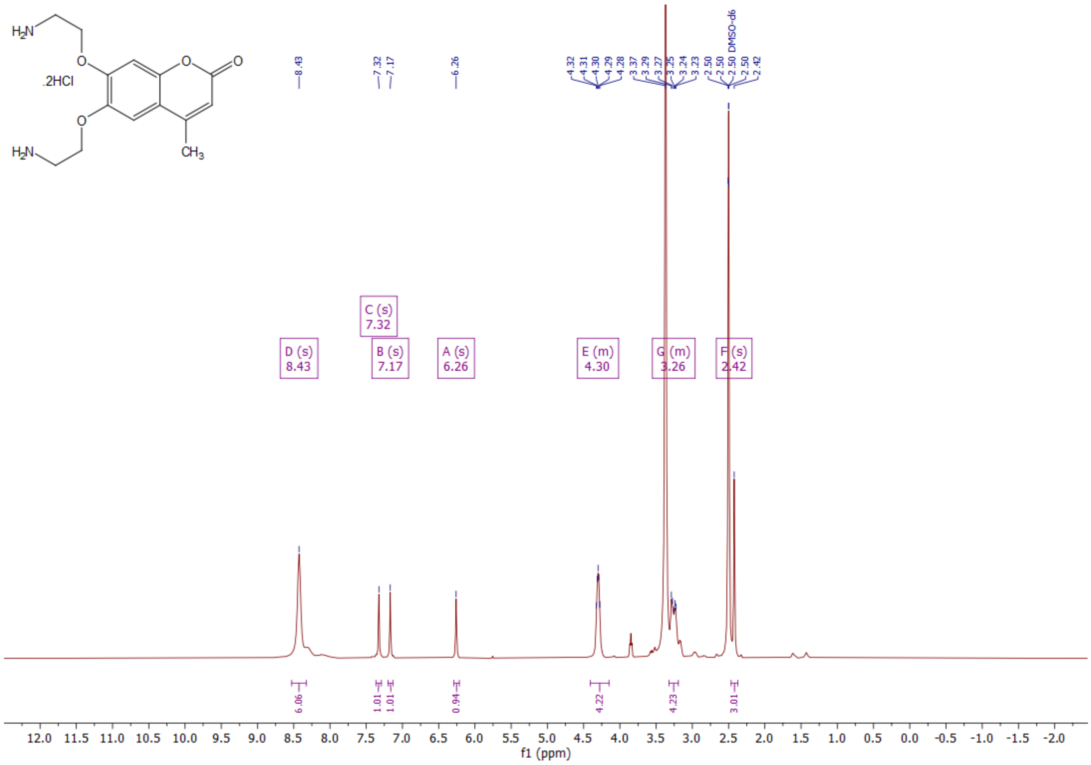

Figure S127. ^{1}H NMR spectrum of **S8** (400 MHz, DMSO-d_6).

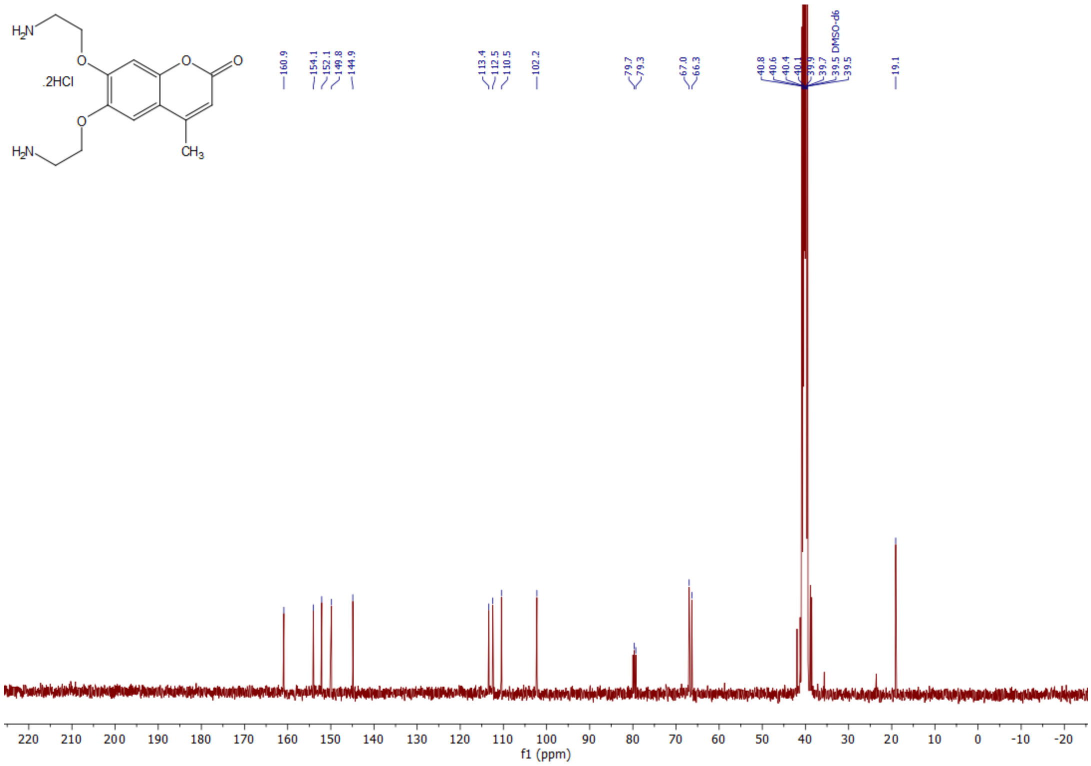

Figure S128. ^{13}C NMR spectrum of **S8** (101 MHz, DMSO-d_6).

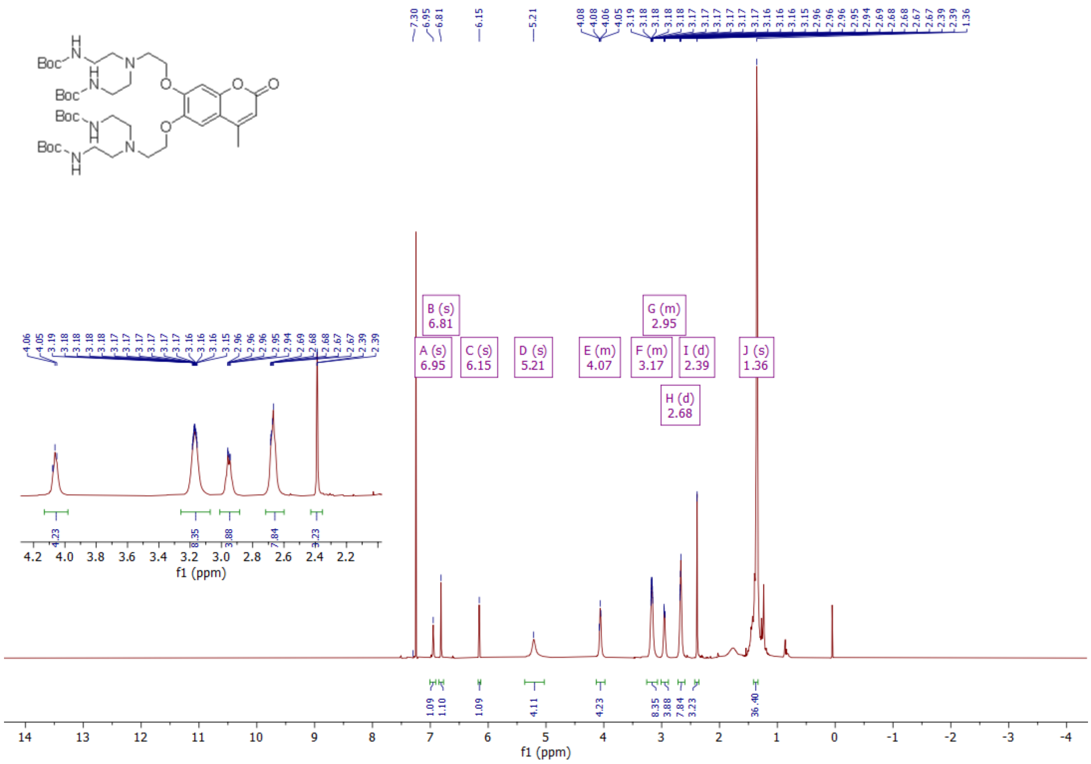

Figure S129. ^{1}H NMR spectrum of **S10** (400 MHz, $CDCl_3$).

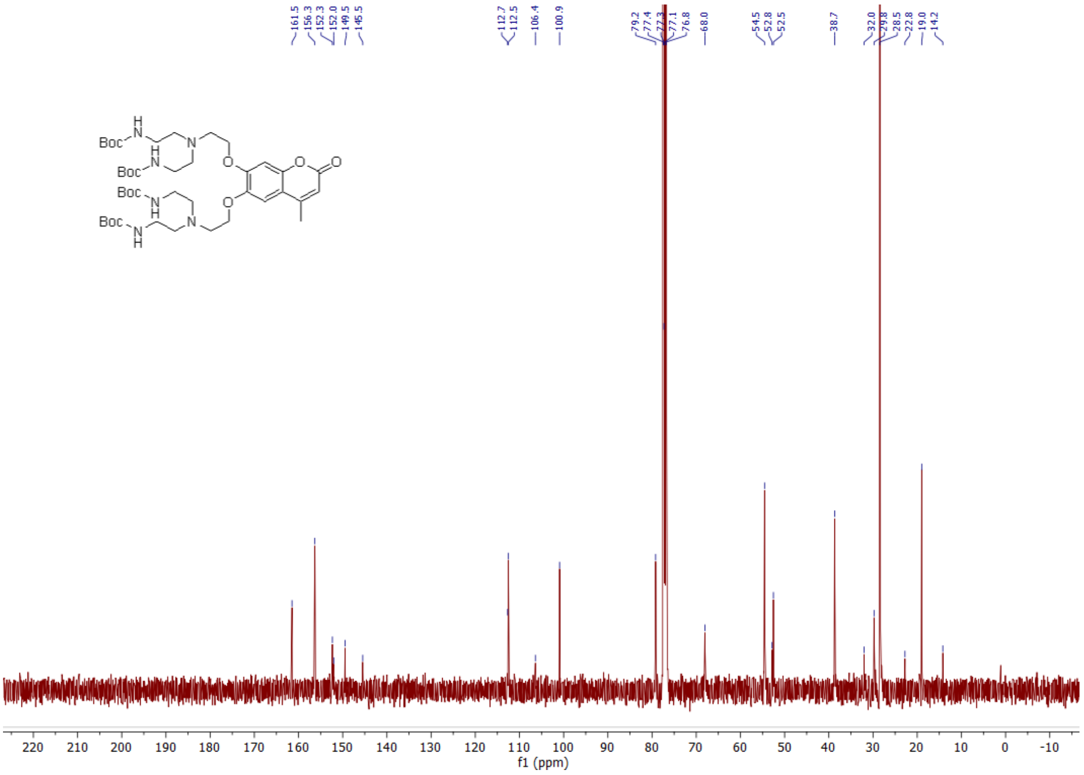

Figure S130. ^{13}C NMR spectrum of **S10** (101 MHz, $CDCl_3$).

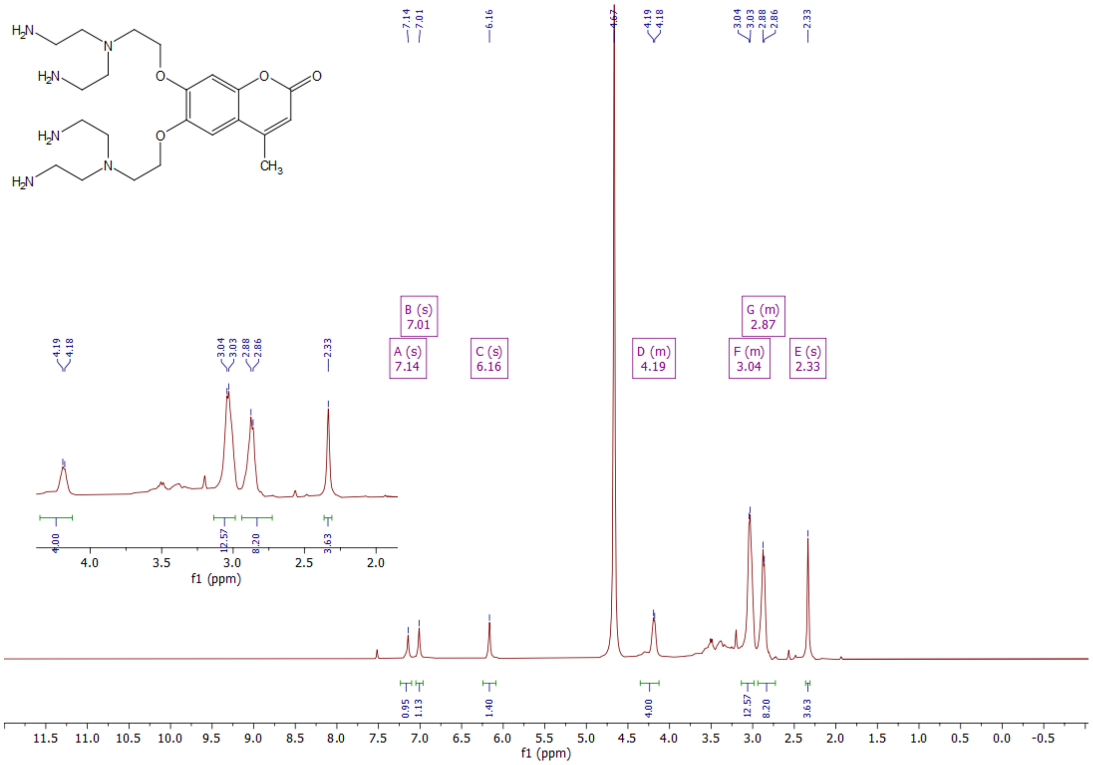

Figure S131. ^{1}H NMR spectrum of **L2** (400 MHz, D_2O).

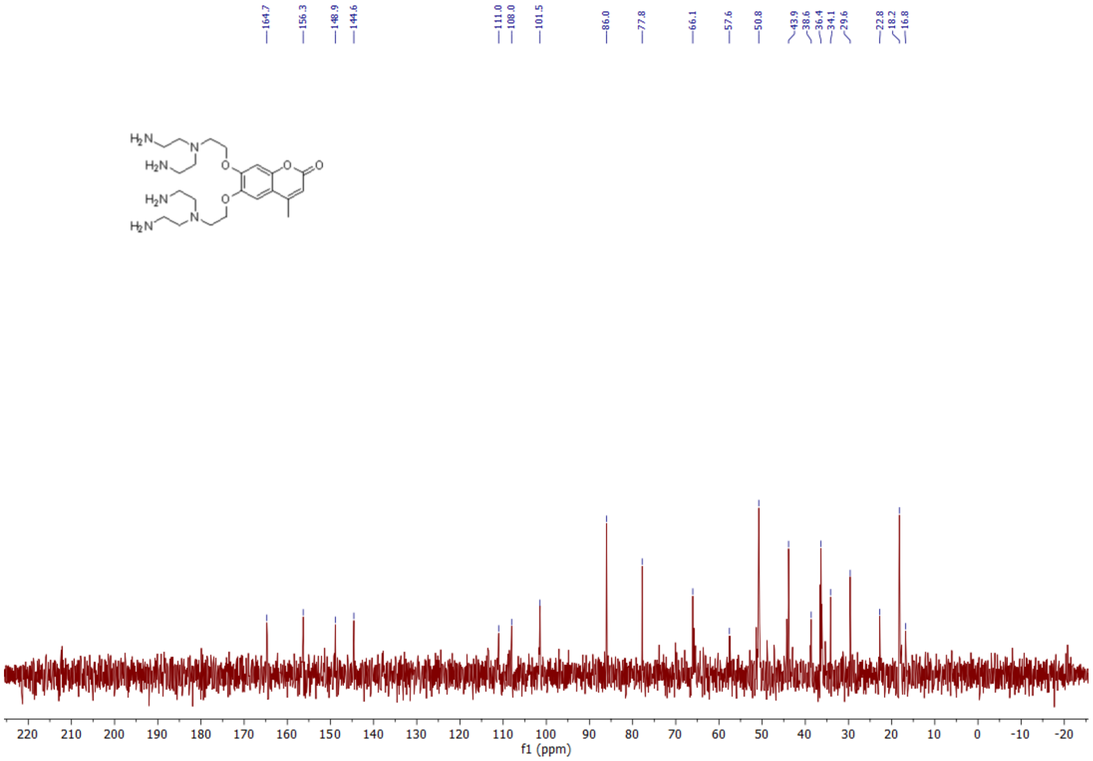

Figure S132. ^{13}C NMR spectrum of **L2** (101 MHz, $CDCl_3$).

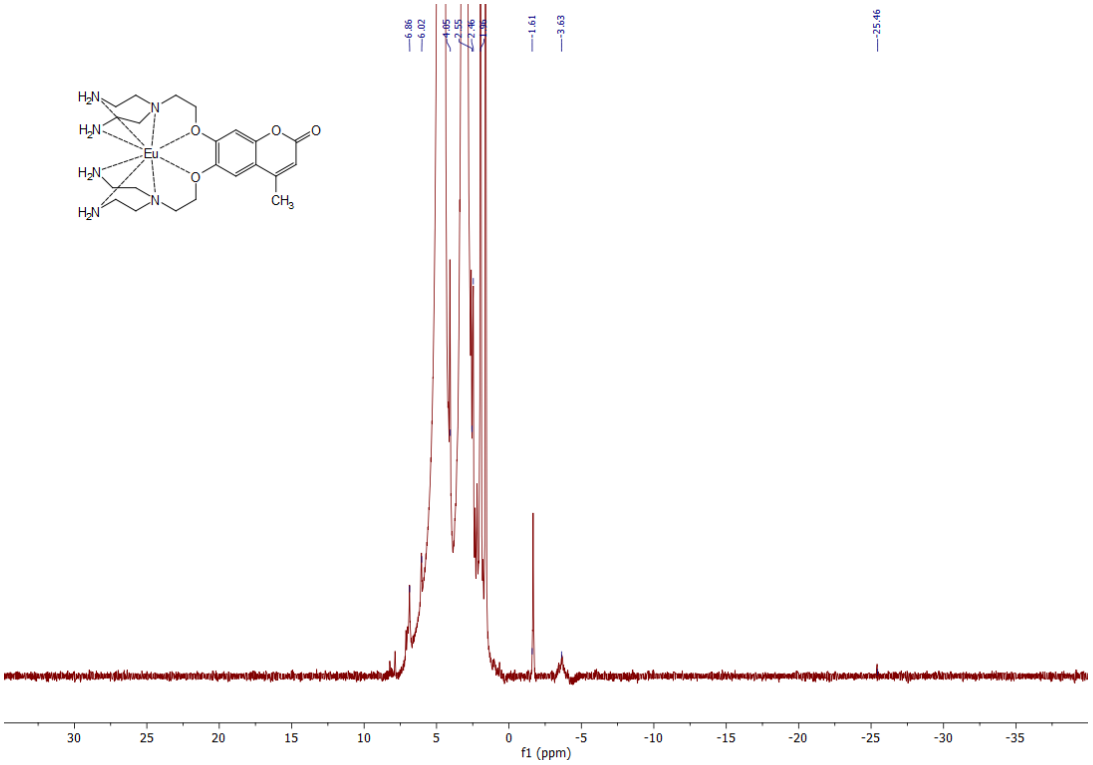

Figure S133. ^{1}H NMR spectrum of **EuL2** (400 MHz, D_2O).

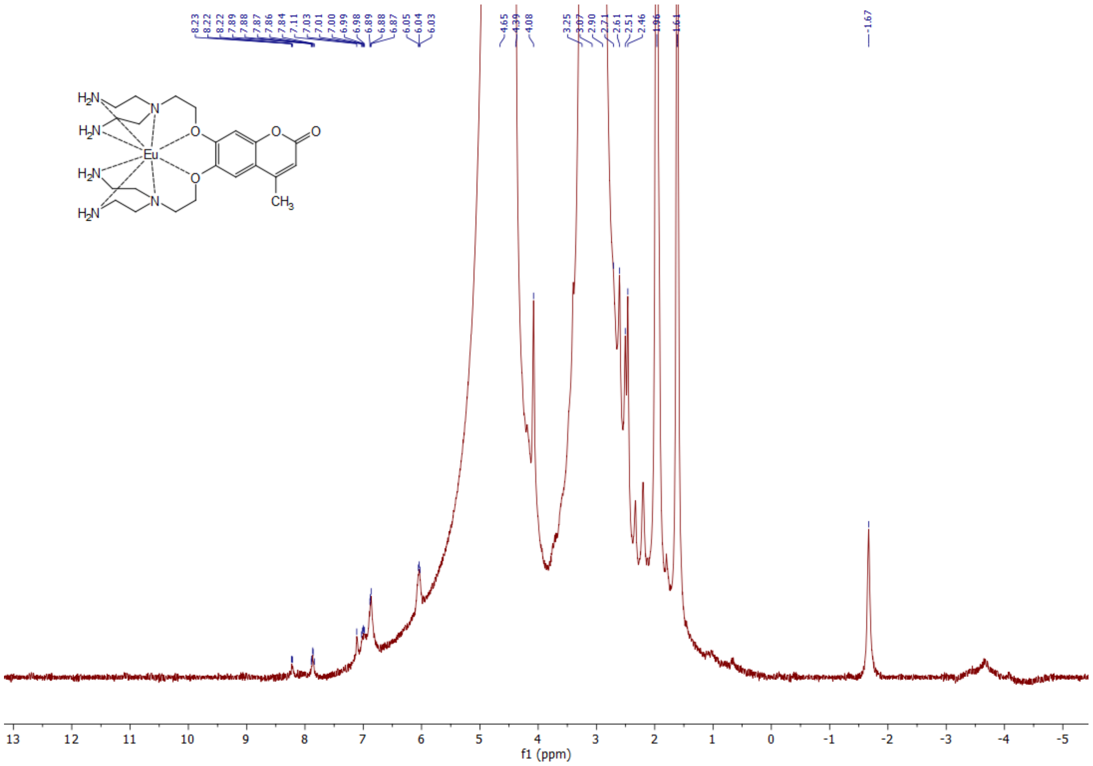

Figure S134. Expansion of ^{1}H NMR spectrum of **EuL2** (400 MHz, D_2O).

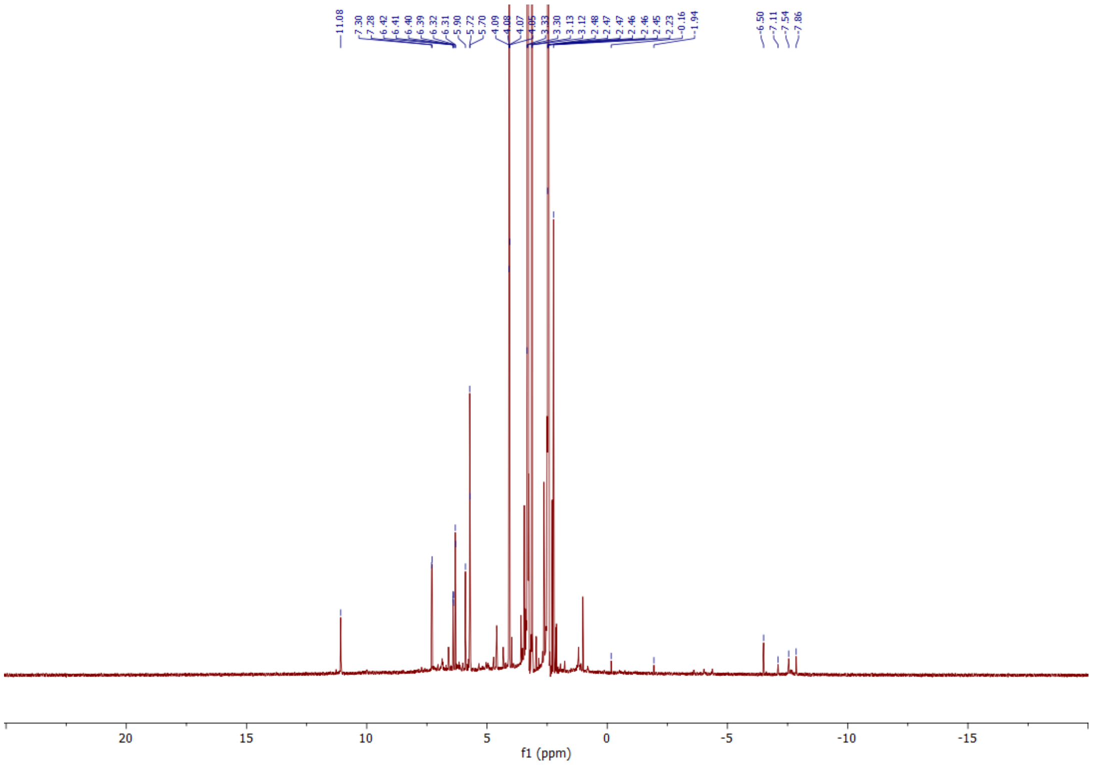

Figure S135. ^{1}H NMR spectrum of **EuL3** (400 MHz, DMSO-d_6).

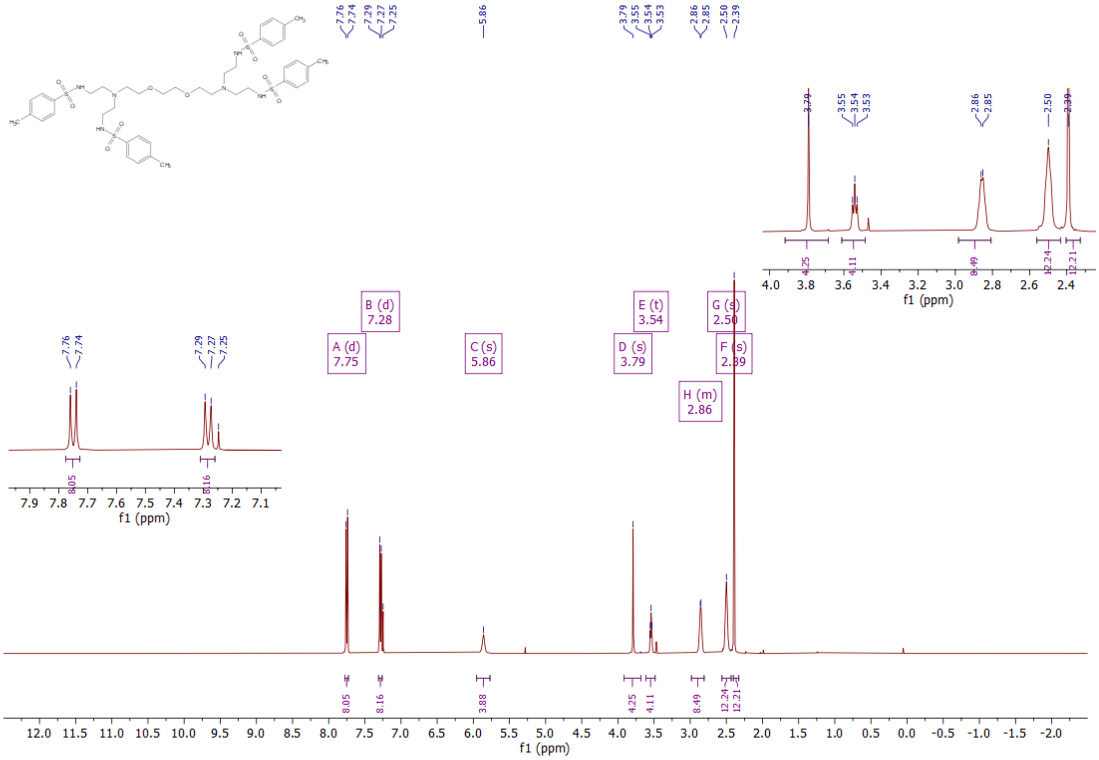

Figure S136. ^{1}H NMR spectrum of **S13** (400 MHz, D_2O).

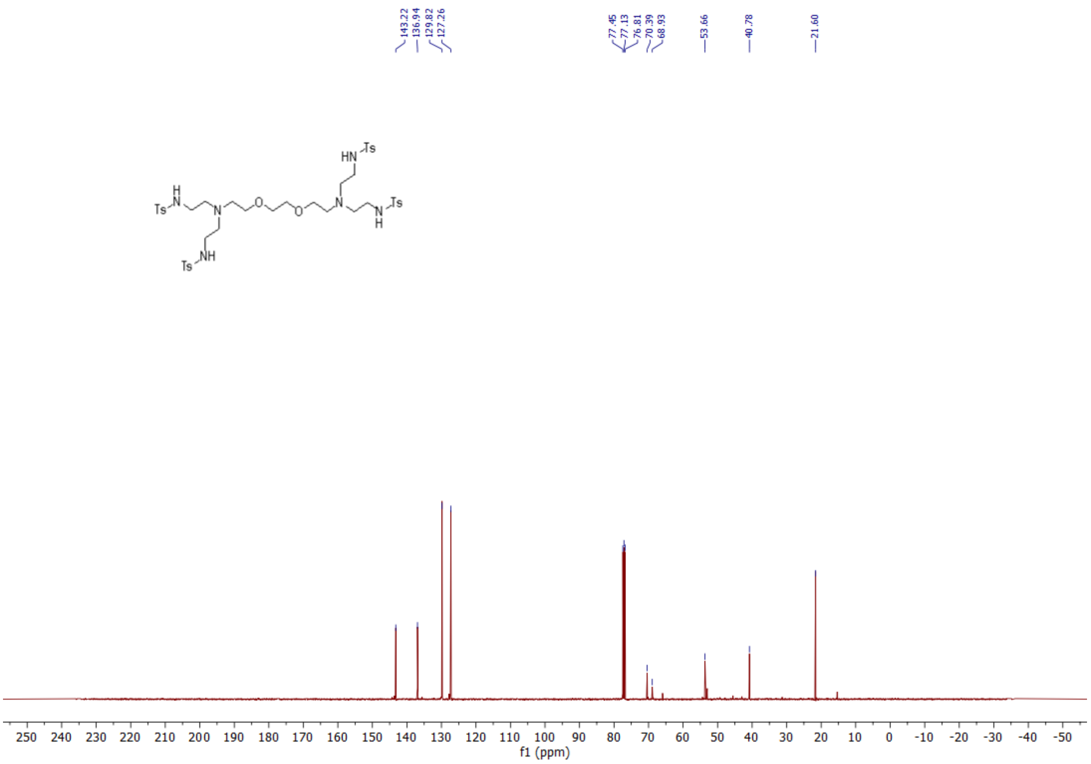

Figure S137. ^{13}C NMR spectrum of **S13** (101 MHz, D_2O).

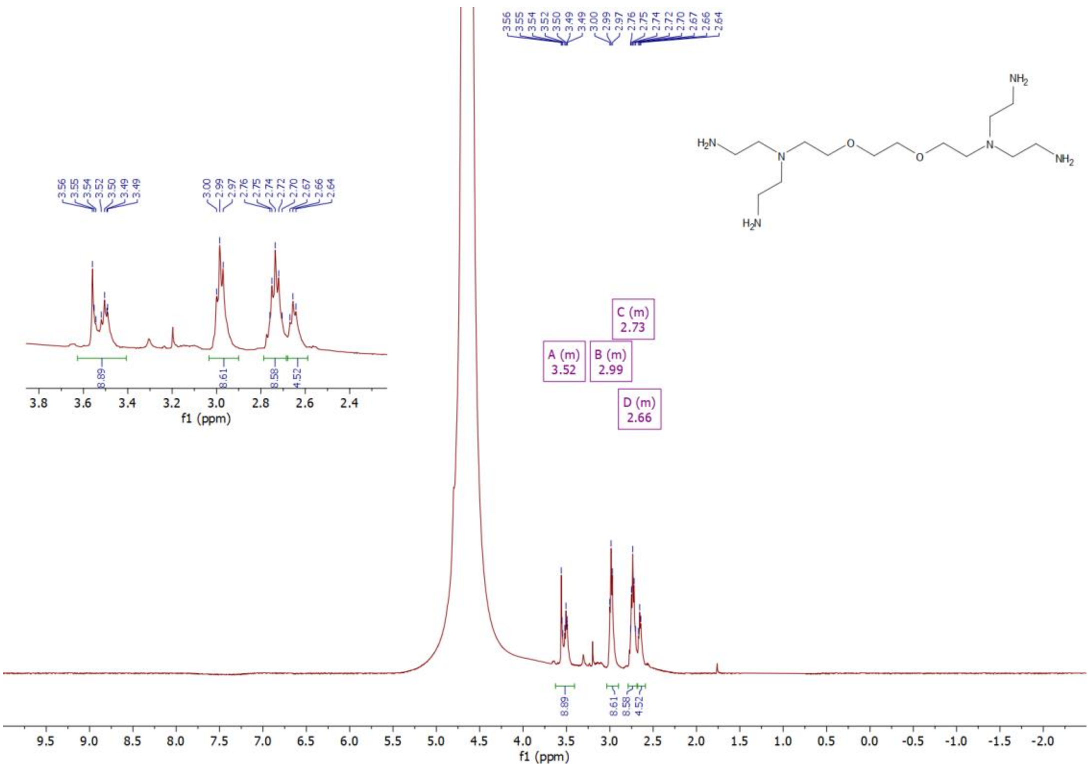

Figure S138. ^{1}H NMR spectrum of **L2m** (400 MHz, D_2O).

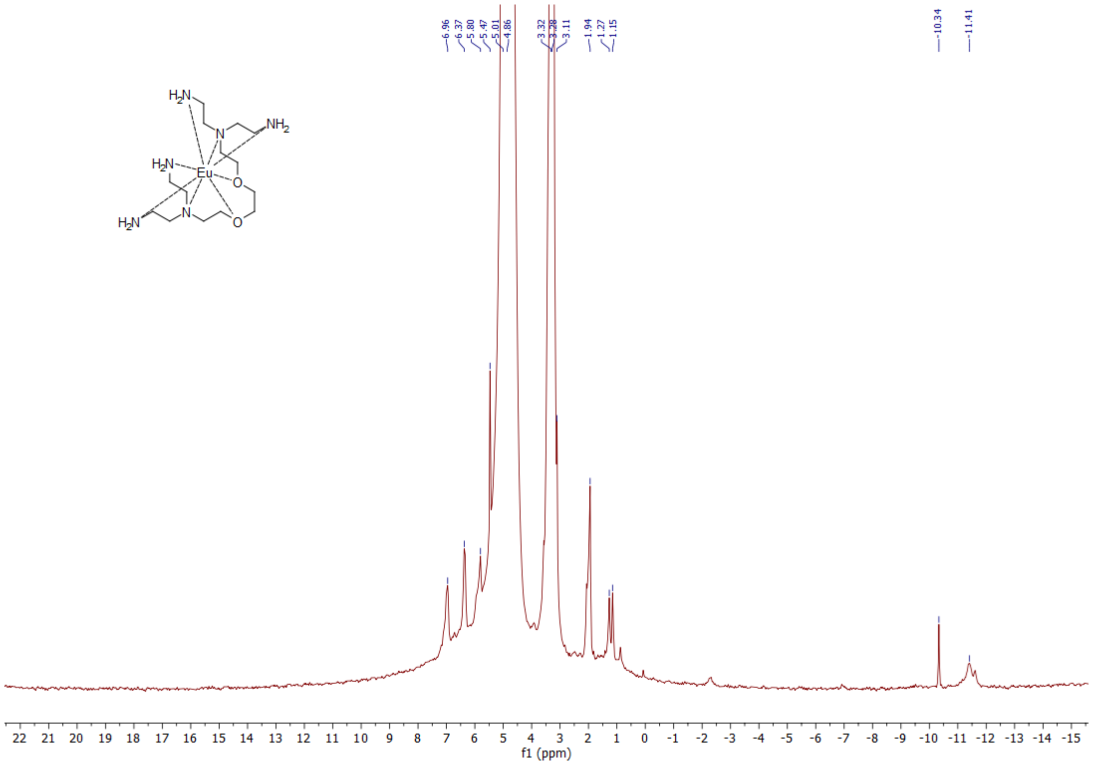

Figure S139. ^{1}H NMR spectrum of **EuL2m** (400 MHz, D_2O).

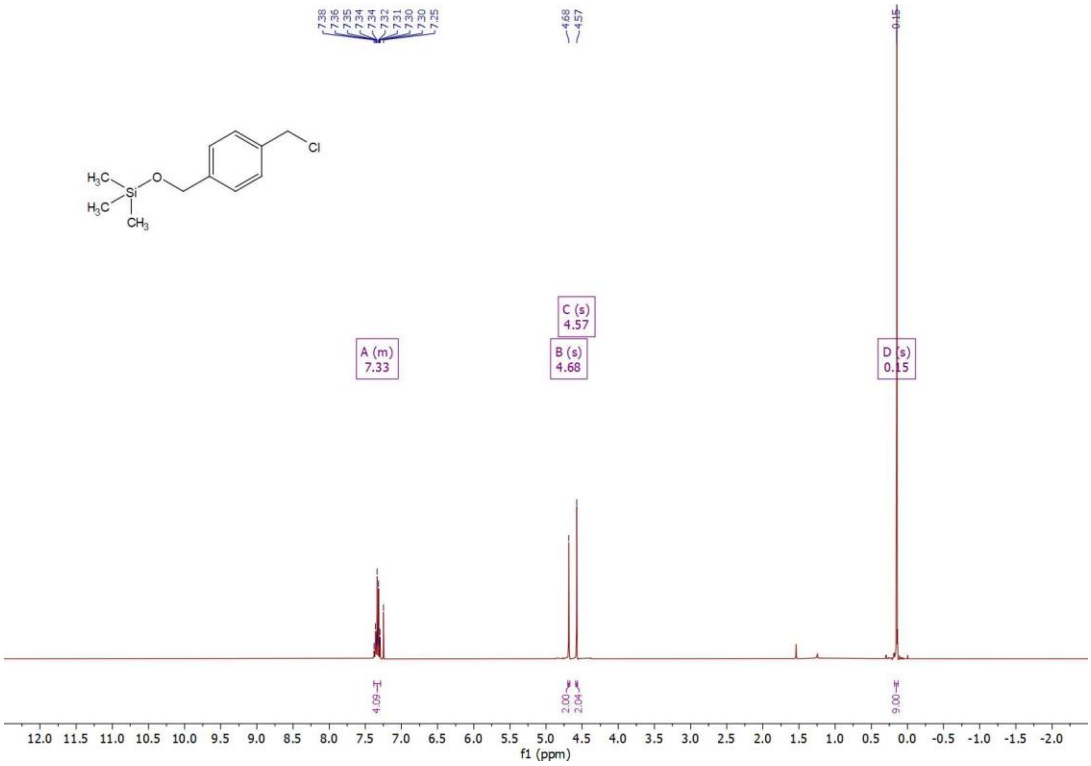

Figure S140. ^{1}H NMR spectrum of **12a** (400 MHz, $CDCl_3$).

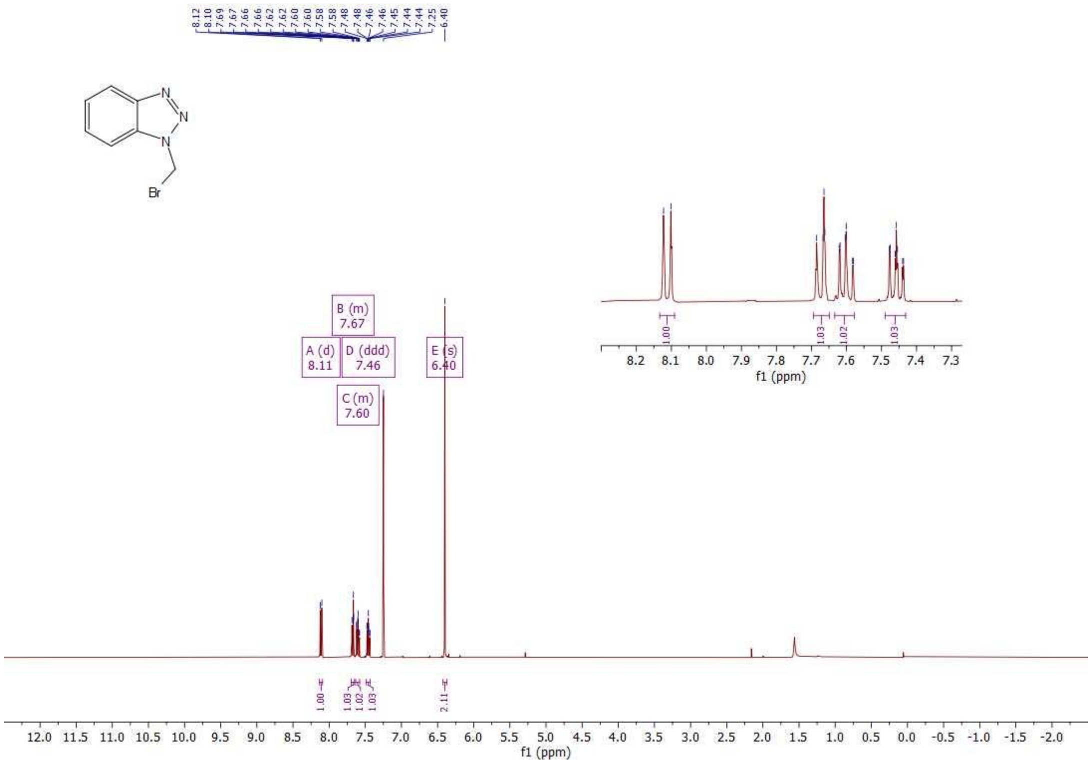

Figure S141. ^1H NMR spectrum of **17a** (400 MHz, $CDCl_3$).

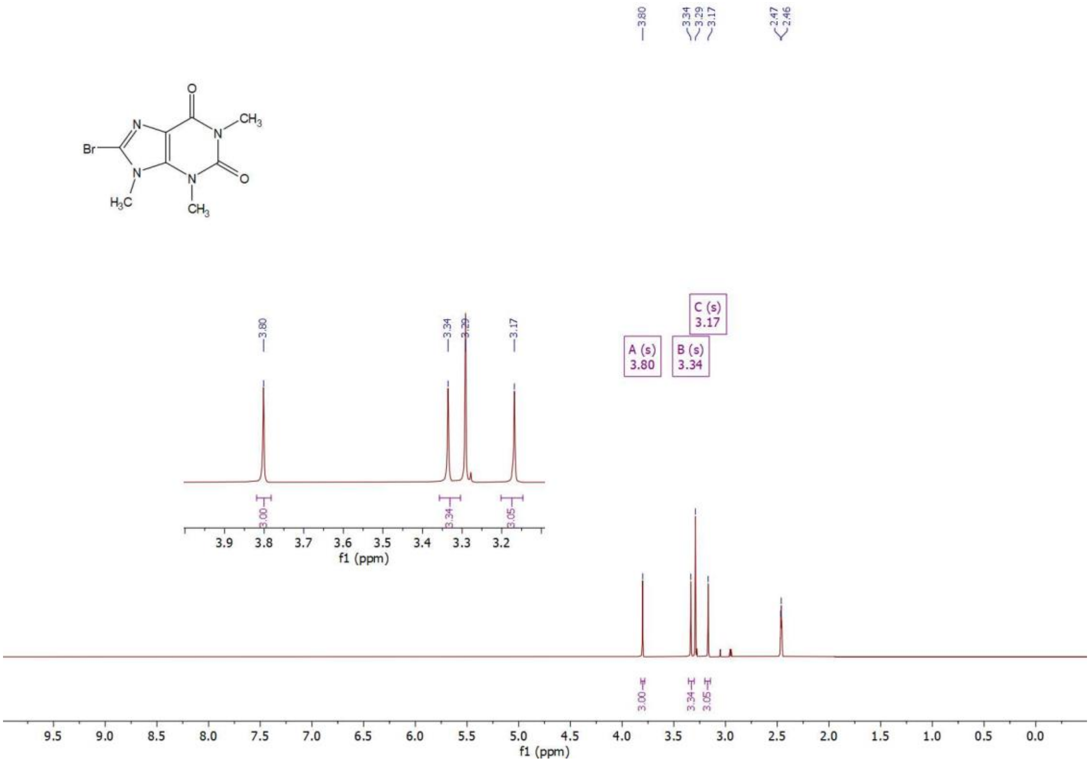

Figure S142. ^1H NMR spectrum of **36a** (400 MHz, DMSO-d_6).

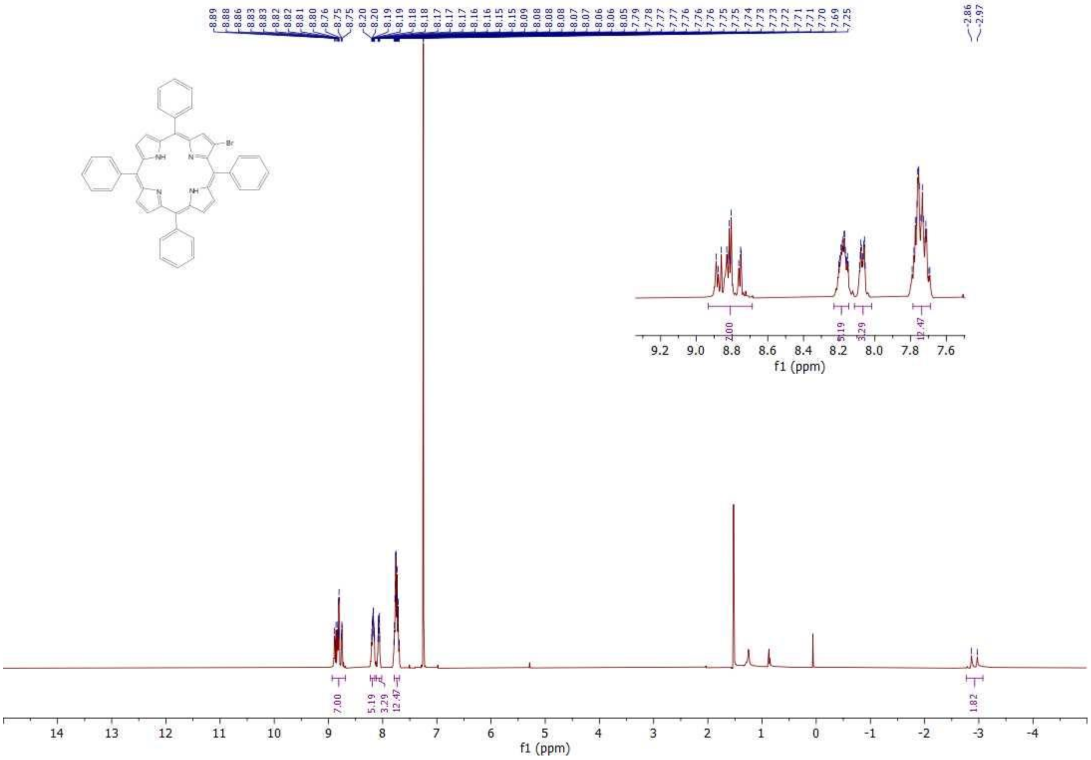

Figure S143. ^{1}H NMR spectrum of **37a** (400 MHz, $CDCl_3$).

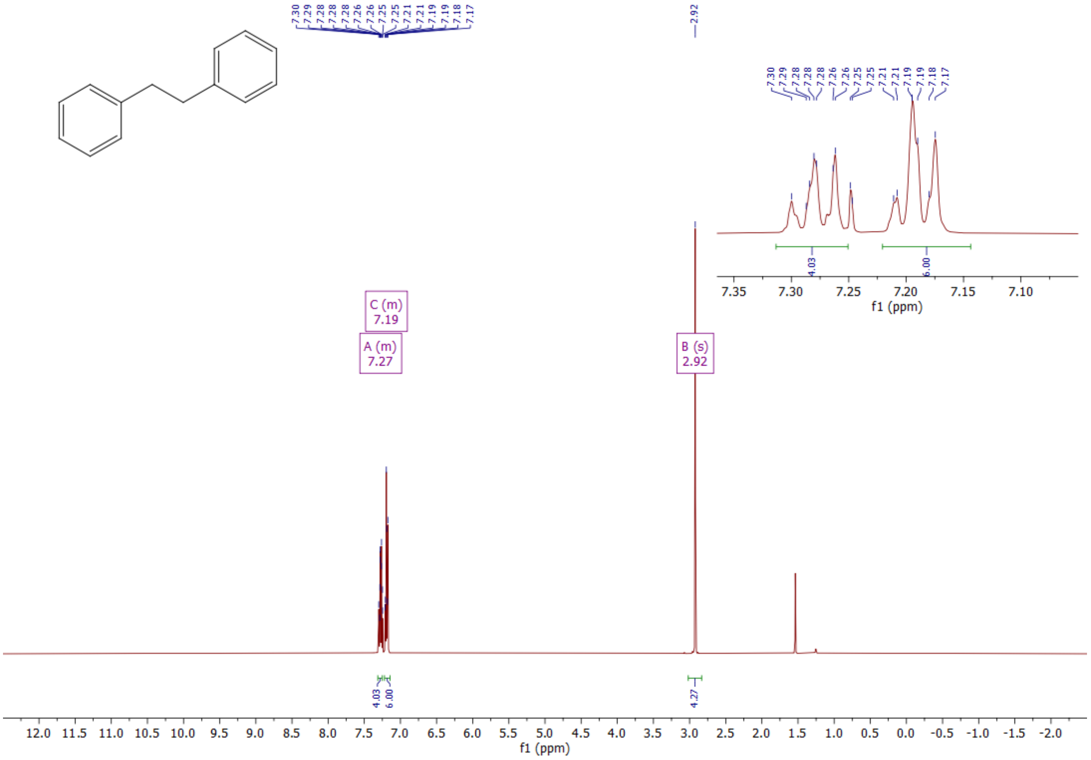

Figure S144. ^{1}H NMR spectrum of **1b** (400 MHz, $CDCl_3$).

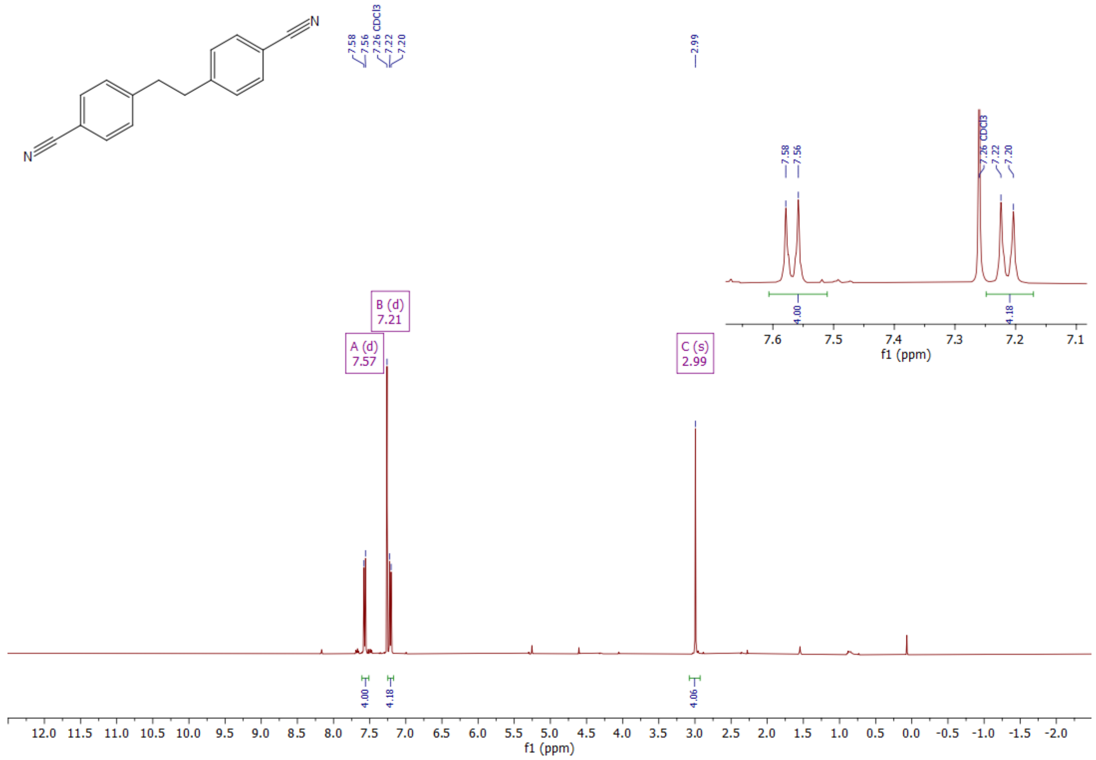

Figure S145. ^{1}H NMR spectrum of **2b** (400 MHz, $CDCl_3$).

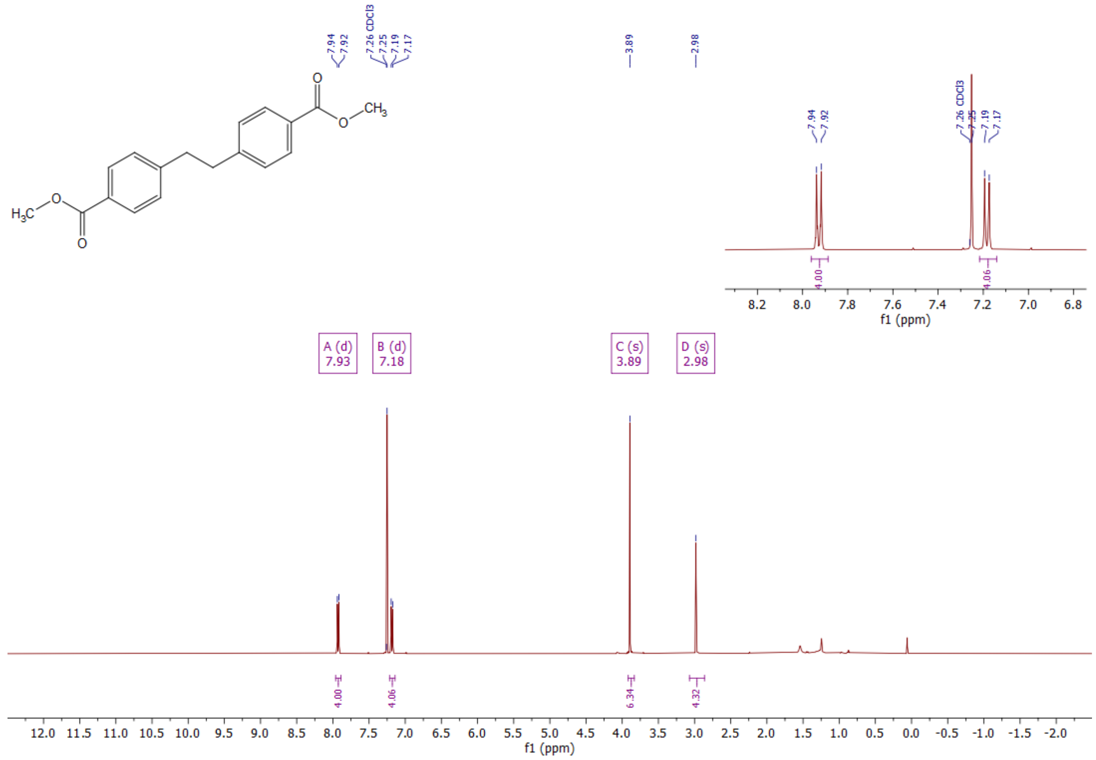

Figure S146. ^{1}H NMR spectrum of **3b** (400 MHz, $CDCl_3$).

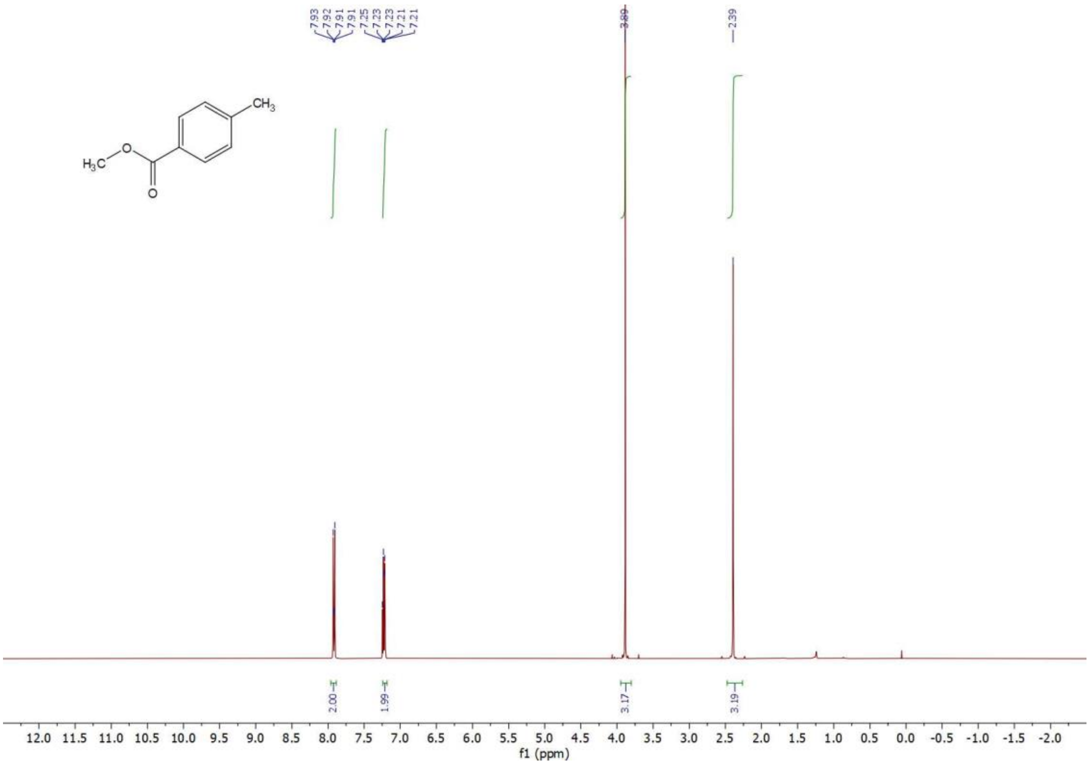

Figure S147. ^{1}H NMR spectrum of **3c** (400 MHz, $CDCl_3$).

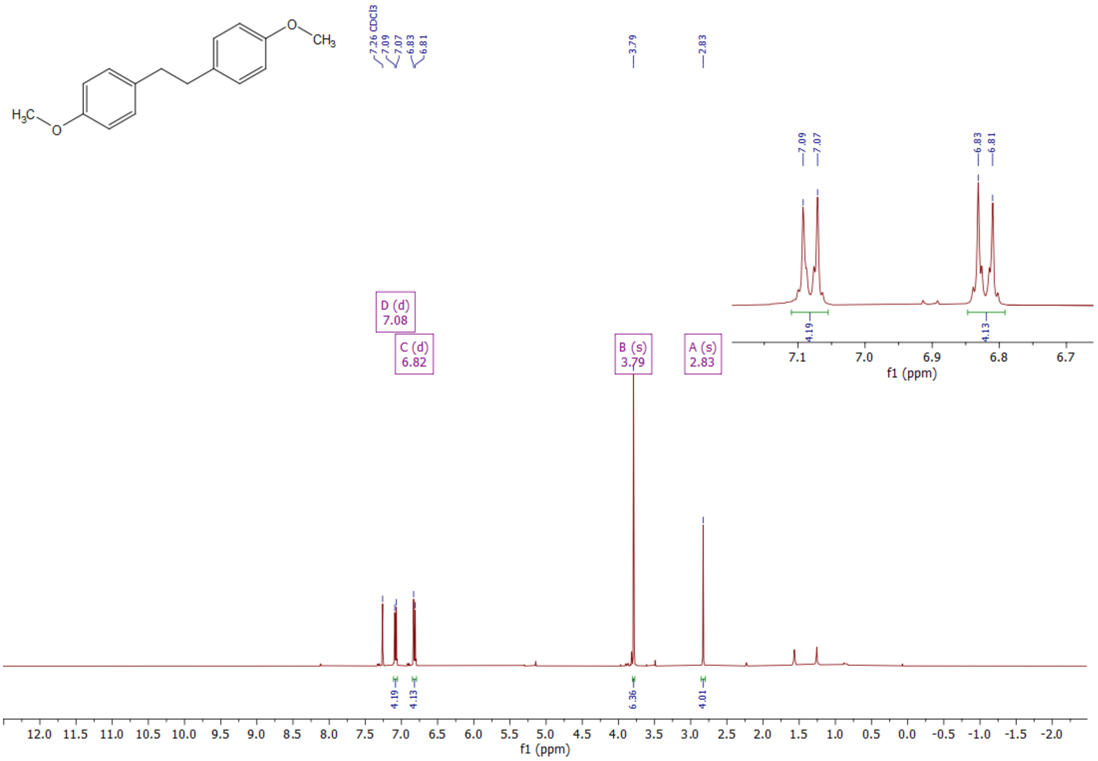

Figure S148. ^{1}H NMR spectrum of **4b** (400 MHz, $CDCl_3$).

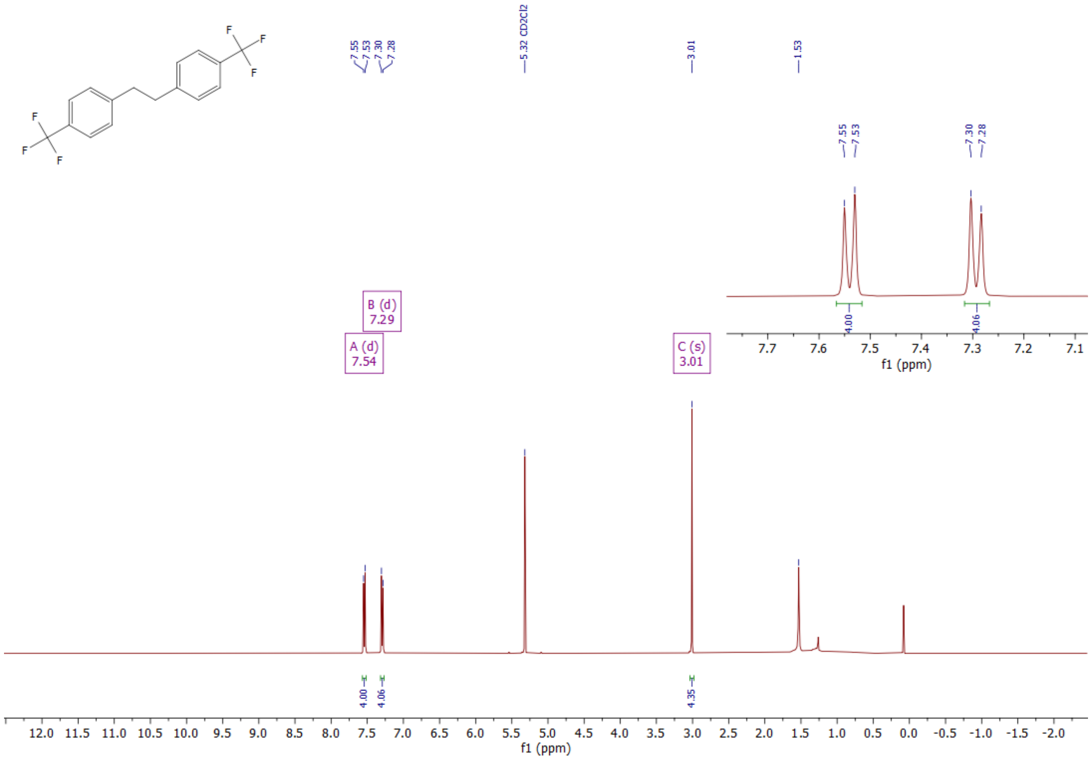

Figure S149. ^{1}H NMR spectrum of **5b** (400 MHz, CD_2Cl_2).

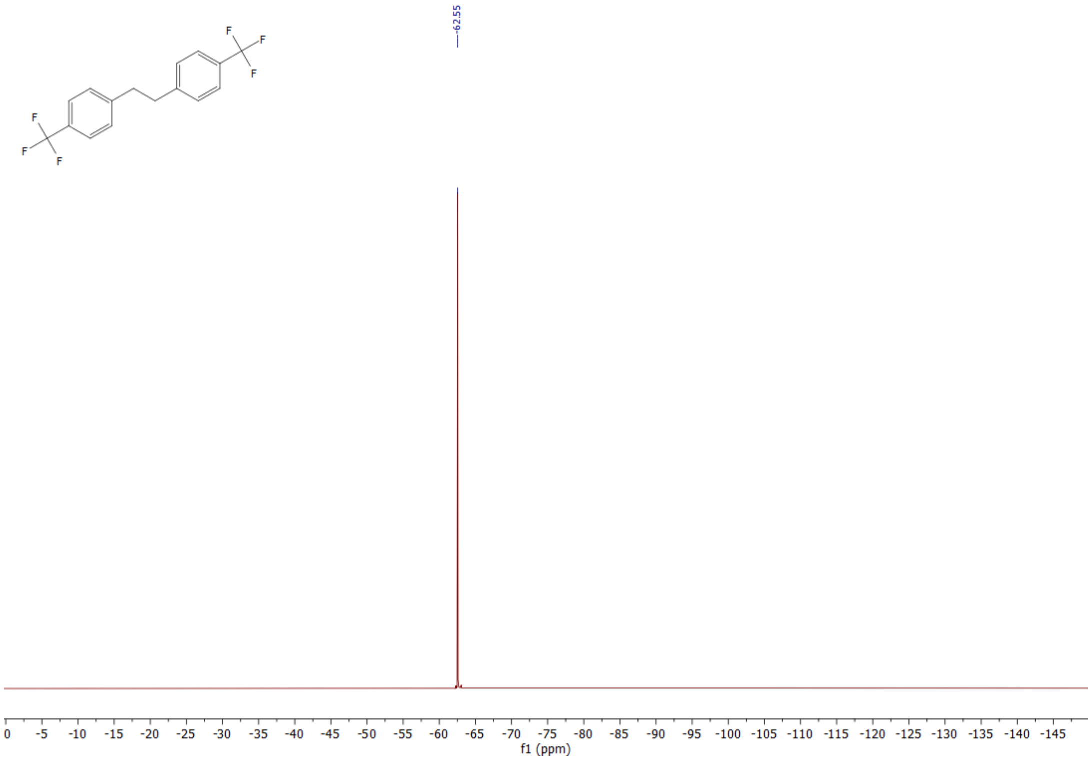

Figure S150. ^{19}F NMR spectrum of **5b** (376 MHz, CD_2Cl_2).

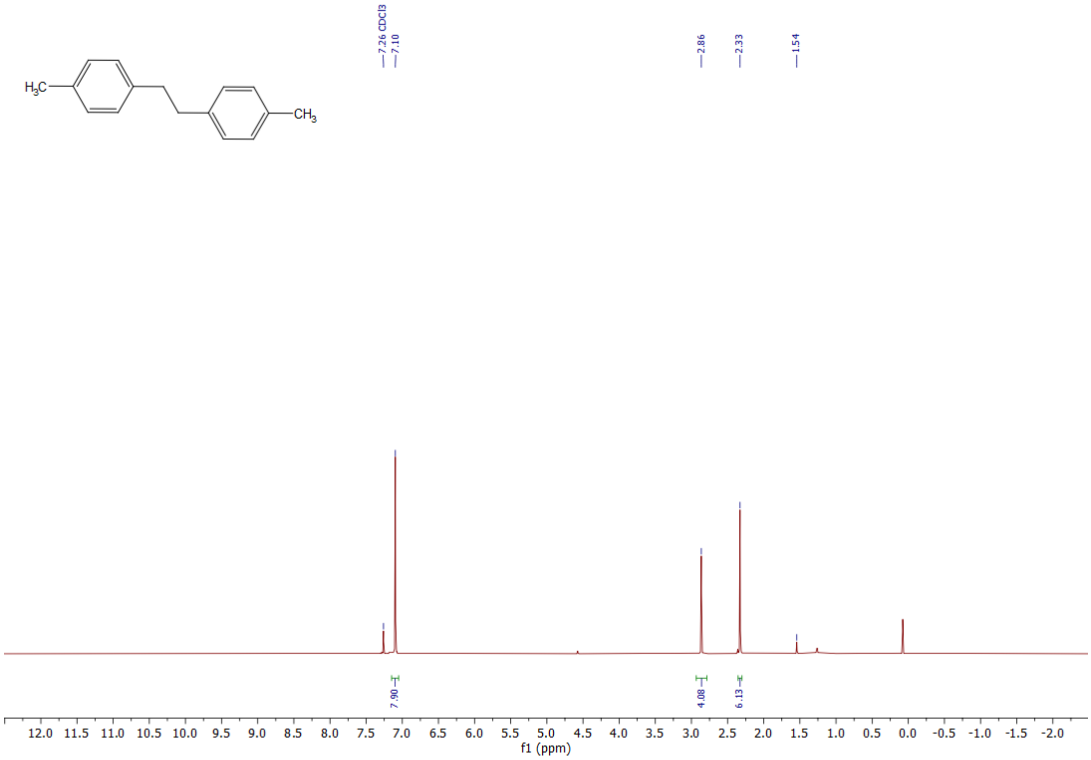

Figure S151. ^{1}H NMR spectrum of **6b** (400 MHz, $CDCl_3$).

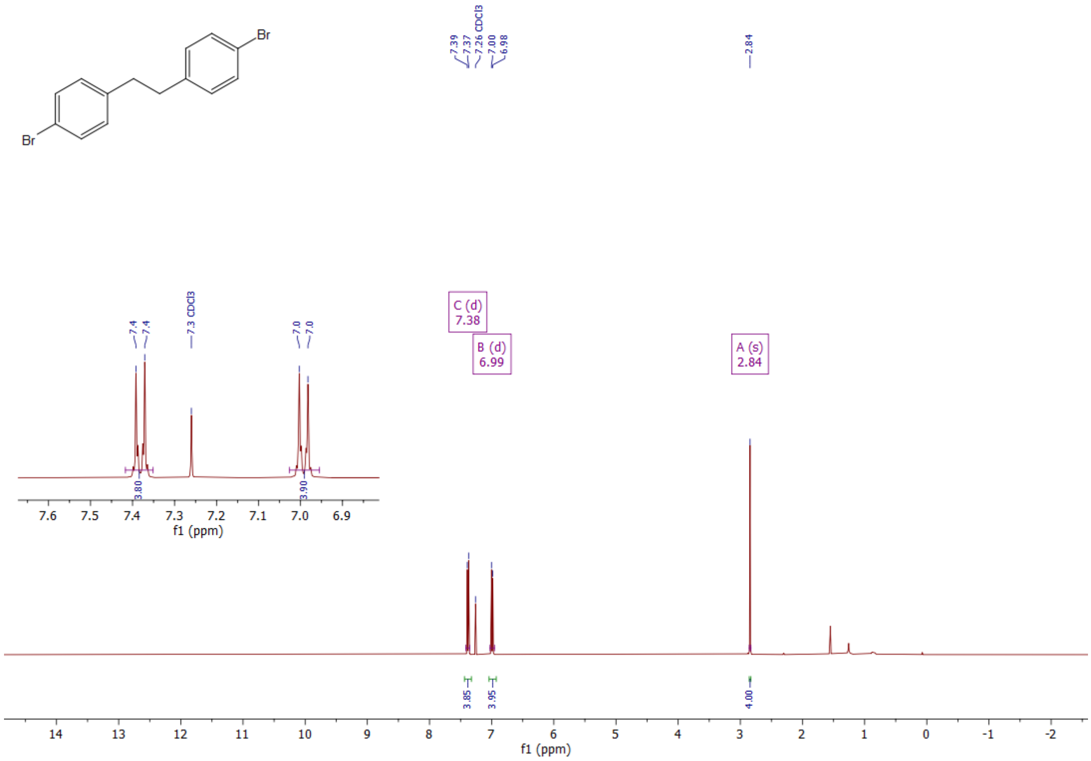

Figure S152. ^{1}H NMR spectrum of **7b** (400 MHz, $CDCl_3$).

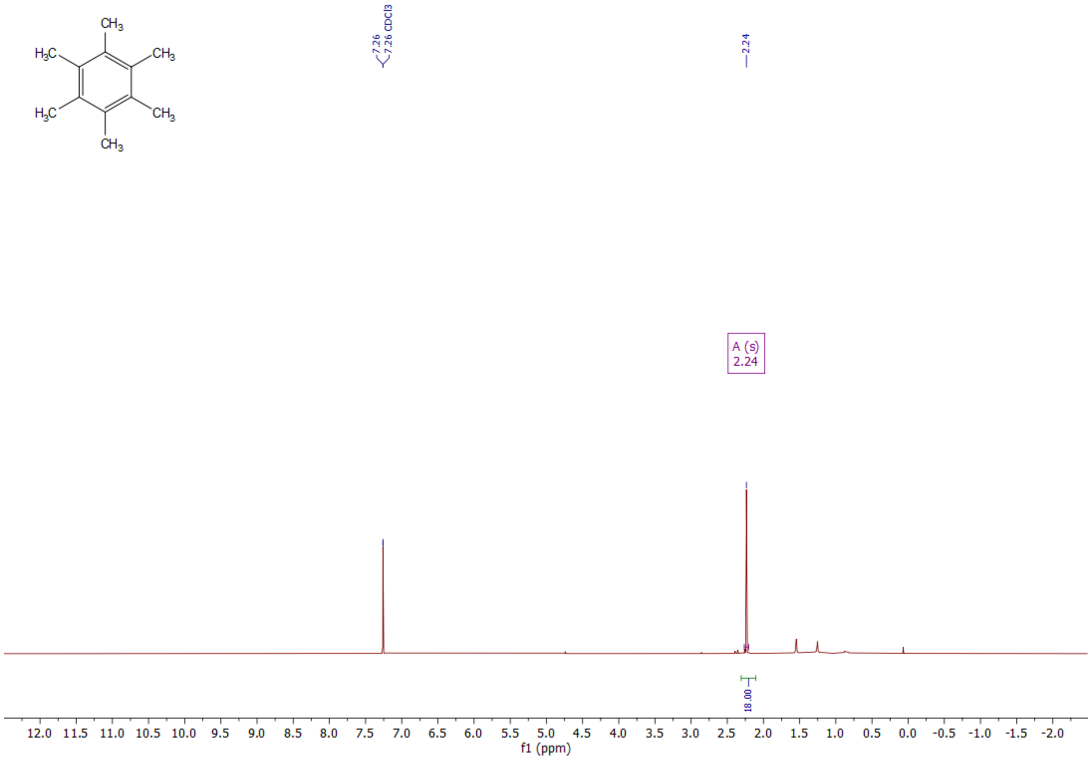

Figure S153. ^{1}H NMR spectrum of **8c** (400 MHz, $CDCl_3$).

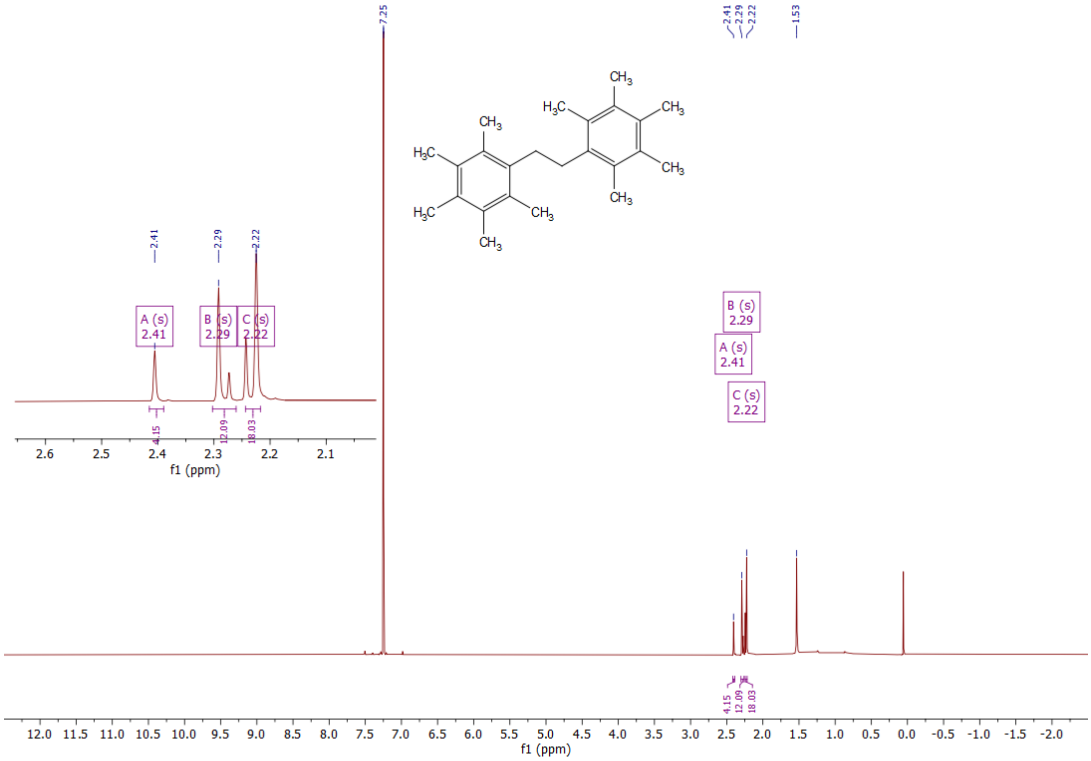

Figure S154. ^{1}H NMR spectrum of **8b** (400 MHz, $CDCl_3$).

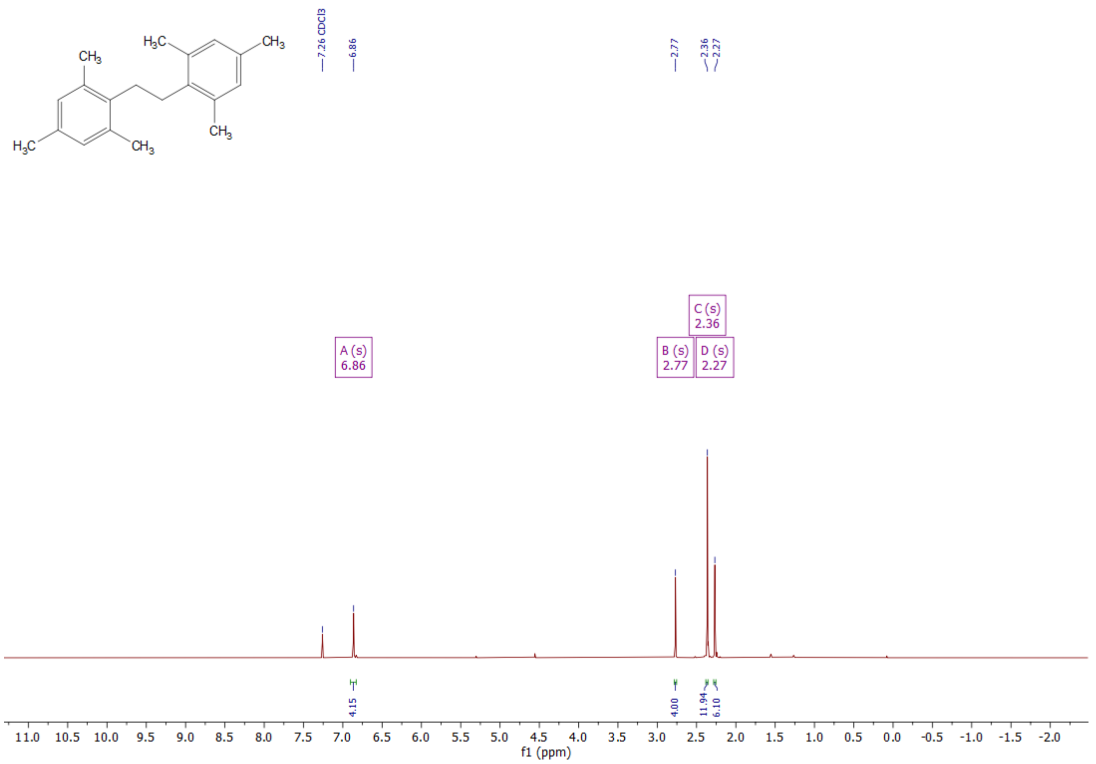

Figure S155. ${}^{1}H$ NMR spectrum of **9b** (400 MHz, $CDCl_3$).

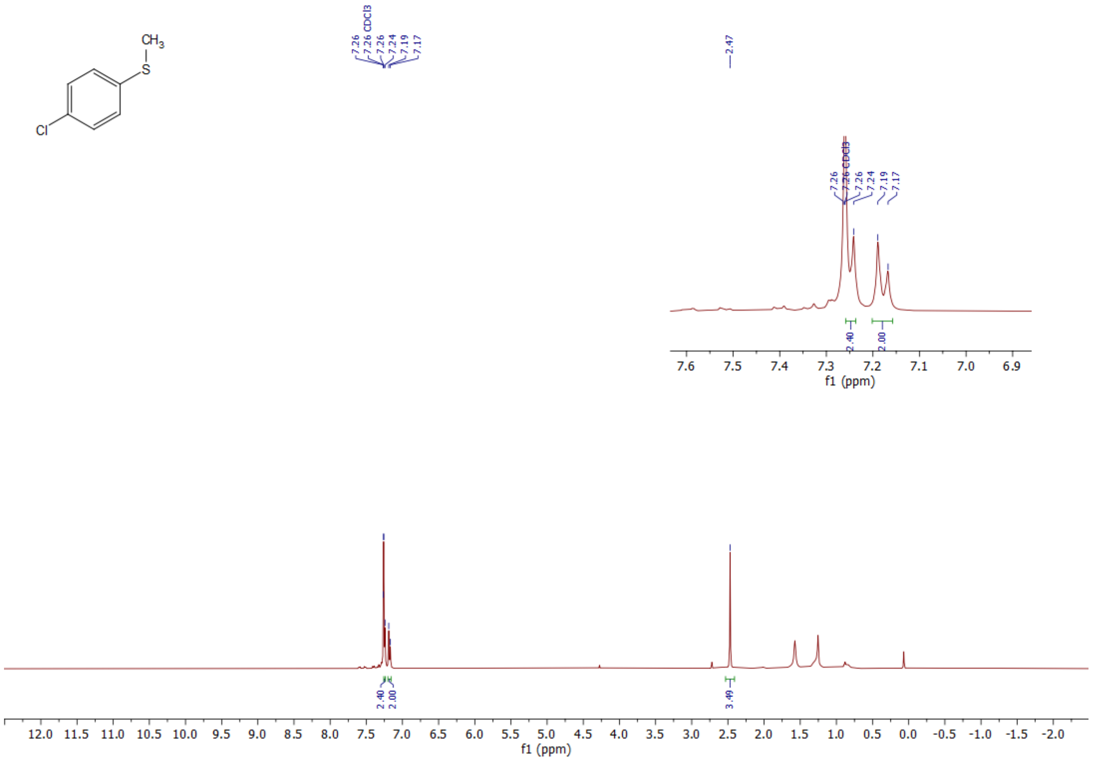

Figure S156. ${}^{1}H$ NMR spectrum of **10c** (400 MHz, $CDCl_3$).

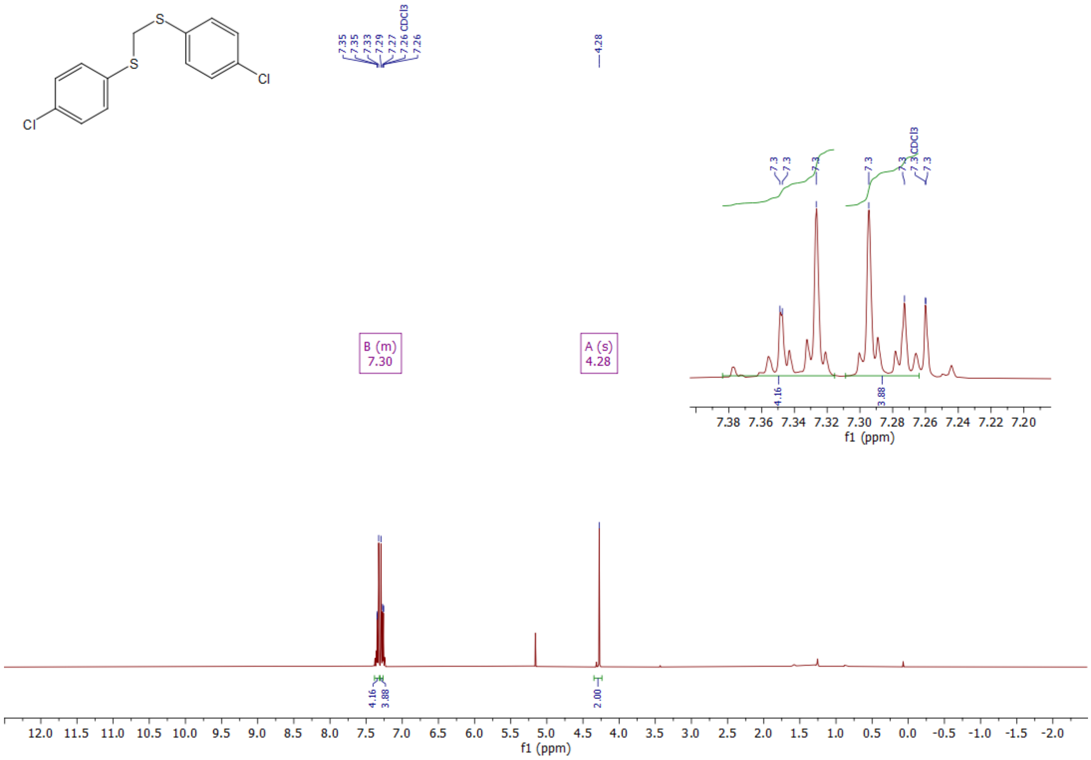

Figure S157. ^{1}H NMR spectrum of **10c** (400 MHz, $CDCl_3$).

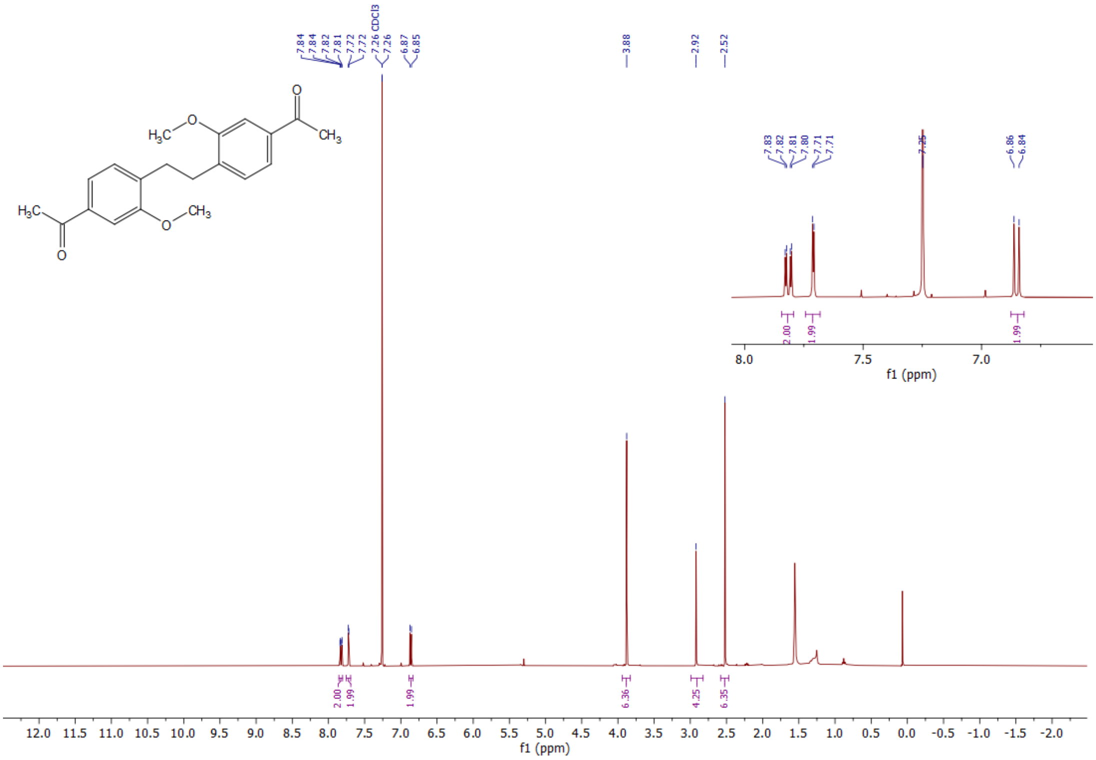

Figure S158. ^{1}H NMR spectrum of **11b** (400 MHz, $CDCl_3$).

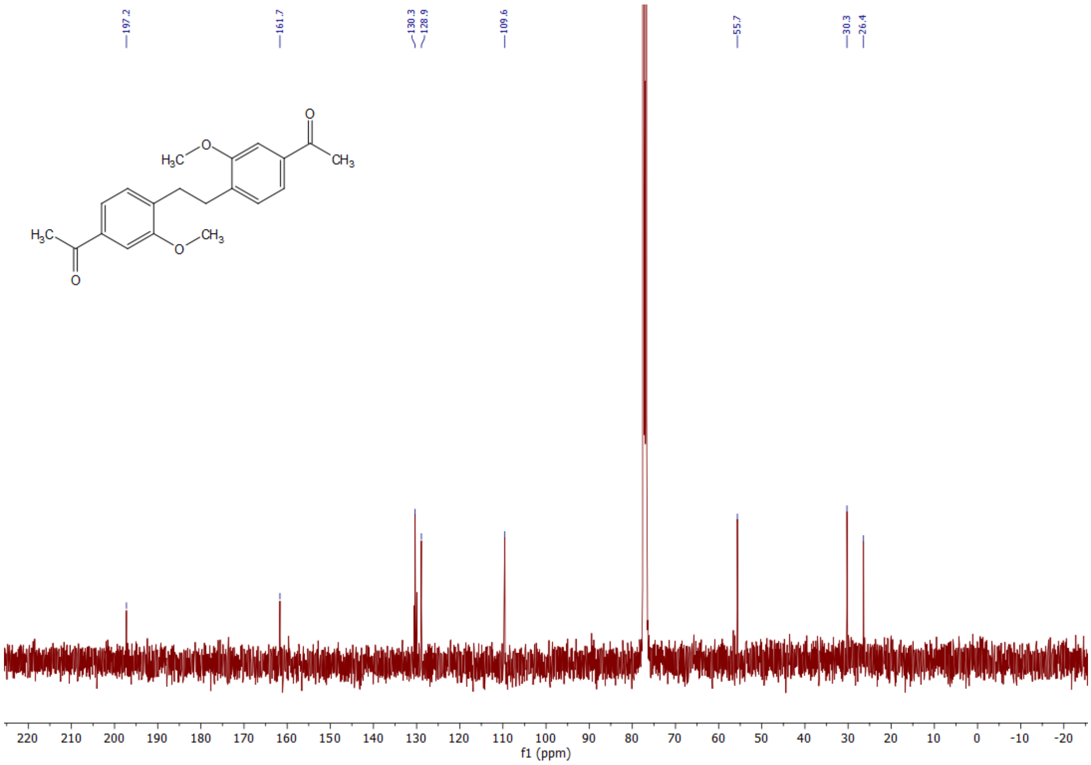

Figure S159. ^{13}C NMR spectrum of **11b** (101 MHz, $CDCl_3$).

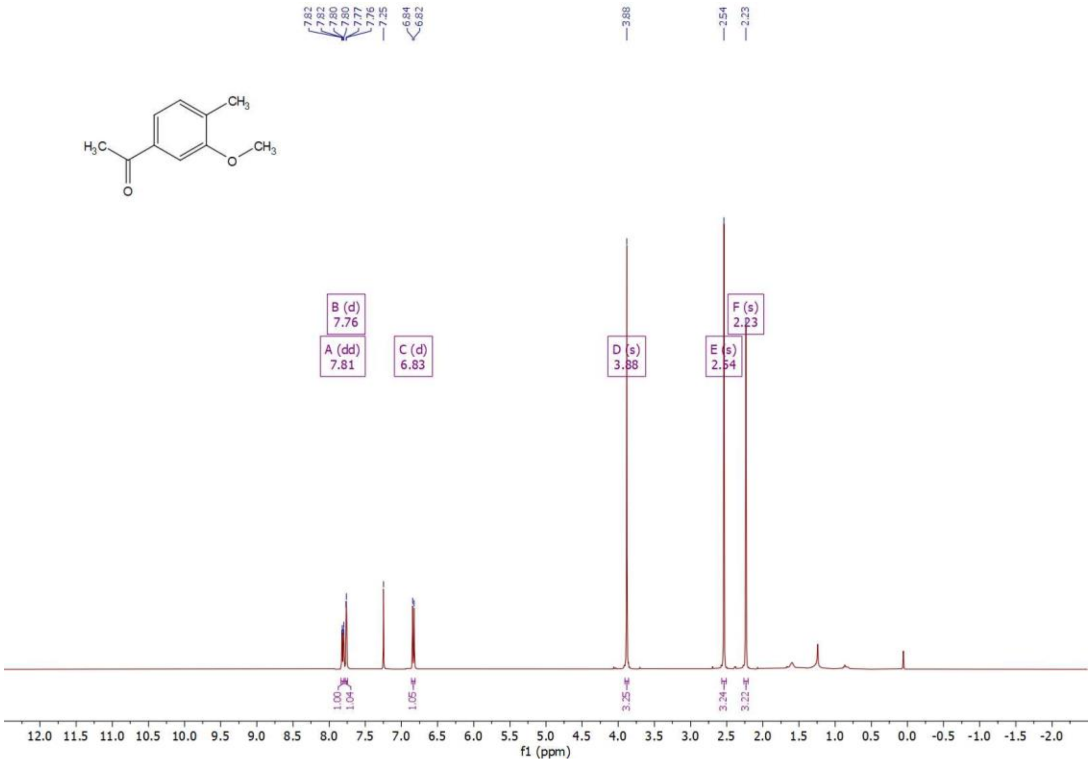

Figure S160. ^{1}H NMR spectrum of **11c** (400 MHz, $CDCl_3$).

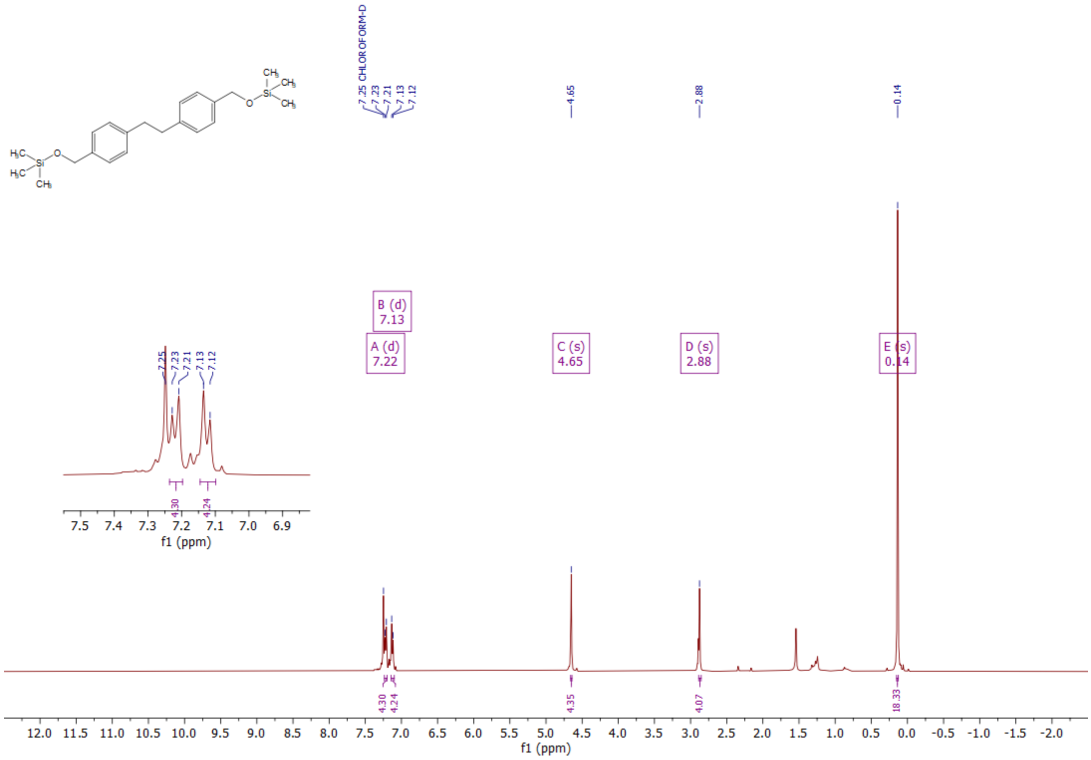

Figure S161. ^{1}H NMR spectrum of **12b** (400 MHz, $CDCl_3$).

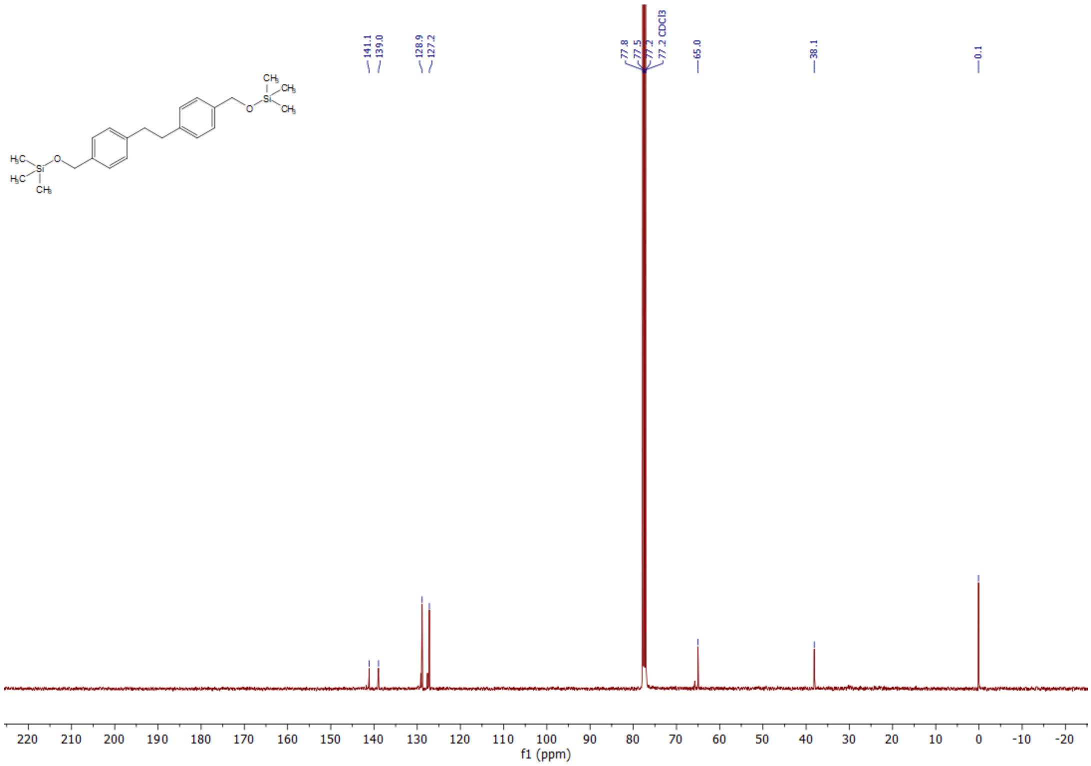

Figure S162. ^{13}C NMR spectrum of **12b** (101 MHz, $CDCl_3$).

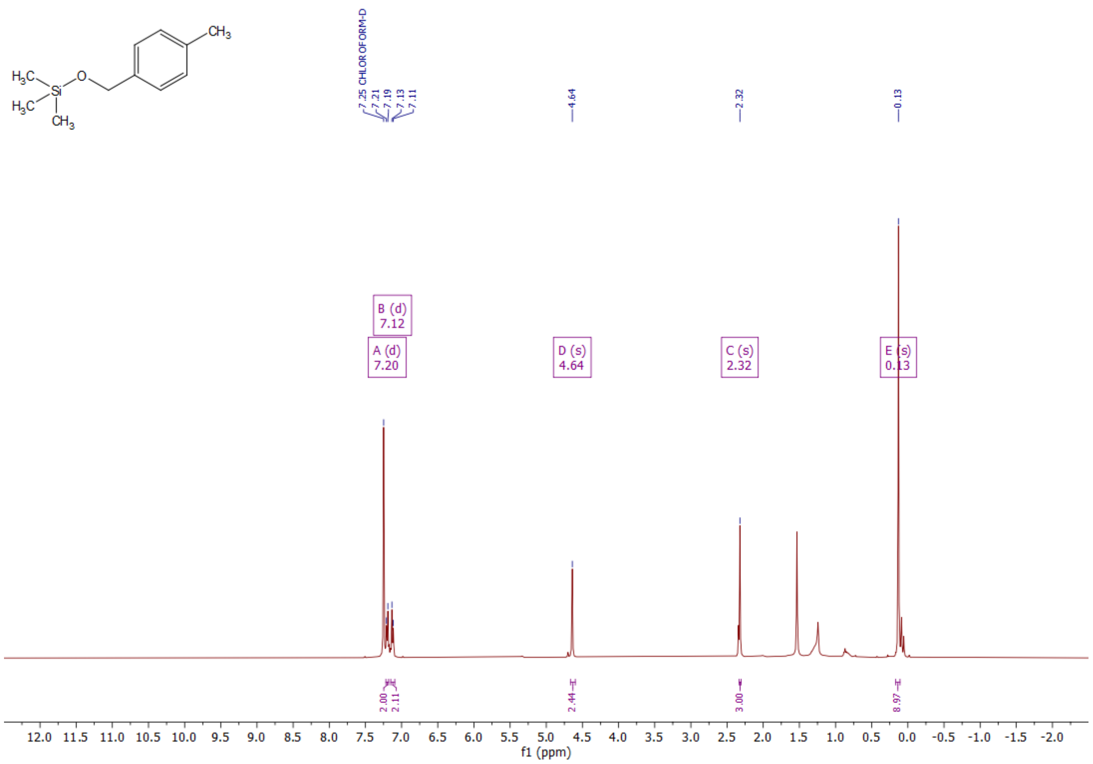

Figure S163. ^{1}H NMR spectrum of **12c** (400 MHz, $CDCl_3$).

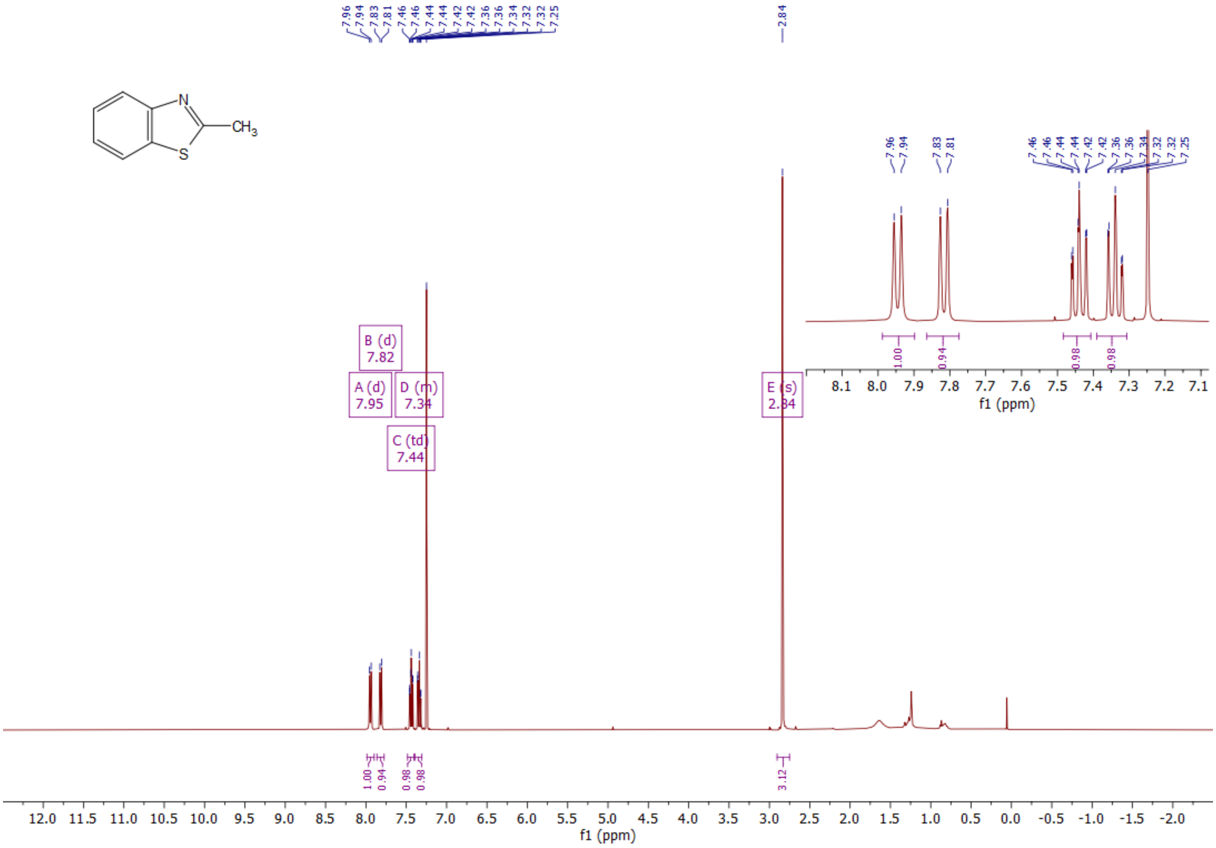

Figure S164. ^{1}H NMR spectrum of **15c** (400 MHz, $CDCl_3$).

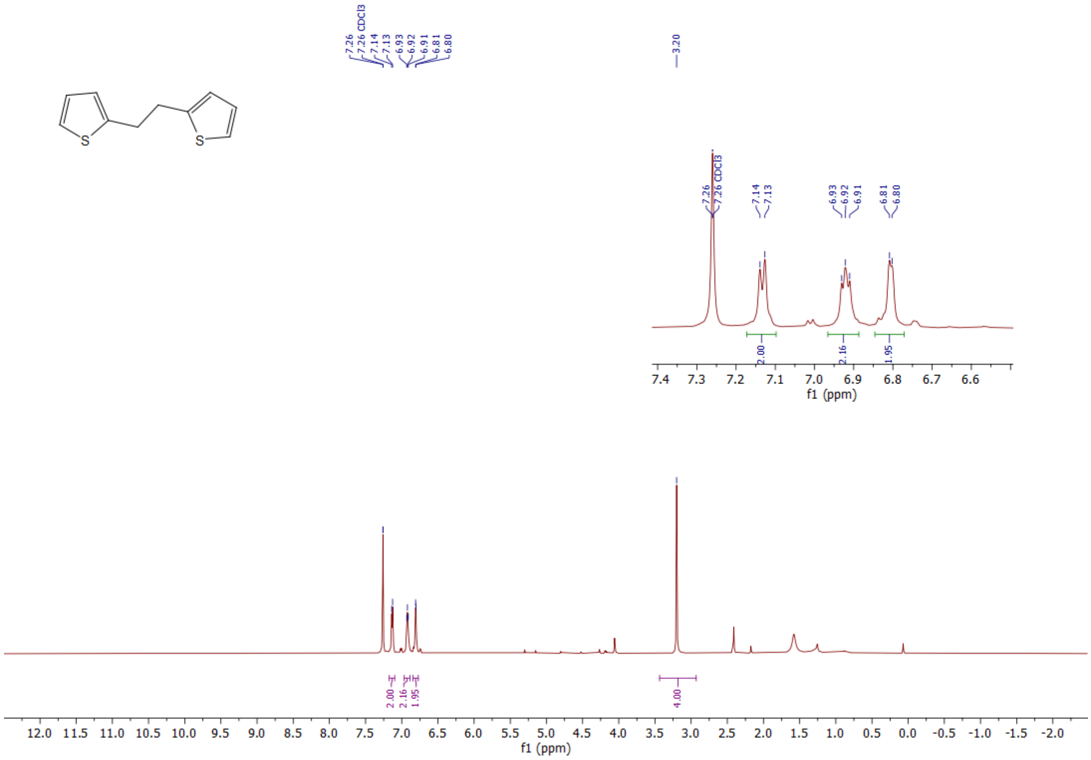

Figure S165. ^{1}H NMR spectrum of **16b** (400 MHz, $CDCl_3$).

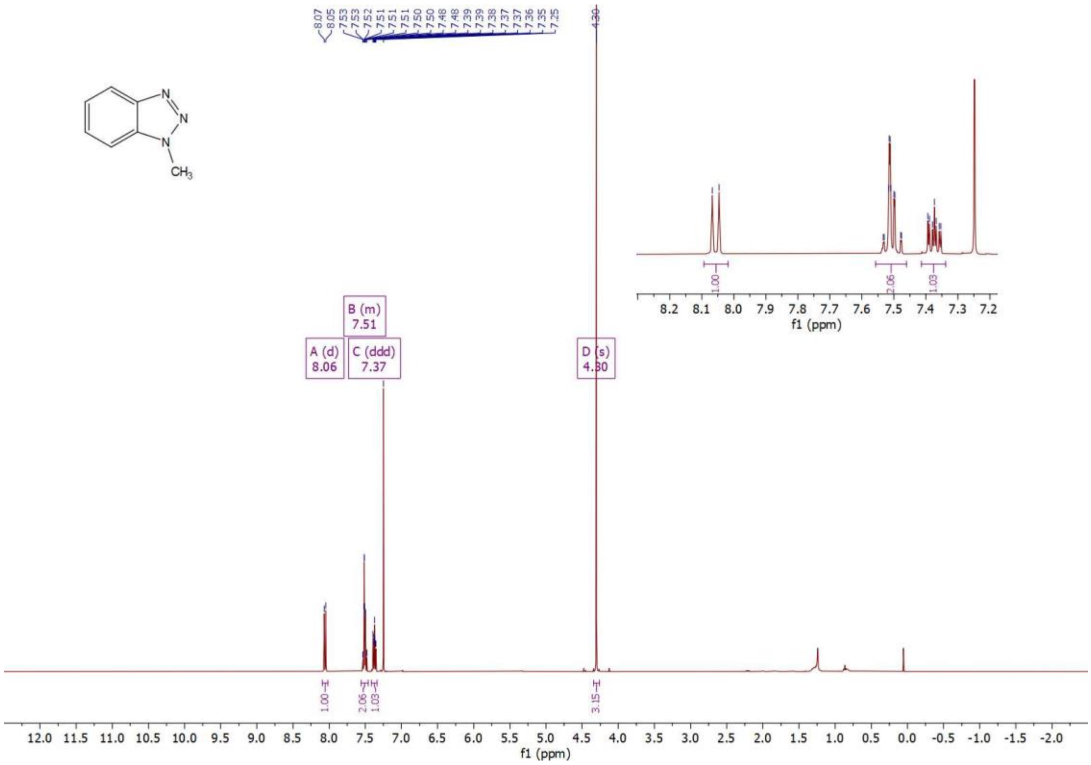

Figure S166. ^{1}H NMR spectrum of **17c** (400 MHz, $CDCl_3$).

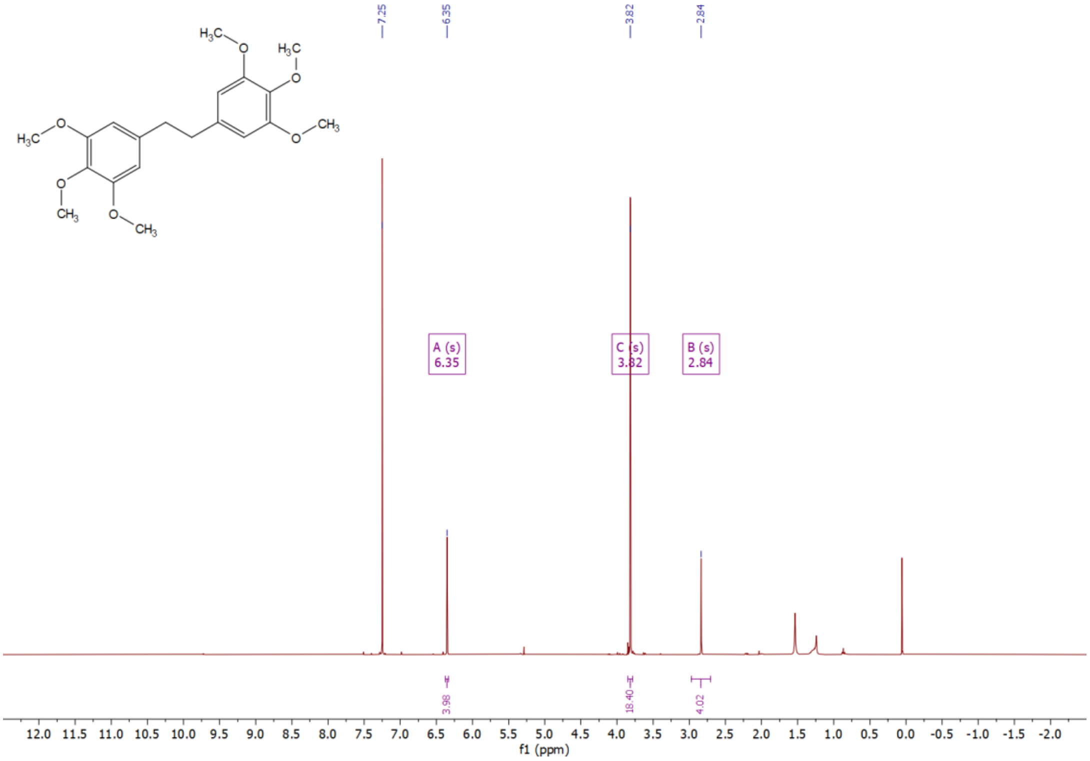

Figure S167. ^{1}H NMR spectrum of Brittonin A **(18b)** (400 MHz, $CDCl_3$).

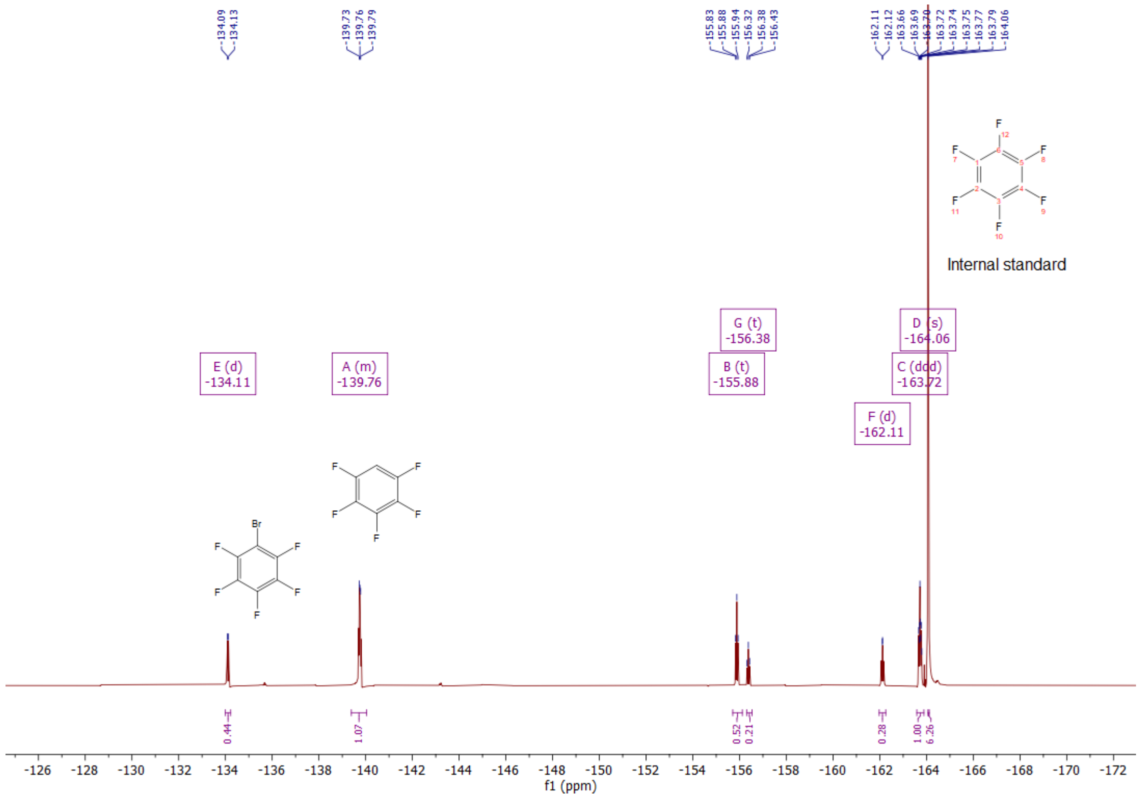

Figure S168. ^{19}F NMR spectrum of reaction mixture of **19b** (Entry 1) (376 MHz, Benzene-d_6).

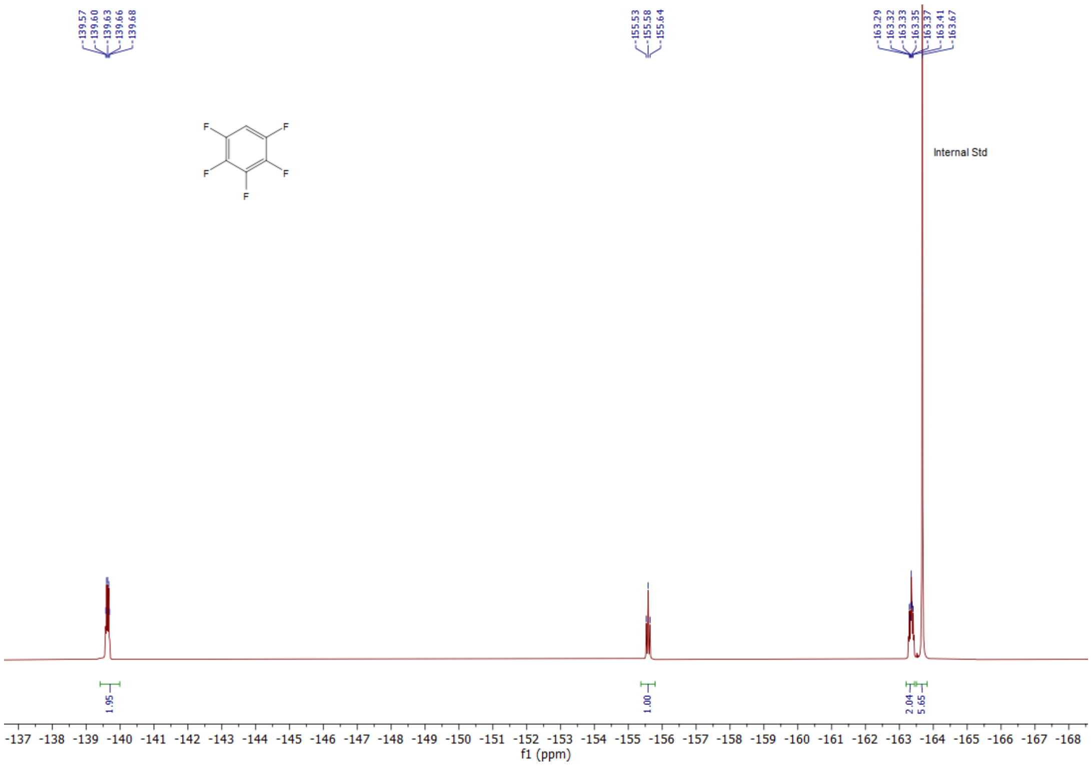

Figure S169. ^{19}F NMR spectrum of reaction mixture of **19b** (Entry 2) (376 MHz, Benzene-d_6).

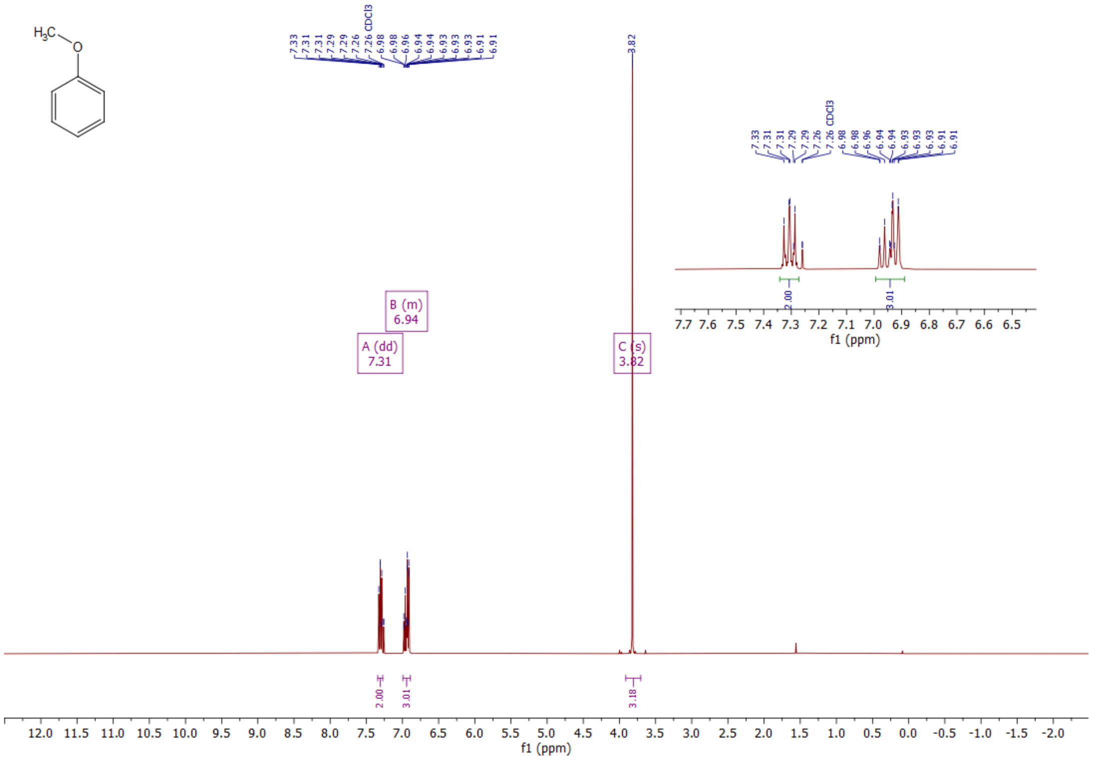

Figure S170. ^{1}H NMR spectrum of **20b** (400 MHz, $CDCl_3$).

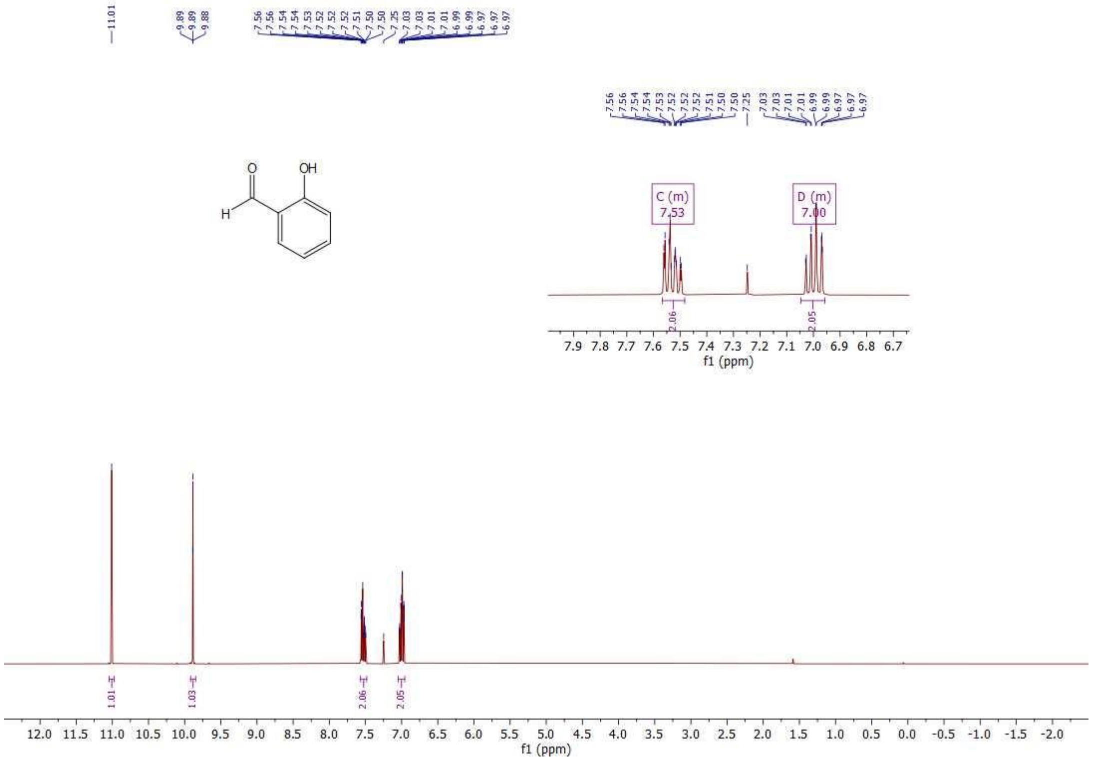

Figure S171. ^{1}H NMR spectrum of **21b** (400 MHz, $CDCl_3$).

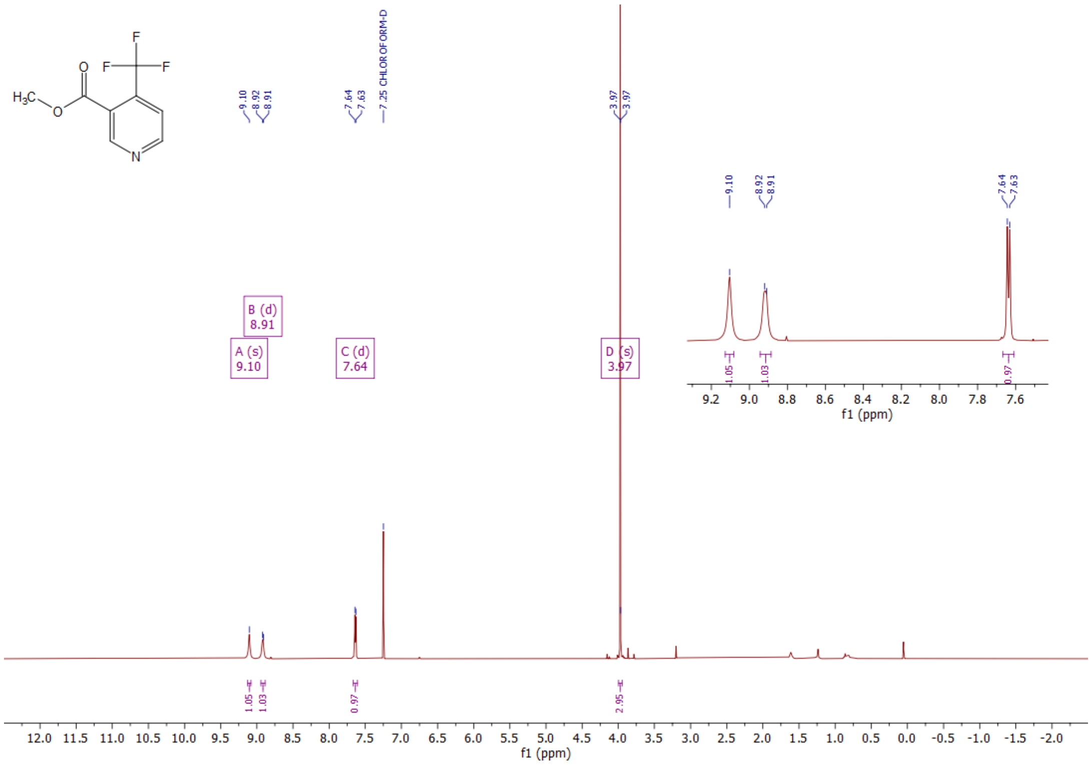

Figure S172. ^{1}H NMR spectrum of **22b** (400 MHz, $CDCl_3$).

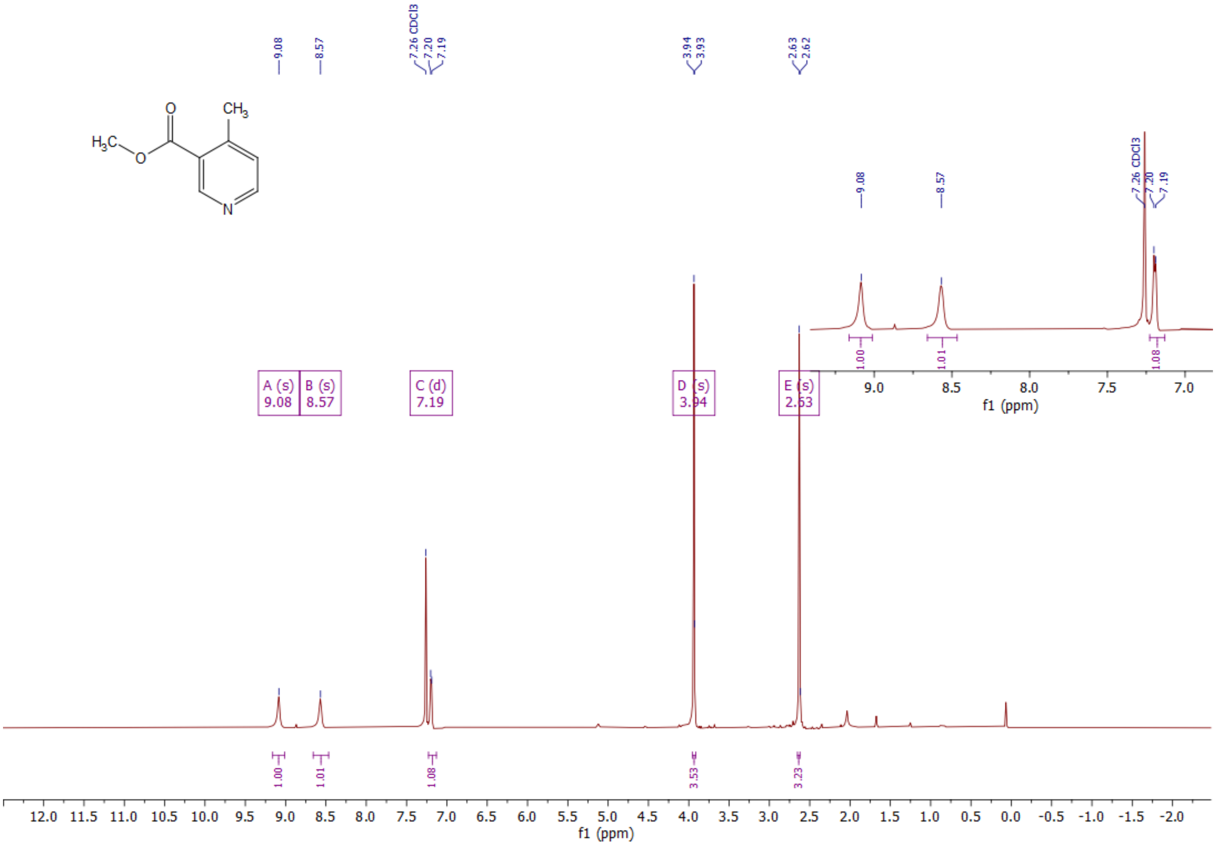

Figure S173. ^{1}H NMR spectrum of **23b** (400 MHz, $CDCl_3$).

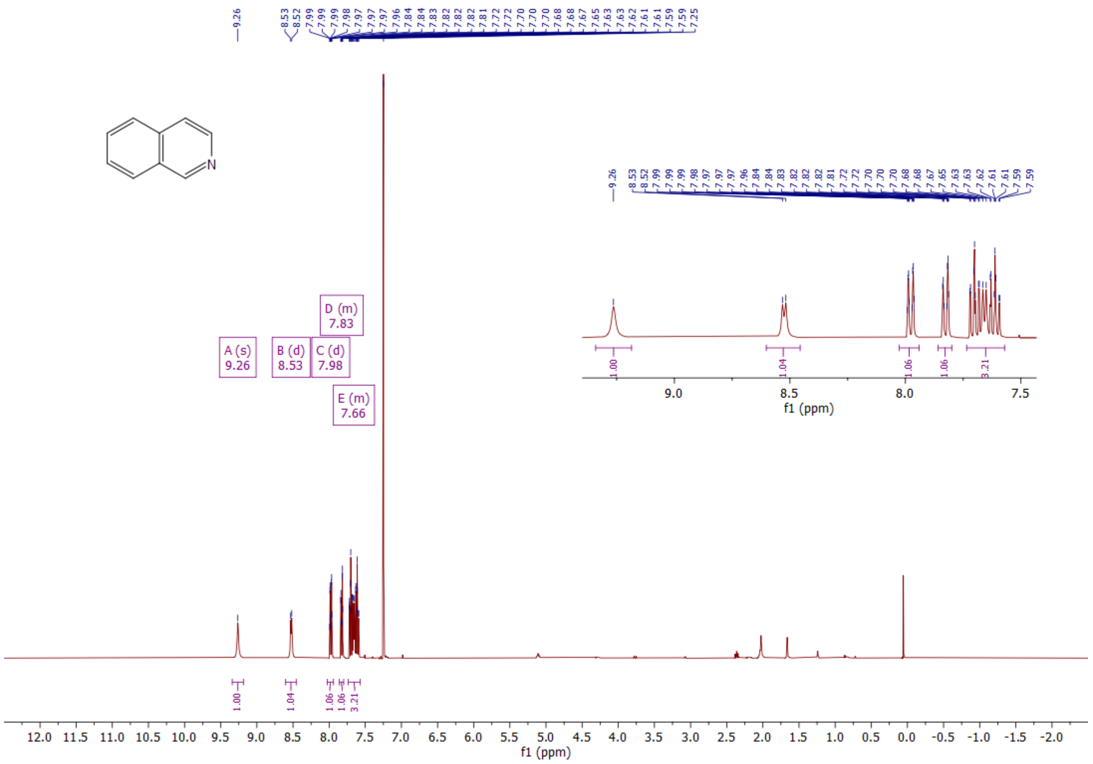

Figure S174. ^{1}H NMR spectrum of **24b** (400 MHz, $CDCl_3$).

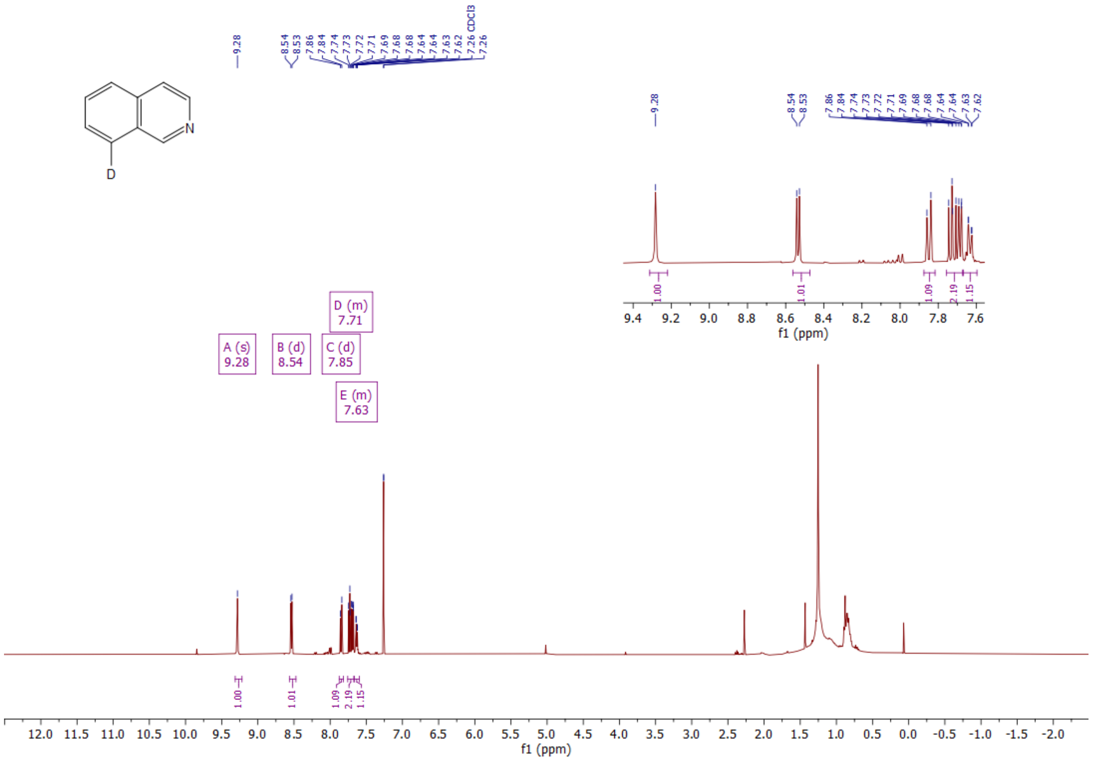

Figure S175. ^{1}H NMR spectrum of **24c** (400 MHz, $CDCl_3$).

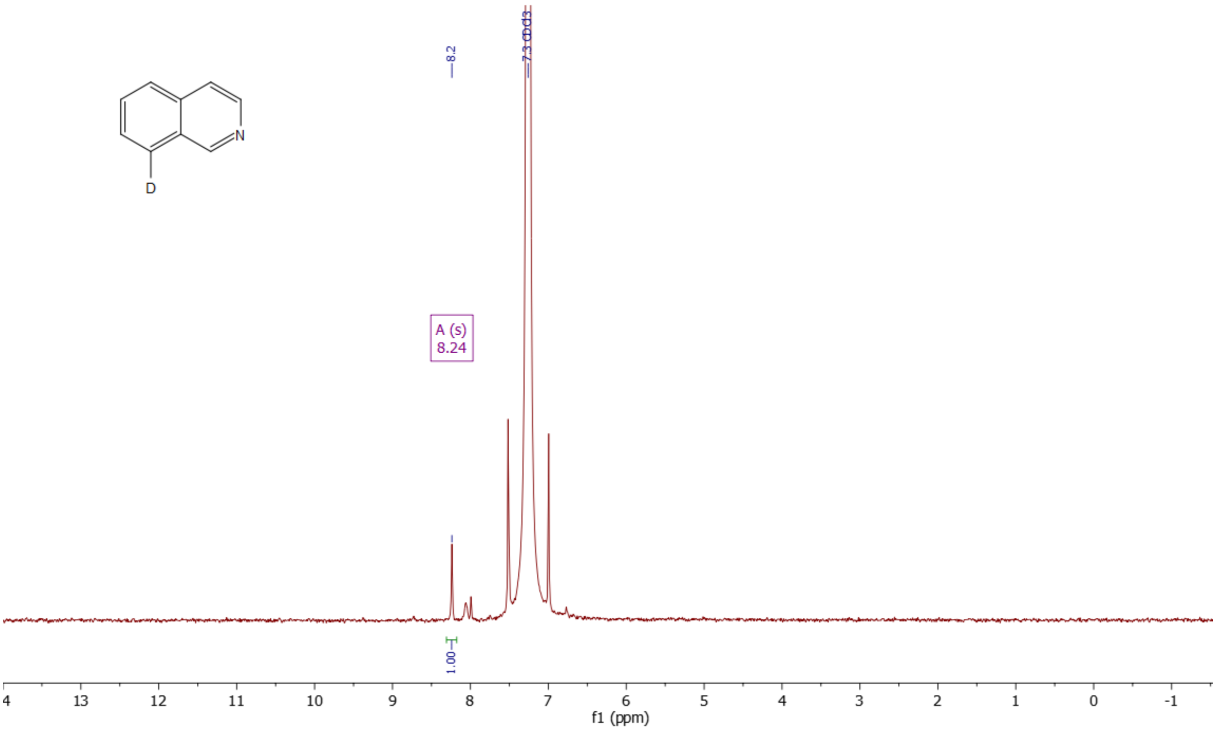

Figure S176. ^{2}H NMR spectrum of **24c** (61 MHz, $CDCl_3$).

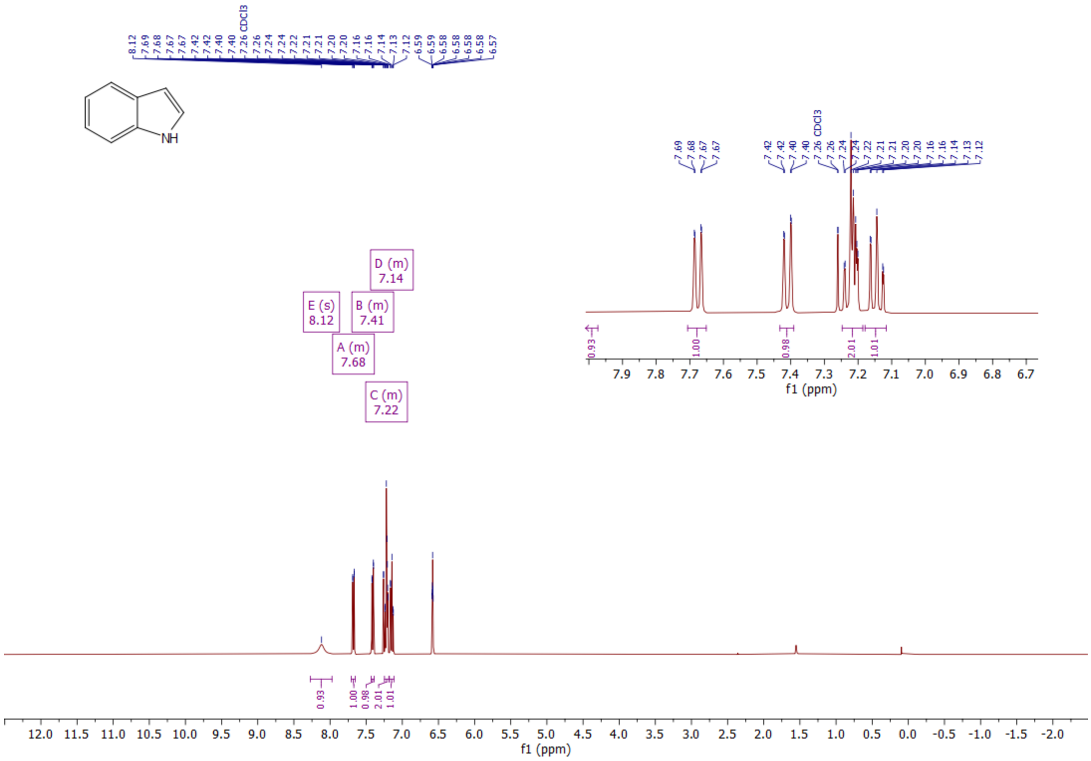

Figure S177. ^{1}H NMR spectrum of **25b** (400 MHz, $CDCl_3$).

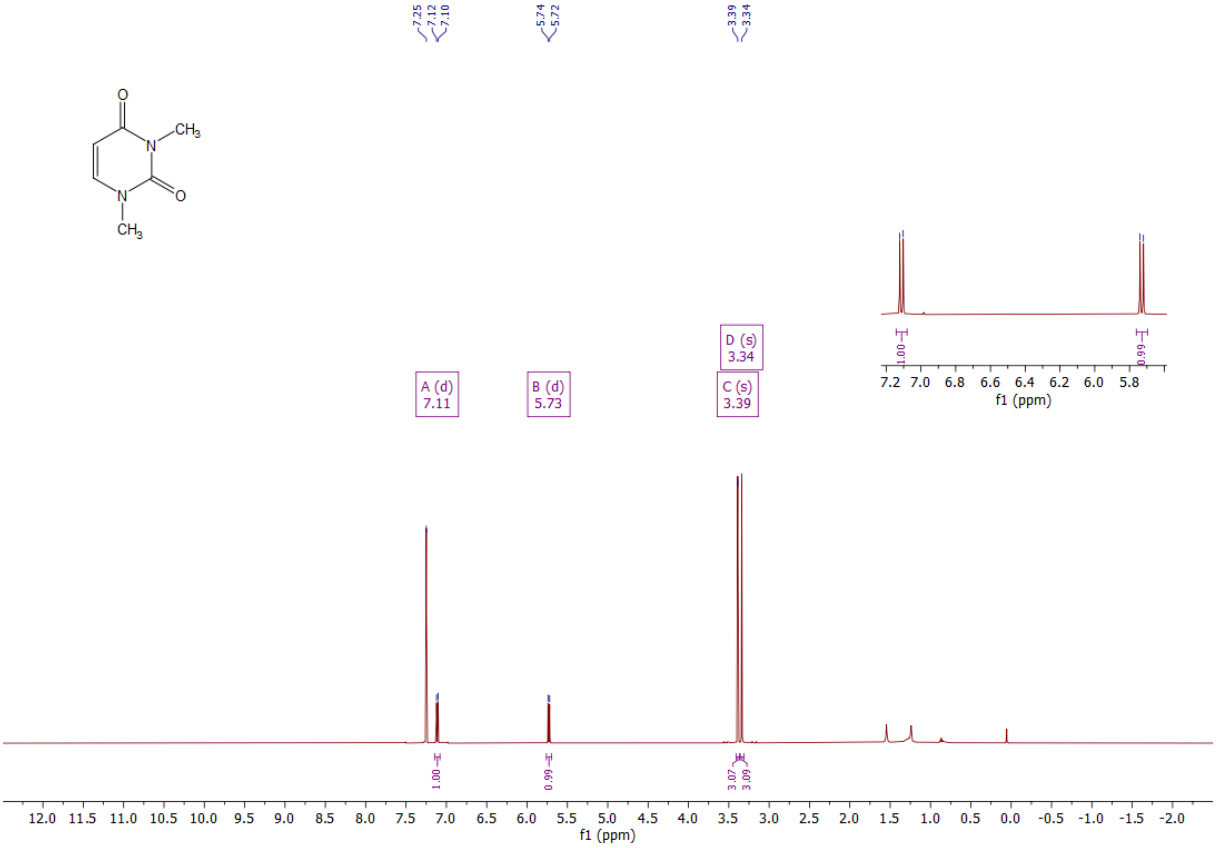

Figure S178. ^{1}H NMR spectrum of **26b** (400 MHz, $CDCl_3$).

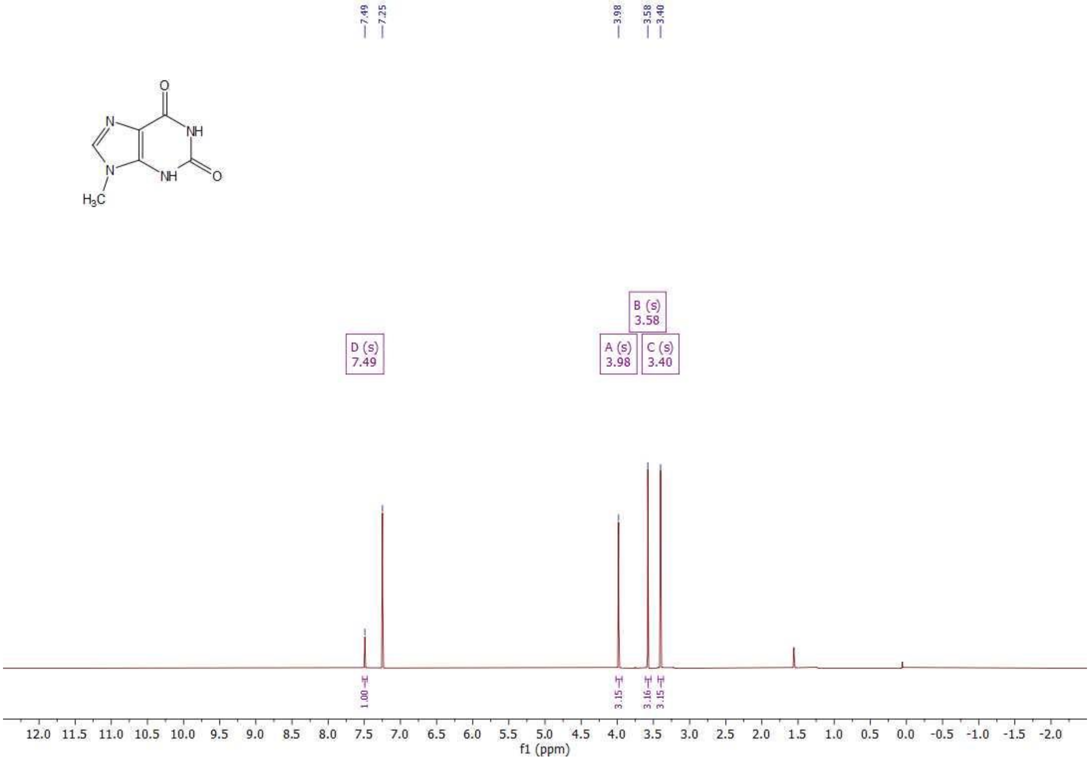

Figure S179. ^{1}H NMR spectrum of **27b** (400 MHz, $CDCl_3$).

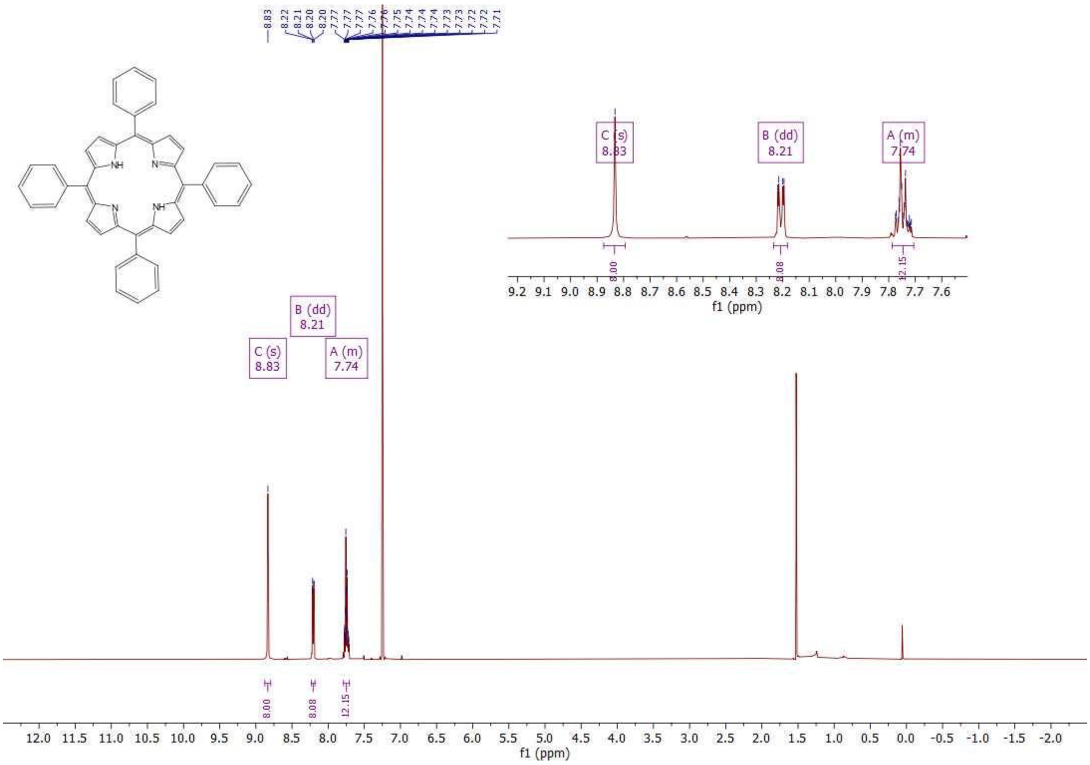

Figure S180. ^{1}H NMR spectrum of **28b** (400 MHz, $CDCl_3$).

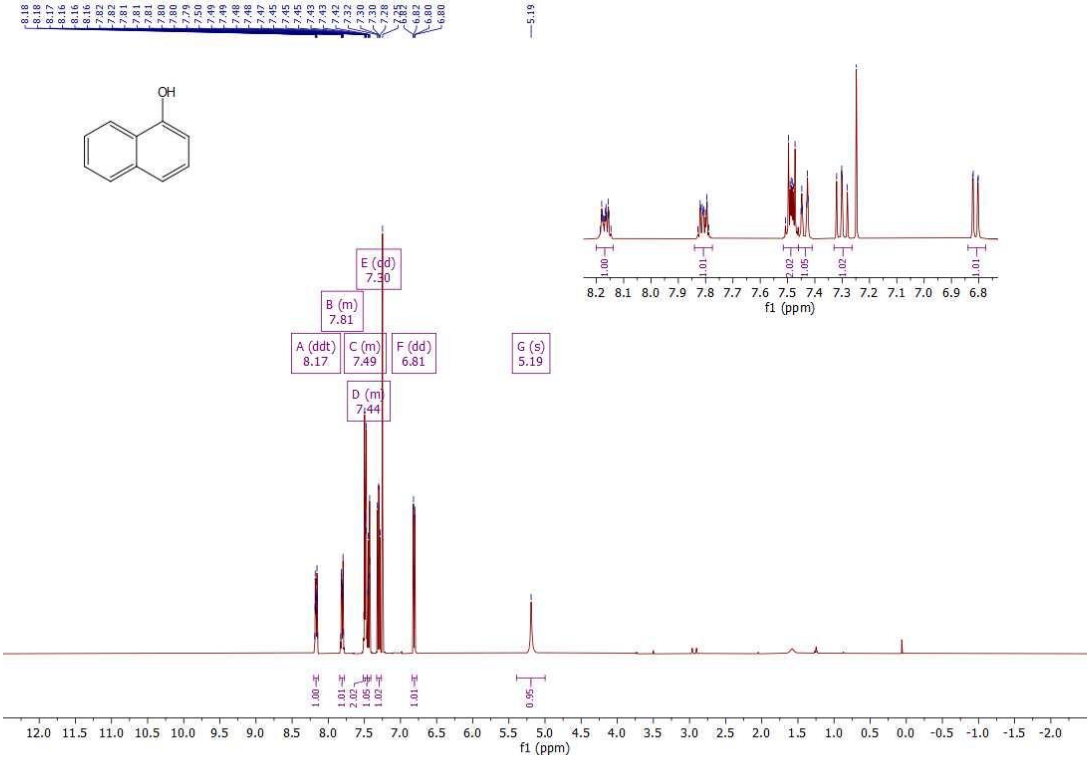

Figure S181. ^{1}H NMR spectrum of **29b** (400 MHz, $CDCl_3$).

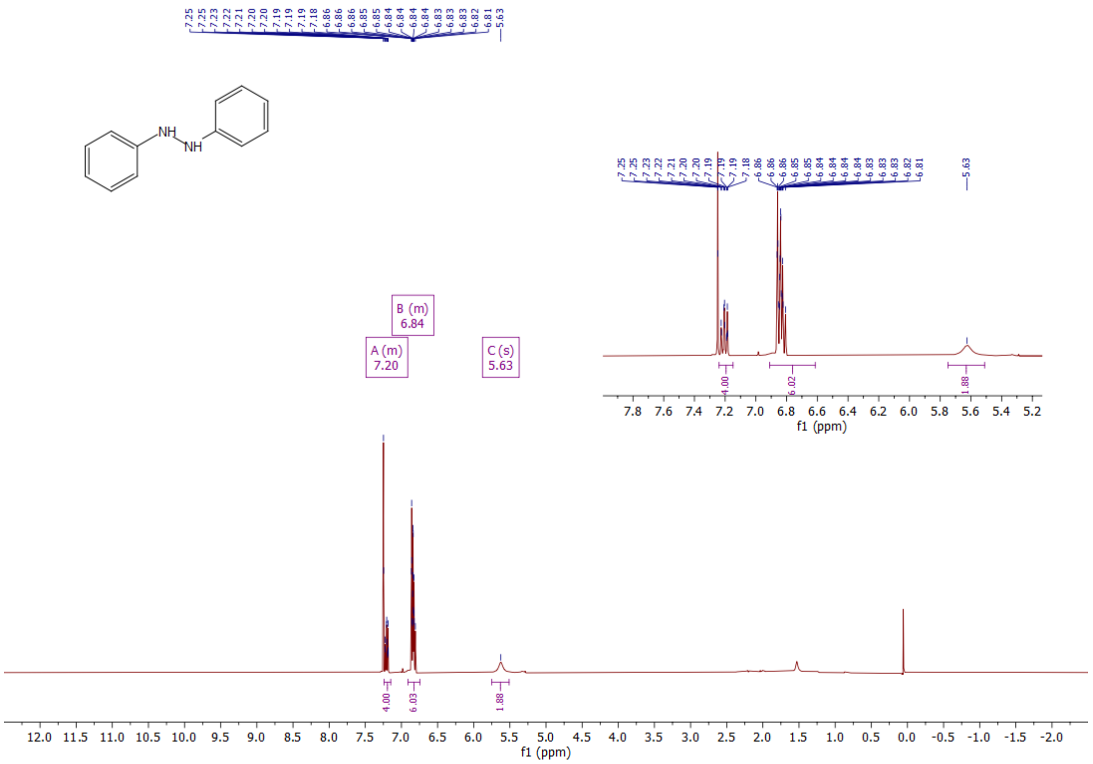

Figure S182. ^{1}H NMR spectrum of **30b** (400 MHz, $CDCl_3$).

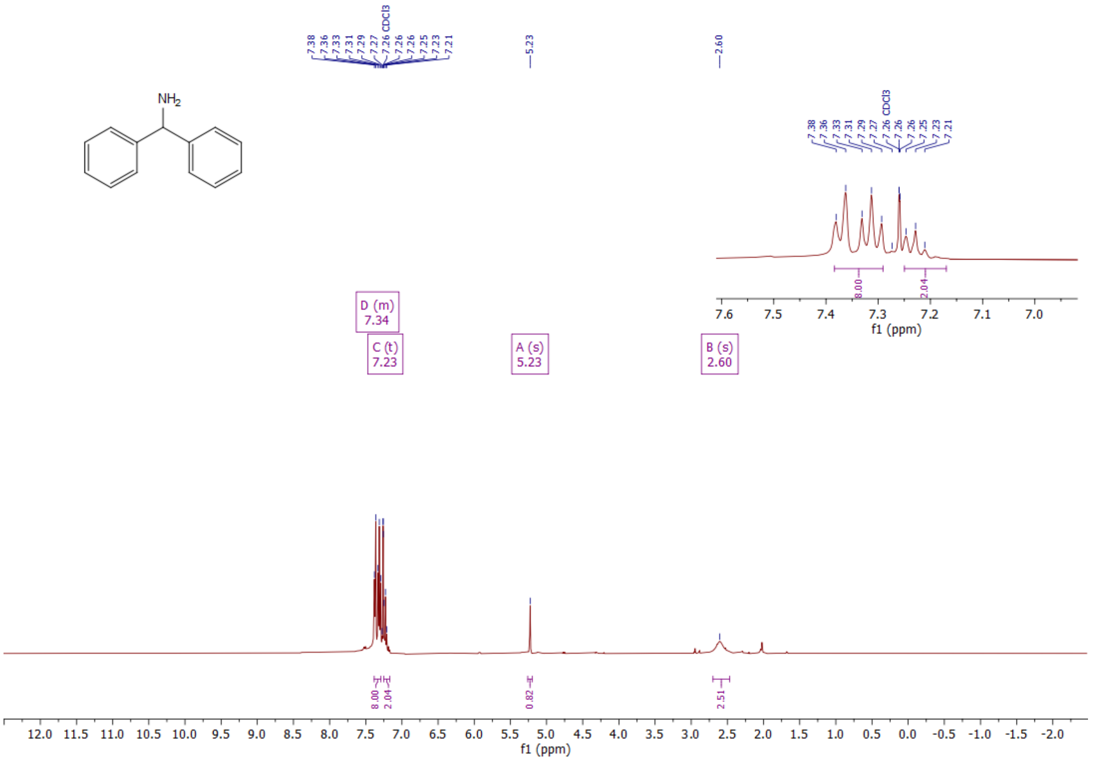

Figure S183. ^{1}H NMR spectrum of **31b** (400 MHz, $CDCl_3$).

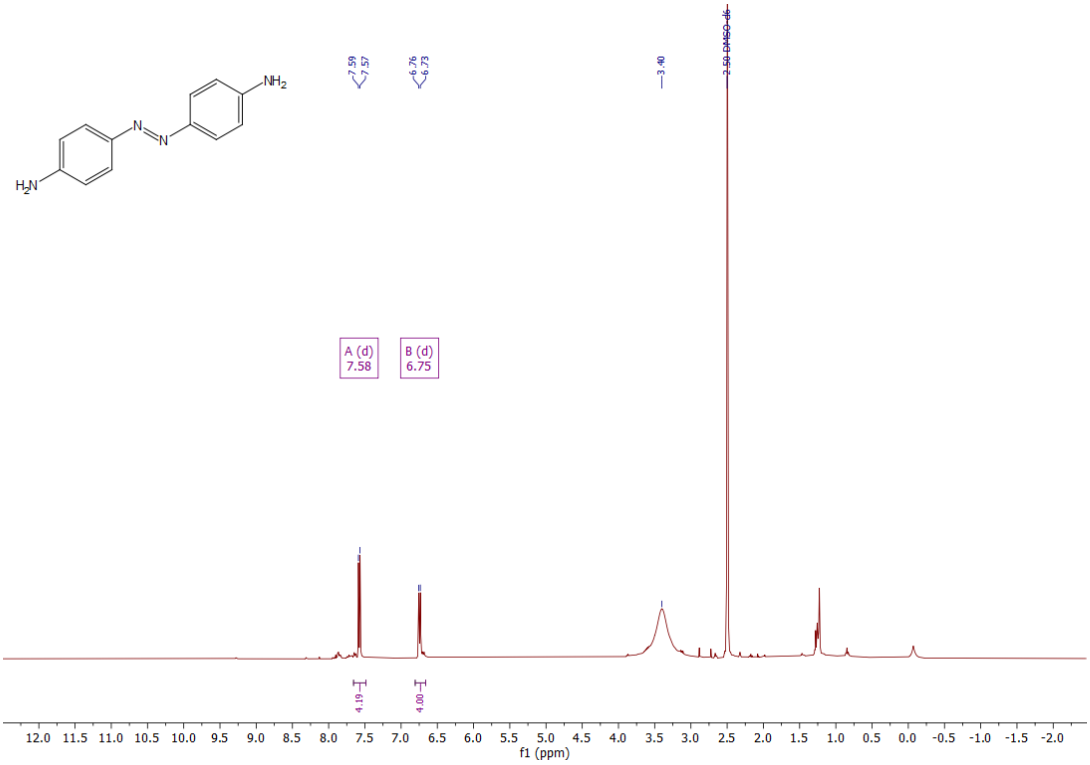

Figure S184. ^{1}H NMR spectrum of **32b** (400 MHz, $CDCl_3$).

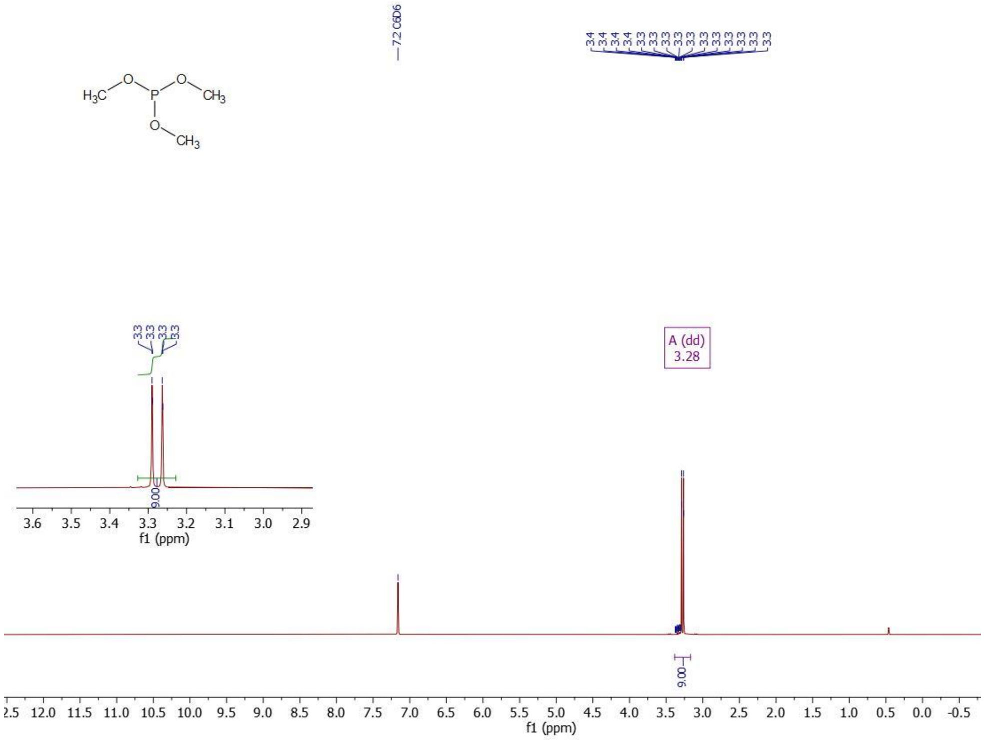

Figure S185. ^{1}H NMR spectrum of **33b** (162 MHz, Benzene-d_6).

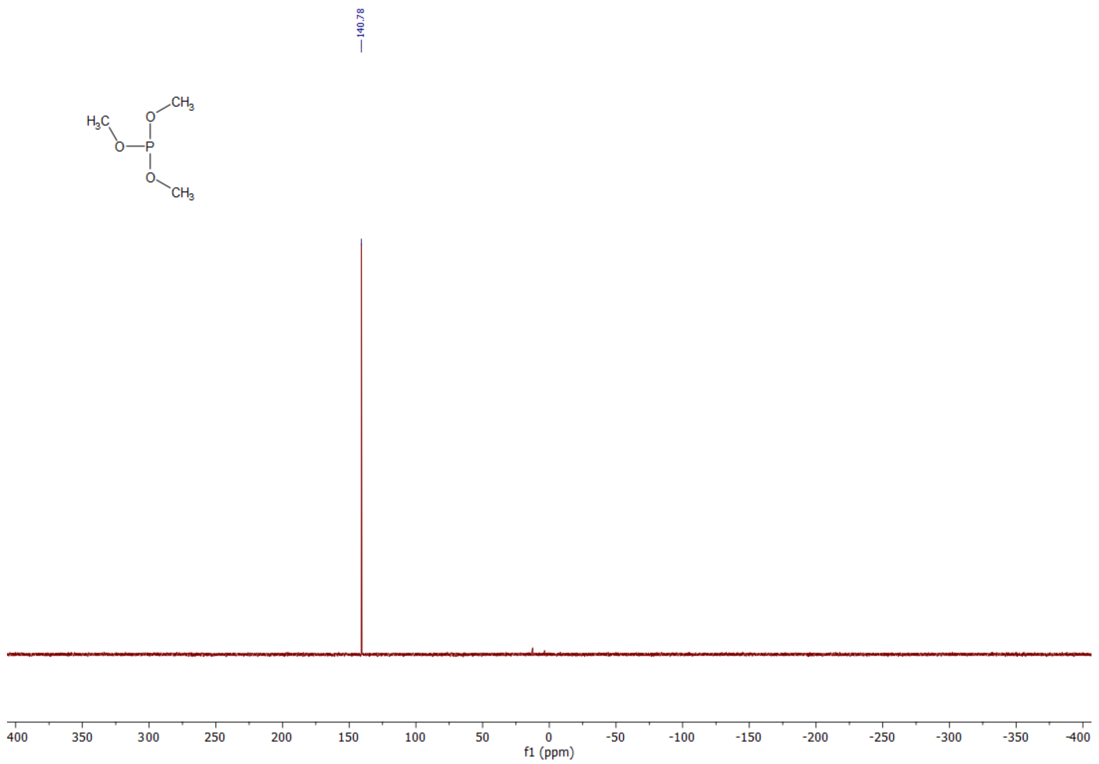

Figure S186. ^{31}P NMR spectrum of **34b** (162 MHz, Benzene-d_6).

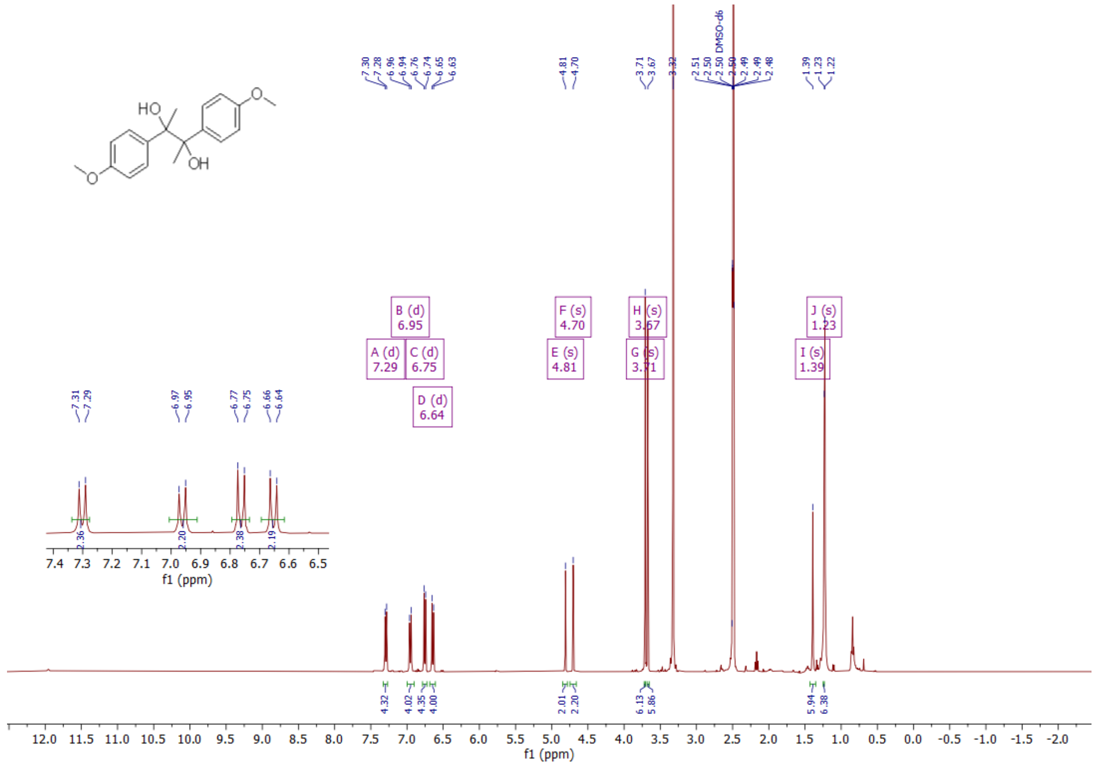

Figure S187. ^{1}H NMR spectrum of **35b** (400 MHz, DMSO-d_6).

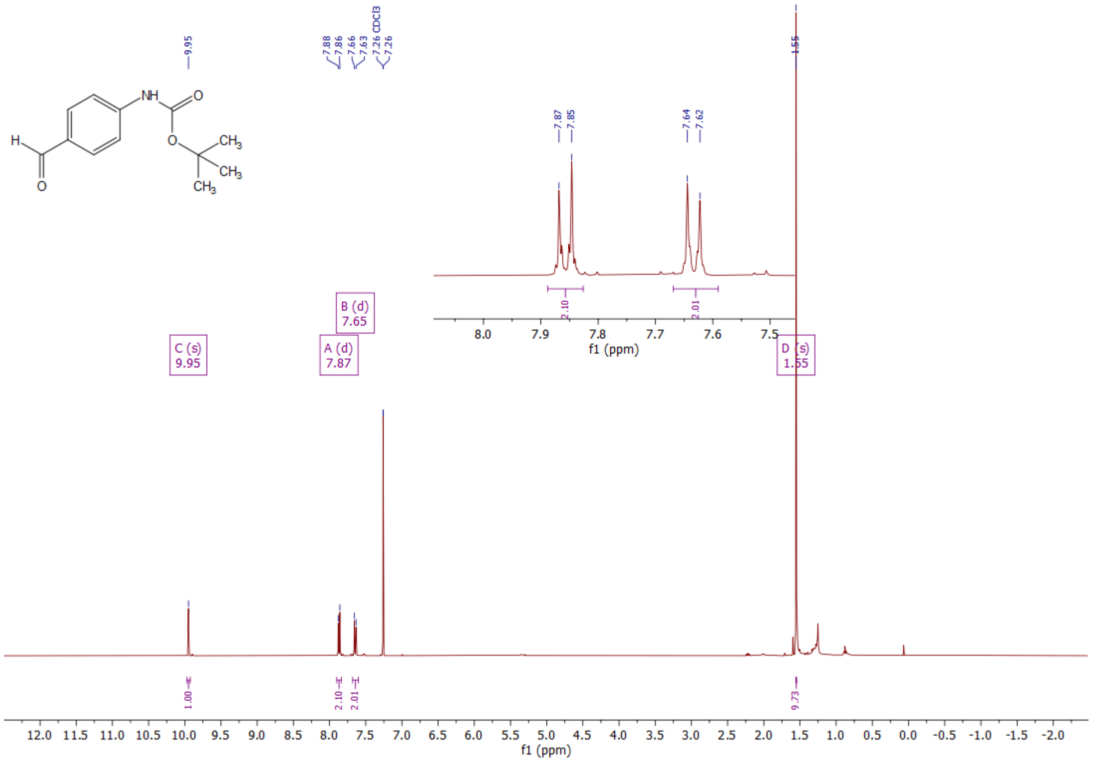

Figure S188. ^{1}H NMR spectrum of **36b** (400 MHz, $CDCl_3$).

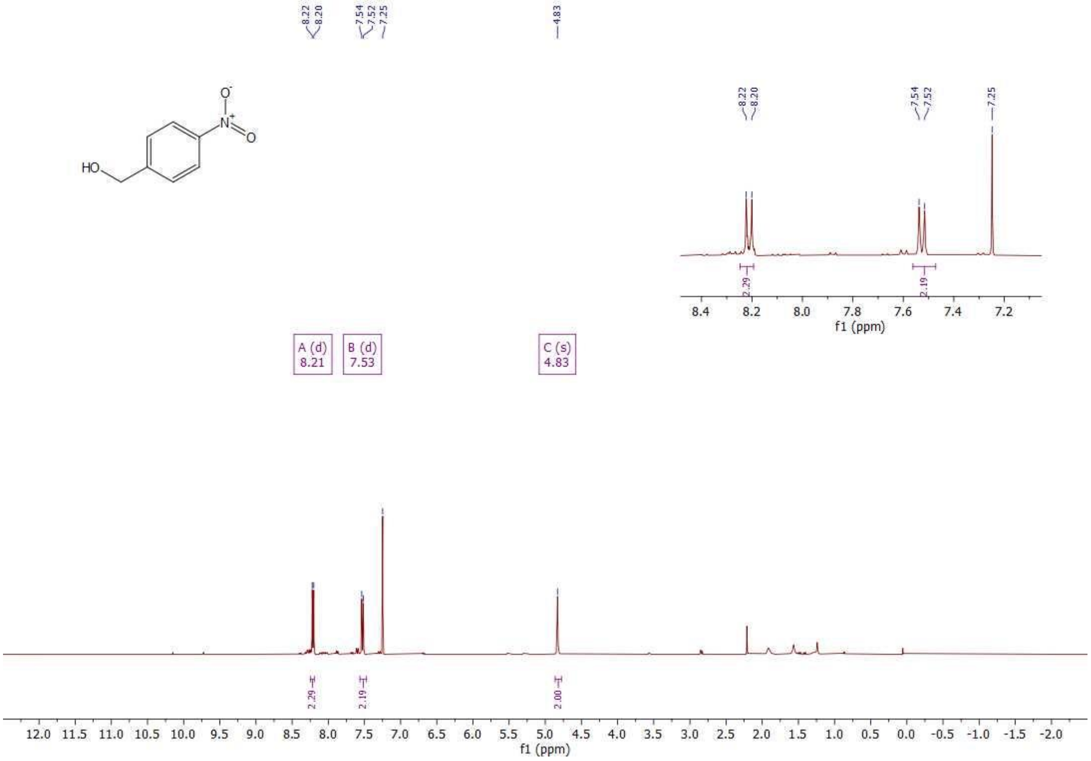

Figure S189. ^{1}H NMR spectrum of **36c** (400 MHz, $CDCl_3$).

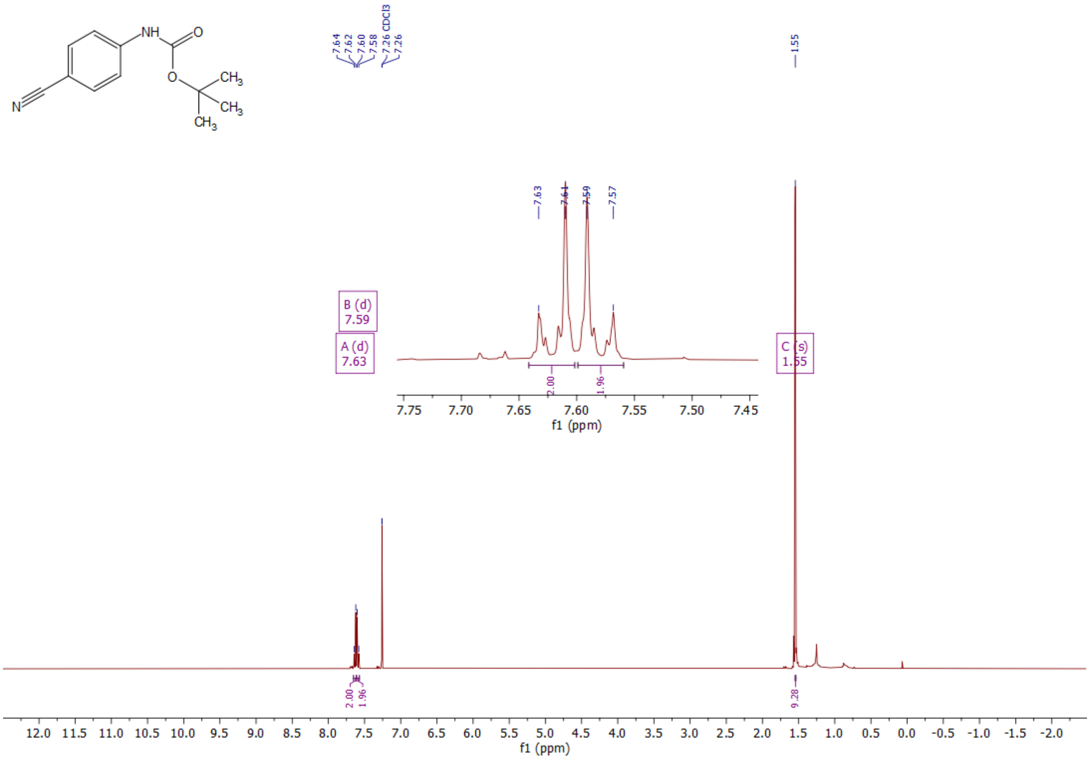

Figure S190. ^{1}H NMR spectrum of **37b** (400 MHz, $CDCl_3$).

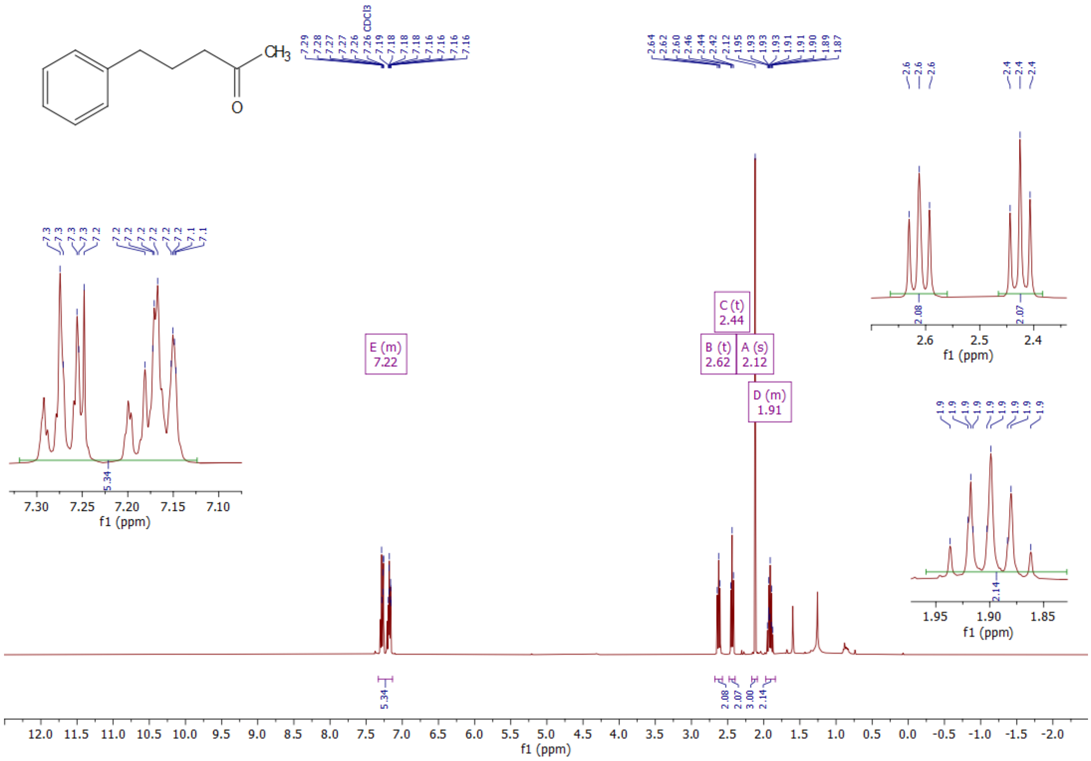

Figure S191. ^{1}H NMR spectrum of **38b** (400 MHz, $CDCl_3$).

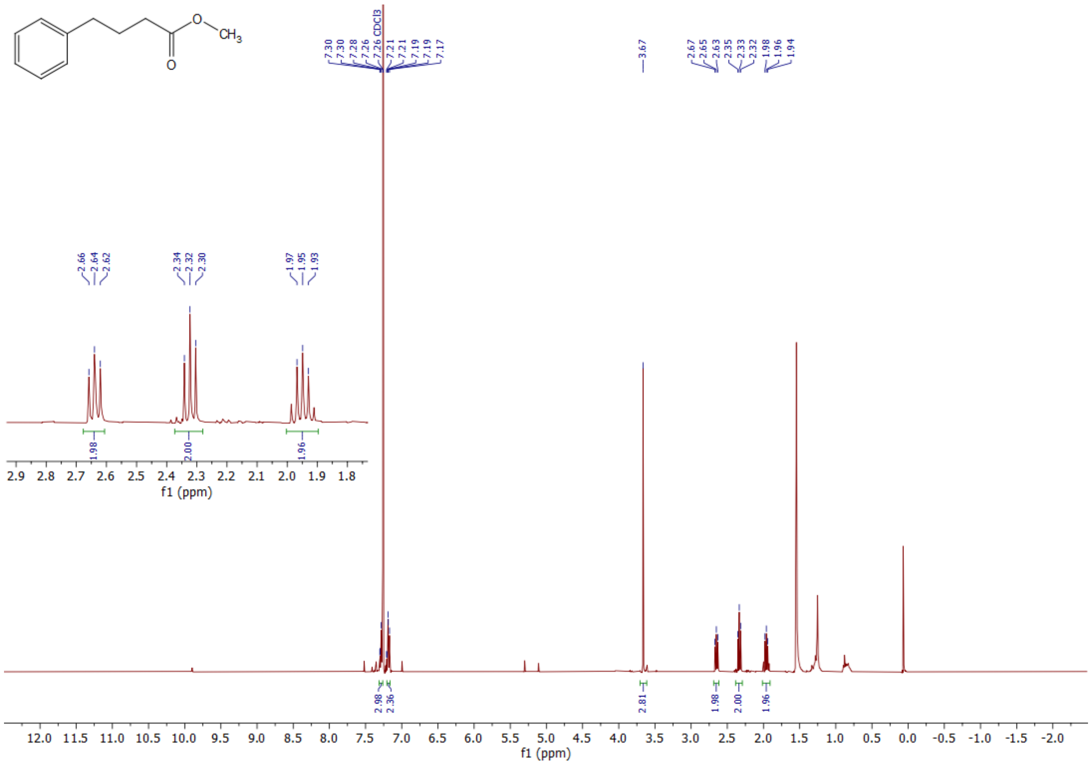

Figure S192. ^{1}H NMR spectrum of **39b** (400 MHz, $CDCl_3$).

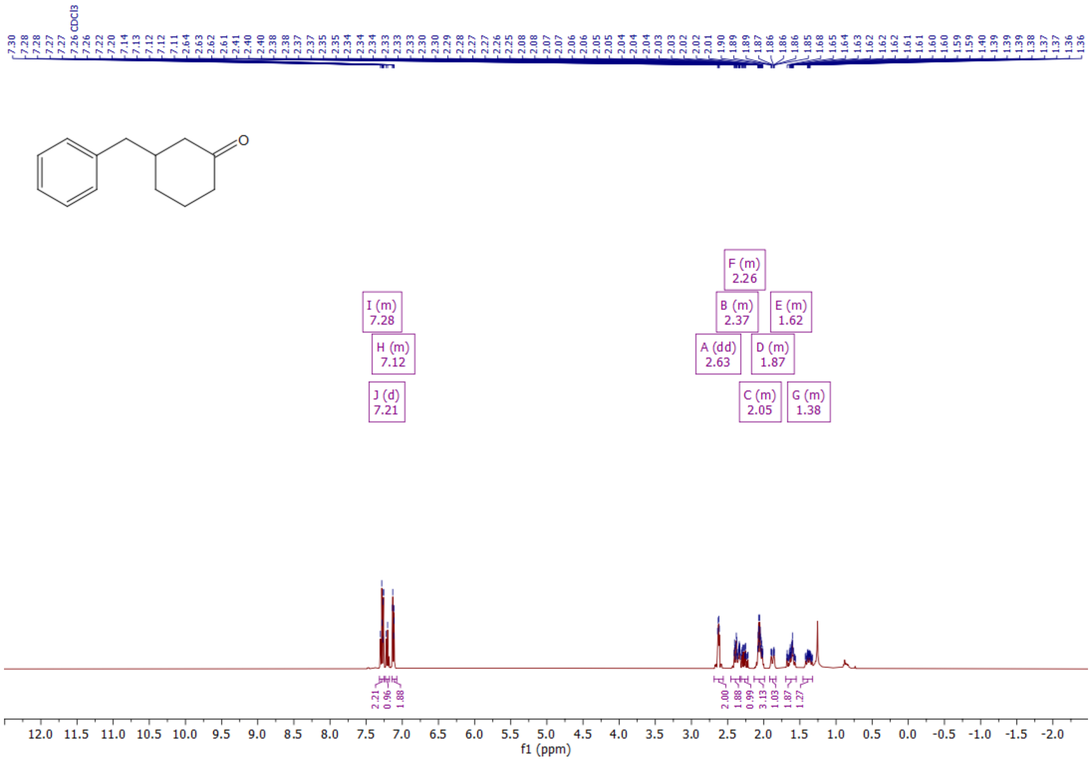

Figure S193. ^{1}H NMR spectrum of **40b** (400 MHz, $CDCl_3$).

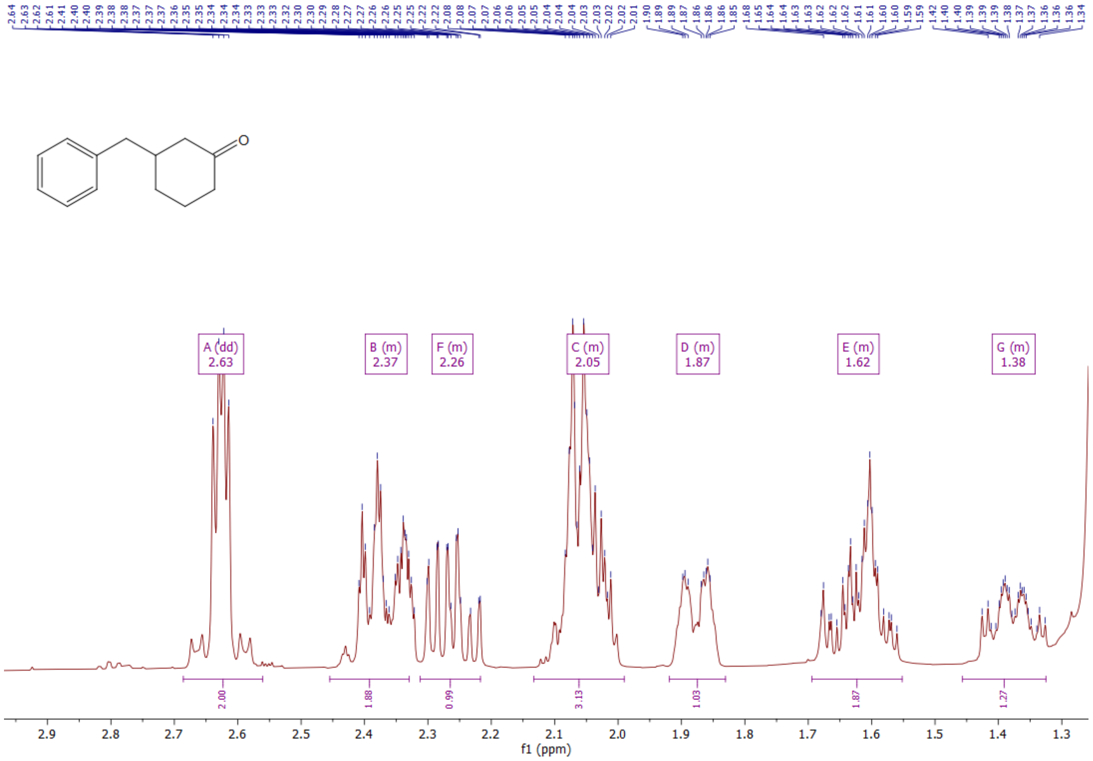

Figure S194. Expansion of ^{1}H NMR spectrum of **40b** (400 MHz, $CDCl_3$).

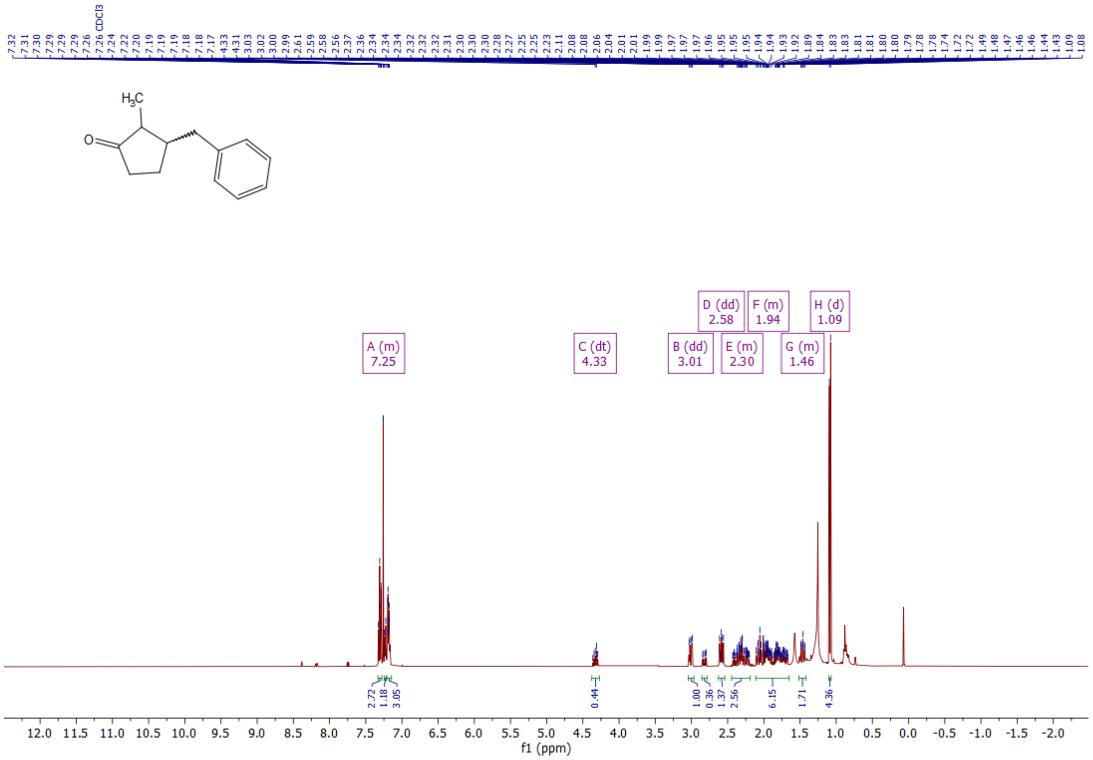

Figure S195. ^{1}H NMR spectrum of **41b** (400 MHz, $CDCl_3$).

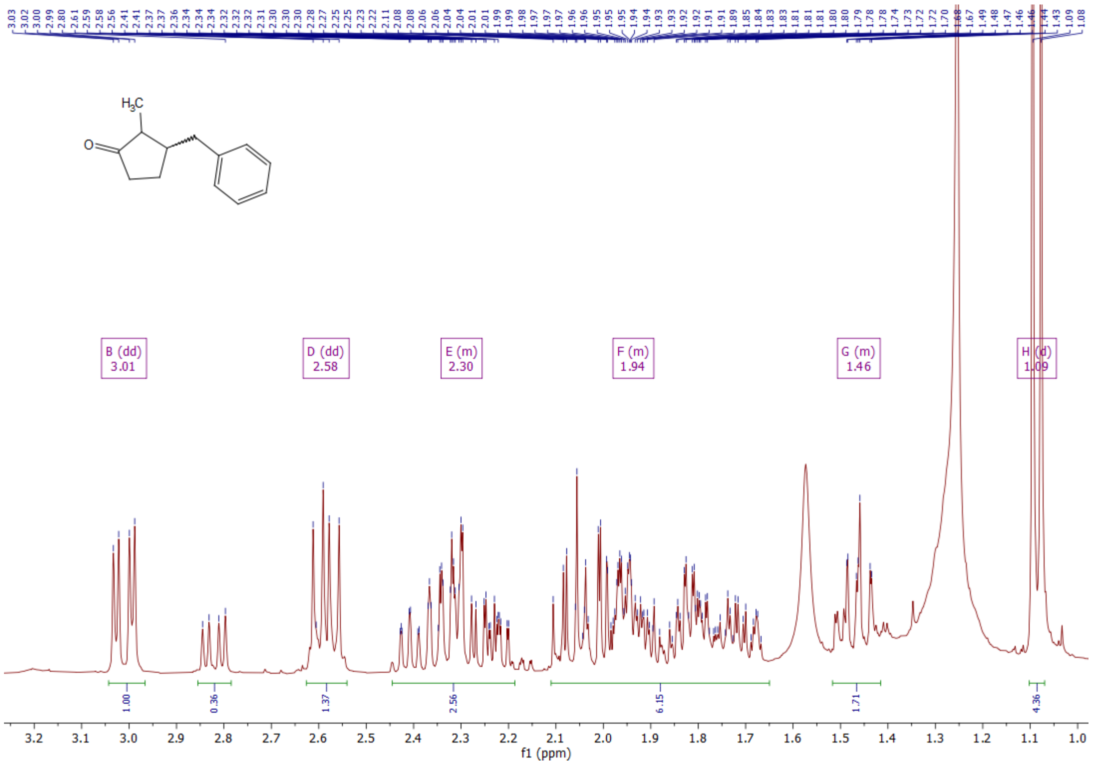

Figure S196. Expansion of ^{1}H NMR spectrum of **41b** (400 MHz, $CDCl_3$).

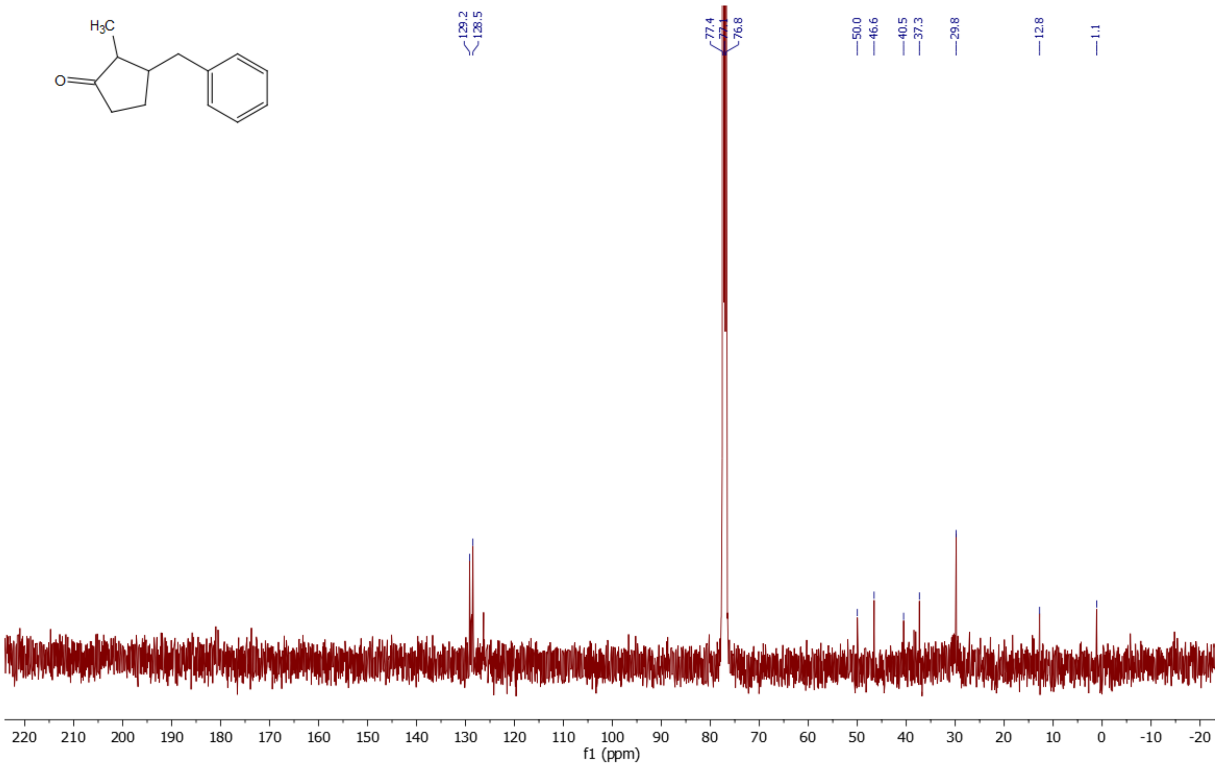

Figure S197. Expansion of ^{13}C NMR spectrum of **41b** (400 MHz, $CDCl_3$).

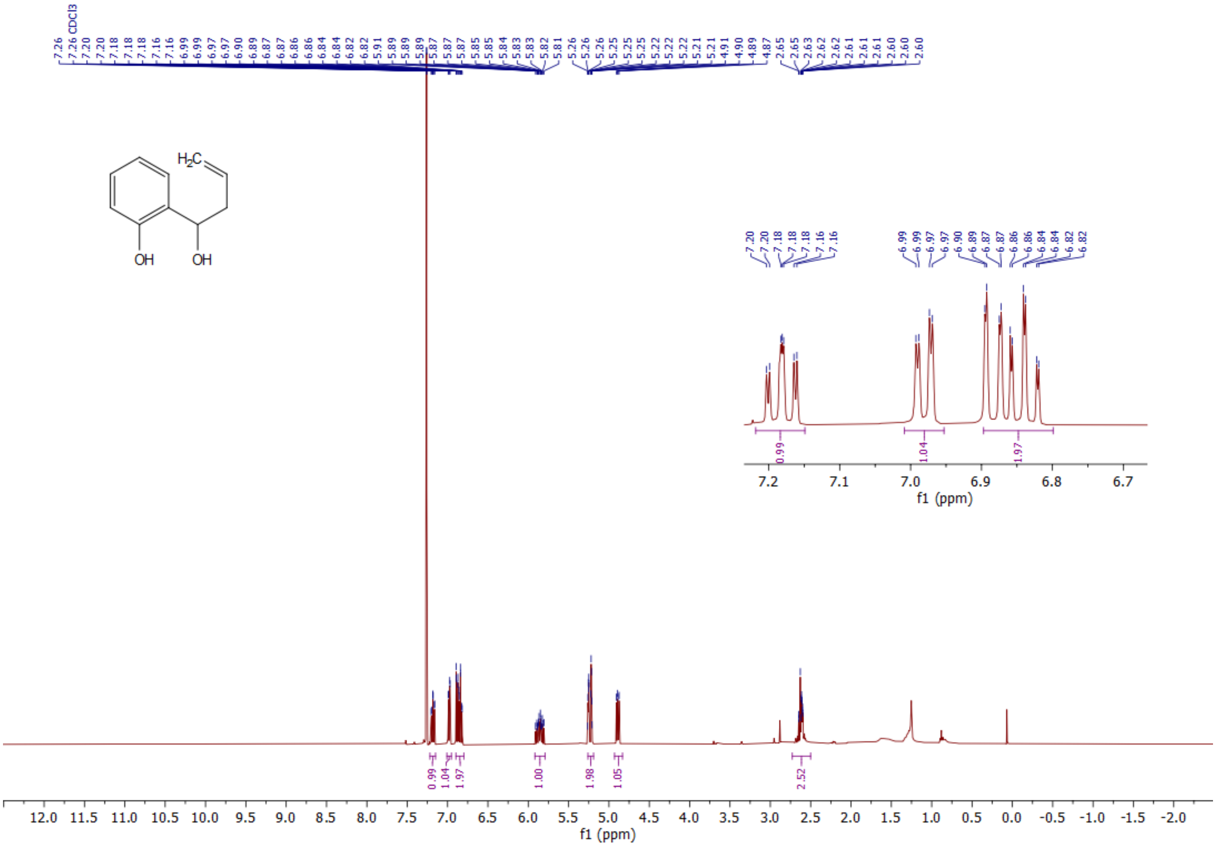

Figure S198. ^1H NMR spectrum of **42c** (400 MHz, $CDCl_3$).

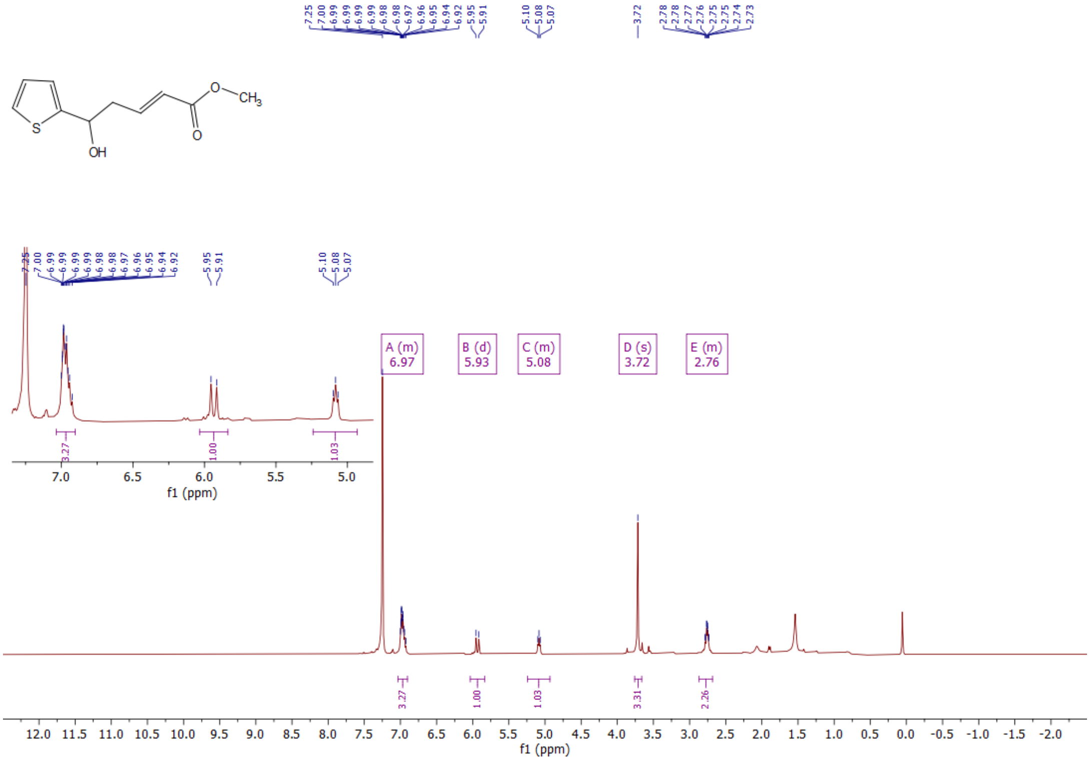

Figure S199. ^1H NMR spectrum of **43c** (400 MHz, $CDCl_3$).

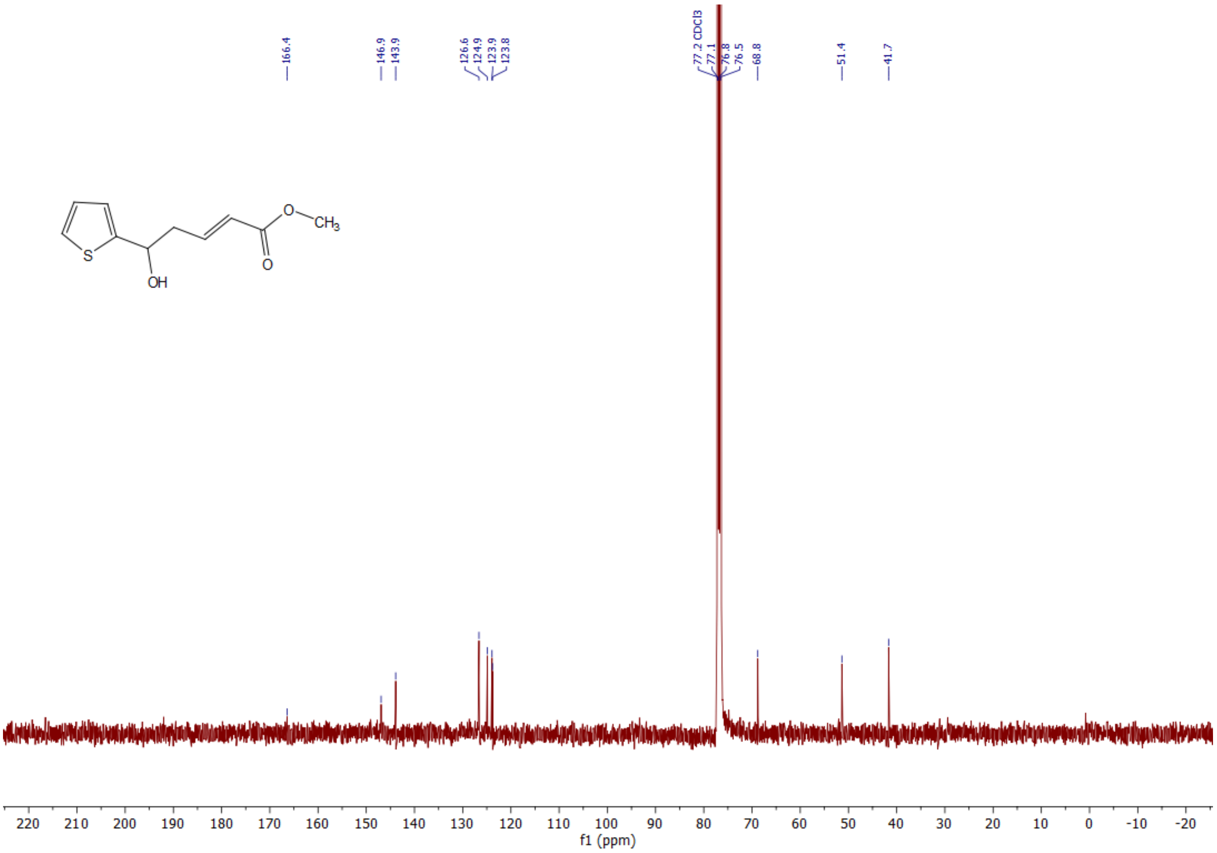

Figure S200. ^{13}C NMR spectrum of **43c** (101 MHz, $CDCl_3$).

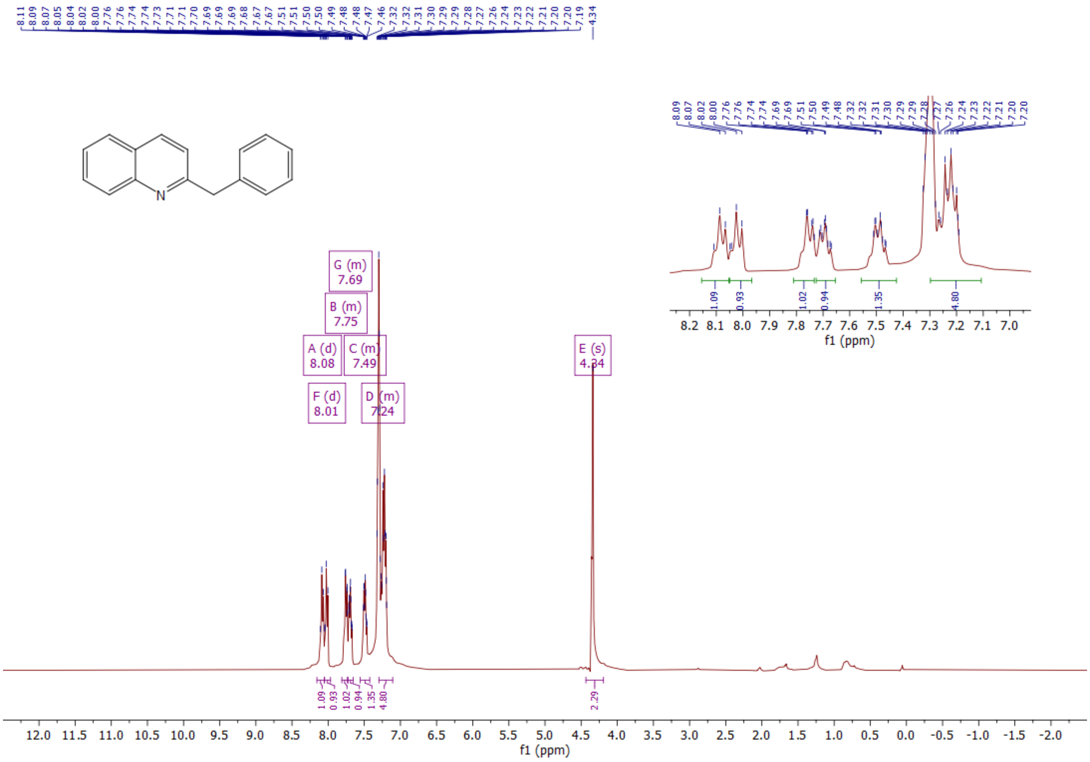

Figure S201. ^{1}H NMR spectrum of **44b** (400 MHz, $CDCl_3$).

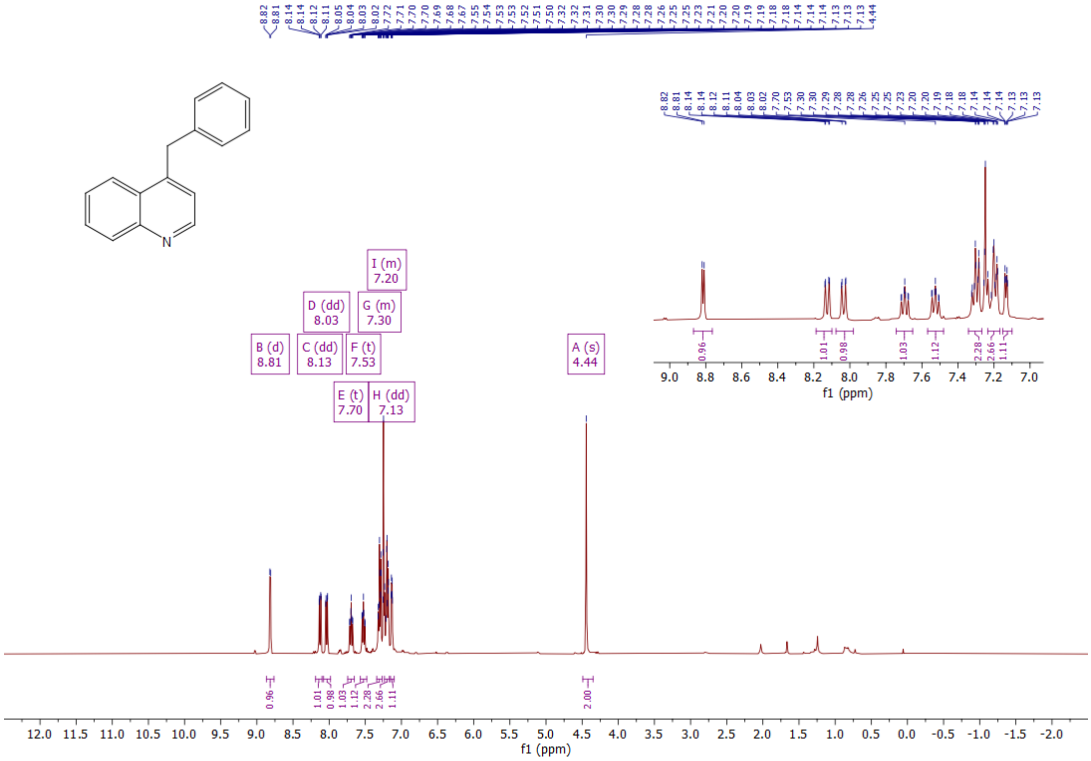

Figure S202. ^{1}H NMR spectrum of **44c** (400 MHz, $CDCl_3$).

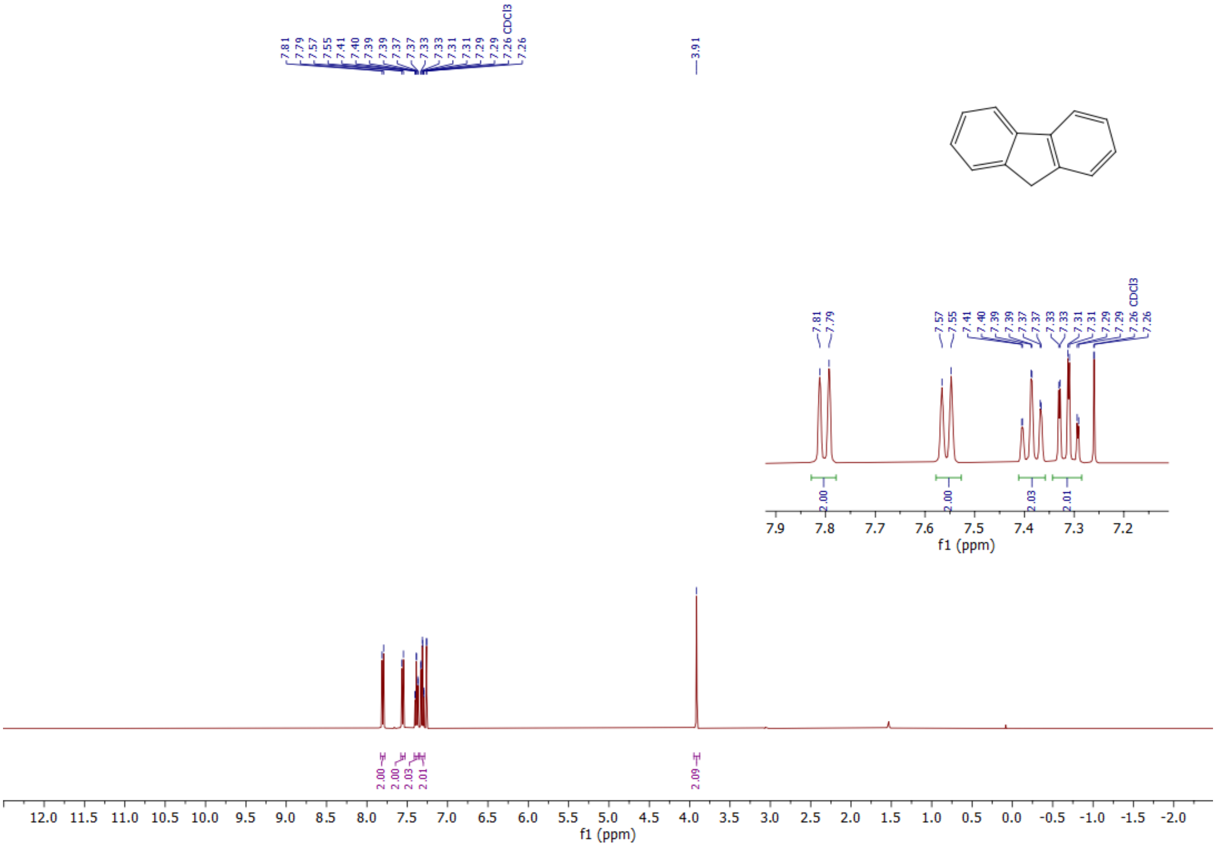

Figure S203. ^{1}H NMR spectrum of **45b** (400 MHz, $CDCl_3$).

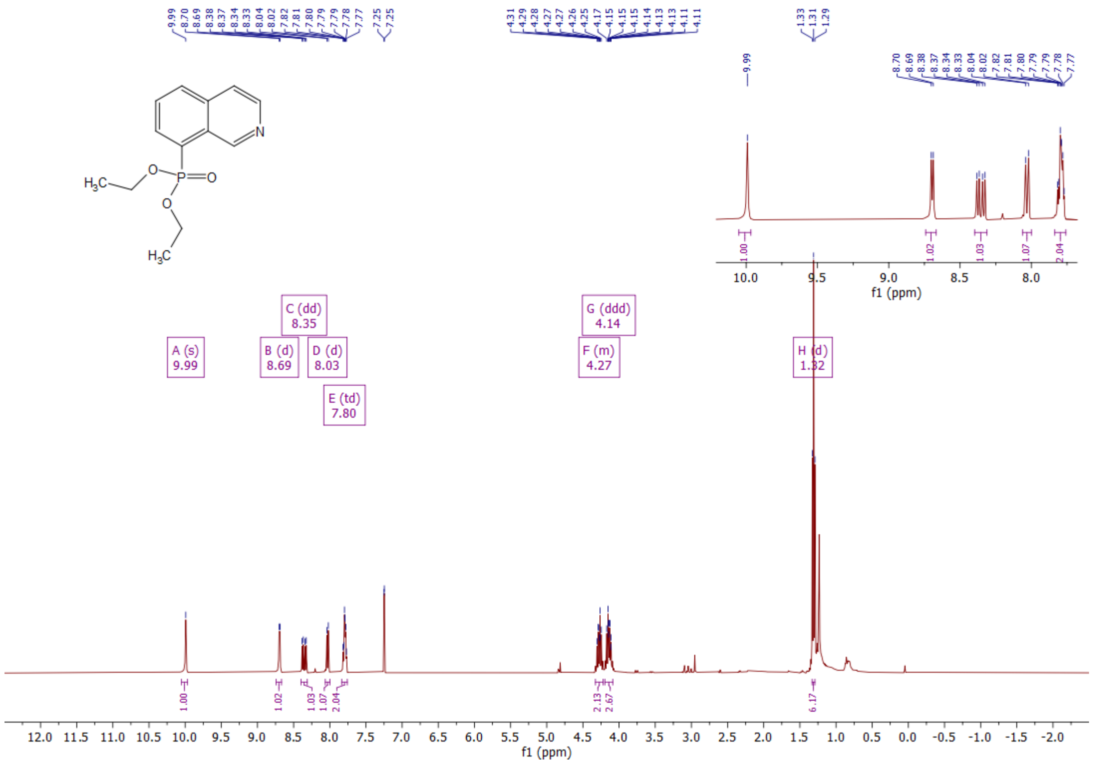

Figure S204. ^{1}H NMR spectrum of **47b** (400 MHz, $CDCl_3$).

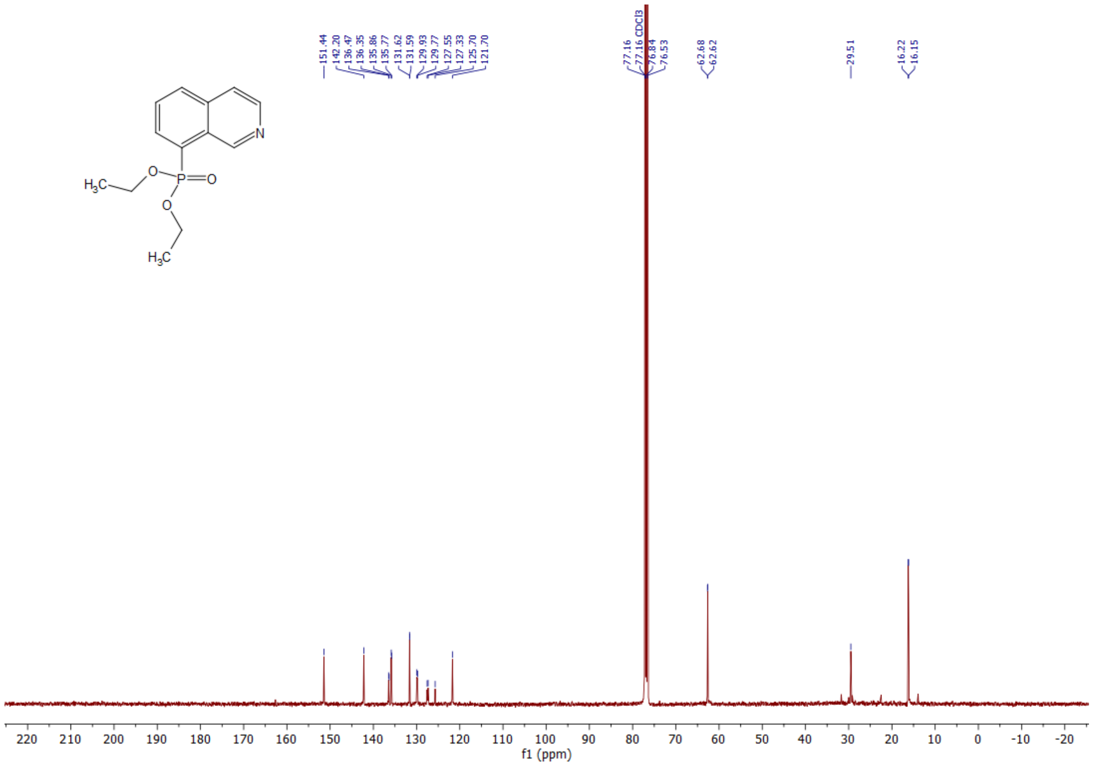

Figure S205. ^{13}C NMR spectrum of **47b** (101 MHz, $CDCl_3$).

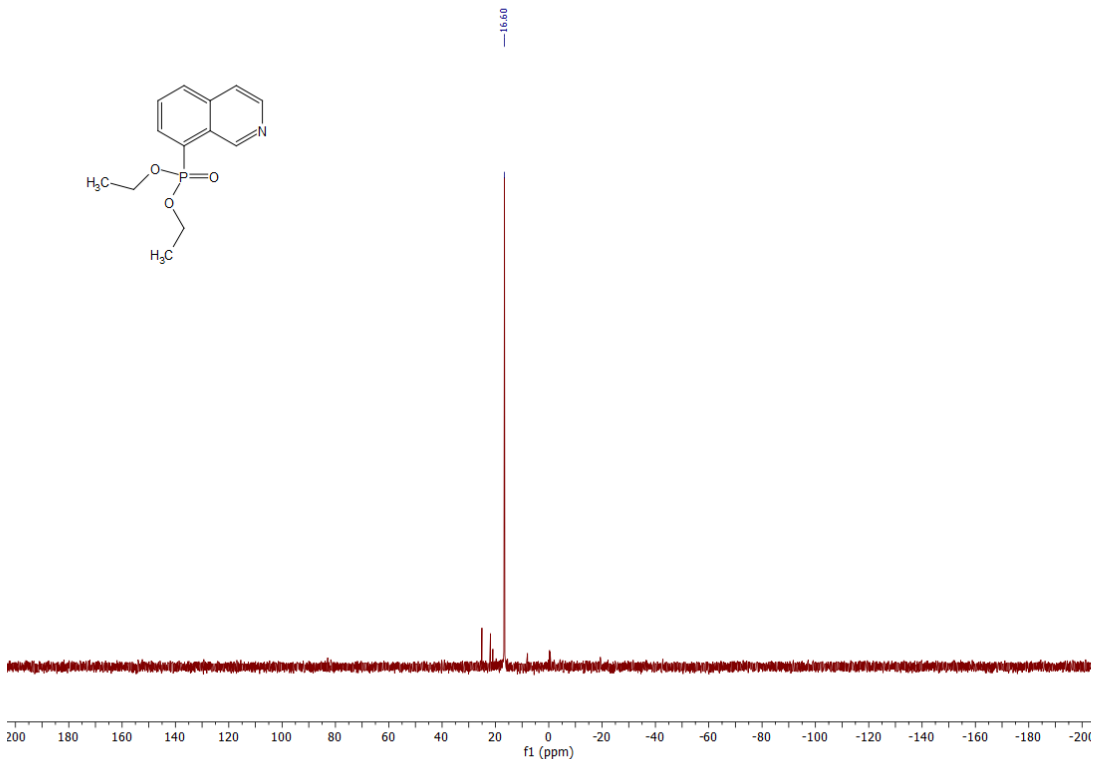

Figure S206. ^{31}P NMR spectrum of **47b** (162 MHz, $CDCl_3$).

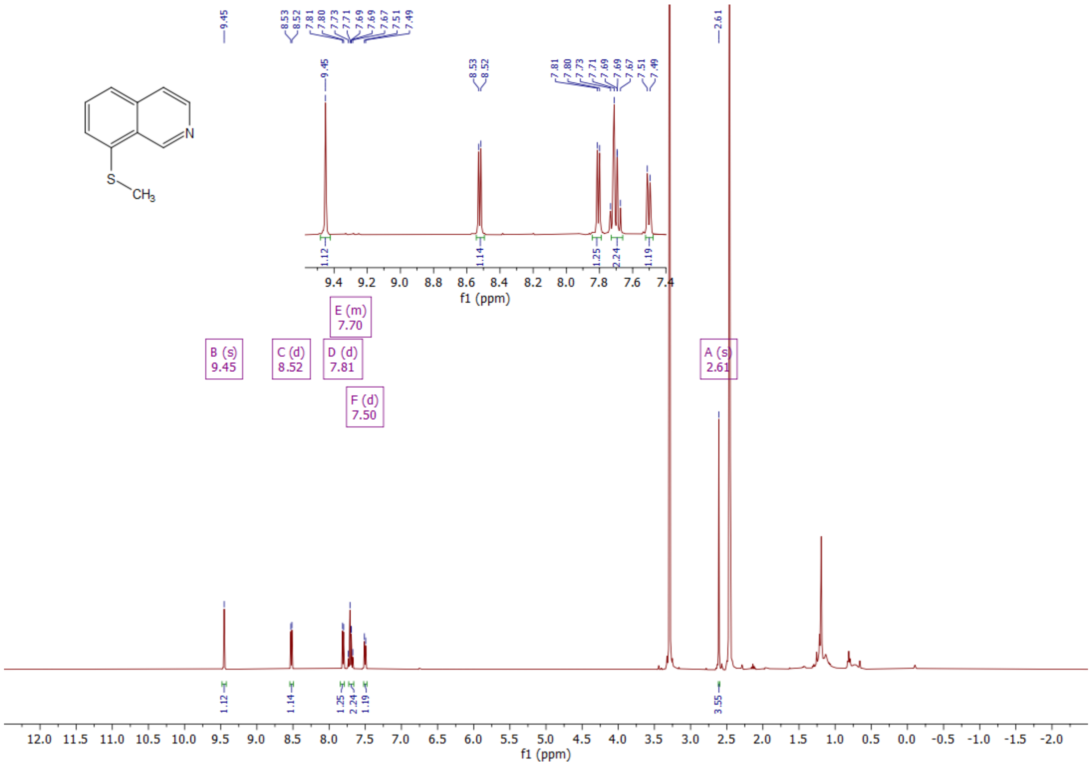

Figure S207. ^{1}H NMR spectrum of **47c** (400 MHz, DMSO-d_6).

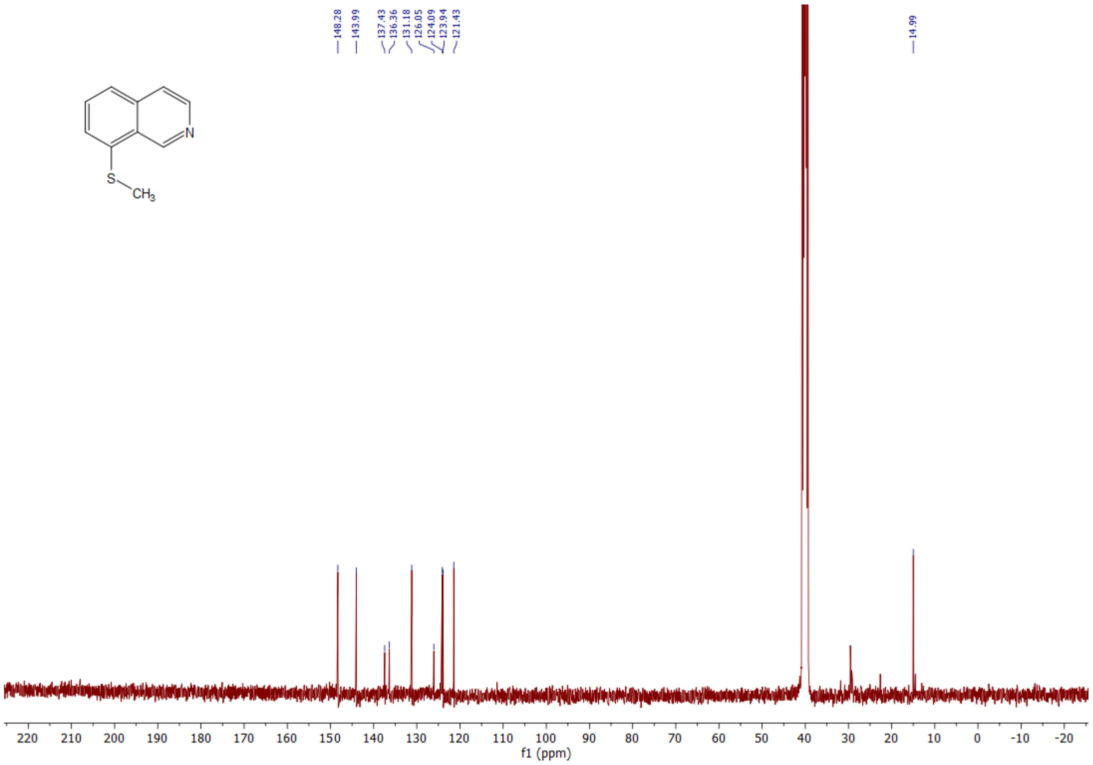

Figure S208. ^{13}C NMR spectrum of **47c** (101 MHz, DMSO-d_6).

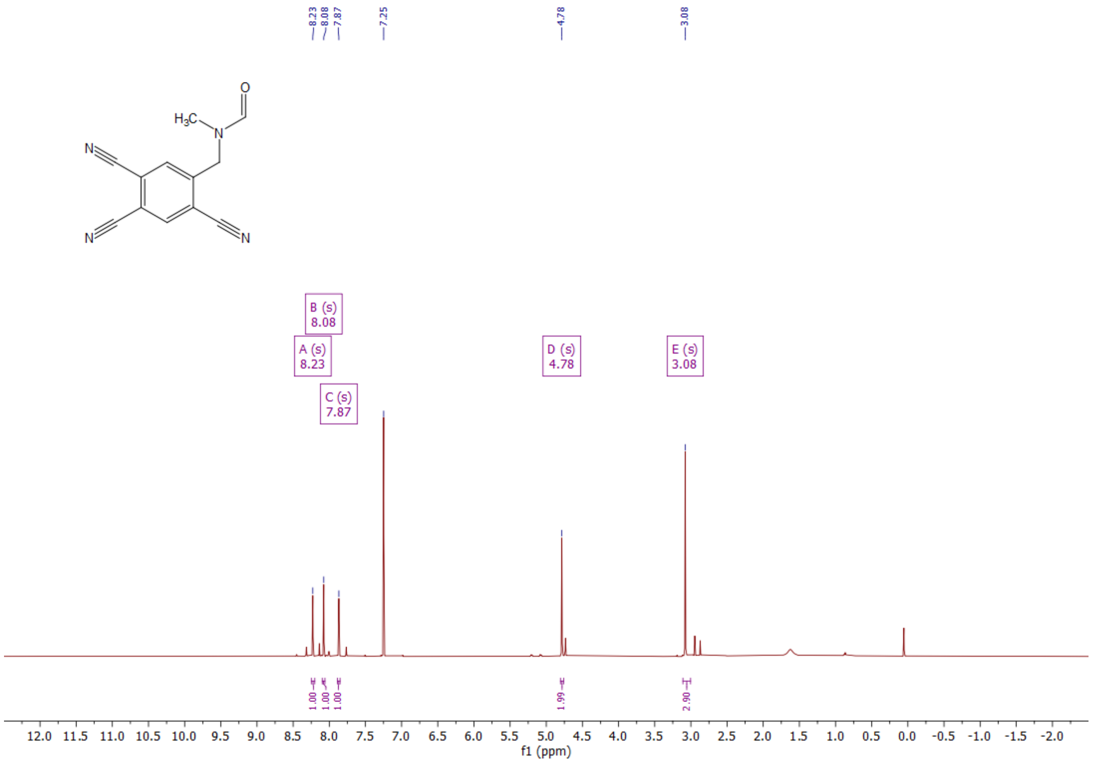

Figure S209. ^{1}H NMR spectrum of **48b** (400 MHz, $CDCl_3$).

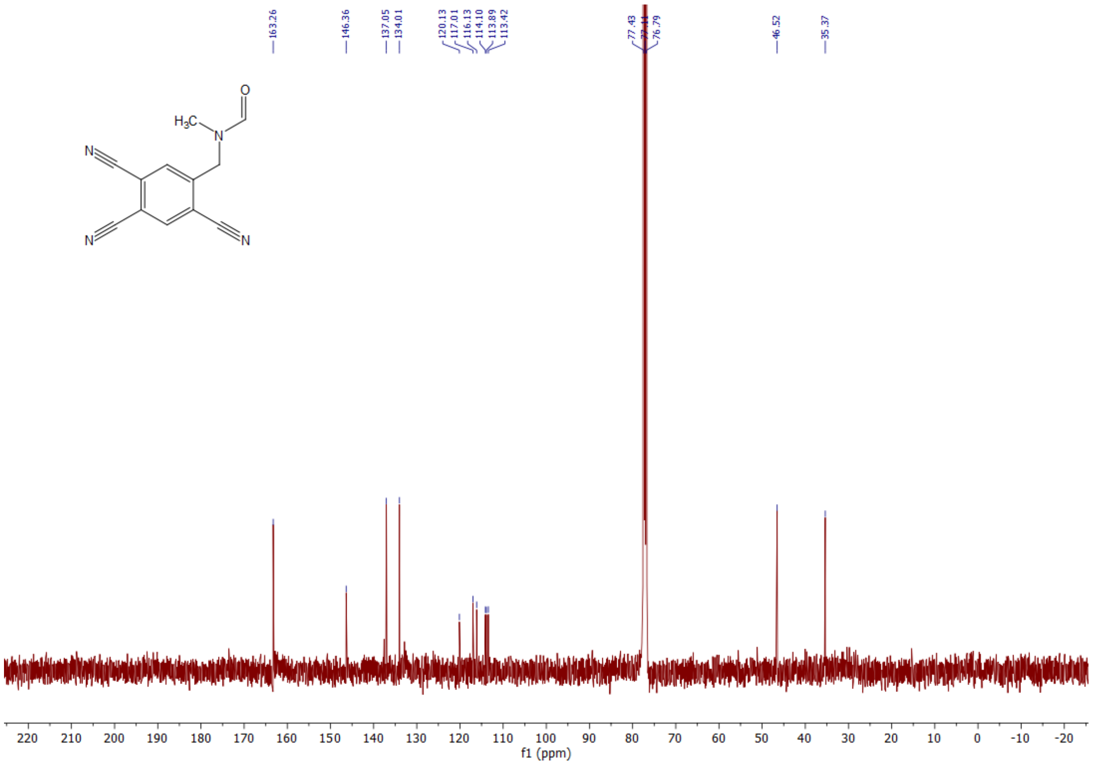

Figure S210. ^{13}C NMR spectrum of **48b** (101 MHz, $CDCl_3$).

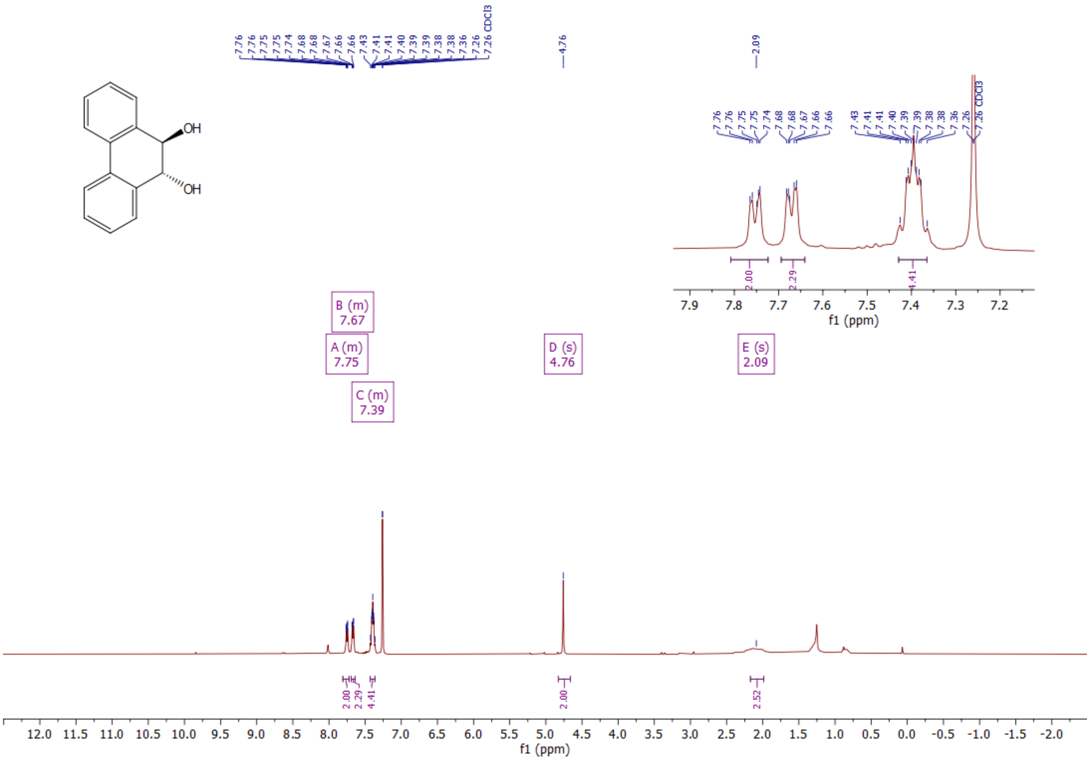

Figure S211. ^{1}H NMR spectrum of **49b** (400 MHz, $CDCl_3$).

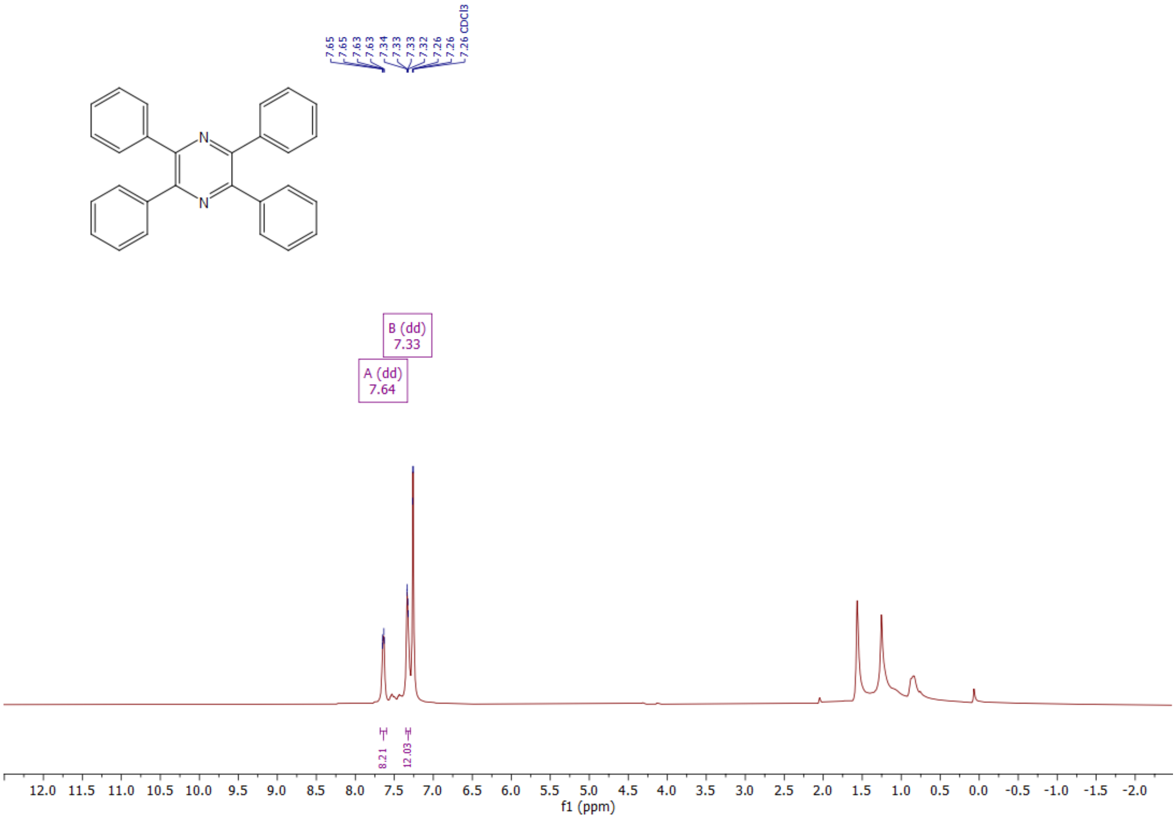

Figure S212. ^{1}H NMR spectrum of **50b** (400 MHz, $CDCl_3$).

References

1 Grant, J.-A. A. *et al.* Synthesis, pharmacological studies and molecular modeling of some tetracyclic 1,3-diazepinium chlorides. *Bioorg. Med. Chem.* **18**, 909-921 (2010).

2 Mohamadi, A. & Miller, L. W. Brightly Luminescent and Kinetically Inert Lanthanide Bioprobes Based on Linear and Preorganized Chelators. *Bioconjugate Chem.* **27**, 2540-2548 (2016).

3 Lee, C.-H., Lee, S.-M., Min, B.-H., Kim, D.-S. & Jun, C.-H. Ferric(III) Chloride Catalyzed Halogenation Reaction of Alcohols and Carboxylic Acids Using α,α-Dichlorodiphenylmethane. *Org. Lett.* **20**, 2468-2471 (2018).

4 Suzuki, K. *et al.* Reevaluation of absolute luminescence quantum yields of standard solutions using a spectrometer with an integrating sphere and a back-thinned CCD detector. *Phys. Chem. Chem. Phys.* **11**, 9850-9860 (2009).

5 Kumashiro, M., Ohsawa, K. & Doi, T. Photocatalyzed Oxidative Decarboxylation Forming Aminovinylcysteine Containing Peptides. *Catalysts* **12**, 1615 (2022).

6 Masilamani, D., Lucas, M. E. & Hammond, G. S. Ion-selective fluorogenic reagents. US5136033 (1992).

7 D'Aleo, A., Moore, E. G., Xu, J., Daumann, L. J. & Raymond, K. N. Optimization of the Sensitization Process and Stability of Octadentate Eu(III) 1,2-HOPO Complexes. *Inorg. Chem.* **54**, 6807-6820 (2015).

8 Baryza, J. L. *et al.* Preparation of RNA conjugates to mediate RNA interference and to treat hepatitis B virus. WO2015050871 (2015).

9 Katritzky, A. R., Wu, J., Wrobel, L., Rachwal, S. & Steel, P. J. Novel conversions of benzotriazol-1-ylmethyl derivatives. *Acta Chem. Scand.* **47**, 167 (1993).

10 Arsenjans, P. *et al.* Preparation of ethynylxanthines as calcium ion channel modulators. WO2016159747 (2016).

11 Tomé, J. P. C. *et al.* Synthesis and Photophysical Studies of New Porphyrin–Phthalocyanine Dyads with Hindered Rotation. *Eur. J. Org. Chem.* **2006**, 257-267 (2006).

12 Sahoo, S. K. An unprecedented oxidative intermolecular homo coupling reaction between two sp3C–sp3C centers under metal-free condition. *Tetrahedron Lett.* **57**, 3476-3480 (2016).

13 Boldt, A. M. *et al.* Reactions of benzyltriphenylphosphonium salts under photoredox catalysis. *Org. Biomol. Chem.* **19**, 7810-7815 (2021).

14 Tomizawa, T., Orimoto, K., Niwa, T. & Nakada, M. Preparation of Imides via the Palladium-Catalyzed Coupling Reaction of Organoborons with Methyl N-[Methoxy(methylthio)methylene]carbamate as a One-Carbon Elongation Reaction. *ChemInform* **44** (2013).

15 Park, G., Yi, S. Y., Jung, J., Cho, E. J. & You, Y. Mechanism and Applications of the Photoredox Catalytic Coupling of Benzyl Bromides. *Chem. Eur. J.* **22**, 17790-17799 (2016).

16 Sekine, Y. & Boekelheide, V. A study of the synthesis and properties of [26](1,2,3,4,5,6)cyclophane (superphane). *J. Am. Chem. Soc.* **103**, 1777-1785 (1981).

17 Siemiaszko, G. & Six, Y. Can the $Ti(OiPr)_4$/nBuLi combination of reagents function as a catalyst for [2+2+2] alkyne cyclotrimerisation reactions? *New J. Chem.* **42**, 20219-20226 (2018).

18 Chen, T., Yang, L., Li, L. & Huang, K.-W. Homocoupling of benzyl halides catalyzed by POCOP–nickel pincer complexes. *Tetrahedron* **68**, 6152-6157 (2012).

19 Ding, Y., Luo, S., Ma, L. & An, J. Reductive Cleavage of Unactivated Carbon–Cyano Bonds under Ammonia-Free Birch Conditions. *J. Org. Chem.* **84**, 15827-15833 (2019).

20 Pace, V. *et al.* Bromomethyllithium-mediated chemoselective homologation of disulfides to dithioacetals. *Chem. Commun.* **52**, 2639-2642 (2016).
21 Montiel, L. E., Zepeda, L. G. & Tamariz, J. Efficient Total Synthesis of Racemic Bisabolane Sesquiterpenes Curcuphenol and Xanthorrhizol Starting from Substituted Acetophenones. *Helv. Chim. Acta* **93**, 1261-1273 (2010).
22 Moores, L. C., Kaur, D., Smith, M. D. & Poole, J. S. Regioselectivity of Hydroxyl Radical Reactions with Arenes in Nonaqueous Solutions. *J. Org. Chem.* **84**, 3260-3269 (2019).
23 Sun, Y., Jiang, H., Wu, W., Zeng, W. & Wu, X. Copper-Catalyzed Synthesis of Substituted Benzothiazoles via Condensation of 2-Aminobenzenethiols with Nitriles. *Org. Lett.* **15**, 1598-1601 (2013).
24 Zou, X., Zou, J., Yang, L., Li, G. & Lu, H. Thermal Rearrangement of Sulfamoyl Azides: Reactivity and Mechanistic Study. *J. Org. Chem.* **82**, 4677-4688 (2017).
25 Singh, D. & Silakari, O. Sodium hydrogen exchanger inhibitory activity of benzotriazole derivatives. *Eur. J. Med. Chem.* **126**, 183-189 (2017).
26 Pang, Y., Leutzsch, M., Nöthling, N., Katzenburg, F. & Cornella, J. Catalytic Hydrodefluorination via Oxidative Addition, Ligand Metathesis, and Reductive Elimination at Bi(I)/Bi(III) Centers. *J. Am. Chem. Soc.* **143**, 12487-12493 (2021).
27 Yan, Z., Yuan, X.-A., Zhao, Y., Zhu, C. & Xie, J. Selective Hydroarylation of 1,3-Diynes Using a Dimeric Manganese Catalyst: Modular Synthesis of Z-Enynes. *Angew. Chem. Int. Ed.* **57**, 12906-12910 (2018).
28 Ji, S. *et al.* Confined Pyrolysis within Metal–Organic Frameworks To Form Uniform Ru3 Clusters for Efficient Oxidation of Alcohols. *J. Am. Chem. Soc.* **139**, 9795-9798 (2017).
29 Edmunds, A. *et al.* Preparation of pyridyl ketones as herbicides. WO2000015615 (2000).
30 Watterson, S. H. *et al.* Small Molecule Antagonist of Leukocyte Function Associated Antigen-1 (LFA-1): Structure−Activity Relationships Leading to the Identification of 6-((5S,9R)-9-(4-Cyanophenyl)-3-(3,5-dichlorophenyl)-1-methyl-2,4-dioxo-1,3,7-triazaspiro[4.4]nonan-7-yl)nicotinic Acid (BMS-688521). *J. Med. Chem.* **53**, 3814-3830 (2010).
31 Xu, P. & Xu, H. C. Electrochemical Deoxygenation of N-Heteroaromatic N-Oxides. *Synlett* **30**, 1219-1221 (2019).
32 Motoyama, Y., Kamo, K. & Nagashima, H. Catalysis in Polysiloxane Gels: Platinum-Catalyzed Hydrosilylation of Polymethylhydrosiloxane Leading to Reusable Catalysts for Reduction of Nitroarenes. *Org. Lett.* **11**, 1345-1348 (2009).
33 Lubriks, D., Sokolovs, I. & Suna, E. Indirect C–H Azidation of Heterocycles via Copper-Catalyzed Regioselective Fragmentation of Unsymmetrical λ^3-Iodanes. *J. Am. Chem. Soc.* **134**, 15436-15442 (2012).
34 Müller, C. E. *et al.* Imidazo[2,1-i]purin-5-ones and Related Tricyclic Water-Soluble Purine Derivatives: Potent A2A- and A3-Adenosine Receptor Antagonists. *J. Med. Chem.* **45**, 3440-3450 (2002).
35 Lindsey, J. S., Schreiman, I. C., Hsu, H. C., Kearney, P. C. & Marguerettaz, A. M. Rothemund and Adler-Longo reactions revisited: synthesis of tetraphenylporphyrins under equilibrium conditions. *J. Org. Chem.* **52**, 827-836 (1987).
36 Zou, Y.-Q. *et al.* Highly Efficient Aerobic Oxidative Hydroxylation of Arylboronic Acids: Photoredox Catalysis Using Visible Light. *Angew. Chem. Int. Ed.* **51**, 784-788 (2012).
37 Wang, F., Planas, O. & Cornella, J. Bi(I)-Catalyzed Transfer-Hydrogenation with Ammonia-Borane. *J. Am. Chem. Soc.* **141**, 4235-4240 (2019).

38 Semwal, S. & Choudhury, J. Switch in Catalyst State: Single Bifunctional Bi-state Catalyst for Two Different Reactions. *Angew. Chem. Int. Ed.* **56**, 5556-5560 (2017).
39 Dąbrowa, K., Niedbała, P. & Jurczak, J. Anion-tunable control of thermal Z→E isomerisation in basic azobenzene receptors. *Chem. Commun.* **50**, 15748-15751 (2014).
40 Lloyd, J. R., Lowther, N., Zsabo, G. & Hall, C. D. Kinetics and mechanism of the reaction of trico-ordinate phosphorus compounds with octasulphur. *J. Chem. Soc., Perkin Trans. 2*, 1813-1817 (1985).
41 Speckmeier, E., Fischer, T. G. & Zeitler, K. A Toolbox Approach To Construct Broadly Applicable Metal-Free Catalysts for Photoredox Chemistry: Deliberate Tuning of Redox Potentials and Importance of Halogens in Donor–Acceptor Cyanoarenes. *J. Am. Chem. Soc.* **140**, 15353-15365 (2018).
42 Yu, J., Zhang, P., Wu, J. & Shang, Z. Metal-free C–N bond-forming reaction: straightforward synthesis of anilines, through cleavage of aryl C–O bond and amide C–N bond. *Tetrahedron Lett.* **54**, 3167-3170 (2013).
43 Liu, C. *et al.* Palladium-Catalyzed Aerobic Oxidative Direct Esterification of Alcohols. *Angew. Chem. Int. Ed.* **50**, 5144-5148 (2011).
44 Galbiati, A. *et al.* Development of Potent 3-Br-isoxazoline-Based Antimalarial and Antileishmanial Compounds. *ACS Med. Chem. Lett.* **12**, 1726-1732 (2021).
45 Moriyama, K., Takemura, M. & Togo, H. Selective Oxidation of Alcohols with Alkali Metal Bromides as Bromide Catalysts: Experimental Study of the Reaction Mechanism. *J. Org. Chem.* **79**, 6094-6104 (2014).
46 Kim, I. & Lee, C. Rhodium-Catalyzed Oxygenative Addition to Terminal Alkynes for the Synthesis of Esters, Amides, and Carboxylic Acids. *Angew. Chem. Int. Ed.* **52**, 10023-10026 (2013).
47 Kim, Y. J. & Kim, D. Y. Electrochemical Radical Selenylation/1,2-Carbon Migration and Dowd–Beckwith-Type Ring-Expansion Sequences of Alkenylcyclobutanols. *Org. Lett.* **21**, 1021-1025 (2019).
48 Huang, J.-M. & Dong, Y. Zn-mediated electrochemical allylation of aldehydes in aqueous ammonia. *Chem. Commun.*, 3943-3945 (2009).
49 Zhou, X. *et al.* Synthesis of 2-Substituted Quinolines via Rhodium(III)-Catalyzed C–H Activation of Imidamides and Coupling with Cyclopropanols. *Adv. Synth. Catal.* **359**, 1620-1625 (2017).
50 Morimoto, K. *et al.* Synthesis of Fluorene Derivatives through Rhodium-Catalyzed Dehydrogenative Cyclization. *Angew. Chem. Int. Ed.* **51**, 5359-5362 (2012).
51 Melzer, B. & Bracher, F. A divergent approach to benzylisoquinoline-type and oxoaporphine alkaloids via regioselective direct ring metalation of alkoxy isoquinolines. *Org. Biomol. Chem.* **13**, 7664-7672 (2015).
52 Tsuji, M., Higashiyama, K., Yamauchi, T., Kubo, H. & Ohmiya, S. Photosubstitution reaction of cyanoaromatics with aliphatic amides. *Heterocycles* **54**, 1027-1032 (2001).
53 Lawlor, D. A. *et al.* Hyperaromatic Stabilization of Arenium Ions: A Remarkable Cis Stereoselectivity of Nucleophilic Trapping of β-Hydroxyarenium Ions by Water. *J. Am. Chem. Soc.* **133**, 19718-19728 (2011).
54 Khafizova, L. O., Shaibakova, M. G., Rikhter, N. A., Tyumkina, T. V. & Dzhemilev, U. M. One-pot synthesis of 2,3,5-substituted 1H-pyrroles via the reaction of terminal acetylenes with nitriles and $EtAlCl_2$ catalyzed by Cp_2TiCl_2. *Tetrahedron* **75**, 906-911 (2019).
55 Jones, W. E., Jr. & Fox, M. A. Determination of Excited-State Redox Potentials by Phase-Modulated Voltammetry. *J. Phys. Chem.* **98**, 5095-5099 (1994).
56 Weller, A. Electron-transfer and complex formation in the excited state. *Pure Appl. Chem.* **16**, 115 (1968).

57 Beeby, A., Faulkner, S. & Williams, J. A. G. pH Dependence of the energy transfer mechanism in a phenanthridine-appended ytterbium complex. *J. Chem. Soc., Dalton Trans.*, 1918-1922 (2002).

58 Katsumi, A. *et al*. Conspicuous effects due to complex formation and contact term upon lanthanide-induced shifts in 19F NMR of some fluoroaromatic compounds. *Chem. Lett.* **2**, 1205-1208 (1973).
